# Environmental Stresses Disrupt Telomere Length Homeostasis

**DOI:** 10.1371/journal.pgen.1003721

**Published:** 2013-09-05

**Authors:** Gal Hagit Romano, Yaniv Harari, Tal Yehuda, Ariel Podhorzer, Linda Rubinstein, Ron Shamir, Assaf Gottlieb, Yael Silberberg, Dana Pe'er, Eytan Ruppin, Roded Sharan, Martin Kupiec

**Affiliations:** 1 Department of Molecular Microbiology and Biotechnology, Tel Aviv University, Tel Aviv, Israel; 2 Blavatnik School of Computer Science, Tel Aviv University, Tel Aviv, Israel; 3 Department of Biological Sciences, Columbia University, New York, New York, United States of America; Chinese Academy of Sciences, China

## Abstract

Telomeres protect the chromosome ends from degradation and play crucial roles in cellular aging and disease. Recent studies have additionally found a correlation between psychological stress, telomere length, and health outcome in humans. However, studies have not yet explored the causal relationship between stress and telomere length, or the molecular mechanisms underlying that relationship. Using yeast as a model organism, we show that stresses may have very different outcomes: alcohol and acetic acid elongate telomeres, whereas caffeine and high temperatures shorten telomeres. Additional treatments, such as oxidative stress, show no effect. By combining genome-wide expression measurements with a systematic genetic screen, we identify the Rap1/Rif1 pathway as the central mediator of the telomeric response to environmental signals. These results demonstrate that telomere length can be manipulated, and that a carefully regulated homeostasis may become markedly deregulated in opposing directions in response to different environmental cues.

## Author Summary

Over 70 years ago, Barbara McClintock described telomeres and hypothesized about their role in protecting the integrity of chromosomes. Since then, scientists have shown that telomere length is highly regulated and associated with cell senescence and longevity, as well as with age-related disorders and cancer. Here, we show that despite their importance, the tight, highly complex regulation of telomeres may be disrupted by environmental cues, leading to changes in telomere length. We have introduced yeast cells to 13 different environmental stresses to show that some stresses directly alter telomere length. Our results indicate that alcohol and acetic acid elongate telomeres, while caffeine and high temperatures shorten telomeres. Using expression data, bioinformatics tools, and a large genetic screen, we explored the mechanisms responsible for the alterations of telomere length under several stress conditions. We identify Rap1 and Rif1, central players in telomere length maintenance, as the central proteins directly affected by external cues that respond by altering telomere length. Because many human diseases are related to alterations in telomere length that fuel the disease's pathology, controlling telomere length by manipulating simple stressing agents may point the way to effective treatment, and will supply scientists with an additional tool to study the machinery responsible for telomere length homeostasis.

## Introduction

Telomeres are nucleoprotein structures located at the ends of chromosomes. Telomeres are essential for chromosome replication and stability [1], and protect chromosome ends from degradation and deleterious chromosomal rearrangements [1], [2]. In human embryonic cells, telomeres are elongated by the enzyme telomerase [3]. In somatic cells, however, telomerase expression is low, and telomeres shorten with each cell division due to the incomplete replication of the linear chromosome ends by conventional DNA polymerases. This progressive telomere shortening constitutes a “molecular clock” that underlies cellular aging [4]. Accordingly, telomere length is associated with cell senescence and longevity [5], as well as with age-related disorders and cancer [6]. While short telomeres have been reported to predict early mortality [7], recent work has shown that telomerase reactivation may reverse tissue degeneration in aged telomerase-deficient mice [8].

Three systematic genome-wide surveys in the yeast *Saccharomyces cerevisiae* [9]–[11] have revealed that mutations in at least 6% of the genes lead to alterations of telomere length. These *TLM* (*Telomere Length Maintenance*) genes span a broad range of functional categories and different cellular compartments. Integration of data from these large-scale mutant screens with information about protein–protein interactions has further permitted charting of the cellular sub-network underlying telomere length regulation in yeast [12], [13], revealing a complex set of interactions responsible for a very tight length homeostasis.

Environmental stresses affect the regulation and the activity of many genes and accordingly may perturb telomere length homeostasis by altering the expression or activity of genes in the TLM network described above. Previous studies have suggested that emotional stress in humans is associated with telomere shortening, presumably through its effect on oxidative stress [14], [15]. These studies, however, establish a correlation, but not causality. Here, we use controlled experimental approaches to explore a possible effect of the environment on yeast telomere length, and to identify the molecular mechanisms by which external signals exert their effect.

## Results and Discussion

### Environmental signals can affect telomere length

**Figure 1:**
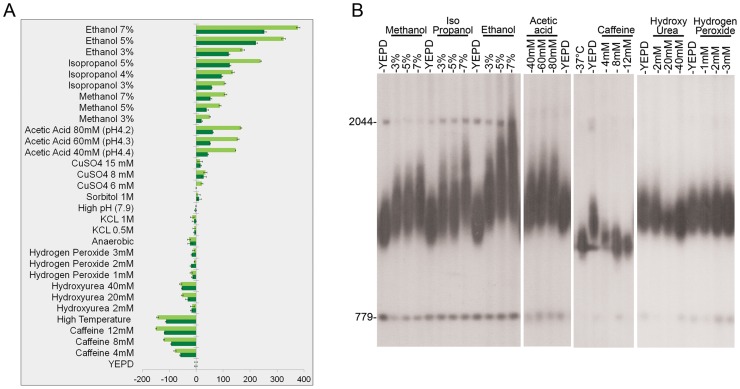
The effect of environmental stress on telomere length. Strain BY4741 was grown for 100 generations on YEPD at optimal conditions and under mild stress conditions. DNA was extracted after 50 and 100 generations, digested with *Xho*I, and analyzed by Southern blot. The membrane was probed with a telomere sequence and with unique genomic sequences used as markers (779 bp and 2044 bp) to enable telomere length measurements. Telomere length was measured for at least three independent colonies. **A**. The difference (in bp) between telomere length grown under stress and that of cells grown on YEPD is represented by dark green bars (after 50 generations) and by light green bars (after 100 generations). **B**. Southern blots analyses after 100 generations showing the effect of various stresses on telomere length.

**Figure 2:**
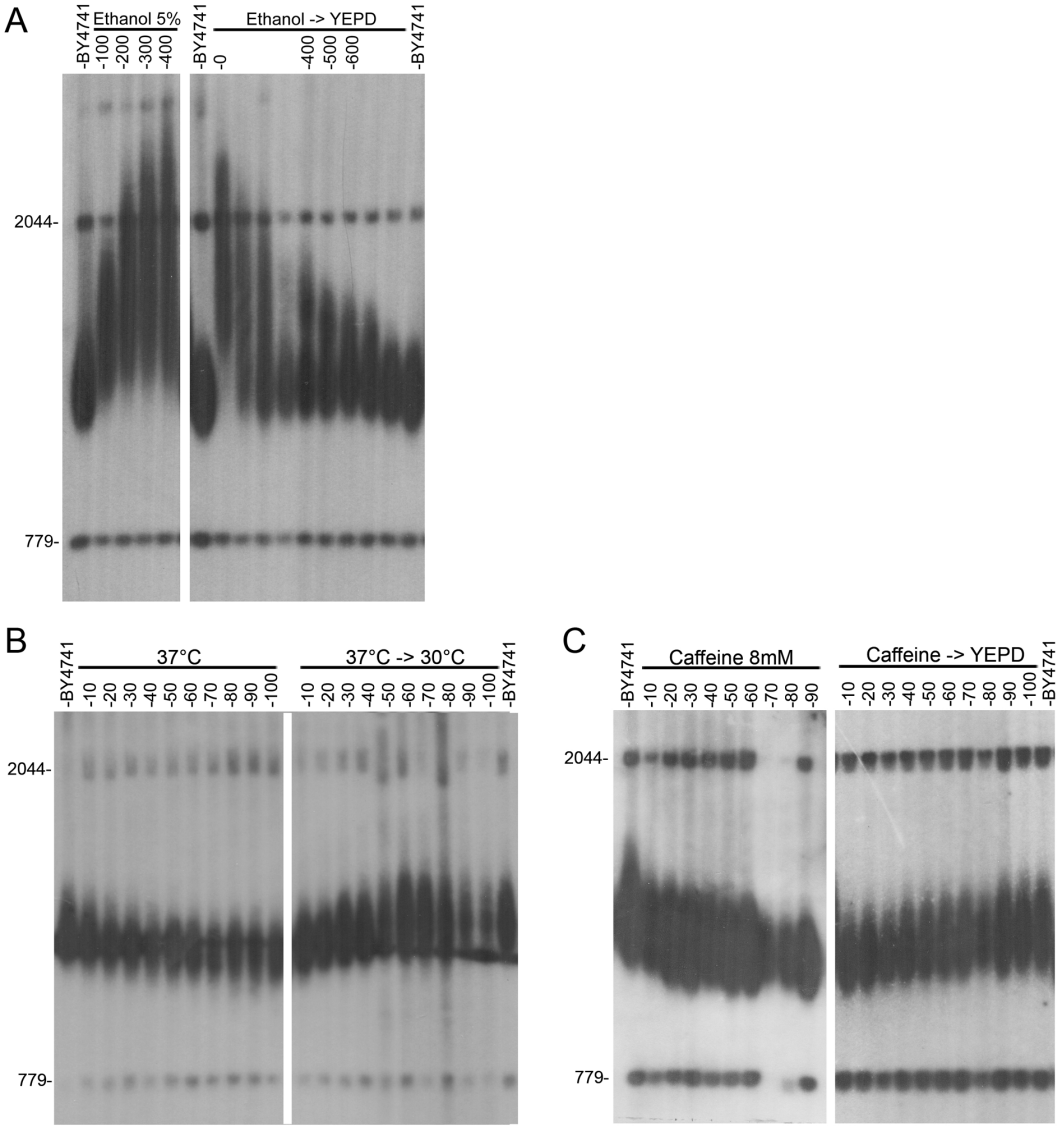
Kinetics of telomere length change after exposure to environmental stresses. Wild-type yeast strain BY4741 was grown in the presence of various stresses and then released. **A**. YEPD containing 5% ethanol (released to YEPD after 300 generations). **B**. YEPD+8 mM caffeine (released to YEPD after 90 generations). **C**. YEPD at 37°C (released back to 30°C after 100 generations).

We exposed yeast cells (*S. cerevisiae*) to thirteen different environmental signals for 100–400 generations (Figure 1 and Table S1). Our results show that some stresses, such as high temperature, the addition of caffeine, and low levels of hydroxyurea resulted in telomere shortening, while others, such as added acetic acid and alcohols including ethanol, methanol, and isopropanol, caused a significant increase in telomere length (Figure 1). Strikingly, under alcohol stress telomeres were not only longer, but also exhibited length heterogeneity, indicating that the mechanism responsible for telomere length homeostasis, which preferentially elongates short, but not long telomeres [16], was disrupted (Figures 1, 2). The effect of alcohols on telomere length was independent of the ability of these cells to metabolize the alcohol: Upon ethanol treatment, isogenic petite yeast strains (lacking mitochondrial function, and thus unable to utilize ethanol) exhibited elongated telomeres (Figure S1).

Importantly, however, many other environmental stresses, including oxidative stress, did not significantly alter telomere length (Figure 1 and Table S1), indicating that telomere length homeostasis is robust under many other environmental conditions. The effect of each stress on telomere length was concentration-dependent. In all cases, removal of the stressing agent resulted in a gradual restoration of wild type telomere length (Figure 2A–C), demonstrating that the changes in telomere length were physiological rather than genetic, and thus may have been mediated by altered gene expression and protein activity.

### Telomere length alteration under stress is not recombination-dependent

Under unperturbed conditions, telomere length can be modified either by disrupting the regulation of telomerase/telomere-associated nucleases or by recombination. To distinguish between these two mechanisms, we analyzed the response to stresses of cells unable to carry out homologous recombination due to a deletion of the *RAD52* gene. *rad52* cells responded to the stresses much as would a wild type strain, indicating that telomere length alteration in response to these stresses is not recombination-dependent (Figure S2) and that the external signals affect telomerase or telomere-associated nucleases.

### Exploring the mechanisms in which stress affect telomere length

To understand how external signals affect telomere length and to identify the mechanism behind this telomeric response to stress, we measured genome-wide transcript levels in yeast cells grown for 20 generations in the presence of stresses that showed an effect on telomere length (ethanol, caffeine or high temperature), as well as in the presence of H_2_O_2_, a stress that does not alter telomere length. The results were compared to genome-wide transcript levels of the same strain grown under standard conditions (YEPD medium, 30°C). Using Significance Analysis of Microarrays (SAM) [17] with a false discovery rate (FDR) below 0.01, we obtained a set of 1,744, 1,404, 1,670 and 1,019 differentially expressed genes for caffeine, 37°C, ethanol and H_2_O_2_, respectively. General environmental stress responding (ESR) genes were not induced under these conditions, as expression level was measured after a long-term exposure to the stresses while ESR genes are induced for a short time period [18]. To identify the mechanisms responsible for telomere elongation and shortening, we sought genes that were differentially expressed only under shortening or only under elongating conditions (Figure S3). We integrated transcript abundance data with the known TLM network [13] that uses protein-protein interactions data, connecting TLM genes to the telomere maintenance machinery. The (unweighted) pairwise distances between stress-specific differentially expressed TLM genes were compared with pairwise distances of other TLM genes. This revealed that stress-specific, differentially expressed TLM genes lie significantly closer to each other for ethanol, caffeine and 37°C (p<2E-33,p<3E-27 and p<3E-50, respectively), but not for hydrogen peroxide stress, which does not affect telomere length (Materials and Methods). This phenomenon was unique to TLM genes under stresses that affect telomere length, suggesting that the differentially expressed TLM genes may be involved in transducing the external signals and disrupting telomere length homeostasis.

**Figure 3:**
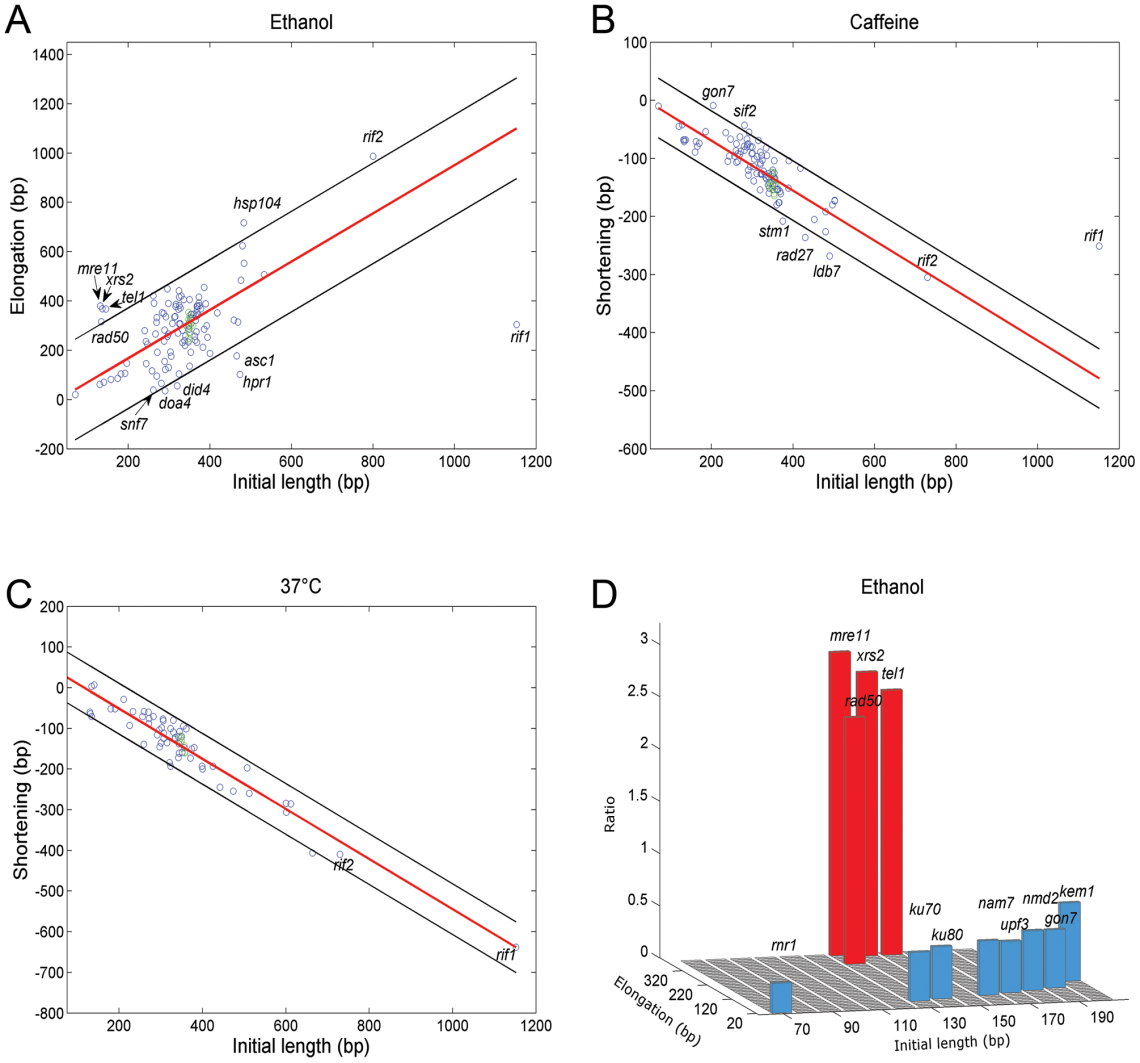
Different stresses affect telomere length via different genes. The effect of ethanol, caffeine and high temperature on telomere length was tested on strains carrying individual gene deletions/hypomorphic mutations. Each mutant was grown under the relevant stress for 100 generations and its telomere length was measured using Southern blot analysis. **A–C**. The X-axis shows the initial length of each mutant and the Y-axis shows the elongation or shortening after 100 generations. Each strain analyzed is represented by a circle (wt in green). A strong correlation (demarked by a red line; ±5% SD) was seen between the initial length and the effect of the stress. **A**. Ethanol. **B**. Caffeine. **C**. 37°C. **D**. Each bar represents the ratio between the initial telomere length and the elongation after 100 generations in ethanol. Very short *tlm* mutants (below 200 nt long) could be clearly separated into two groups: mutants of the Tel1 pathway (*tel1Δ*, *mre11Δ*, *rad50Δ*, *xrs2Δ*) were highly responsive to ethanol stress, while mutants of the NMD (*nam7Δ*, *upf3Δ*, *nmd2Δ*) and Ku (*yku70Δ*, *yku80Δ*) pathways show little telomeric elongation under ethanol stress.

Based on the analysis above, we generated a list of candidate genes for further analysis. Using strains from the yeast deletion library [19] and the DAmP library of hypomorphic mutants [20] we screened mutants in this list to identify genes important for telomere length maintenance under stress conditions. Strikingly, we found a strong correlation between the rate of change in telomere length and the initial length of the mutant: in ethanol, long *tlm* mutants elongate more rapidly than the wild type, while short *tlm* mutants elongate more slowly (Pearson correlation, r = 0.61, p<E-12, Figure 3A). Similarly, in caffeine and at 37°C long *tlm* mutants shorten more rapidly, while short *tlm* mutants shorten more slowly than does the wild type (Pearson correlation, r = −0.78, p<2E-22 and r = −0.96, p<9E-34, respectively; Figure 3B–C). This correlation between abnormal telomere length and response magnitude to the stresses suggests that telomere elongation/shortening in the presence of external cues is carried out by the same basic mechanisms that maintain telomere length under unperturbed conditions.

### Telomere length alteration under stress is Rif1, but not Rif2 dependent

**Figure 4:**
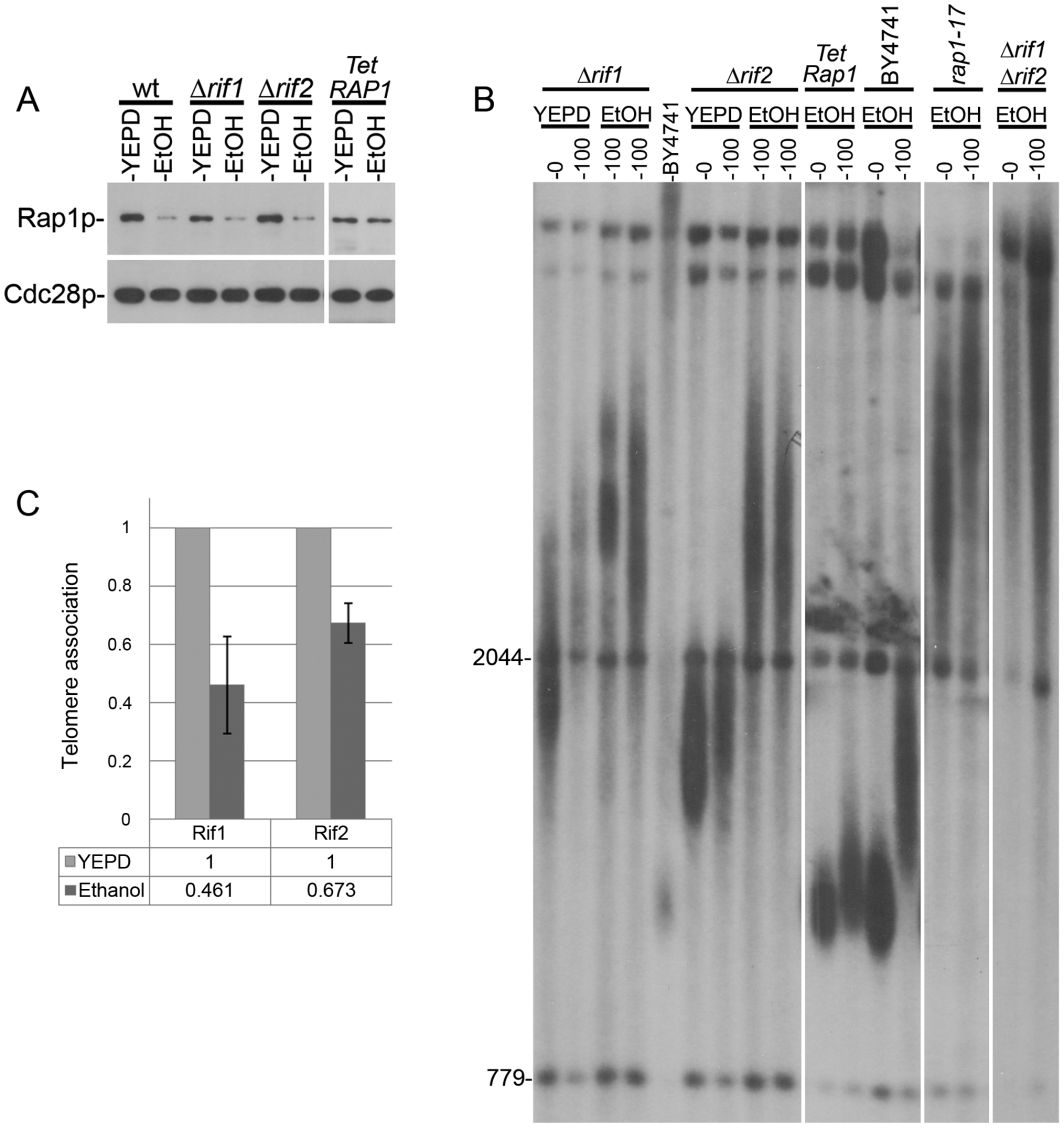
Telomere elongation of different mutants grown in the presence of ethanol. **A**. The level of Rap1 protein is reduced upon exposure to ethanol in wt, *rif1Δ* and *rif2Δ* mutants, but not in a strain in which the *RAP1* gene is under the Tet promoter. **B**. The initial telomere length and the elongation after 100 generations in ethanol were measured by Southern blot. A deletion of *RIF1* (two independent cultures) inhibits the response to ethanol while deletion of *RIF2* (two independent cultures) increases it. A *rap1-17* strain, unable to bind Rif1 or Rif2, behaves similarly to the double mutant *rif1Δ rif2Δ*. Expressing Rap1 under the tetracycline promoter (which is not affected by ethanol) prevents the telomeric elongation under ethanol stress. **C**. Chromatin Immunoprecipitation (ChIP) analysis for the recruitment levels of Rif1 and Rif2 proteins to telomeres, in the absence and in the presence of ethanol. The non-telomeric *ARO1* locus was used to normalize the relative levels.

To identify the genes that mediate the telomeric response to stress and to understand how external signals are transduced to altering telomere length, we focused on mutants that disrupt this transduction and, therefore, show an atypical response to each stress (Figure 3). A remarkable such *tlm* mutant is *rif1Δ*, which exhibited a reduced response to ethanol and caffeine but normal response to 37°C (Figures 3A and 4), indicating that elongation by ethanol and shortening by caffeine are Rif1-dependent, while telomere shortening by high temperature relies on a different mechanism.

The Rif1 and Rif2 proteins are negative regulators of telomerase that interact with the C-terminus of Rap1, an essential protein that binds to the telomeric repeats [21]. Under normal growth conditions, short telomeres are preferentially elongated by a mechanism that depends on Rap1. Mutations in the carboxy-terminus of *RAP1* or down-regulation of the *RAP1* gene lead to extreme telomere elongation and to an increase in telomere length variability, similar to what we observed in the presence of ethanol ([22], [23]; Figure 2). Our transcript measurements detected a reduction in the level of Rap1 expression in cells grown in the presence of ethanol [(Table S2); and [24]]. These results suggest a model in which telomere elongation under ethanol stress is primarily due to reduced levels of Rap1, which reduce Rif1 recruitment to telomeres. To test this hypothesis, we used a strain in which *RAP1* was expressed from a Tetracycline-inducible promoter [25]. In this strain the level of Rap1 remained unchanged in the presence of ethanol (Figure 4A) and only a slight telomere elongation was observed (Figure 4B). Also consistent with the model, a *rap1-17* strain (deleted for the C terminus of Rap1), a *rif1Δ* single mutant and a *rif1Δ rif2Δ* double mutant exhibited attenuated responses to ethanol (Figure 4B). Thus, the telomere elongation response to ethanol was abolished when a steady level of Rap1 protein was maintained or when Rif1 activity was eliminated, indicating that the Rap1- Rif1 pathway is central to telomere elongation in response to ethanol. Consistent with this hypothesis, chromatin immunoprecipitation (ChIP) experiments showed that upon exposure to ethanol there is a two-fold reduction in the level of Rif1 at telomeres, as well as a slighter reduction in the level of Rif2 (Figure 4C). Since it is necessary for both elongation and shortening responses, Rif1 may play a general sensing/structural/regulatory role, rather than a catalytic one, in the telomeric response to environmental signals. This is consistent with recent studies that found a role for Rif1 in the regulation of chromatin structure and of DNA replication origin firing [26], [27].

Remarkably, *rif2Δ* cells exhibited a strong response to ethanol (Figure 3A), underscoring the different roles of Rif1 and Rif2 in telomere length maintenance [28]–[32]. We suggest that exposure to ethanol reduces the recruitment of the Rif proteins at the telomere ends, resulting in conditions permissive for indiscriminate telomerase recruitment, elongating both short and long telomeres, and yielding a broad distribution of telomere lengths (Figure 2A). The insensitivity of *rif1Δ* mutants to ethanol could be due to the importance of Rif1p for the telomere elongation response, and/or the increased binding of Rif2 to telomeres in the absence of Rif1. In agreement with this model, deletion of *RIF2* caused over-extension of telomeres in ethanol (Figure 3A); a reduction of Rif1 telomere recruitment by ethanol in the strain deleted for *RIF2* mimics a *rif1Δ rif2Δ* double mutant, which exhibits increased levels of telomere elongation. In contrast to these results, the *RIF2* deletion had no effect on the reduction in telomere length upon exposure to caffeine or 37°C (Figure 3B,C).

### The Tel1 and NMD pathways have separate roles in telomere elongation under ethanol stress

Mutations in the *TEL1* gene, which encodes the yeast ortholog of the mammalian ATM protein kinase, result in very short telomeres. Tel1 regulates the preferential elongation of short telomeres [33] by a pathway that also includes the MRX complex (Mre11, Rad50, Xrs2; [34]). A separate regulatory branch includes the yeast Ku proteins [35]. Figure 3D shows that the *tlm* mutants with very short telomeres could be clearly separated into two groups: telomeres of mutants of the Tel1 pathway (*tel1Δ*, *mre11Δ*, *rad50Δ*, *xrs2Δ*) were hyper-responsive, while mutants of the NMD (nonsense mediated decay, *nmd2Δ*, *nam7Δ* and *upf3Δ*) and Ku pathways had only a mild response to ethanol. The fact that telomeres can be elongated by ethanol in the absence of Tel1 or of components of the MRX complex is surprising; notably, the wide size distribution observed upon exposure to ethanol (Figures 1, 2), is consistent with a mechanism independent of the one that preferentially elongates the shortest telomeres, which depends on the Tel1 pathway [16].

The NMD pathway degrades mRNAs carrying nonsense mutations. In addition, it affects the steady state level of hundreds of mRNAs, including those known to act at telomeres (e.g., Est1, Est2, and two components of the CST telomeric capping complex, Stn1 and Ten1 [36]). Mutations in the NMD machinery lead to higher mRNA levels of these proteins and to short telomeres [37]. The NMD pathway has been recently shown to affect the fitness of *cdc13-1* and *yku70* mutants by controlling the expression of Stn1, an essential telomere capping protein, which interacts with Cdc13 and participates in the recruitment of telomerase [38]. In *nmd* mutants, the response of telomeres to ethanol stress is reduced relative to wild-type strains, indicating that the NMD pathway is involved in telomere elongation during ethanol stress. We asked if upregulation of Ten1 and Stn1 is involved in this effect by overexpressing these genes in naïve cells and measuring the effect of ethanol on telomere length in these cells (Figure S4). Overexpression of Stn1 reduced the ethanol response and overexpression of both Stn1 and Ten1 completely abolished the telomere length response to ethanol. These results suggest that the level of CST activity, controlled by the NMD pathway, plays an important role in the telomere elongation response to ethanol. This is consistent with the proposed role of the CST complex in telomerase activation. Interestingly, mutations in the CST proteins are lethal when combined with a deletion of *RIF1* [28]–[32], indicating the existence of an essential overlapping function between the two telomere regulatory components. The roles of the CST and Rif1 in transducing the ethanol signal to the telomeres will be the subject of future research.

### Additional mutants affecting telomere response to ethanol

Among the additional mutants with a reduced response to ethanol were *doa4Δ*, *snf7Δ* and *did4Δ* (Figure 3A). *DOA4* encodes an enzyme that removes ubiquitin from membrane proteins destined for vacuolar degradation. The Doa4 protein resides in the late endosome, where it interacts with the ESCRT-III machinery, which includes Did4 and Snf7 [39]. A role was previously observed for vacuolar traffic proteins in telomere length maintenance [40]; however, the precise mechanism remains enigmatic. Another mutant that shows apathy towards ethanol is *hpr1Δ*, defective for a component of the THO complex. Consistent with these results, mutations in *HPR1* were recently shown to affect the expression levels of *RIF1* [41].

In contrast to these genes, a deletion of *HSP104* was hyper-responsive to ethanol. Hsp104 is a stress chaperone that plays an important role in maintaining prion particles in the cell [42]. It is unclear whether its role in telomere length regulation is related to its role in prion maintenance.

### Telomere response to caffeine and high temperature

Deletion of Rif1 and mutations in Rap1 also significantly decrease the telomeric response to caffeine, indicating that Rif1-Rap1 is not only involved in telomere elongation under ethanol stress, but also in telomere shortening under caffeine. Caffeine is a known inhibitor of phosphatydyl inositol-3 kinase related kinases (PI3K-like kinases) such as human ATR and ATM [43] and their yeast counterparts, Tel1 and Mec1 [44]. Therefore, we tested whether mutations in these target genes would abolish the telomere shortening caused by caffeine. Indeed, deletion of either *TEL1* or *MEC1* individually does not prevent the response to caffeine, but a double mutant *tel1Δ mec1Δ* is completely insensitive to the telomeric effect of caffeine (Figure S5), consistent with the known redundant function that these two kinases play in telomere biology [45]. Thus, caffeine causes telomere shortening by inhibiting the ATM/ATR-like regulatory kinases.

Mutations in Rap1 and the deletion of Rif1 affect only the shortening rate in the presence of caffeine but do not affect the response to high temperature. High temperature has a broad, pleiotropic effect, and may alter telomere length via several mechanisms. Several TLM genes that, when mutated, result in short telomeres, are down regulated by high temperature (Table S3). However, no single deletion mutant failed to respond to high temperature by shortening its telomere length, suggesting that there are redundant functions among these responding genes. This result is consistent with a recent study [46] proposing that one or more telomerase components are intrinsically thermolabile.

Accurate telomere length homeostasis is dependent on a large genetic network that includes ∼400 (largely evolutionarily conserved) genes [9]–[11]. Our results show that this network can be disrupted by several environmental signals, and by different regulation mechanisms that lead to altered telomere length. These responses are distinct from the stereotypic responses to stress [18], and seem to be specific only to particular conditions.

Telomere length and telomerase activity are important factors in the pathobiology of human disease. Age-related diseases and premature aging syndromes, for example, are characterized by the shortening of telomeres [47]. Tumor cells, on the other hand, prevent telomere shortening and telomere loss by up-regulating telomerase, thereby perpetuating cells with short telomeres and high chromosomal instability [48]. Thus, although the mechanisms at work differ, changes in telomere length fuel disease pathology in cancer and other premature aging syndromes. While previous studies have identified correlations between telomere length and environmental conditions such as mental stress [49], socioeconomic status [50], and health-related behavior in adults [51], we extend those findings here by demonstrating direct causality between environmental cues and changes in telomere length. This identification of mechanisms by which external signals modify telomere length significantly advances our understanding of the complex interplay of genes and environment. More critically, however, these findings also point a future path to strategic manipulations of telomere length that may well have important therapeutic implications in the treatment of human disease.

## Materials and Methods

### Yeast strains

All the yeast strains used in this study are derivatives of BY4741 (*MAT*a *ura3Δ met15Δ leu2Δ his3Δ*), unless otherwise specified. Mutants were obtained from the yeast deletion library [19] or from the DAmP library of hypomorphic alleles [20]. Strains carrying genes with tetracycline-inducible promoters were taken from the library described in [25]. Petite BY4741 derivatives were obtained by plating cells on YEPD plates containing ethidium bromide. Strains deleted for *MEC1*, *TEL1* and *SML1* were in the MS71 background [52] (kindly provided by T. Petes).

### Telomere length measurement

Telomeric Southern blots were carried out as in [53]. PCR fragments containing telomeric sequences and a genomic region that hybridizes to two size marker bands (2044 and 779 bp) were used as probes. The telomere length was measured with an in-house software (TelQuant) using the size marker bands as reference. Telomere length was ∼1250 bp in wt cells [composed of the sub-telomeric region (∼900 bp) and the telomere repeats (∼350 bp)].

### Exposing cells to mild environmental stresses

Stress levels were calibrated to reduce growth by 40%–60%. Cells were subjected to the various stresses by serial transfer growth: a single colony of BY4741 was grown in rich medium (YEPD), and 5 µl were used to inoculate 5 ml cultures under the various stress conditions (in triplicates). The cultures were grown ∼10 generations before being diluted (1∶1000) into fresh medium.

### Extracting differentially expressed genes in each stress

We analyzed stress-induced RNA response for caffeine, temperature of 37°C, ethanol and hydrogen peroxide (H_2_O_2_), using Affymetrix GeneChip Yeast Genome 2.0 arrays. Transcript levels were measured for three independent cultures grown in the presence of the stress agent, and were compared to a control set comprised of four wild-type measurements. To obtain differentially expressed genes between the stress-induced response and the control measurements, we (i) employed the Robust Multi-array Average (RMA) method for normalization and summarization of the Affymetrix arrays [54]; (ii) filtered probes which had more than half of their detection calls marked as absent; and (iii) employed the Significance Analysis of Microarrays (SAM) [17] with false discovery rate (FDR) below 0.01. Following these procedures, we obtained a set of 1,744, 1,404, 1,670 and 1,019 differentially expressed genes for caffeine, 37°C, ethanol and H_2_O_2_, respectively.

### Integrating transcript abundance data with PPI network

We used the un-weighted TLM-based network described in [13], representing the most likely network connecting TLM genes to the telomere maintenance machinery. We next compared the pairwise shortest (unweighted) distances in the network between stress-specific differentially expressed TLM genes and other TLM genes, revealing that stress-specific differentially expressed TLMs for ethanol, caffeine and 37°C lie significantly closer to each other than other TLM genes (Wilcoxon ranked sum test, p<2e^−33^,p<3e^−27^ and p<3e^−50^ for ethanol, caffeine and 37°C stresses, respectively). Reassuringly, the hydrogen peroxide stress showed no significant difference between the two types of TLM genes. Last, using an assembled yeast protein-protein interaction network [13], we verified that stress-specific differentially expressed TLM genes are significantly closer in this network than other stress-specific differentially expressed genes (p<6e^−9^ for all stresses), verifying that closeness on the network is not a general property of differentially expressed genes.

### Detection of over- and under-responsive genes in the presence of stress

In an attempt to identify stress-response related genes, we examined the elongation or shortening of the telomere for each knockout gene in the absence or presence of the stress. The elongation/shortening of the telomere in the presence of the stress displayed a linear relation with the initial length of the telomere (Pearson correlation coefficient between the two variables is ρ = −0.77 (p<9e^−25^), −0.95 (p<2e^−38^) and 0.36 (p<7e^−6^) for caffeine, 37°C and ethanol, respectively). In order to detect outliers, we performed a robust linear regression analysis. Following [55], we assumed that the residuals follow a normal distribution and identified the outlier genes as the most extreme 5% (2.5% from each side). The computations were performed using Matlab.

### Chromatin immuno-precipitation (ChIP)

Chromatin immuno-precipitation (ChIP) was carried out by standard methods [56]. The association of Rif1-HA, and Rif2-HA with Y′-element telomeres was detected using Santa Cruz Mouse anti HA monoclonal IgG antibodies (SC-7392). Real-time PCR (RT-PCR) reactions were carried out using the following primers: Y′-element : 5′-GGCTTGATTTGGCAAACGTT-3′, and 5′-GTGAACCGCTACCATCAGCAT-3′. ARO1: 5′-GTCGTTACAAGGTGATGCC-3′, and 5′- CGAAATAGCGGCAACAAC-3′. The relative fold enrichment\depletion of the telomere-associated proteins Rif1 and Rif2 was calculated as follows: [telIP/ARO1IP]/[tel input/ARO1input] [57].

## Supporting Information

Figure S1 Ethanol causes telomere length increase in strains unable to metabolize it. Strain BY4741 and two independent petite derivatives were grown for 60 generations in the presence of 5% ethanol.
(PDF)

Figure S2 The changes in telomere length caused by environmental stress are independent of homologous recombination. A *rad52Δ* strain shows telomere elongation in the presence of ethanol and telomere shortening in the presence of caffeine and high temperature.
(PDF)

Figure S3 Venn diagram showing the number of differentially expressed genes under each of the stress conditions tested.
(PDF)

Figure S4 The NMD pathway affects the response to ethanol through the Ten1 and Stn1 genes (components of the CST complex). Wild type cells carrying various plasmids were grown in the presence of 5% ethanol for the number of generations shown. Overexpression of either Stn1 or Ten1 has no effect or only a mild effect on telomeric elongation, while overexpression of both together inhibits the telomeric elongation under ethanol stress.
(PDF)

Figure S5 **Mec1 and Tel1 mediate caffeine stress.** Wild type cells, as well as two independent colonies of strains deleted for either *MEC1*, *TEL1* or both (all in a *sml1Δ* background) were grown in the presence of caffeine for 100 generations. The double mutant *tel1Δ mec1Δ* did not exhibit telomere shortening by caffeine.
(PDF)

Table S1 The effect of environmental signals on telomere length.
(DOCX)

Table S2 Expression levels as measured by DNA microarray hybridization. Cells were grown in the presence of either ethanol, caffeine, H2O2 at 30°C, in YEPD at 30°C and at 37°C.
(PDF)

Table S3 List of genes whose expression changed upon growth on stressing conditions. TLM genes are marked “1”, non-TLM genes “0”. Increased expression upon stress is denoted “1”, reduced expression appears as “−1”.
(PDF)
