# Supplementary figures and images for: Environmental Stresses Disrupt Telomere Length Homeostasis
Source: PLoS Genet. 2013 Sep 5;9(9):e1003721. doi: 10.1371/journal.pgen.1003721 (PMC3764183; doi:10.1371/journal.pgen.1003721)

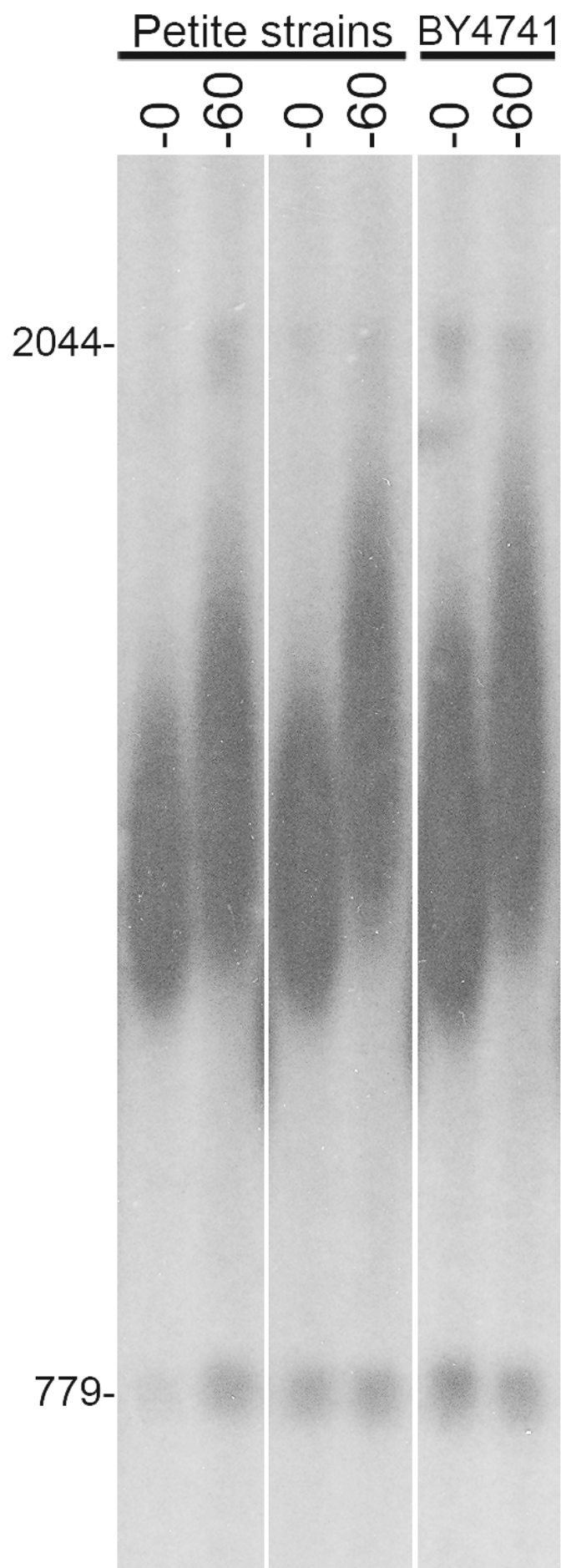

Romano *et al.* Figure S1.

Supplement: Figure S1 — Ethanol causes telomere length increase in strains unable to metabolize it. Strain BY4741 and two independent petite derivatives were grown for 60 generations in the presence of 5% ethanol. (PDF) [file pgen.1003721.s001.pdf]

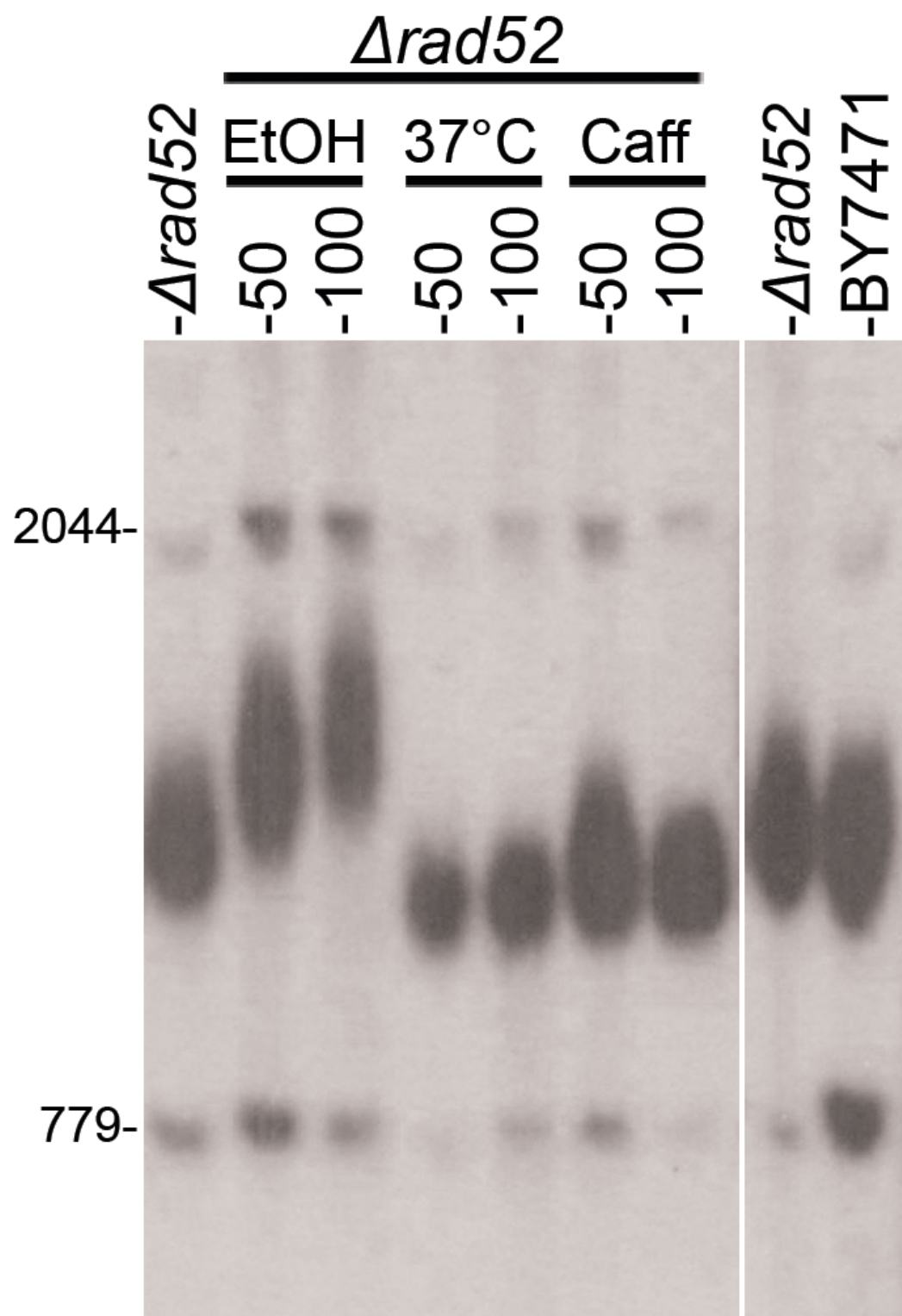

Supplement: Figure S2 — The changes in telomere length caused by environmental stress are independent of homologous recombination. A rad52Δ strain shows telomere elongation in the presence of ethanol and telomere shortening in the presence of caffeine and high temperature. (PDF) [file pgen.1003721.s002.pdf]

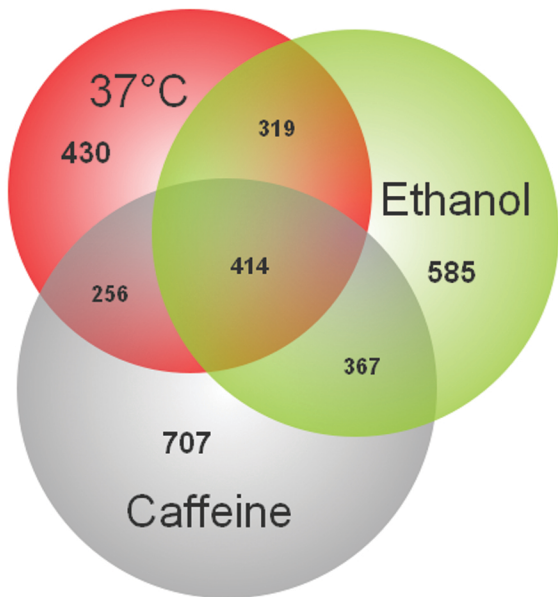

Romano *et al.* Supp. Fig. 3

Supplement: Figure S3 — Venn diagram showing the number of differentially expressed genes under each of the stress conditions tested. (PDF) [file pgen.1003721.s003.pdf]

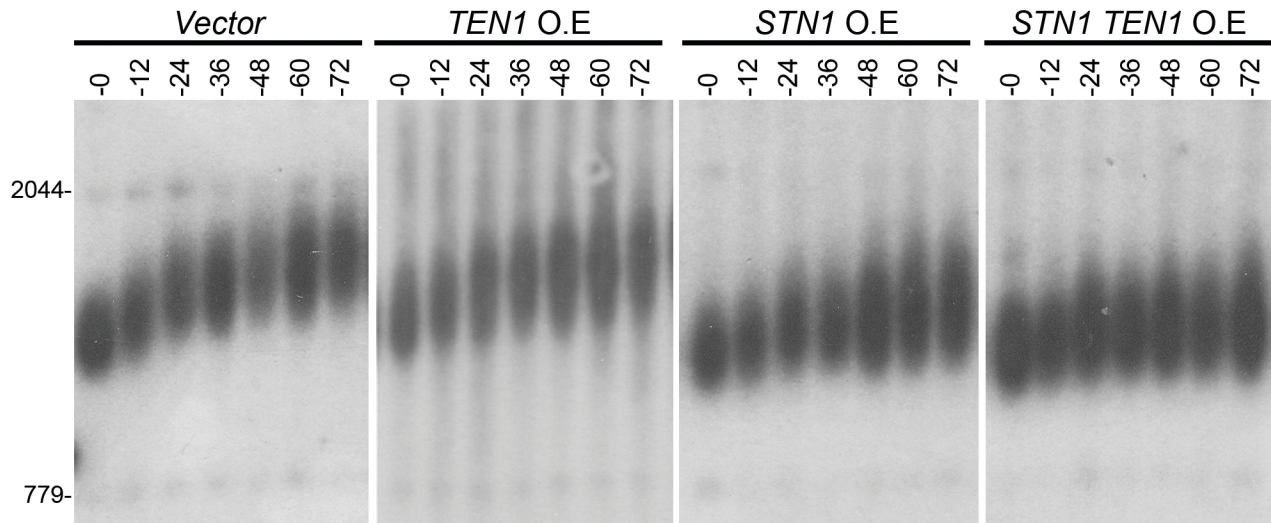

Supplement: Figure S4 — The NMD pathway affects the response to ethanol through the Ten1 and Stn1 genes (components of the CST complex). Wild type cells carrying various plasmids were grown in the presence of 5% ethanol for the number of generations shown. Overexpression of either Stn1 or Ten1 has no effect or only a mild effect on telomeric elongation, while overexpression of both together inhibits the telomeric elongation under ethanol stress. (PDF) [file pgen.1003721.s004.pdf]

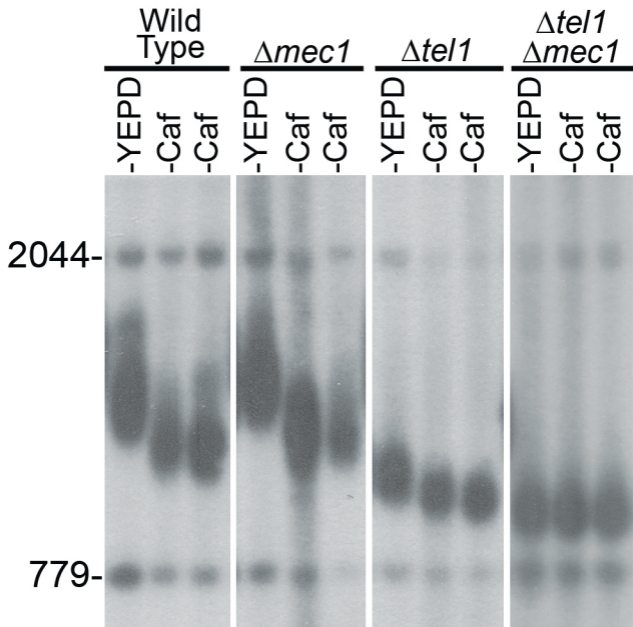

Supplement: Figure S5 — Mec1 and Tel1 mediate caffeine stress. Wild type cells, as well as two independent colonies of strains deleted for either MEC1, TEL1 or both (all in a sml1Δ background) were grown in the presence of caffeine for 100 generations. The double mutant tel1Δ mec1Δ did not exhibit telomere shortening by caffeine. (PDF) [file pgen.1003721.s005.pdf]
