# Supplementary material for: Environmental Stresses Disrupt Telomere Length Homeostasis
Source: PLoS Genet. 2013 Sep 5;9(9):e1003721. doi: 10.1371/journal.pgen.1003721 (PMC3764183; doi:10.1371/journal.pgen.1003721)
Supplement: Table S1 — The effect of environmental signals on telomere length. (DOCX) [file pgen.1003721.s006.docx]

Supplementary Table 1 – The effect of environmental signals on telomere length.

|  | **After 50 generations** | | | **After 100 generations** | | |
| --- | --- | --- | --- | --- | --- | --- |
| **Condition** | **Average length** | **Standard deviation** | **P value** | **Average length** | **Standard deviation** | **P value** |
| **No Stress** | 348 | 11.10 | 1 | 349 | 9.906708 | 1 |
| **Ethanol 3%** | 471 | 7.81 | 2.15E-06 | 522 | 14.3846 | 7.69E-06 |
| **Ethanol 5%** | 572 | 10.97 | 1.01E-05 | 674 | 14.37591 | 4.09E-07 |
| **Ethanol 7%** | 603 | 11.93 | 1.6E-05 | 728 | 12.68529 | 3.55E-08 |
| **Methanol 3%** | 370 | 6.51 | 0.007185 | 400 | 5.686241 | 2.44E-05 |
| **Methanol 5%** | 389 | 13.80 | 0.017227 | 438 | 8 | 3.28E-05 |
| **Methanol 7%** | 403 | 9.50 | 0.000837 | 458 | 9.865766 | 0.000115 |
| **Isopropanol 3%** | 408 | 2.00 | 3.14E-06 | 456 | 6.601767 | 9.32E-09 |
| **Isopropanol 4%** | 445 | 6.00 | 3.83E-07 | 486 | 9.165151 | 2.19E-05 |
| **Isopropanol 5%** | 475 | 6.51 | 1.6E-07 | 589 | 4.163332 | 1.85E-11 |
| **Acetic Acid 40mM (pH4.4)** | 393 | 5.03 | 2.5E-05 | 496 | 1.414214 | 4.73E-09 |
| **Acetic Acid 60mM (pH4.3)** | 402 | 2.89 | 4.05E-06 | 505 | 7.071068 | 0.000725 |
| **Acetic Acid 80mM (pH4.2)** | 412 | 2.89 | 1.18E-06 | 516 | 6.363961 | 0.000227 |
| **H_2_O_2_ 1 mM** | 336 | 8.14 | 0.101082 | 331 | 8.995369 | 0.016517 |
| **H_2_O_2_ 2 mM** | 331 | 8.39 | 0.040034 | 343 | 2.872281 | 0.160374 |
| **H_2_O_2_ 3 mM** | 333 | 4.51 | 0.017155 | 340 | 6.70199 | 0.246142 |
| **KCL 0.5M** | 343 | 6.66 | 0.427858 | 343 | 12.12436 | 0.491881 |
| **KCL 1M** | 342 | 15.89 | 0.606464 | 335 | 13.97617 | 0.137293 |
| **High pH (7.9)** | 347 | 7.64 | 0.816832 | 349 | 3.41565 | 0.879714 |
| **High osmolarity (Sorbitol 1M)** | 361 | 15.31 | 0.27166 | 357 | 14.3846 | 0.366301 |
| **CuSO_4_ 6 mM** | 349 | 2.00 | 0.849541 | 371 | 8.180261 | 0.005176 |
| **CuSO_4_ 8 mM** | 378 | 18.52 | 0.091591 | 384 | 12.71482 | 0.005192 |
| **CuSO_4_ 15 mM** | 366 | 6.03 | 0.01457 | 364 | 18.40969 | 0.215182 |
| **Anaerobic** | 327 | 16.62 | 0.147538 | 323 | 11.29528 | 0.009742 |
| **Hydroxyurea 2mM** | 332 | 9.07 | 0.060319 | 335 | 15.34872 | 0.159582 |
| **Hydroxyurea 20mM** | 318 | 16.56 | 0.070093 | 298 | 7.505553 | 1.04E-05 |
| **Hydroxyurea 40mM** | 297 | 4.00 | 5.24E-06 | 292 | 9.215024 | 3.25E-05 |
| **Caffeine 4mM** | 291 | 4.73 | 3.29E-06 | 271 | 8.346656 | 1.37E-06 |
| **Caffeine 8mM** | 257 | 3.06 | 7.72E-08 | 230 | 3.872983 | 1.15E-09 |
| **Caffeine 12mM** | 232 | 2.89 | 1.38E-08 | 201 | 3.316625 | 3.87E-10 |
| **High Temperature (37^o^C)** | 238 | 6.66 | 5.18E-07 | 206 | 7.675719 | 5.39E-09 |
