# Supplementary material for: Environmental Stresses Disrupt Telomere Length Homeostasis
Source: PLoS Genet. 2013 Sep 5;9(9):e1003721. doi: 10.1371/journal.pgen.1003721 (PMC3764183; doi:10.1371/journal.pgen.1003721)
Supplement: Table S2 — Expression levels as measured by DNA microarray hybridization. Cells were grown in the presence of either ethanol, caffeine, H2O2 at 30°C, in YEPD at 30°C and at 37°C. (PDF) [file pgen.1003721.s007.pdf]

ematic	Name	Score	Chan	ematic	Name	Score	Chan	ematic	Name	Score	Chan	ematic	Name	Score	Change
THANOL				CAFFEINE				37C				H2O2			
YDR38	ARO1(	35.3	11.1	YJR15	DAN4	23.9	23.5	YFL01	HSP1	37.1	23.8	YEL03	CYC	25.6	5.6
YIL014	-	29.7	4.6	YPR00	PDH1	20.0	5.8	YGR03	GSC2	27.6	5.4	YHR20	---	23.2	3.8
YNR03	-	29.7	8.1	YJR15	DAN1	19.9	56.4	YNR03	-	24.6	4.6	YNR03	---	22.3	13.7
YFL01	HSP12	29.0	8.7	YEL04	PAU2	18.9	9.3	YGR04	NQM	20.3	8.0	YDR53	FIT1	20.7	7.7
YER15	SPI1	26.3	5.2	YBR30	DAN3	17.2	13.1	YGL12	GPG	20.0	11.1	YKL16	PIR3	20.5	25.8
YMR09	-	23.8	5.0	YJL13	-	16.2	7.5	YEL03	CYC7	19.9	4.5	YFL01	HSP	20.2	14.9
YBL02	-	22.9	4.2	YHR20	CRG1	16.1	3.8	YGR24	SOL4	19.4	13.3	YLR14	PUT	20.1	4.3
YHR05	RSC30	22.6	3.0	YKR09	SRL3	16.1	4.8	YBR08	-	18.8	3.6	YGR03	GSC	18.6	3.4
YHR05	RSC30	22.6	3.0	YOR00	TIR4	16.0	6.7	YER15	SPI1	18.0	7.0	YFR01	---	18.6	3.2
YGR03	GSC2	21.2	4.1	YBR05	-	14.6	3.9	YNL16	YGP1	16.6	4.1	YIL099	SGA	18.4	2.8
YEL01	GLC3	20.2	3.7	YMR31	-	14.1	14.2	YJR07	BNA2	16.0	8.5	YLR32	TMA	17.5	12.7
YJL079	PRY1	19.6	4.6	YIL011	TIR3	14.0	4.3	YBR05	YRO2	15.3	5.3	YOR38	FIT3	17.5	5.2
YBR28	-	18.7	4.6	YIR039	YPS6	14.0	2.5	YAL00	SSA1	14.9	1.9	YMR09	SNZ	17.4	3.3
YHR13	ARO9	18.6	4.8	YFL02	PAU5	13.7	6.3	YPL24	HSP8	14.8	2.5	YOL05	DDR	16.9	17.0
YDL07	MRK1	18.1	2.4	YDR53	FIT1	13.5	8.6	YDR38	ARO1	14.8	3.9	YBR08	---	16.8	2.9
YKL10	HAP4	18.1	4.0	YGR03	GSC2	13.4	3.3	YDR03	-	14.6	5.6	YDR03	---	16.6	9.3
YDR53	APA2	18.1	2.1	YEL07	DLD3	13.4	2.7	YHR05	RSC3	14.3	2.7	YFL05	---	16.5	1.6
YJL048	UBX6	18.1	2.8	YMR19	CMC4	13.0	2.5	YHR05	RSC3	14.3	2.7	YEL01	GLC	16.5	2.6
YGL12	GPG1	17.8	7.9	YLR05	OSW	12.4	3.3	YOR37	ALD4	13.9	2.9	YGL12	GPG	16.4	6.7
YGR24	SOL4	17.5	4.0	YOR38	FIT2	12.4	5.9	YLR03	-	13.3	3.3	YIR039	YPS	15.8	2.5
YNL16	YGP1	16.5	3.9	YGL12	GPG1	12.3	5.4	YMR19	-	13.2	4.1	YBR05	---	15.6	3.2
YCR02	HSP30	16.4	8.8	YOR38	FIT3	11.9	4.9	YEL05	-	12.9	2.2	YEL06	PRB	15.4	2.7
YFR01	IGD1	16.1	2.7	YGL15	AMS1	11.8	3.9	YLL02	HSP1	12.4	2.4	YOR12	GCY	14.8	3.0
YLR34	DIC1	15.7	4.1	YAL06	BDH1	11.7	2.5	YER03	PHM8	12.4	3.3	YPR00	PDH	14.5	1.7
YEL03	CYC7	14.9	2.7	YIL172	FSP2	11.6	2.6	YBR11	RAD1	11.9	2.4	YBL07	ATG	14.4	2.8
YOR28	-	14.6	4.6	YJL22	FSP2	11.6	2.6	YJL03	LOH1	11.7	5.3	YGR14	---	14.1	2.0
YGL15	AMS1	14.5	3.8	YOL15	FSP2	11.6	2.6	YOL05	DDR2	11.3	8.3	YOR38	FIT2	14.0	4.8
YDR04	NRG1	14.3	3.2	YMR12	ADE1	11.6	3.5	YJR00	MHO	11.3	2.4	YJR00	---	13.9	2.2
YOR17	DCS2	14.3	3.7	YGR24	SOL4	11.6	2.8	YOR17	DCS2	11.1	3.9	YDL08	NDE	13.8	2.1
YHR00	SOD2	14.3	2.2	YFL02	PAU5	11.3	3.9	YAL06	BDH1	11.0	1.9	YGR24	SOL	13.8	5.9
YER05	PIC2	14.3	6.7	YKR05	YSR3	11.0	5.3	YLR17	TFS1	10.9	6.2	YGL15	AMS	13.5	4.3
YNL14	-	14.1	2.3	YAR01	ADE1	10.7	2.2	YML12	MSC1	10.8	4.7	YFL02	PAU	13.3	2.7
YDL04	STP4	13.9	3.5	YMR09	SNZ1	10.5	2.8	YBR10	VID24	10.8	2.6	YHR13	---	13.3	2.6
YER15	-	13.8	3.4	YGL06	PYC1	10.4	3.3	YMR17	PAI3	10.8	3.2	YLR20	HMX	12.7	4.3
YDL18	INH1	13.8	4.1	YDR03	-	10.4	4.2	YDR21	ADR1	10.8	2.6	YHR07	PCL5	12.7	2.0
YDR03	MIC14	13.7	2.2	YML11	NGL3	10.2	2.3	YGR13	TPO2	10.7	7.1	YMR19	---	12.6	3.2
YJR07	BNA2	13.5	5.2	YJR10	CPA2	10.2	2.4	YMR24	-	10.7	2.0	YER15	---	12.3	1.8
YMR19	ICY1	13.5	3.1	YDR40	DIT1	9.9	2.6	YLL01	KNS1	10.7	2.4	YDR26	DIN7	12.0	1.6
YLR25	GSY2	13.5	4.2	YLR26	BOP2	9.9	4.3	YER10	SSA4	10.5	2.9	YLR26	---	12.0	3.8
YOR03	HMS1	13.2	7.4	YCL03	HIS4	9.7	2.1	YCR02	HSP3	10.5	10.5	YCL02	---	11.8	2.9
YER03	PHM8	13.2	3.4	YLR23	THI7	9.7	2.2	YLR32	TMA1	10.5	6.4	YPL01	---	11.8	2.1
YKL04	PHD1	13.1	2.7	YER04	ERG2	9.6	2.6	YLR03	-	10.4	2.9	YFL05	AAD	11.6	3.8
YPR16	GPH1	13.1	12.1	YCR07	SSK2	9.6	2.5	YPR16	GPH1	10.4	9.3	YKL08	SRX	11.6	7.2
YJL13	-	13.1	3.6	YEL06	PRB1	9.6	2.7	YDR05	PST1	10.4	2.2	YMR09	SNO	11.5	3.4
YNL27	-	13.0	6.1	YPL08	-	9.5	3.2	YGL17	SPO1	10.3	3.5	YPR18	GDB	11.3	2.4
YJR12	RSF2	12.9	3.0	YJL16	FMP3	9.5	5.0	YIR02	DAL1	10.2	2.0	YJL08	ARG	11.2	1.9
YJR00	MHO1	12.5	2.3	YCR00	CIT2	9.4	4.1	YNL27	-	10.2	5.9	YPL18	UIP4	11.1	2.7
YHR00	QCR10	12.5	2.2	YPL01	IRC15	9.4	2.6	YLR05	OSW	10.1	2.4	YHL04	ARN	11.1	4.3
YGL11	-	12.3	2.9	YGR02	MTL1	9.4	2.0	YOR12	GCY1	10.0	1.9	YJL14	---	11.0	3.2
YDR34	HXT7	12.3	8.0	YDR45	TSA2	9.3	7.9	YHR13	ARO9	9.9	2.3	YKR09	SRL3	10.9	2.0
YDR34	HXT6	12.3	8.0	YIR03	DAL3	9.2	3.0	YAL06	GDH3	9.9	2.0	YJL13	---	10.9	2.7
YHR04	FSH1	12.2	2.0	YKL16	KDX1	9.1	6.6	YDR00	NTH1	9.9	2.7	YJL15	INO1	10.8	3.0
YNL12	ESBP6	12.1	2.8	YGR21	RTA1	9.1	5.3	YKL16	PIR3	9.9	4.8	YDL24	THI1	10.8	2.1
YMR19	-	12.0	3.7	YOR13	SIA1	9.1	1.9	YPL01	IRC1	9.9	2.3	YFL05	THI5	10.8	2.1
YKL16	PIR3	11.5	5.8	YIL116	HIS5	9.1	2.0	YML10	TSL1	9.9	7.8	YJR15	THI1	10.8	2.1
YKL06	MSN4	11.4	3.5	YMR18	RGM	9.0	2.0	YDR25	HSP7	9.6	3.2	YNL33	THI1	10.8	2.1
YKL08	SRX1	11.4	3.4	YMR10	YPK2	8.9	2.1	YEL06	PRB1	9.6	2.6	YCR02	HSP	10.8	8.4
YLR32	TMA10	11.4	5.6	YMR09	SNO1	8.9	3.9	YMR0	-	9.5	2.4	YKL10	---	10.7	2.5
YBL07	ATG8	11.4	2.2	YGL11	-	8.8	2.5	YGL15	AMS1	9.5	2.4	YML05	HUG	10.6	17.4
YOR05	-	11.3	1.9	YHR04	DOG1	8.8	1.9	YLR25	GSY2	9.3	3.2	YHR00	SOD	10.6	1.8
YLR24	ERF2	11.2	1.6	YDR05	PST1	8.7	1.8	YHR13	-	9.3	2.2	YLR14	---	10.5	3.5
YLR29	-	11.1	3.2	YDL24	THI13	8.7	2.3	YJL03	IRC1	9.3	3.5	YGR17	---	10.4	2.2
YOR37	ALD4	11.1	2.2	YFL05	THI5	8.7	2.3	YHR20	CRG1	9.3	1.7	YJR10	CPA	10.4	1.8
YOR22	AIM41	10.9	1.8	YJR15	THI1	8.7	2.3	YDL22	FMP4	9.2	3.1	YJL16	---	10.4	4.1
YML00	-	10.8	1.8	YNL33	THI12	8.7	2.3	YMR30	ADH2	9.1	1.9	YLL02	HSP	10.4	1.9
YGR13	TPO2	10.8	4.8	YOR38	-	8.6	2.2	YOR05	-	9.1	1.8	YDR51	GRX	10.3	1.5
YBR05	REG2	10.7	2.7	YPL27	-	8.6	2.2	YGR02	MTL1	9.1	2.8	YDR40	DIT1	10.2	2.2
YBR06	TIP1	10.6	1.6	YER01	TIR1	8.6	3.2	YJL16	FMP3	9.1	4.2	YAL06	BDH	10.2	1.6
YIL107	PFK26	10.6	2.4	YLL05	-	8.6	2.3	YGR25	GND2	9.0	2.9	YNL16	YGP	10.2	2.8

YHR07	PCL5	10.5	1.9	YMR2	GTO	8.5	2.9	YPR1	GDB	9.0	2.3	YHL02	RIM	10.2	3.0
YLL01	KNS1	10.3	2.4	YNR0	CIT1	8.5	2.5	YFR01	-	9.0	2.6	YER0	SER	10.1	2.0
YKL15	MCR1	10.3	1.7	YDR3	ARO	8.4	2.3	YAR0	SWH	8.9	1.7	YJL11	NCA	10.1	7.5
YBR0	FMP2	10.3	2.5	YBR0	-	8.4	1.9	YOR2	HER	8.9	2.0	YOR2	ISU2	10.1	1.8
YDR0	TGL2	10.2	2.7	YJR07	BNA2	8.4	3.5	YMR2	GAD	8.9	6.1	YOR3	PUT	9.9	1.8
YKR0	MTD1	10.1	1.8	YOR1	ADE	8.4	1.9	YBR2	THI2	8.8	1.8	YER17	TMT	9.9	2.0
YGL05	PKP2	10.1	1.8	YAL06	GDH	8.3	2.2	YDL12	-	8.8	1.7	YOR1	DCS	9.9	3.1
YDR2	MTH1	10.1	3.0	YIR02	DAL1	8.2	2.2	YGR0	ROM	8.7	2.3	YJR00	---	9.8	5.3
YGL22	EDC1	10.1	2.2	YLR14	-	8.2	3.2	YAL05	FLC2	8.7	1.5	YNL09	YPT	9.8	2.2
YNL10	LEU4	10.0	1.8	YHL04	ARN	8.2	4.4	YLR29	-	8.7	2.8	YJR07	BNA	9.8	3.0
YKL08	MDH1	9.9	1.7	YFL05	-	8.2	1.7	YDL04	KNH	8.6	1.8	YPR1	GPH	9.7	5.7
YOL05	DDR2	9.9	5.3	YMR3	ADE	8.2	1.9	YBR2	-	8.6	2.6	YGR1	RTS	9.7	3.1
YJL16	FMP3	9.8	4.2	YNL11	CYB	8.1	1.7	YDR0	FMP	8.6	3.3	YLR19	---	9.7	1.8
YDR2	COX2	9.7	1.7	YOR3	-	8.1	2.4	YDR3	-	8.5	2.9	YER0	PHM	9.6	2.4
YLR08	ALT1	9.6	2.4	YLR41	PUN1	8.1	1.9	YCR0	TRX	8.5	1.7	YMR0	ECM	9.6	1.4
YLR14	-	9.5	3.4	YNL01	SPO1	8.1	2.2	YJL04	-	8.4	2.4	YCL03	ATG	9.5	1.6
YOR3	FIT3	9.4	2.5	YOL05	DDR	8.1	4.9	YHR0	-	8.4	2.4	YLR05	OSW	9.4	2.3
YDR0	GCV1	9.4	4.5	YER0	CEM	8.0	2.3	YFR01	IGD1	8.4	2.9	YKL07	---	9.3	4.5
YDL02	GPM2	9.4	8.9	YBR1	YSW	8.0	2.7	YGL0	PYC	8.4	2.1	YKR0	PET	9.3	2.8
YIL11	RPI1	9.3	2.0	YLR32	TMA1	7.9	4.1	YLR14	-	8.3	3.6	YDR21	ADR	9.3	2.1
YER07	-	9.3	2.5	YLL01	KNS1	7.9	2.3	YMR0	-	8.3	1.9	YMR2	GTO	9.3	2.8
YLR34	GAS2	9.3	1.7	YBL10	SRO	7.9	2.3	YDL08	NDE	8.3	1.6	YOR3	PDR	9.2	1.6
YLR23	THI7	9.3	1.6	YJL07	PRY1	7.9	2.2	YJL14	-	8.2	1.9	YDL24	AAD	9.1	2.5
YMR0	SNO1	9.3	2.6	YAL05	FLC2	7.8	1.9	YPL22	FMP	8.1	1.9	YFL05	AAD	9.1	2.5
YDR1	SDH4	9.2	1.7	YEL07	-	7.8	2.8	YMR1	PGM	8.0	6.2	YGR0	MTL	9.0	1.6
YNL09	YPT53	9.2	2.6	YPR01	-	7.8	1.9	YLR36	GRX	7.9	1.8	YLR41	---	9.0	1.6
YMR0	SNZ1	9.2	1.9	YBL00	-	7.7	2.3	YDL04	STP4	7.9	3.1	---	---	9.0	7.6
YKR0	KTR2	9.2	1.8	YBL00	-	7.7	2.3	YDL24	THI1	7.9	1.8	YOR0	---	9.0	1.7
YDR0	GAL3	9.2	1.8	YDR1	-	7.7	2.3	YFL05	THI5	7.9	1.8	YDL16	UGX	8.9	3.4
YKL17	MRPL	9.2	1.4	YMR0	-	7.7	2.3	YJR15	THI1	7.9	1.8	YDL19	---	8.9	1.5
YKR07	ECM4	9.1	3.3	YMR0	-	7.7	2.3	YNL33	THI1	7.9	1.8	YDR0	PST	8.9	1.5
YER0	CEM1	9.1	2.0	YNL28	-	7.7	2.3	YAL03	CYC	7.9	1.9	YLR17	IDP2	8.9	1.8
YFL03	AGX1	9.1	1.8	YNL28	-	7.7	2.3	YBR1	SSE	7.8	2.5	YOR3	COT	8.8	1.6
YEL02	RIP1	9.0	1.8	YNR0	ATO2	7.7	3.0	YLR21	CPR	7.8	1.5	YLR03	SMF	8.8	1.6
YML12	MSC1	9.0	2.5	YMR1	PAI3	7.6	2.9	YDL07	MRK	7.8	1.6	YLR13	TIS1	8.7	3.5
YGR1	CLB1	9.0	1.7	YMR0	PLB2	7.6	2.1	YJL13	-	7.8	2.3	YFL02	PAU	8.6	2.1
YPL22	FMP4	9.0	1.8	YIL11	PRM	7.6	2.3	YMR2	HOR	7.7	2.0	YBR2	GPX	8.6	2.2
YDR3	-	8.9	3.0	YHR0	MSC	7.6	1.9	YOR3	CPA	7.7	1.8	---	---	8.6	6.6
YBR0	YRO2	8.9	4.2	YBR0	NRG	7.5	2.1	YJL01	-	7.6	1.7	YLR29	ECM	8.5	1.7
YPR1	QCR2	8.9	1.8	YER0	PHM	7.5	2.3	YBR0	UGA	7.6	1.6	YOR2	---	8.5	1.5
YOR1	SIA1	8.8	1.6	YAR0	PAU7	7.4	2.7	YNL27	GOR	7.6	2.7	YDL11	TMA	8.5	2.4
YKL10	LAP4	8.8	4.1	YPR0	CIT3	7.3	1.9	YDL02	GPM	7.6	6.4	YOR2	---	8.5	2.3
YOL0	ATG1	8.8	2.1	YOR2	PTP2	7.3	2.1	YJL11	NCA	7.6	4.7	YFL03	AGX	8.4	1.7
YLL04	SDH2	8.8	1.8	YCR0	KIN8	7.3	2.0	YGL17	MPT	7.6	1.4	YLR17	TFS	8.3	3.4
YJR07	OPI3	8.7	3.3	YJR15	AAD1	7.3	1.9	YML11	NGL	7.5	1.5	YNL23	YTP	8.3	2.0
YOR0	-	8.7	1.4	YGR0	NQM	7.2	2.4	YAL05	ACS	7.5	2.2	YDR4	TSA	8.3	5.4
YGR1	QCR9	8.7	1.9	YGR2	ADE	7.2	1.7	YOR1	SIA1	7.5	1.4	YBL00	---	8.2	1.7
YBR2	LDH1	8.7	1.7	YBR1	SMP	7.2	1.9	YOR2	-	7.5	2.3	YBL00	---	8.2	1.7
YDL11	TMA1	8.7	2.5	YGR0	SPR	7.2	3.3	YKL10	LAP4	7.4	3.5	YDR1	---	8.2	1.7
YKR01	-	8.7	2.0	YKR0	MTD	7.2	1.7	YLL03	UBI4	7.4	3.1	YMR0	---	8.2	1.7
YDR2	-	8.6	1.4	YPR0	GLN1	7.2	1.7	YBR0	HSP	7.4	19.2	YMR0	---	8.2	1.7
YKL01	ATP7	8.6	1.6	YPL08	RLM1	7.1	1.8	YJR09	-	7.4	2.3	YNL28	---	8.2	1.7
YFL02	PAU5	8.6	2.1	YHR0	SLT2	7.1	1.9	YPR0	PDH	7.4	1.6	YNL28	---	8.2	1.7
YMR2	HOR7	8.6	2.1	YER0	MEI4	7.1	2.6	YCR0	KIN8	7.4	1.7	YDR1	HSP	8.2	3.9
YDR0	-	8.6	2.6	YFR01	IGD1	7.1	1.8	YMR2	MRP	7.4	1.6	YBR2	---	8.2	1.9
YPL01	IRC15	8.6	2.0	YFR01	-	7.0	1.7	YBR2	OM1	7.3	2.0	YDL12	---	8.1	1.5
YMR2	-	8.5	1.7	YIL01	-	6.9	1.8	YGL2	-	7.3	2.7	YBL06	PRX	8.1	2.8
YGR0	MTL1	8.5	1.6	YOR0	TIR2	6.8	2.0	YNL23	YTP1	7.3	2.1	YBR0	UGA	8.1	1.5
YJL05	TDH1	8.4	5.6	YGR0	ERG	6.8	1.7	YCL03	GRX	7.3	1.9	YDR0	NTH	8.0	2.1
YMR1	DDR4	8.4	2.3	YOR2	-	6.8	2.6	YEL04	PAU	7.2	1.8	YIL10	PFK	7.9	2.1
YMR1	DDR4	8.4	2.3	YKL16	PIR3	6.8	3.4	YHR01	MIP6	7.2	1.9	YDR2	HSP	7.9	2.5
YNL17	MDG1	8.4	1.9	YGR1	RTS3	6.8	2.8	YPR0	ATH1	7.2	2.2	YLR22	---	7.9	1.3
YGR2	KEL2	8.4	1.6	YHL04	ARN1	6.7	3.1	YER0	EDC	7.1	2.1	YGR1	BTN	7.9	2.5
YER1	SSA4	8.3	2.2	YGR0	SCM	6.7	2.7	YLL00	COX	7.1	2.7	YOR2	PTP	7.8	1.8
YMR1	MRPL	8.3	1.6	YHR0	RSC	6.7	1.8	YHR1	-	7.1	4.8	YKL15	MCR	7.8	1.5
YGL0	PYC1	8.3	2.0	YHR0	RSC	6.7	1.8	YDL11	TMA	7.1	2.1	YIL06	RNR	7.8	3.0
YLR17	TFS1	8.3	3.5	YOR3	-	6.7	2.1	YBL06	PRX1	7.1	2.6	YCR0	TRX	7.7	1.2
YCL03	GRX1	8.3	1.9	YPL27	-	6.7	2.1	YDR0	TPS2	7.1	2.9	YEL04	PAU	7.6	1.8
YBL06	PRX1	8.2	2.9	YIL10	PFK2	6.7	2.1	YJR07	MOG	7.0	1.6	YOL0	ARG	7.6	3.8
YJR06	-	8.2	1.4	YER1	-	6.6	1.8	YPR0	SMK	7.0	1.6	---	---	7.5	16.0
YAL02	FRT2	8.1	2.2	YMR1	GCV	6.6	3.4	YLR06	-	7.0	1.5	YMR3	DIA1	7.5	1.4

YDL16	UGX2	8.1	3.0	YNL29	RIM2	6.6	1.9	YOR18	GSP2	7.0	3.2	YDR07	SED	7.5	1.4
YJR15	DAN1	8.1	4.4	YLL05	FRE6	6.6	1.7	YDL16	UGX2	7.0	2.8	YML11	NGL	7.5	1.7
YPR03	-	8.1	2.4	YGR15	-	6.6	2.1	YER06	CEM1	6.9	1.8	YGR07	ROM	7.4	1.9
YDR40	DIT1	8.0	2.1	YPR03	-	6.5	2.4	YHR19	AIM4	6.9	1.5	YDR39	---	7.4	1.5
YNL27	GOR1	8.0	2.6	YPL26	DIP5	6.5	2.3	YJL15	SNA3	6.9	2.0	YDR07	---	7.4	5.7
YDR00	NTH1	8.0	2.1	YLR20	HMX1	6.5	2.4	YNL01	PBI2	6.8	3.4	YBR07	HSP	7.4	18.0
YJR07	MOG1	8.0	2.0	YHR16	YAP1	6.5	1.9	YLR17	IDP2	6.8	1.5	YAL06	GDH	7.3	1.6
YPR18	GDB1	8.0	2.2	YBL07	ATG8	6.5	1.9	YKR04	FMP4	6.8	2.0	YBL00	---	7.3	1.5
YKL19	MIA40	7.9	1.4	YGL00	ERG2	6.5	1.8	YGR00	STF2	6.8	3.3	YBR01	---	7.3	1.5
YOR04	IRC23	7.9	2.0	YDR25	YAP6	6.4	2.3	YHR08	RTC3	6.8	6.2	YDR26	---	7.3	1.5
YPR09	-	7.9	2.0	YAR03	YAT1	6.4	2.7	YFL02	PAU5	6.8	1.8	YER16	---	7.3	1.5
YNR00	CIT1	7.9	2.1	YLR35	ADE1	6.4	1.6	YOR02	HSP1	6.7	1.5	YGR02	---	7.3	1.5
YBR10	VID24	7.9	1.8	YHL00	-	6.4	4.2	YIR03	DAL3	6.7	2.0	YGR16	---	7.3	1.5
YOR17	GAC1	7.9	3.5	YJL11	-	6.4	4.2	YER05	PIC2	6.7	2.6	YJR02	---	7.3	1.5
YPL17	OYE3	7.9	3.2	YOR22	HER1	6.3	1.8	YHR08	KSP1	6.7	1.4	YLR03	---	7.3	1.5
YML11	NGL3	7.8	1.6	YHR13	-	6.3	1.9	YGR25	-	6.6	1.7	YMR04	---	7.3	1.5
YLL06	GTT2	7.8	3.6	YNL14	MEP2	6.3	2.6	YOR34	PYK2	6.6	1.7	YMR05	---	7.3	1.5
YGR17	-	7.8	1.8	YEL03	CYC7	6.3	1.9	YGR23	PEX2	6.6	1.6	YNL28	---	7.3	1.5
YLR17	-	7.7	1.9	YGL23	ADE5	6.3	1.6	YLR34	GAS2	6.6	1.8	YOR14	---	7.3	1.5
YKL15	RSM2	7.7	1.5	YER03	-	6.3	1.7	YAL00	FUN1	6.6	1.7	---	---	7.3	7.3
YBR23	-	7.7	2.3	YML12	MSC1	6.3	2.2	YJR10	URA8	6.6	1.5	YPL08	---	7.3	1.9
YOL08	DUF1	7.7	1.5	YOR18	SER1	6.3	1.5	YER04	MEI4	6.5	2.3	YJL01	---	7.3	1.5
YLR43	ECM30	7.6	1.4	YLR05	ERG3	6.3	1.9	YER05	GIP2	6.5	2.3	YER10	SSA	7.3	3.3
YLR27	-	7.6	1.4	YLL05	AQY2	6.2	2.5	YOR13	IDH2	6.5	1.5	YOR38	---	7.2	1.7
YOR22	ISU2	7.6	1.5	YOR31	COT1	6.2	1.7	YDR23	COX2	6.5	1.5	YPL27	---	7.2	1.7
YNL05	POR1	7.6	1.6	YCR10	AAD3	6.1	1.7	YFL05	-	6.5	1.5	YOR05	---	7.2	1.6
YER08	DOT6	7.6	1.9	YDR24	AMD2	6.1	1.8	YIL120	QDR1	6.5	1.4	YML12	MSC	7.1	4.2
YOR39	-	7.6	2.2	YPL21	THI6	6.1	1.5	YKR07	ECM4	6.5	2.4	YOR38	---	7.1	1.8
YAL01	PSK1	7.6	1.7	YLL02	HSP1	6.1	1.8	YPL14	PPT2	6.5	2.0	YIR03	LYS1	7.1	1.5
YJR05	PTK2	7.6	1.6	YER02	YAT2	6.1	2.0	YPR12	-	6.5	1.9	YDR35	TRR	7.0	1.3
YJL13	AIM23	7.6	1.7	YCR04	ARE1	6.1	1.6	YJL08	IML2	6.4	1.8	YKR07	ECM	7.0	2.5
YML09	RPM2	7.6	2.0	YBR24	THI2	6.0	1.7	YDR00	GAL3	6.4	1.5	YDL01	---	7.0	1.3
YNL01	PBI2	7.5	3.7	YER08	SER3	6.0	2.3	YOR31	FAA1	6.4	1.9	YDR08	AFR	7.0	1.8
YER04	ERG28	7.5	1.7	YCL03	ATG2	6.0	1.7	YPL10	MSD1	6.4	1.5	YLR25	GSY	6.9	2.0
YBR16	SSE2	7.5	2.6	YMR25	HOR7	6.0	2.0	YGL08	-	6.4	2.2	YIL136	OM4	6.9	1.9
YML11	ATR1	7.5	1.5	YGL26	COS1	6.0	2.2	YJL05	TDH1	6.4	3.9	YGR21	RTA	6.9	2.9
YFL06	COS4	7.4	1.6	YGL24	PDE1	6.0	1.8	YHL02	RIM4	6.4	2.0	YDR04	NRG	6.9	1.9
YGR29	COS6	7.4	1.6	YOR01	AUS1	6.0	1.9	YJR12	RSF2	6.4	2.0	YMR09	---	6.9	2.3
YNL33	COS1	7.4	1.6	YLR25	HAP1	6.0	1.6	YJL14	YAK1	6.4	1.6	YNL27	---	6.9	2.4
YDL02	-	7.4	1.7	YFR01	-	5.9	1.9	YEL01	GLC3	6.4	2.1	YNR00	ATO	6.9	2.2
YFL05	AAD6	7.4	1.5	YJR00	MHO	5.9	1.6	YDL18	INH1	6.3	2.2	YOL11	---	6.9	1.8
YMR18	GCV2	7.4	3.3	YKL21	SRY1	5.9	2.5	YGL08	MMS	6.3	1.7	YKR09	UBP	6.8	1.6
YMR08	YTA12	7.4	1.3	YOL11	MCH4	5.9	2.1	YMR19	SPG5	6.3	1.7	YLL01	KNS	6.8	1.9
YBR07	HSP26	7.4	18.1	YIL083	CAB2	5.9	1.9	YFR05	HXK1	6.3	4.9	YBR28	---	6.8	2.1
YGR04	NQM1	7.4	2.0	YBR20	DUR1	5.9	1.9	YEL06	HPA3	6.3	2.2	YDR05	UBC	6.7	1.6
YBR00	COQ1	7.4	1.3	YLR30	ACO1	5.9	1.8	YDR21	AHA1	6.3	1.8	YBL07	SSA	6.7	2.8
YKR05	-	7.4	1.3	YMR29	TDA1	5.9	2.1	YIL136	OM45	6.3	1.7	YMR18	---	6.7	1.9
YCR00	CIT2	7.3	2.6	YDR34	HXT3	5.8	1.5	YDR45	TSA2	6.3	3.6	YGR20	TRX	6.7	1.3
YBR25	-	7.3	1.8	YKR03	GAP1	5.8	3.0	YKR04	PET1	6.3	2.4	YKR01	---	6.7	1.7
YJL03	LOH1	7.3	2.6	YOR06	-	5.8	1.8	YNR04	-	6.2	2.0	YOL15	HXT	6.7	1.9
YBR14	RTC2	7.3	5.6	YDL13	STF1	5.8	3.2	YDR07	PET1	6.2	1.8	YMR25	GAD	6.6	3.5
YLR06	MEF1	7.3	1.4	YDL02	GPM2	5.8	4.5	YLR29	ECM3	6.2	1.6	YOR06	---	6.6	1.6
YOR38	-	7.2	1.8	YKR07	ECM4	5.8	2.5	YLR30	CDA1	6.2	3.6	YGR04	---	6.6	5.3
YPL27	-	7.2	1.8	YGR17	ERG1	5.8	1.6	YOR04	RSB1	6.1	2.9	YGR00	STF2	6.5	3.0
YMR25	COX7	7.2	1.7	YIL013	PDR1	5.8	1.6	YJR07	OPI3	6.1	2.4	YOR37	ALD4	6.5	1.6
YEL07	-	7.2	2.1	YKL10	LAP4	5.7	2.9	YLR26	BOP2	6.1	1.9	YBR16	SSE	6.5	2.4
YNL20	-	7.2	2.8	YOR17	DCS2	5.7	2.0	YHR18	PEX1	6.1	1.9	YGL17	SPO	6.5	1.8
YMR18	-	7.2	2.2	YNL09	YPT5	5.7	2.0	YER04	ERG2	6.1	1.6	YMR10	PGM	6.5	4.0
YJL14	YAK1	7.2	1.6	YGR23	KEL2	5.7	1.6	YIL056	VHR1	6.1	1.3	YER14	MAG	6.4	1.4
YDR07	SED1	7.2	1.5	YER05	GIP2	5.7	2.2	YPL18	UIP4	6.1	2.1	YKL08	MDH	6.4	1.3
YDL13	STF1	7.2	3.6	YJL07	-	5.7	2.3	YLR14	PUT1	6.0	1.5	YOR38	---	6.4	1.6
YFL01	-	7.2	1.7	YLR14	PUT1	5.7	1.7	YNR01	ARE2	6.0	2.0	YPL27	---	6.4	1.6
YLL02	TPO1	7.1	1.6	YER09	IES5	5.7	1.8	YCR04	ARE1	6.0	1.5	YBR20	FTH1	6.4	1.3
YOR06	CYT1	7.1	1.8	YMR10	SRT1	5.7	3.1	YDL13	STF1	6.0	3.0	YMR25	HOR	6.3	1.7
YAL00	FUN14	7.1	1.6	YDL21	GDH2	5.7	1.9	YJL08	SIP4	6.0	2.5	YFL06	COS	6.3	1.4
YKR04	PET10	7.1	2.3	YLR08	ALT1	5.7	1.7	YDR12	SAC6	6.0	1.3	YGR29	COS	6.3	1.4
YFR05	HXK1	7.1	5.3	YOR07	NRT1	5.6	1.6	YNR06	DSE4	6.0	1.7	YNL33	COS	6.3	1.4
YBR02	SCO2	7.1	1.7	YJL03	LOH1	5.6	2.4	YDR17	HSP4	6.0	3.2	YMR10	SRT	6.3	3.0
YML03	RCF1	7.0	1.5	YDL04	KNH1	5.6	1.6	YDR07	SED1	6.0	1.6	YHR16	PEX	6.3	1.8
YPL18	UIP4	7.0	1.9	YBL00	-	5.6	1.6	YBR19	AIM4	5.9	1.6	YOR19	PEX	6.2	1.5
YMR27	URA10	7.0	1.8	YBR01	-	5.6	1.6	YKL17	STE3	5.9	2.5	YGL00	PMC	6.2	1.4

YLR29	-	7.0	1.6	YDR26	-	5.6	1.6	YPL08	RLM	5.9	1.4	YHL03	---	6.2	2.4
YGR0	MSB2	7.0	1.7	YER16	-	5.6	1.6	YMR1	ALD3	5.9	1.5	YLR12	YPS	6.2	1.8
YDR47	JIP4	7.0	2.0	YGR02	-	5.6	1.6	YAL06	BDH2	5.9	3.5	YIR01	MET	6.2	1.8
YJL01	-	7.0	1.5	YGR16	-	5.6	1.6	YEL06	NPR2	5.9	1.3	YMR0	YET	6.2	1.8
YGR08	MRP1	6.9	1.4	YJR02	-	5.6	1.6	YOL11	RRI2	5.9	1.6	YOL01	CMK	6.2	1.8
YOR15	PET12	6.9	1.5	YLR03	-	5.6	1.6	YJL04	UBX6	5.9	1.5	YER05	GIP2	6.2	2.2
YDR03	RAD28	6.9	1.5	YMR0	-	5.6	1.6	YHR10	GRE3	5.9	2.3	YGR25	GND	6.1	2.5
YDR17	HSP42	6.9	3.3	YMR05	-	5.6	1.6	YMR18	-	5.9	1.9	YNL20	---	6.1	1.5
YMR1	GID8	6.9	1.6	YNL28	-	5.6	1.6	YKL15	MCR	5.9	1.5	---	---	6.1	9.2
YML10	TSL1	6.9	4.2	YOR14	-	5.6	1.6	YOL08	ATG3	5.8	2.2	YGL11	---	6.1	1.6
YGL19	COX13	6.9	1.6	YAL06	PAU8	5.6	1.9	YMR26	TPS3	5.8	1.6	YJL03	---	6.1	2.1
YEL06	PRB1	6.9	2.2	YBL10	PAU9	5.6	1.9	YBL05	CMC	5.8	1.8	YJL02	RNR	6.1	1.6
YFR01	-	6.9	1.4	YDR54	PAU1	5.6	1.9	YER03	ZRG8	5.8	2.3	YBR04	---	6.1	1.6
YNL23	YTP1	6.9	2.0	YGL26	PAU1	5.6	1.9	YER08	DOT6	5.8	1.6	---	---	6.1	1.5
YLL03	UBI4	6.9	2.8	YGR29	PAU1	5.6	1.9	YJL00	CYR1	5.8	1.7	YOR22	---	6.0	1.8
YLR05	-	6.9	2.3	YHL04	PAU1	5.6	1.9	YJL06	MPM	5.8	2.1	YIL155	GUT	6.0	1.7
YMR22	MRPL	6.9	1.4	YBR04	FMP2	5.6	1.8	YCR07	IMG2	5.8	1.4	YDR27	CCC	6.0	1.6
YNL27	ALP1	6.8	2.3	YNL14	-	5.5	1.6	YPL08	YDC1	5.8	2.0	YDR37	---	6.0	1.9
YGR13	TPO2	6.8	1.6	YGL01	ERG4	5.5	1.5	YGR24	LSC2	5.8	1.7	YAL00	SSA	6.0	1.2
YPR15	TPO3	6.8	1.6	YNR05	BIO3	5.5	1.4	YNL17	MDG	5.7	1.7	YPL24	HSP	6.0	1.4
YJL06	MPM1	6.8	2.1	YJL14	-	5.5	1.5	YEL05	SOM	5.7	2.6	YBL00	---	6.0	1.5
YBR12	ATG14	6.8	1.7	YJL01	-	5.5	1.5	YDR51	ACN9	5.7	1.8	YDR26	---	6.0	1.5
YLL02	HSP10	6.8	1.9	YHR07	ERG7	5.5	1.7	YDL20	RTN2	5.7	2.0	YDR36	---	6.0	1.5
YPL06	LPE10	6.8	1.5	YMR19	ICY1	5.5	1.9	YIL107	PFK2	5.7	1.7	YGR02	---	6.0	1.5
YPL17	MRPL	6.8	1.6	YDR00	NTH1	5.5	1.9	YNL27	ALP1	5.7	2.1	YJR02	---	6.0	1.5
YOR22	HER1	6.8	1.8	YLR13	TIS11	5.4	2.6	YDL18	-	5.7	1.5	YMR0	---	6.0	1.5
YER01	AFG3	6.7	1.4	YEL05	SOM1	5.4	2.8	YCL04	GLK1	5.6	3.0	YNL28	---	6.0	1.5
YOR08	TCB1	6.7	1.7	YIR03	LYS1	5.4	1.6	YGL03	PNC1	5.6	2.6	YER06	ARG	6.0	1.4
YDR53	HSP31	6.7	4.4	YDR25	HSP7	5.4	2.2	YNL06	AQR1	5.6	2.4	YHR03	SLT2	6.0	1.4
YJR09	-	6.7	2.1	YOR23	HES1	5.4	7.8	YBL02	PIM1	5.6	1.3	YER05	PIC2	6.0	2.2
YDL14	ATG9	6.7	1.8	YDL22	HBT1	5.4	1.8	YIL014	-	5.6	1.8	YJR10	SOD	6.0	1.2
YCR09	KIN82	6.7	1.9	YOL01	CMK2	5.4	2.0	YML13	ERO1	5.6	1.4	YPL15	PEP	6.0	1.6
YLR04	-	6.7	1.9	YDL03	GPR1	5.4	1.5	YPL13	GIP3	5.6	1.5	YGR17	---	6.0	2.6
YKL14	SDH1	6.6	1.8	YEL01	GLC3	5.3	1.7	YNR01	-	5.5	2.2	YDL20	RTN	5.9	4.1
YDL19	SNF3	6.6	1.7	YER03	EDC2	5.3	1.9	YFL03	AGX1	5.5	1.4	YLL03	UBI4	5.9	2.4
YJL04	MHP1	6.6	2.0	YPR14	-	5.3	1.5	YKL06	MSN	5.5	2.0	YLR23	THI7	5.9	1.3
YMR24	-	6.6	3.4	YMR31	DIA1	5.3	1.6	YLR24	CDD1	5.5	1.6	YDR50	PLM	5.9	1.7
YHR12	NDT80	6.6	1.7	YCL04	-	5.3	2.7	YCR06	-	5.5	2.0	YGL25	---	5.9	1.7
YOL05	AIM39	6.6	1.6	YJR07	OPI3	5.2	2.4	YDR00	SOK1	5.5	1.4	YOR02	CIN5	5.9	1.5
YOR31	FAA1	6.6	1.8	YMR23	DFG5	5.2	1.5	YOR15	PET1	5.5	1.4	YML10	TSL1	5.8	3.3
YCL03	ATG22	6.6	1.7	YDR01	GCV1	5.2	2.6	YOR38	FIT2	5.4	2.3	YHR08	---	5.8	6.0
YLL05	YCT1	6.5	1.6	YLR19	-	5.2	1.5	YOL02	IFM1	5.4	1.6	YMR18	RGM	5.8	1.4
YGR25	-	6.5	1.6	YKL06	YET1	5.2	1.6	YFL00	CDC	5.4	1.3	YKR06	CCP	5.8	1.7
YER05	RSM18	6.5	1.4	YDR37	-	5.2	2.1	YER16	-	5.4	1.8	YJL07	PRY	5.8	1.5
YHR05	CUP1-	6.5	1.5	YDL20	MGT1	5.2	1.5	YBR02	ETR1	5.4	1.5	YHL04	ARN	5.8	2.3
YHR05	CUP1-	6.5	1.5	YKR02	VPS5	5.2	1.6	YER06	HOR	5.4	3.0	YHR05	CUP	5.8	1.3
YEL05	SOM1	6.5	2.9	YAL06	-	5.2	2.1	YHR11	SET1	5.4	1.3	YHR05	CUP	5.8	1.3
YDR45	PPN1	6.5	1.3	YOR34	PYK2	5.1	1.7	YER05	PET1	5.4	2.1	YLR12	YPS	5.8	1.4
YDL08	NDE2	6.5	1.6	YDR07	SED1	5.1	1.5	YJL02	BBC1	5.3	1.5	YMR10	SPG	5.8	5.2
YPR11	-	6.5	1.5	YOR12	GCY1	5.1	1.5	YDL04	MRP	5.3	1.3	YKL06	YET	5.8	1.4
YHR16	YAP18	6.4	1.5	YPR00	SUT2	5.1	1.4	YOR30	-	5.3	1.7	YJL05	TDH	5.8	3.2
YGL00	PMC1	6.4	1.5	YHL01	DUR3	5.1	1.7	YGL16	CUP2	5.3	1.4	YLR34	---	5.8	1.6
YFL03	RIM15	6.4	1.6	YER01	HEM1	5.1	1.9	YGR13	TPO2	5.3	1.9	YAR01	---	5.8	1.4
YBR29	MAL33	6.4	1.8	YDL02	RTK1	5.1	1.7	YPR15	TPO3	5.3	1.9	YBR01	---	5.8	1.4
YDR07	FMP16	6.4	2.2	YML05	HUG1	5.1	4.5	YBL00	ECM	5.3	1.4	YBR01	---	5.8	1.4
YBL09	BNA4	6.4	1.6	YLR05	SHM2	5.0	1.6	YHR11	COX2	5.3	1.8	YDR09	---	5.8	1.4
YPL27	ATP15	6.4	1.3	YMR24	-	5.0	1.6	YBL10	ECM	5.3	1.5	YDR09	---	5.8	1.4
YOR18	GSP2	6.4	2.8	YOR10	CRC1	5.0	3.7	YBL07	KTI1	5.3	1.4	YDR21	---	5.8	1.4
YKR09	SRL3	6.4	1.6	YCR09	-	5.0	2.1	YFL03	RIM1	5.3	1.5	YDR21	---	5.8	1.4
YBL03	-	6.4	1.6	YCR10	AAD3	4.9	1.5	YNL12	ESBP	5.3	1.7	YDR26	---	5.8	1.4
YER02	GPA2	6.4	1.6	YOL16	AAD1	4.9	1.5	YDR03	RAD2	5.2	1.6	YDR26	---	5.8	1.4
YPR14	TAZ1	6.3	1.8	YMR06	ECM	4.9	1.4	YFL01	SMX2	5.2	1.4	YDR31	---	5.8	1.4
YGR13	PHB1	6.3	1.3	YHR04	NCP1	4.9	1.5	YKL12	MYO	5.2	1.6	YDR31	---	5.8	1.4
YPR03	GLN1	6.3	1.3	YGL20	CHC1	4.9	1.6	YIL101	XBP1	5.2	3.1	YDR36	---	5.8	1.4
YOL08	ATG34	6.3	2.3	YCR06	-	4.9	2.0	YJL19	RPS1	5.2	1.3	YDR36	---	5.8	1.4
YJR08	AIM24	6.3	1.3	YNL23	YTP1	4.9	1.8	YDL22	HBT1	5.2	1.7	YER13	---	5.8	1.4
YER06	RGI1	6.3	7.2	YML00	ERG6	4.9	1.4	YMR30	YME	5.2	1.4	YER13	---	5.8	1.4
YDL19	-	6.3	1.5	YDR00	GAL3	4.9	1.7	YER14	UBP5	5.2	1.3	YER16	---	5.8	1.4
YIL111	COX5	6.3	3.6	YGR17	ATF2	4.8	2.0	YPL08	-	5.2	1.7	YER15	---	5.8	1.4
YCL04	-	6.3	2.8	YLR05	-	4.8	1.8	YDL02	GPD1	5.2	1.8	YGR02	---	5.8	1.4
YHR05	FYV4	6.3	1.6	YHR00	ERG1	4.8	1.5	YER16	RPH1	5.2	1.4	YGR02	---	5.8	1.4

YNL30	BXI1	6.3	2.0	YDL24	AAD	4.8	1.9	YGL16	RAD	5.2	1.4	YGR0	---	5.8	1.4
YBR03	ATP3	6.2	1.5	YEL05	PCM	4.8	1.4	YDR4	DIT1	5.2	1.6	YGR0	---	5.8	1.4
YHR09	HXT4	6.2	5.7	YNL09	APP1	4.8	1.5	YCR07	SOL	5.2	1.7	YGR1	---	5.8	1.4
YER03	EDC2	6.2	1.8	YJL08	SIP4	4.8	2.2	YJR11	-	5.1	1.6	YGR1	---	5.8	1.4
YLR32	-	6.2	1.3	YGL25	-	4.8	1.8	YHR15	YSP1	5.1	1.4	YHR21	---	5.8	1.4
YGR19	XKS1	6.2	2.3	YMR1	-	4.8	1.9	YML00	MRP	5.1	1.4	YHR21	---	5.8	1.4
YPR06	JID1	6.2	1.6	YNL29	MSB	4.8	1.5	YGR0	UGA	5.1	2.1	YJR02	---	5.8	1.4
YGR1	-	6.2	1.6	YKL18	-	4.8	1.7	YNL12	-	5.1	1.6	YJR02	---	5.8	1.4
YLR29	ATP14	6.2	1.5	YOL01	IRC1	4.8	2.4	YLR37	STP	5.1	1.4	YJR02	---	5.8	1.4
YMR1	GAT2	6.2	1.8	YLR12	YPS	4.7	1.9	YIL157	COA	5.1	1.5	YJR02	---	5.8	1.4
YDL21	GDH2	6.2	1.8	YHR0	STP2	4.7	1.4	YER15	-	5.1	1.6	YLR15	---	5.8	1.4
YHL03	GUT1	6.2	1.4	YDR03	RAD	4.7	1.4	YDR1	STB	5.1	1.4	YLR15	---	5.8	1.4
YER01	TIR1	6.2	2.0	YMR1	GYL1	4.7	1.6	YPL24	GYP	5.1	1.4	YLR22	---	5.8	1.4
YIL01	MNT3	6.2	1.5	YHL01	-	4.7	2.0	YDR2	SSD	5.1	1.4	YLR22	---	5.8	1.4
YMR1	PGM2	6.2	4.1	YDL19	-	4.7	1.5	YGR0	CTT1	5.1	2.3	YLR25	---	5.8	1.4
YBL04	ECM1	6.1	2.6	YNR06	-	4.7	1.8	YGR1	UBR	5.1	1.3	YML0	---	5.8	1.4
YER1	MAG1	6.1	1.4	YPL22	FMP	4.7	1.5	YBR12	MRP	5.1	1.4	YML0	---	5.8	1.4
YPL24	HSP8	6.1	1.5	YDR2	AKR1	4.7	1.5	YLR06	MEF	5.1	1.3	YML0	---	5.8	1.4
YFR01	-	6.1	1.8	YGL0	PMC	4.7	1.5	YNR0	CIT1	5.1	1.7	YML0	---	5.8	1.4
YCL04	GLK1	6.1	3.2	YPL19	-	4.7	1.5	YHR0	TDA	5.1	1.6	YMR0	---	5.8	1.4
YMR1	RIM11	6.0	1.3	YNL27	GOR	4.7	2.0	YJL07	ARG	5.1	1.3	YMR0	---	5.8	1.4
YGR2	LSC2	6.0	1.6	YIL111	COX	4.6	3.0	YCL04	POF	5.1	1.6	YNL05	---	5.8	1.4
YPL16	SET6	6.0	1.7	YLL05	YCT1	4.6	1.6	YLR25	HSP	5.1	1.4	YOL1	---	5.8	1.4
YDR0	UBC5	6.0	1.6	YAR03	PRM	4.6	1.6	YDL13	LYS2	5.0	1.3	YOL1	---	5.8	1.4
YLL00	COX1	6.0	2.2	YJL11	NCA	4.6	2.9	YPR06	JID1	5.0	1.6	YOR1	---	5.8	1.4
YLR39	COX8	6.0	1.4	YPL27	-	4.6	8.0	YNL09	YPT5	5.0	1.7	YOR1	---	5.8	1.4
YDR2	DIN7	6.0	1.3	YIR03	GTT1	4.6	2.1	YKL09	CWP	5.0	2.8	YPL25	---	5.8	1.4
YGR0	STF2	6.0	2.7	YER07	-	4.5	1.8	YIL09	SGA	5.0	1.8	YPL25	---	5.8	1.4
YHR1	AIM18	6.0	1.9	YMR0	FMS1	4.5	1.4	YDL21	GDH	5.0	1.6	YPR1	---	5.8	1.4
YMR1	RGM1	6.0	1.7	YGR2	GND	4.5	1.8	YJL05	IKS1	5.0	1.4	YPR1	---	5.8	1.4
YLR34	-	5.9	1.8	YPR1	ASN1	4.5	1.5	YHL03	ECM	5.0	1.5	YPR1	---	5.8	1.4
YDR5	QCR7	5.9	1.4	YCL05	PRD1	4.5	1.5	YER0	-	5.0	4.7	YPR1	---	5.8	1.4
YML0	ATP18	5.9	1.4	YDL16	UGX	4.5	2.1	YOR2	HIS3	5.0	1.6	YPR1	---	5.8	1.4
YOR1	IDH2	5.9	1.4	YJL21	-	4.5	2.1	YKL15	-	5.0	2.3	YPR1	---	5.8	1.4
YER0	GIP2	5.9	1.9	YDR0	NRG	4.5	1.6	YFL01	MDJ1	5.0	1.3	YOR1	GAC	5.8	2.5
YMR1	ALD3	5.9	1.4	YMR1	GID8	4.5	1.5	YIL097	FYV1	5.0	1.7	YOR3	PYK	5.7	1.5
YMR1	PAI3	5.9	1.8	YPR1	GDB	4.4	1.6	YKL00	MRP	5.0	1.5	YBL10	---	5.7	1.4
YOR3	-	5.8	1.8	YKR01	-	4.4	1.6	YGR0	FMP	4.9	2.2	YCL01	---	5.7	1.4
YOL11	-	5.8	1.6	YKR0	UBP1	4.4	1.5	YKL16	MRP	4.9	1.6	YDR0	---	5.7	1.4
YPL15	PEP4	5.8	1.6	YJL02	BBC1	4.4	1.6	YBL07	NUP	4.9	1.3	YDR0	---	5.7	1.4
YMR2	GAD1	5.8	3.1	YBR0	QDR	4.4	1.4	YMR3	UBP	4.9	1.5	YDR21	---	5.7	1.4
YPR0	CIT3	5.8	1.4	YLR15	-	4.4	1.4	YLR25	SYM	4.9	2.4	YDR21	---	5.7	1.4
YKL08	CYT2	5.8	1.7	YLR21	CRR	4.4	2.2	YJR04	SSC	4.9	1.3	YDR3	---	5.7	1.4
YOR2	-	5.8	1.6	YOR2	RIM2	4.4	1.4	YOR2	PTP2	4.9	1.6	YFL00	---	5.7	1.4
YML12	COX1	5.8	1.7	YLL05	-	4.4	1.7	YDL07	VAM	4.9	1.3	YGR0	---	5.7	1.4
YJR02	BNA1	5.8	3.1	YKR0	PET1	4.4	1.9	YJL06	COA	4.9	1.8	YGR1	---	5.7	1.4
YPR1	PIN3	5.8	1.4	YGL1	VPS7	4.4	1.8	YKL21	SRY	4.9	2.0	YJR02	---	5.7	1.4
YDR0	GIS1	5.8	1.7	YNL27	ALP1	4.4	1.9	YLR43	ECM	4.9	1.5	YLR22	---	5.7	1.4
YGR1	CLD1	5.8	1.9	YMR0	AAC1	4.4	1.5	YPR1	MLC	4.9	1.3	YLR41	---	5.7	1.4
YGL24	PDE1	5.8	1.5	YOL01	PLB3	4.4	1.4	YNR0	ATO	4.9	1.9	YOR1	---	5.7	1.4
YNR0	PET49	5.7	1.5	YHR1	ERG	4.3	1.4	YLL02	TPO	4.9	1.5	YOR3	---	5.7	1.4
YDR4	NHX1	5.7	1.5	YLR12	YPS1	4.3	1.4	YML0	HUG	4.9	3.9	YPR1	---	5.7	1.4
YHR0	RTC3	5.7	4.3	YKL10	-	4.3	1.6	YPL09	MSY	4.9	1.4	YPR0	CIT3	5.7	1.4
YDR4	YHP1	5.7	2.3	YNL20	-	4.3	1.5	YJR13	XPT1	4.9	1.4	YAL05	---	5.7	1.3
YMR0	STB2	5.7	1.6	YDL23	GYP	4.3	1.5	YDR3	MRP	4.8	1.5	YDL02	---	5.7	1.5
YJR15	PGU1	5.7	2.4	YDR2	GTB1	4.3	1.4	YDR2	MSS	4.8	1.3	YGR1	---	5.7	1.9
YGL22	VID30	5.7	1.5	YLR17	TFS1	4.3	2.2	YLR17	-	4.8	1.3	---	---	5.7	3.4
YHR1	NVJ1	5.7	1.6	YLR02	IZH3	4.3	1.6	YJR15	AAD	4.8	1.3	YNL01	SPO	5.7	1.5
YIL13	OM45	5.7	1.5	YFL02	EPL1	4.3	1.4	YGR0	-	4.8	2.1	YDR5	EMI1	5.7	1.3
YEL01	UBC8	5.7	1.8	YPL07	MUK	4.3	1.5	YBR2	-	4.8	2.0	YLL06	GTT	5.7	2.6
YDR3	MRPS	5.7	1.4	YHR1	YCK1	4.3	1.4	YBR0	FMP	4.8	1.6	YLR10	---	5.7	1.4
YMR3	GLC8	5.7	1.3	YGR2	RAD	4.2	1.4	YLR12	YPS1	4.8	1.4	YOL1	---	5.7	2.5
YLR28	-	5.7	1.7	YNR0	-	4.2	1.5	YIL111	COX	4.8	2.8	YFR05	HXK	5.7	3.6
YPR0	HAA1	5.7	1.5	YOR3	SNC	4.2	1.5	YLR34	KAP	4.8	1.3	YCR0	---	5.7	1.5
YPL07	ATP4	5.7	1.3	YLL03	UBI4	4.2	2.1	YBR12	TPS1	4.8	2.0	YOR1	GSP	5.6	2.4
YCL02	HBN1	5.7	1.7	YKL07	-	4.2	1.8	YER15	-	4.7	1.7	YBR01	GAL	5.6	1.5
YDR1	STB3	5.7	1.5	YBR1	KTR4	4.2	1.4	YDR2	EXG	4.7	1.4	YDR01	GCV	5.6	2.4
YDL04	MRP1	5.6	1.3	YIR01	MET	4.2	1.7	YBR1	-	4.7	1.9	YJL08	IML2	5.6	1.4
YDR2	HSP7	5.6	2.0	YBL09	SCS	4.2	1.7	YOR2	RDL1	4.7	1.5	YIL101	XBP	5.6	2.9
YKL16	MRP4	5.6	1.7	YJL21	REE1	4.2	1.7	YLR43	CAR	4.7	1.6	YBR0	YRO	5.6	2.4
YBL00	-	5.6	1.4	YOL11	MSB	4.2	1.5	YPL16	MLH	4.7	1.5	---	---	5.6	8.5

YBR01	-	5.6	1.4	YOL08	ATG3	4.2	1.9	YLR39	CST9	4.7	1.2	---	---	5.5	1.6
YDR26	-	5.6	1.4	YDL24	AAD4	4.2	1.4	YPR00	HAA1	4.7	1.4	YGR13	TPO	5.5	2.5
YER16	-	5.6	1.4	YFL05	AAD1	4.2	1.4	YBR00	NTH2	4.7	1.7	YBR14	---	5.5	3.6
YGR02	-	5.6	1.4	YER04	ACA1	4.2	1.6	YEL04	YEF1	4.7	1.8	YKL09	MBR	5.5	1.4
YGR16	-	5.6	1.4	YPL15	PEP4	4.1	1.6	YAL06	-	4.7	1.8	YNL24	ZWF	5.5	1.4
YJR02	-	5.6	1.4	YKL17	STE3	4.1	2.0	YPR02	YME1	4.7	1.3	YKL06	MSN	5.5	1.4
YLR03	-	5.6	1.4	YPL11	GDE1	4.1	1.4	YOR16	PNS1	4.6	1.6	YMR17	DDR	5.5	1.7
YMR04	-	5.6	1.4	YPR00	ICL2	4.1	1.8	YKR08	MTD1	4.6	1.3	YMR17	DDR	5.5	1.7
YMR05	-	5.6	1.4	YHR07	PCL5	4.1	1.4	YJR06	-	4.6	1.2	YKL16	---	5.4	2.6
YNL28	-	5.6	1.4	YJR09	JSN1	4.1	1.5	YLR40	-	4.6	1.3	YJL03	---	5.4	2.0
YOR14	-	5.6	1.4	YGL03	-	4.1	1.6	YNL20	SPS1	4.6	2.0	YKL10	LAP4	5.4	2.3
YLR20	QRI5	5.6	1.4	YEL06	HPA3	4.1	1.8	YGL06	MRH	4.6	1.3	YGR25	---	5.4	1.5
YHR04	DOG2	5.6	1.8	YGR12	MEP1	4.1	1.5	YOR10	RGS2	4.6	1.7	YJR04	CYC	5.4	1.3
YDL24	THI13	5.6	1.5	YLR43	DIF1	4.1	1.5	YMR24	-	4.6	2.3	YPL23	---	5.4	2.3
YFL05	THI5	5.6	1.5	YLR25	NDL1	4.1	1.4	YFL02	PAU5	4.6	1.5	YIL055	---	5.4	1.7
YJR15	THI11	5.6	1.5	YLR21	-	4.1	1.6	YNR01	RCF2	4.6	1.4	YPR02	ATH	5.4	1.6
YNL33	THI12	5.6	1.5	YHR09	TRA1	4.1	1.4	YNL06	FKH2	4.6	1.4	YLR46	---	5.4	2.0
YNL25	MRPL1	5.6	1.4	YCR00	-	4.1	1.6	YLR23	THI7	4.6	1.4	YMR27	URA	5.4	1.5
YJL00	COX16	5.6	1.4	YOR18	GSP2	4.1	2.1	YMR19	CMC	4.6	1.4	YLL05	---	5.3	1.4
YDR00	-	5.5	1.3	YJL14	DAS1	4.1	1.5	YER13	-	4.6	1.4	YLR31	---	5.3	4.0
YOL04	RRT8	5.5	1.7	YOR12	UBP2	4.1	1.3	YNL20	-	4.6	2.1	YGR18	RNR	5.3	1.5
YDR11	MRPL1	5.5	1.4	YDR35	CNL1	4.1	1.4	YOR28	RDL2	4.6	1.4	YOR18	---	5.3	1.4
YDL20	MRPL1	5.5	1.3	YFR02	ROG3	4.1	1.7	YAR03	YAT1	4.6	1.7	YCL03	GRX	5.3	1.5
YLR27	DCS1	5.5	2.1	YBL00	-	4.0	1.5	YIR02	-	4.6	1.4	YDR27	MTH	5.2	1.7
YHL02	WSC4	5.5	4.3	YDR26	-	4.0	1.5	YNR03	RSM	4.6	1.8	YNR06	---	5.2	1.8
YBL02	-	5.5	1.5	YDR36	-	4.0	1.5	YDR49	MZM	4.6	1.6	YMR18	GCV	5.2	2.2
YPR15	CUR1	5.5	1.7	YGR02	-	4.0	1.5	YJR05	PTK2	4.6	1.4	YDL18	LYS2	5.2	1.3
YGR12	-	5.5	1.5	YJR02	-	4.0	1.5	YGL22	VID3	4.6	1.4	YHR03	---	5.2	1.6
YPR02	ATH1	5.5	1.7	YMR04	-	4.0	1.5	YMR12	RPL1	4.6	1.3	YDL02	GPM	5.2	3.3
YBL03	MRPL1	5.5	1.4	YNL28	-	4.0	1.5	YDL14	ATG9	4.6	1.5	YCL04	---	5.2	2.3
YKL09	-	5.5	1.8	YGR20	SER2	4.0	1.3	YFL01	-	4.6	1.6	YER03	EDC	5.2	1.6
YKL14	SDH3	5.5	1.4	YOR37	ALD4	4.0	1.5	YDR03	PST2	4.6	1.7	YDL07	MRK	5.2	1.4
YMR11	-	5.5	1.3	YOR05	-	4.0	1.3	YOL05	THI20	4.5	1.3	YER15	SPI1	5.1	3.3
YGR20	CIR1	5.4	1.4	YOR32	PDR1	4.0	1.4	YJL08	ARG3	4.5	1.6	YMR17	PAI3	5.1	2.9
YDR03	PST2	5.4	1.7	YPL15	PRM4	4.0	1.5	YCR06	TAH1	4.5	1.4	YCL04	GLK	5.1	2.4
YBR14	ADH5	5.4	2.1	YAL02	FRT2	4.0	1.7	YOR10	CRC1	4.5	2.9	YCR09	KIN8	5.1	1.4
YBR23	OM14	5.4	1.5	YCR03	PHO8	4.0	1.4	YBR06	TIP1	4.5	1.3	YOL16	---	5.1	2.3
YAL04	GCV3	5.4	1.7	YPR15	TDA6	4.0	1.9	YAL01	PSK1	4.5	1.5	YDR25	YAP	5.1	1.7
YGR05	-	5.3	1.8	YER07	TDA2	4.0	1.4	YJL14	DAS1	4.5	1.5	YDL24	AAD	5.1	1.8
YPR00	PDH1	5.3	1.4	YNL12	ESBP	4.0	1.6	YPL09	SSU1	4.5	1.7	YIL108	---	5.1	1.3
YAL00	SSA1	5.3	1.5	YPR00	AIM4	4.0	1.5	YFR02	ROG	4.5	1.6	YAL03	FUN	5.1	1.5
YLL02	POM3	5.3	1.5	YOR37	GDH1	4.0	1.3	YNR02	MRP	4.5	1.4	YBR23	---	5.1	1.4
YDR40	MRP20	5.3	1.5	YPR12	-	4.0	1.6	YDR34	HXT7	4.5	3.1	YFL01	MDJ	5.1	1.3
YDR51	GRX2	5.3	1.3	YOL15	ENB1	4.0	2.1	YDR34	HXT6	4.5	3.1	YMR05	STB	5.1	1.5
YJL03	IRC18	5.3	2.0	YJL10	SET4	4.0	6.0	YDR47	SNM	4.5	1.2	YIL024	---	5.1	1.4
YGL16	RAD54	5.3	1.4	YOR30	CPA1	4.0	1.5	YFR01	AIM1	4.5	1.3	YDR01	---	5.1	2.0
YGL18	COX4	5.3	1.6	YAR00	-	4.0	1.5	YPR03	-	4.5	1.7	YBL00	---	5.0	1.5
YGR17	-	5.3	2.3	YBL00	-	4.0	1.5	YMR05	STB2	4.5	1.5	YBR01	---	5.0	1.5
YJL08	ARG3	5.3	1.5	YBR01	-	4.0	1.5	YBR05	RFS1	4.5	1.6	YDR31	---	5.0	1.5
YGR22	PET54	5.3	1.5	YER13	-	4.0	1.5	YNL03	IDH1	4.5	1.6	YER16	---	5.0	1.5
YPL08	YDC1	5.3	1.8	YGR02	-	4.0	1.5	YKL09	-	4.5	1.8	YGR02	---	5.0	1.5
YBR05	AKL1	5.3	1.5	YGR16	-	4.0	1.5	YOR34	CIN1	4.5	1.5	YGR16	---	5.0	1.5
YPL02	RMI1	5.3	1.5	YJR02	-	4.0	1.5	YOL15	-	4.5	1.4	YHR21	---	5.0	1.5
YPL02	SKS1	5.3	2.0	YJR02	-	4.0	1.5	YCL02	HBN1	4.5	1.6	YJR02	---	5.0	1.5
YOR18	-	5.2	1.6	YLR15	-	4.0	1.5	YIL159	BNR1	4.5	1.4	YLR03	---	5.0	1.5
YHR04	DOG2	5.2	1.4	YML03	-	4.0	1.5	YAL00	VPS8	4.4	1.3	YML04	---	5.0	1.5
YHR04	DOG1	5.2	1.4	YMR04	-	4.0	1.5	YGR22	MRP	4.4	1.2	YMR04	---	5.0	1.5
YBR05	RFS1	5.2	1.7	YNL28	-	4.0	1.5	YAL03	FUN1	4.4	1.4	YMR05	---	5.0	1.5
YLR08	EMP46	5.2	2.3	YOL10	-	4.0	1.5	YLR17	RFX1	4.4	1.4	YNL28	---	5.0	1.5
YNR02	CPR8	5.2	1.3	YOR14	-	4.0	1.5	YGR10	GTF1	4.4	1.4	YOR14	---	5.0	1.5
YGR22	MRPL9	5.2	1.3	YPL25	-	4.0	1.5	YDL23	GYP7	4.4	1.4	YPR15	---	5.0	1.5
YJL05	IKS1	5.2	1.4	YLR27	-	4.0	1.4	YOR17	GAC1	4.4	2.4	YPR15	---	5.0	1.5
YER15	BUR6	5.2	1.3	YJR10	ECM2	4.0	1.5	YOL01	CMK2	4.4	1.6	YBL03	---	5.0	1.3
YKR05	GLG1	5.1	2.1	YOR04	GLO4	4.0	2.0	YGL11	ABC1	4.4	1.3	YMR11	---	5.0	1.4
YOR18	TUF1	5.1	1.5	YAR01	-	4.0	1.4	YNL30	BXI1	4.4	1.7	YPL17	OYE	5.0	2.6
YIL124	AYR1	5.1	1.5	YBR01	-	4.0	1.4	YLR07	FMP2	4.4	1.3	YIL176	PAU	5.0	1.4
YMR00	PLB2	5.1	1.5	YBR01	-	4.0	1.4	YLR39	ECM1	4.4	1.7	YJL22	PAU	5.0	1.4
YDR29	ATP5	5.1	1.3	YDR09	-	4.0	1.4	YHR10	KIC1	4.4	1.6	YNL20	SPS	5.0	1.9
YIL155	GUT2	5.1	1.5	YDR09	-	4.0	1.4	YER11	AVT6	4.4	2.0	YBR30	DAN	5.0	1.8
YBL10	-	5.1	1.4	YDR21	-	4.0	1.4	YPL02	RMI1	4.4	1.4	YDR00	GAL	5.0	1.3
YGL23	-	5.1	2.1	YDR21	-	4.0	1.4	YOL15	GRE2	4.4	1.5	YPL25	ICY2	5.0	1.7

YPL12	RNY1	5.1	2.3	YDR26	-	4.0	1.4	YMR19	ICY1	4.4	1.7	YKR05	MRS	5.0	1.3
YLR06	PET30	5.1	1.2	YDR26	-	4.0	1.4	YGR05	SPR3	4.4	2.1	YPL11	CAR	5.0	1.3
YPL00	LSP1	5.1	1.5	YDR31	-	4.0	1.4	YPL08	MOT3	4.4	1.4	YBR18	YPC	5.0	1.7
YMR27	ZDS1	5.1	1.3	YDR31	-	4.0	1.4	YMR06	ECM4	4.4	1.2	YDR34	HXT3	5.0	1.2
YBL10	-	5.1	1.4	YDR36	-	4.0	1.4	YFR03	CDC2	4.4	1.5	YNL27	ALP1	4.9	1.8
YCL01	-	5.1	1.4	YDR36	-	4.0	1.4	YDR43	CYM1	4.4	1.5	YGR13	TPO	4.9	1.3
YDR03	-	5.1	1.4	YER13	-	4.0	1.4	YOL07	EMI5	4.4	1.7	YPR15	TPO	4.9	1.3
YDR09	-	5.1	1.4	YER13	-	4.0	1.4	YDR05	TGL2	4.4	1.5	YIL169	---	4.9	2.5
YDR21	-	5.1	1.4	YER16	-	4.0	1.4	YMR17	ALD2	4.4	1.6	YOL15	---	4.9	2.5
YDR21	-	5.1	1.4	YER15	-	4.0	1.4	YCR07	SSK2	4.3	1.4	YNL11	---	4.9	1.7
YDR36	-	5.1	1.4	YGR02	-	4.0	1.4	YCR04	IMG1	4.3	1.5	YOL08	---	4.9	1.8
YFL00	-	5.1	1.4	YGR02	-	4.0	1.4	YMR06	NAT4	4.3	1.5	YJL14	YAK	4.9	1.5
YGR03	-	5.1	1.4	YGR03	-	4.0	1.4	YPL06	-	4.3	1.8	YBR02	SCO	4.9	1.4
YGR16	-	5.1	1.4	YGR03	-	4.0	1.4	YIL108	-	4.3	1.2	YKR08	MTD	4.9	1.3
YJR02	-	5.1	1.4	YGR16	-	4.0	1.4	YNL18	MRPI	4.3	1.4	YDL04	KNH	4.9	1.3
YLR22	-	5.1	1.4	YGR16	-	4.0	1.4	YDL11	-	4.3	1.7	YGL03	PNC	4.9	2.2
YLR41	-	5.1	1.4	YHR21	-	4.0	1.4	YHR11	-	4.3	1.5	---	---	4.9	4.9
YOR19	-	5.1	1.4	YHR21	-	4.0	1.4	YGR28	IMA1	4.3	1.3	YDR26	---	4.8	1.1
YOR34	-	5.1	1.4	YJR02	-	4.0	1.4	YJL159	HSP1	4.3	1.3	YDL13	STF1	4.8	2.3
YPR13	-	5.1	1.4	YJR02	-	4.0	1.4	YLR32	-	4.3	1.3	YGL18	ATG	4.8	1.5
YER11	SWI4	5.1	1.4	YJR02	-	4.0	1.4	YDR42	SNX4	4.3	1.3	YOR22	---	4.8	1.4
YML02	YML6	5.1	1.4	YJR02	-	4.0	1.4	YLR17	-	4.3	1.6	YJR13	STR	4.8	1.3
YFR01	GSY1	5.1	3.4	YLR15	-	4.0	1.4	YPL06	RGL1	4.3	1.3	YJL08	SIP4	4.8	2.0
YBR18	YPC1	5.1	1.7	YLR15	-	4.0	1.4	YJL07	-	4.3	1.5	YGR05	SPR	4.8	2.0
YKL05	MDM3	5.1	1.4	YLR22	-	4.0	1.4	YOL12	SMF1	4.3	1.4	---	---	4.8	4.6
YPR15	KRE6	5.0	1.3	YLR22	-	4.0	1.4	YPR00	HAL1	4.3	1.4	YLR32	PEX	4.8	1.3
YDL02	RTK1	5.0	1.7	YLR25	-	4.0	1.4	YMR08	VBA1	4.3	1.3	YDR42	ARO	4.8	1.3
YDR28	DPP1	5.0	1.5	YML04	-	4.0	1.4	YGR23	KEL2	4.3	1.3	YDL11	---	4.8	1.7
YOR22	RCN2	5.0	1.7	YML04	-	4.0	1.4	YHR16	YAP1	4.3	1.4	YHR15	SPO	4.8	1.3
YPL09	SSU1	5.0	1.7	YML04	-	4.0	1.4	YCR01	SRD1	4.3	1.4	YLR46	---	4.7	1.9
YER06	ICL1	5.0	1.5	YML03	-	4.0	1.4	YKR08	MRPI	4.3	1.4	YGR25	RAD	4.7	1.3
YML11	COQ5	5.0	1.4	YMR05	-	4.0	1.4	YOR27	RIM2	4.2	1.3	YOR03	PEP	4.7	1.5
YPR06	ROX1	5.0	1.4	YMR05	-	4.0	1.4	YMR19	SGS1	4.2	1.4	YIL097	FYV	4.7	1.4
YDR07	TPS2	5.0	2.0	YNL05	-	4.0	1.4	YLR30	ACO1	4.2	1.4	YOR13	SIA1	4.7	1.4
YBR16	CSH1	5.0	1.5	YOL10	-	4.0	1.4	YBR25	SPO2	4.2	2.0	YDL24	---	4.6	1.3
YDL23	GYP7	5.0	1.4	YOL10	-	4.0	1.4	YAL02	FRT2	4.2	1.7	YDL05	RAD	4.6	1.2
YDR07	PET10	5.0	1.6	YOR14	-	4.0	1.4	YOL08	DUF1	4.2	1.3	YOR38	RDR	4.6	1.2
YER09	AIM11	5.0	1.5	YOR14	-	4.0	1.4	YPR19	OPT2	4.2	2.0	YDR45	PPN	4.6	1.3
YML00	MRPL3	5.0	1.4	YPL25	-	4.0	1.4	YEL01	UBC8	4.2	1.6	YAR02	PAU	4.6	1.7
YJL00	CYR1	5.0	1.8	YPL25	-	4.0	1.4	YGL18	ATG1	4.2	1.5	YMR01	SPO	4.6	1.6
YLR40	-	5.0	1.2	YPR13	-	4.0	1.4	YHR00	SOD2	4.2	1.4	YJL03	SNX	4.6	1.2
YGL03	PNC1	4.9	2.3	YPR13	-	4.0	1.4	YNL02	ARK1	4.2	1.3	YLR21	CPR	4.6	1.2
YLR03	SMF3	4.9	1.5	YPR15	-	4.0	1.4	YIL065	FIS1	4.2	1.4	YPL11	GDE	4.6	1.3
YBR05	-	4.9	1.8	YPR15	-	4.0	1.4	YGL23	MTO1	4.2	1.4	YJL04	UBX	4.6	1.4
YKL15	-	4.9	2.2	YPR15	-	4.0	1.4	YOL08	PHM7	4.2	2.2	YAL06	SEO	4.6	1.6
YOR07	SGO1	4.9	1.2	YPR15	-	4.0	1.4	YMR12	-	4.2	1.3	YAR00	---	4.6	1.4
YKL06	YET1	4.9	1.3	YBR23	-	3.9	1.6	YNL24	ATG2	4.2	1.5	YBL00	---	4.6	1.4
YLR41	PUN1	4.9	1.3	YNL17	TDA7	3.9	1.4	YKL06	-	4.2	1.4	YBR01	---	4.6	1.4
YDL03	GPR1	4.9	1.3	YMR05	STB2	3.9	1.6	YDR05	UBC5	4.2	1.4	YER13	---	4.6	1.4
YJL10	MEF2	4.9	1.4	YOR01	-	3.9	1.4	YOR38	FIT3	4.2	1.8	YGR02	---	4.6	1.4
YHR05	COX6	4.9	1.4	YKR06	GPT2	3.9	1.6	YCR08	AHC2	4.2	1.2	YGR16	---	4.6	1.4
YMR31	TGL3	4.9	1.3	YGL01	PDR1	3.9	1.4	YKL09	MTC2	4.2	1.3	YJR02	---	4.6	1.4
YIL108	-	4.9	1.2	YOL11	RRI2	3.9	1.5	YMR04	YET2	4.1	1.6	YJR02	---	4.6	1.4
YHR09	-	4.9	1.7	YLR05	-	3.9	1.8	YJR11	RSM7	4.1	1.5	YLR15	---	4.6	1.4
YMR14	OSW5	4.9	1.4	YLR08	EMP4	3.9	2.0	YFR00	YFH7	4.1	1.6	YML03	---	4.6	1.4
YBR06	NRG2	4.8	1.5	YHR07	-	3.9	1.4	YPL17	TRE1	4.1	1.3	YMR04	---	4.6	1.4
YBR13	-	4.8	1.9	YJR05	RAD7	3.9	1.4	YCR03	PHO8	4.1	1.3	YNL28	---	4.6	1.4
YOL02	IFM1	4.8	1.5	YMR20	ERG2	3.9	1.3	YDR33	MSN5	4.1	1.3	YOL10	---	4.6	1.4
YJL15	SNA3	4.8	1.5	YKL05	MDM	3.8	1.5	YCL03	MXR2	4.1	1.7	YOR14	---	4.6	1.4
YJL11	NCA3	4.8	2.6	YNL03	IDH1	3.8	1.5	YFL04	-	4.1	1.4	YPL25	---	4.6	1.4
YPL07	UBP16	4.8	1.3	YDR21	ADR1	3.8	1.5	YNL28	HCH1	4.1	1.3	YLR10	AHP	4.6	1.2
YPL04	ISM1	4.8	1.4	YGR04	-	3.8	1.4	YOL00	SIN3	4.1	1.3	YNR06	---	4.6	1.6
YBR21	SDS24	4.8	2.6	YDL24	COS7	3.8	1.7	YER11	SWI4	4.1	1.5	YHR10	TRR	4.6	1.2
YGL03	OCH1	4.8	1.6	YJR16	COS5	3.8	1.7	YOR33	-	4.1	4.3	YLR08	EMP	4.6	2.0
YGR25	ENO1	4.8	2.0	YOR03	AKR2	3.8	1.4	YDR51	EMI2	4.1	1.9	YLR29	---	4.5	1.5
YJL16	QCR8	4.8	1.4	YLL06	GTT2	3.8	2.1	YDR31	TFB1	4.1	1.2	YDL21	GDH	4.5	1.5
YER14	COX15	4.8	1.7	YBR16	SSE2	3.8	1.7	YPR00	AIM4	4.1	1.4	YDR34	YPS	4.5	1.2
YOR02	-	4.8	1.4	YJL15	INO1	3.8	1.5	YKR09	SRL3	4.1	1.6	YMR13	RIM1	4.5	1.2
YDR04	RSM10	4.8	1.4	YKL20	MNN4	3.8	1.8	YER05	RSM1	4.1	1.3	YML13	---	4.5	1.6
YCL05	MOS1	4.8	1.4	YMR20	ERG1	3.8	1.5	YAL05	OAF1	4.1	1.3	YLR37	PSY	4.5	1.4
YMR02	MRPL3	4.8	1.4	YBR15	ICS2	3.8	1.5	YJL12	-	4.1	1.6	YKR03	GAP	4.5	2.0

YCR07	IMG2	4.8	1.3	YBL00	-	3.8	1.5	YNL05	POR	4.1	1.3	YLR25	NDL	4.5	1.3
YGR02	-	4.8	2.1	YBR01	-	3.8	1.5	YML12	COX	4.1	1.5	YOR37	GDH	4.4	1.2
YJR00	-	4.8	2.3	YDR31	-	3.8	1.5	YGR21	RSM	4.1	1.4	YJL15	HSP	4.4	1.3
YIL088	AVT7	4.8	1.5	YER16	-	3.8	1.5	YEL07	RMD	4.1	1.3	YOR38	FRE	4.4	1.9
YHR14	-	4.8	2.7	YGR02	-	3.8	1.5	YMR23	BCH	4.1	1.3	YBR29	PCA	4.4	1.7
YHR07	PPE1	4.7	1.7	YGR16	-	3.8	1.5	YJR03	PET1	4.1	1.5	YLR13	SLX	4.4	1.2
YDL04	KNH1	4.7	1.4	YHR21	-	3.8	1.5	YGR17	CBP	4.1	1.4	YLL05	FRE	4.4	1.3
YMR19	CLN1	4.7	1.6	YJR02	-	3.8	1.5	YGL03	-	4.1	1.3	YDR34	HXT	4.4	1.9
YJR10	RSM2	4.7	1.5	YLR03	-	3.8	1.5	YBR09	PHO	4.1	1.4	YDR34	HXT	4.4	1.9
YDR52	SNA2	4.7	1.5	YML04	-	3.8	1.5	YGR11	CLD1	4.0	1.6	---	---	4.4	3.1
YDR37	ATP17	4.7	1.5	YMR04	-	3.8	1.5	YOR09	ECM	4.0	1.3	YLR17	---	4.4	1.4
YNR00	SWM2	4.7	1.4	YMR05	-	3.8	1.5	YBR17	EHT1	4.0	1.6	YJR09	---	4.4	1.8
YBR02	ETR1	4.7	1.4	YNL28	-	3.8	1.5	YMR03	-	4.0	1.3	YIR038	GTT	4.4	1.8
YBR24	THI2	4.7	1.4	YOR14	-	3.8	1.5	YLR32	PEX	4.0	1.4	YKL14	MRP	4.4	1.3
YML12	NDI1	4.7	1.9	YPR15	-	3.8	1.5	YOR27	TPO	4.0	2.7	YIL169	---	4.4	1.8
YMR19	CMC4	4.7	1.4	YPR15	-	3.8	1.5	YCL01	-	4.0	1.4	YGL18	STR	4.4	1.7
YNL20	-	4.7	1.4	YNL01	PBI2	3.8	2.1	YOL14	ARG	4.0	1.3	YGR12	---	4.4	1.4
YPR12	AXL1	4.7	1.3	YGR20	CIR1	3.8	1.4	YDR50	PSP1	4.0	1.3	YDR35	GGA	4.4	1.4
YNL11	MLS1	4.7	1.8	YGR17	PSD2	3.8	1.3	YMR17	DDR	4.0	1.5	YHR13	ARO	4.4	1.1
YER06	THO1	4.7	1.3	YDR23	COX2	3.8	1.4	YMR17	DDR	4.0	1.5	YHR03	PUT	4.3	1.2
YOR38	FIT2	4.6	1.7	YDL13	RGT2	3.8	1.4	YLR05	-	4.0	1.8	YGL10	VPS	4.3	1.5
YHR03	VMA10	4.6	1.2	YBL09	BNA4	3.8	1.5	YOR15	-	4.0	1.6	YKL03	PTM	4.3	1.2
YMR05	AAC1	4.6	1.6	YDR35	GGA1	3.7	1.5	YPR04	THP3	4.0	1.3	YGL05	---	4.3	1.3
YHL02	AIM17	4.6	3.1	YIL066	RNR3	3.7	1.9	YIR034	LYS1	4.0	1.4	YJR09	JSN1	4.3	1.3
YFL02	GAT1	4.6	1.5	YHL02	AIM1	3.7	2.8	YJL153	INO1	4.0	1.4	YPL22	GRE	4.3	6.0
YIL070	MAM3	4.6	1.6	YGR06	ADE6	3.7	1.3	YPL22	FLC1	4.0	1.5	YOL03	---	4.3	1.4
YJR15	AAD10	4.6	1.3	YFR02	LSB3	3.7	1.4	YDR49	PKH1	4.0	1.5	YLR18	ATG	4.2	1.3
YDL24	AAD4	4.6	1.4	YCR10	AAD3	3.7	1.4	YOR12	UBP2	4.0	1.2	YJL093	HPR	4.2	1.2
YFL05	AAD16	4.6	1.4	YGR18	CRH1	3.7	1.6	YNL07	TOM	4.0	1.2	YBR25	RIB5	4.2	1.2
YDR06	OCA6	4.6	1.2	YER17	TMT1	3.7	1.5	YJR09	JSN1	4.0	1.5	YLR27	DCS	4.2	1.7
YKR09	PXL1	4.6	1.3	YGR13	FHN1	3.7	1.5	YLR43	MRP	4.0	1.6	YKL15	---	4.2	2.0
YDL01	GRX6	4.6	1.3	YGL02	PIB2	3.7	1.4	YDR53	HSP	4.0	2.5	YBR10	FES	4.2	1.2
YGR21	RSM27	4.6	1.3	YLL02	TPO1	3.7	1.4	YPR11	-	4.0	1.3	YKL10	HAP	4.2	1.4
YKR06	GPT2	4.6	1.6	YGR14	ECL1	3.7	1.4	YPL24	-	4.0	1.2	YLR26	LCB	4.2	1.3
YPL11	HOS3	4.6	1.3	YPL08	YDC1	3.7	1.7	YMR26	PPA2	4.0	1.4	YDR12	---	4.2	1.2
YJL134	LCB3	4.6	1.3	YLR18	ATG2	3.7	1.4	YGL11	-	4.0	1.4	YKL22	MCH	4.2	1.8
YMR18	MRPS	4.6	1.4	YBR00	RCR1	3.7	1.4	YGL01	JAC1	4.0	1.6	YER07	---	4.2	1.5
YDL13	SCM3	4.5	1.4	YPR08	GRS2	3.7	1.6	YGR25	RAD	4.0	1.4	---	---	4.2	4.3
YGR17	CBP4	4.5	1.4	YHR01	MIP6	3.7	1.5	YIR039	YPS6	3.9	1.5	YGR05	---	4.2	1.5
YJR05	-	4.5	1.3	YKL06	MSN	3.7	1.5	YCR07	-	3.9	1.4	YPL22	---	4.2	1.4
YHL02	RMD1	4.5	1.2	YOL15	HXT1	3.7	1.6	YDL08	-	3.9	1.4	YGR15	---	4.2	1.4
YIL157	COA1	4.5	1.4	YHR04	DOG	3.7	1.4	YNL13	CPT1	3.9	1.4	YKL02	GPX	4.2	1.8
YNL03	IDH1	4.5	1.4	YHR04	DOG	3.7	1.4	YGL24	PDE	3.9	1.3	YGL01	---	4.2	1.2
YDR29	MHR1	4.5	1.4	YFL04	-	3.7	1.4	YFR01	FAB1	3.9	1.4	YGL16	RAD	4.2	1.3
YMR27	SCS7	4.5	1.8	YLR17	-	3.7	1.4	YLR21	COA	3.9	1.7	YLR01	LOT	4.2	1.3
YOR33	MRS2	4.5	1.4	YER03	ZRG8	3.7	1.8	YDR42	ARO	3.9	1.3	YBL04	MOH	4.2	2.8
YNL32	KRE1	4.5	1.3	YIR00	-	3.6	1.8	YBR02	POA	3.9	1.2	YPR15	PIN3	4.2	1.3
YFR01	AIM13	4.5	1.3	YDR29	SSD1	3.6	1.4	YLR36	MDM	3.9	1.3	YER05	---	4.1	3.0
YJL164	TPK1	4.5	2.1	YNL20	-	3.6	1.9	YOR22	RCN	3.9	1.5	YNR01	---	4.1	1.6
YAL06	-	4.5	1.7	YOR28	RDL1	3.6	1.5	YHR00	QCR	3.9	1.4	YGR05	---	4.1	1.5
YLR12	YPS3	4.5	1.5	YDR52	SNA2	3.6	1.5	YMR13	GID8	3.9	1.3	YER06	---	4.1	3.6
YKL14	MRP8	4.5	1.4	YGL24	-	3.6	1.3	YPL21	THI6	3.9	1.3	YDR07	TPS	4.1	1.7
YKR07	MSA2	4.5	1.4	YER11	SWI4	3.6	1.5	YIL172	FSP2	3.9	1.3	YOL15	ENB	4.1	1.9
YDL18	-	4.5	1.3	YPL02	SKS1	3.6	1.6	YJL22	FSP2	3.9	1.3	YPL21	THI6	4.1	1.2
YJL18	-	4.5	1.3	YGL21	CLG1	3.6	2.0	YOL15	FSP2	3.9	1.3	YOR10	CRC	4.1	2.5
YGR06	COX18	4.5	1.5	YLR03	SMF3	3.6	1.4	YJR09	GRR	3.9	1.2	YEL01	UBC	4.1	1.5
YAL00	VPS8	4.5	1.3	YBR28	-	3.6	1.4	YCL05	MOS	3.9	1.4	YMR29	---	4.1	1.5
YOR29	YPK9	4.5	1.4	YJL17	CPS1	3.6	1.6	YBL04	EDE	3.9	1.3	YCR00	CIT2	4.1	1.6
YOR09	ECM3	4.4	1.4	YBR07	-	3.6	1.4	YFR02	PTR	3.9	1.4	YEL07	---	4.0	1.6
YFL02	PAU5	4.4	1.5	YGR07	ROM	3.6	1.5	YIL055	-	3.9	1.5	YDR51	EMI2	4.0	1.8
YJL180	ATP12	4.4	1.3	YAR04	SWH	3.6	1.3	YDL19	-	3.9	1.4	YLR02	SNF	4.0	1.2
YDR01	-	4.4	1.9	YMR25	-	3.6	1.4	YDR51	EMI1	3.9	1.3	YCR07	SSK	4.0	1.3
YER01	-	4.4	1.4	YDR05	TGL2	3.6	1.5	YKL05	MDM	3.9	1.4	YPL15	PET	4.0	1.2
YGR22	TOS2	4.4	1.4	YOL08	DUF1	3.6	1.3	YFR04	BNA6	3.9	1.4	YOR11	---	4.0	1.3
YIR039	YPS6	4.4	1.2	YGR00	SEC9	3.6	1.4	YLR27	-	3.9	1.2	YDL23	GYP	4.0	1.5
YLR20	PNP1	4.4	1.3	YLR29	ECM	3.5	1.4	YPL15	PEP	3.8	1.4	YNR00	CIT1	4.0	1.4
YBR12	MRPL	4.4	1.3	YPR15	PIN3	3.5	1.4	YDR29	MHR	3.8	1.4	YLR30	CDA	4.0	2.2
YKL19	SDS22	4.4	1.5	YBR29	MAL3	3.5	1.5	YKL08	CYT2	3.8	1.5	YPL26	---	4.0	1.3
YOR12	UBP2	4.4	1.2	YCR06	CPR4	3.5	1.5	YOR33	MRS	3.8	1.3	YPL17	---	4.0	1.2
YLR19	UPS1	4.4	1.4	YCL03	GRX1	3.5	1.5	YPL18	RTC6	3.8	1.3	YEL06	SIT1	4.0	2.1
YDR06	AIM7	4.4	1.4	YLR21	COA	3.5	1.8	YAR02	-	3.8	1.4	YPR07	---	4.0	1.8

YPR2(	ARR2	4.4	1.4	YCL04	GLK1	3.5	2.1	YFR04	KEG:	3.8	1.3	YDR0:	RAD	4.0	1.2
YIL017	VID28	4.4	1.5	YDR38	-	3.5	1.5	YJR12	CAF1	3.8	1.5	YML0(	---	4.0	1.3
YGL1(	VPS73	4.4	1.7	YHR12	NDT8	3.5	1.5	YLR30	STT4	3.8	1.2	YMR1:	GID8	4.0	1.4
YGL21	MDM3	4.4	1.4	YGR0:	UGA:	3.5	1.8	YOR18	TUF1	3.8	1.4	YGR08	PDC	4.0	1.6
YNL24	ATG2	4.4	1.5	YPR02	ATH1	3.5	1.5	YPR04	ATG:	3.8	1.3	YBL10	ECM	4.0	1.2
YOR24	DGA1	4.4	1.2	YGR05	FMP4	3.5	1.5	YER01	TIR1	3.8	1.6	YAL06	---	3.9	1.3
YMR3(	YME2	4.4	1.3	YPL18	UIP4	3.5	1.6	YER09	AIM1	3.8	1.4	YBL10	---	3.9	1.3
YOR24	SRL1	4.4	1.2	YDR04	HEM:	3.5	1.5	YNL19	-	3.8	2.2	YDR54	---	3.9	1.3
YLR21	COA4	4.4	1.8	YFL06	SNO:	3.5	1.4	YKL08	SRX1	3.8	1.6	YGL26	---	3.9	1.3
YLR35	BUD8	4.4	1.3	YNL33	SNO2	3.5	1.4	YBR07	ECM8	3.8	2.4	YGR29	---	3.9	1.3
YLR45	-	4.4	1.6	YPL22	FLC1	3.5	1.5	YBR28	-	3.8	1.5	YHL04	---	3.9	1.3
YPL09	MSY1	4.4	1.3	YEL06	SIT1	3.5	2.1	YJR02	BNA:	3.8	2.2	YIL117	PRM	3.9	1.4
YDR27	BSC2	4.3	1.4	YEL05	-	3.5	1.3	YMR27	ZDS1	3.8	1.2	YMR19	SPG	3.9	1.3
YJL089	SIP4	4.3	1.8	YMR01	SPO2	3.5	1.7	YOR1:	AZF1	3.8	1.4	---	---	3.9	1.3
YIL097	FYV10	4.3	1.5	YBR05	AKL1	3.5	1.3	YPR0(	CIT3	3.8	1.3	YHL01	DUR	3.9	1.4
YOL05	ARG1	4.3	2.2	YJR01	ESS1	3.5	1.4	YAL03	SNC:	3.8	1.5	YOR28	---	3.9	1.4
YBL07	-	4.3	1.3	YMR07	PDS5	3.5	1.4	YDR4(	PDR:	3.8	1.4	---	---	3.9	2.3
YGR1(	GTF1	4.3	1.3	YEL01	UBC8	3.5	1.5	YML05	AIM3	3.8	1.3	YDR0(	RCR	3.9	1.3
YDL10	DUN1	4.3	1.2	YHR04	DOG:	3.5	1.6	YFR02	PES4	3.8	3.3	YMR1(	YPK	3.9	1.3
YCR0(	MRPL:	4.3	1.3	YKL08	SRX1	3.4	1.6	YMR1(	YPK2	3.7	1.4	YNL01	PBI2	3.9	1.9
YHR01	DIA4	4.3	1.5	YGL05	PKP2	3.4	1.3	YJL02(	RNR:	3.7	1.4	YDR29	SSD	3.9	1.2
YMR27	PGM3	4.3	1.5	YGR0(	-	3.4	1.8	YPL13	ISU1	3.7	1.2	YKL21	UBA	3.9	1.2
YPR04	MSF1	4.3	1.8	YKL02	-	3.4	1.4	YOR17	MPC5	3.7	1.7	YDR35	---	3.9	1.2
YBR24	-	4.3	1.7	YOR17	MPC5	3.4	1.8	YHL02	AIM1	3.7	2.8	YBL10	---	3.9	1.9
YBR0(	DSF2	4.3	1.4	YOR08	TCB1	3.4	1.4	YCR08	CSM:	3.7	1.3	YDR54	---	3.9	1.9
YBR0(	UGA2	4.3	1.3	YDL01	GRX6	3.4	1.3	YOL15	-	3.7	1.5	YHR21	---	3.9	1.9
YDR21	ADR1	4.3	1.5	YDR21	UPC2	3.4	1.5	YNL32	KRE:	3.7	1.3	YLL06	---	3.9	1.9
YGL08	-	4.3	1.7	YLL02	SPA2	3.4	1.5	YGR17	-	3.7	1.8	YLR46	---	3.9	1.9
YOL0(	MDM1	4.3	1.3	YOR3(	-	3.4	1.5	YGL2(	CHC:	3.7	1.3	YNL33	---	3.9	1.9
YBR15	ICS2	4.3	1.4	YBL03	-	3.4	1.4	YMR0(	MIC1	3.7	1.2	YNL15	NSG	3.9	1.1
YLR16	UPS2	4.3	1.7	YBR24	HIS7	3.4	1.3	YDR06	DOA4	3.7	1.5	YBR05	---	3.8	1.5
YGR2(	TDA10	4.2	2.1	YDR51	EUG:	3.4	1.5	YHR19	AIM1	3.7	1.7	YBR2(	---	3.8	1.2
YOR04	RSB1	4.2	1.8	YPL16	ATG2	3.4	1.4	YGR25	ENO:	3.7	1.8	YIR014	---	3.8	1.8
YKL18	HYM1	4.2	1.2	YML1(	TSL1	3.4	2.2	YKL14	MRP8	3.7	1.3	YOL08	ATG	3.8	1.4
YPL06	VPS28	4.2	1.4	YDL08	-	3.4	1.5	YOR0:	PET1	3.7	1.3	YLR46	PAU	3.8	1.3
YGR25	COQ6	4.2	1.7	YKL03	PTM1	3.4	1.4	YHR14	MRPI	3.7	1.3	YOL16	PAU	3.8	1.3
YLR25	SYM1	4.2	2.1	YHR15	KEL1	3.4	1.5	YJL078	PRY:	3.7	1.7	YER18	---	3.8	1.5
YCR02	SLM5	4.2	1.3	YMR1:	-	3.4	1.3	YMR15	MRP:	3.7	1.4	YCL04	SPS	3.8	1.9
YNL13	-	4.2	2.1	YHR08	KSP1	3.4	1.3	YLR18	ATG2	3.7	1.3	YDL20	MGT	3.8	1.2
YEL05	RML2	4.2	1.3	YKL08	HOT1	3.4	1.4	YLR00	-	3.7	1.5	YNR06	---	3.8	1.6
YDR51	ACN9	4.2	1.5	YIL056	VHR1	3.3	1.3	YML04	CAT2	3.7	1.5	YLR05	---	3.8	1.6
YOL01	CMK2	4.2	1.5	YER12	VFA1	3.3	1.3	YGR0:	-	3.7	1.3	YOR24	DGA	3.8	1.2
YOR15	MRPL:	4.2	1.3	YAR02	UIP3	3.3	1.5	YBL05	-	3.6	1.4	YKL05	MDM	3.8	1.2
YHR0(	TDA3	4.2	1.5	YBR24	-	3.3	1.6	YMR1(	-	3.6	1.5	---	---	3.8	1.6
YBR0(	NTH2	4.2	1.5	YAL06	SEO1	3.3	1.6	YDR43	PPZ2	3.6	1.3	YGR1:	---	3.8	1.3
YJR10	CPA2	4.2	1.3	YMR17	HOT1	3.3	1.3	YNL07	MLF3	3.6	1.4	YAR05	FLO:	3.8	1.3
YIL077	-	4.2	1.4	YPL16	MLH3	3.3	1.5	YOR0(	SGT2	3.6	1.2	YGR24	MGA	3.8	1.8
YML02	YOX1	4.2	1.4	YCR03	BPH1	3.3	1.3	YER03	-	3.6	1.3	YNL20	---	3.8	1.7
YGR29	COS6	4.2	1.4	YPL09	SSU1	3.3	1.5	YOR0:	CRS5	3.6	1.4	YPR11	---	3.8	1.3
YDL06	COX9	4.2	1.3	YJL06	MPM	3.3	1.6	YPL02	SUV3	3.6	1.3	YIR01(	---	3.8	1.3
YCR01	SRD1	4.2	1.5	YLR39	CCW	3.3	1.3	YJL058	BIT6:	3.6	1.3	YFL05	---	3.8	1.2
YPL25	CLN2	4.2	1.5	YBL10	-	3.3	1.4	YKL10	-	3.6	1.4	YMR2:	DFG	3.7	1.2
YBR17	EHT1	4.2	1.5	YCL01	-	3.3	1.4	YDR05	YOS9	3.6	1.2	YNL30	---	3.7	1.5
YIR00:	SGN1	4.2	1.2	YDR03	-	3.3	1.4	YGR26	TNA1	3.6	1.4	YOR1:	---	3.7	1.2
YDR0(	RCR2	4.2	1.2	YDR09	-	3.3	1.4	YPR15	TPO:	3.6	1.3	YML04	CAT	3.7	1.4
YJR04	VPS55	4.2	1.3	YDR21	-	3.3	1.4	YML07	DAK1	3.6	1.4	YKL19	MIA4	3.7	1.1
YAL02	MYO4	4.1	1.2	YDR21	-	3.3	1.4	YPL06	-	3.6	1.4	YJL10:	MEF	3.7	1.3
YKL12	SSH4	4.1	1.5	YDR36	-	3.3	1.4	YHR0(	STP2	3.6	1.2	YDR0:	---	3.7	1.2
YER16	RAD4	4.1	1.5	YFL00	-	3.3	1.4	YKR06	GPT2	3.6	1.5	YOR15	---	3.7	1.5
YLR45	-	4.1	1.2	YGR0:	-	3.3	1.4	YMR1(	SRT1	3.6	1.9	---	---	3.7	1.4
YLR25	NDL1	4.1	1.2	YGR16	-	3.3	1.4	YLR28	GUF:	3.6	1.2	YAR02	UIP3	3.7	1.3
YIR007	-	4.1	1.7	YJR02	-	3.3	1.4	YJR13	TIM8	3.6	1.2	YLR27	PIG1	3.7	1.5
YER06	HOR2	4.1	2.2	YLR22	-	3.3	1.4	YDL16	SFA1	3.6	1.2	YJL16:	---	3.7	1.6
YHR19	MDM3	4.1	1.4	YLR41	-	3.3	1.4	YLR07	-	3.6	1.3	YNR05	BIO3	3.7	1.1
YBR14	YSW1	4.1	1.5	YOR19	-	3.3	1.4	YCR02	SLM5	3.6	1.3	YEL07	DLD:	3.7	1.2
YML1(	CUE4	4.1	1.4	YOR34	-	3.3	1.4	YGL13	ITC1	3.6	1.3	YOL16	---	3.7	2.1
YOR2:	WTM1	4.1	2.0	YPR13	-	3.3	1.4	YIR007	-	3.6	1.7	YDL02	DIA3	3.7	1.5
YBL00	-	4.1	1.4	YML0(	-	3.3	1.5	YPL17	MRPI	3.6	1.3	---	---	3.6	1.3
YBR01	-	4.1	1.4	YNL04	LAP2	3.3	1.3	YLR40	SFP1	3.6	1.2	YNL10	LEU4	3.6	1.2
YDR31	-	4.1	1.4	YER03	CHZ1	3.3	1.4	YCL03	ATG2	3.6	1.3	YOL04	---	3.6	1.4
YER16	-	4.1	1.4	YLR17	-	3.3	1.4	YEL02	-	3.6	1.3	---	---	3.6	1.4

YGR02	-	4.1	1.4	YGR1;	-	3.3	1.5	YER1(	NUP:	3.6	1.3	YHR1ξ	GND	3.6	1.2
YGR1(	-	4.1	1.4	YKL06	BLI1	3.3	1.5	YMR2ξ	MRPI	3.6	1.2	YBR1ς	KTR	3.6	1.2
YHR2:	-	4.1	1.4	YGR04	RME:	3.3	1.6	YBR0(	-	3.6	1.5	YKL14	TGL:	3.6	1.2
YJR02	-	4.1	1.4	YJL16	-	3.2	1.6	YLR08	EMP	3.5	1.8	YLR03	DAN	3.6	1.2
YLR03	-	4.1	1.4	YDL09	PMT5	3.2	1.5	YOL06	CRT1	3.5	1.3	YLL01	BPT:	3.6	1.2
YML04	-	4.1	1.4	YKL15	-	3.2	1.8	YOR2ς	SNF2	3.5	1.2	YDL13	CDC	3.6	1.1
YMR04	-	4.1	1.4	YBR07	SLM4	3.2	1.4	YAR02	PAU;	3.5	1.5	YFL02	GAT	3.6	1.4
YMR0!	-	4.1	1.4	YDR3!	TCM:	3.2	1.4	YOL1(	PKH2	3.5	1.2	YNL05	---	3.6	1.3
YNL28	-	4.1	1.4	YMR1ξ	CTL1	3.2	1.3	YGR1!	CCM	3.5	1.2	YBR11	RAD	3.6	1.3
YOR14	-	4.1	1.4	YOL11	-	3.2	1.4	YPL08	SEC:	3.5	1.3	YER07	---	3.6	1.7
YPR1!	-	4.1	1.4	YMR0(	MVP:	3.2	1.3	YGR1:	SHY:	3.5	1.7	---	---	3.6	1.3
YPR1!	-	4.1	1.4	YOL1(	PKH2	3.2	1.4	YBL00	-	3.5	1.3	YKR04	FMP	3.6	1.3
YPL26	-	4.1	1.8	YLR27	DCS1	3.2	1.6	YBL00	-	3.5	1.3	---	---	3.5	2.3
YPL18	MRN1	4.1	1.3	YNL23	LAP3	3.2	1.4	YDR17	-	3.5	1.3	YKR0(	KTR	3.5	1.2
YAR02	-	4.1	1.4	YDL05	USO:	3.2	1.5	YMR04	-	3.5	1.3	---	---	3.5	1.3
YBR0!	-	4.1	1.6	YBL00	ECM:	3.2	1.3	YMR04	-	3.5	1.3	---	---	3.5	1.3
YPL20	TPK2	4.1	1.5	YHR1!	SPO1	3.2	1.5	YNL28	-	3.5	1.3	YPL15	PRM	3.5	1.3
YHL04	COS8	4.1	1.9	YJR14	-	3.2	1.4	YNL28	-	3.5	1.3	YBL10	---	3.5	1.2
YIL087	AIM19	4.1	1.7	YNL29	MON:	3.2	1.2	YMR1ξ	RGM	3.5	1.4	YER1:	---	3.5	1.2
YHL0(	MRP4	4.1	1.5	YLR21	FRE1	3.2	1.3	YNL18	NPR:	3.5	1.3	YOR1ς	---	3.5	1.2
YLR25	HAP1	4.1	1.3	YKL10	GFA1	3.2	1.3	YBL08	PET1	3.5	1.4	---	---	3.5	1.3
YDR3(	-	4.1	1.4	YNL05	ARP5	3.2	1.3	YGL01	PDR:	3.5	1.3	YGR2:	---	3.5	1.3
YOR0!	VHS3	4.1	1.3	YML0(	-	3.2	1.3	YGL0!	PKP2	3.5	1.3	YDR0!	TGL:	3.5	1.3
YKL14	TGL1	4.1	1.3	YOR3ξ	RDR:	3.2	1.3	YHR1ξ	-	3.5	1.5	---	---	3.5	1.3
YIL042	PKP1	4.1	1.4	YOR2:	MCT:	3.2	1.6	YNL24	CWC	3.5	1.2	YBR1:	TPS:	3.5	1.5
YHR0:	RRF1	4.1	1.3	YPL17	DAP1	3.2	1.6	YLR27	DCS:	3.5	1.6	---	---	3.5	1.3
YGL0ξ	MMS2	4.0	1.4	YLR20	ENT2	3.2	1.4	YOR3:	PDR:	3.5	1.3	YHR0!	RSC	3.5	1.3
YML0!	GAL8(	4.0	1.2	YBR07	HSP2	3.2	3.8	YKR0(	MRPI	3.5	1.4	YHR0!	RSC	3.5	1.3
YGR2:	-	4.0	1.4	YBR21	SDS2	3.2	1.9	YDR14	DOP:	3.5	1.3	YGR2:	KEL:	3.5	1.2
YKL03	UGP1	4.0	1.4	YDL06	IDP1	3.2	1.3	YBR2(	AIM5	3.5	1.3	YMR0!	AAC	3.5	1.3
YIL037	PRM2	4.0	1.8	YOR3:	MYO:	3.2	1.3	YBR01	KAP1	3.5	1.1	YML0:	TSA:	3.5	1.1
YGL04	RIM8	4.0	1.2	YFL06	COS4	3.2	1.3	YGR1:	MEP:	3.5	1.4	YBR21	SDS	3.5	1.8
YDR3ξ	-	4.0	1.7	YGR2ς	COS(	3.2	1.3	YJR10	RSM:	3.5	1.3	YPL12	RNY	3.4	1.7
YKR04	FMP4(	4.0	1.5	YNL33	COS:	3.2	1.3	YKL15	RSM:	3.5	1.2	YAL02	FRT:	3.4	1.4
YPR0(	AIM45	4.0	1.3	YNR0(	DSE4	3.1	1.4	YOL1!	HPF1	3.5	1.8	YHR1(	YAP	3.4	1.2
YHR1(	GRE3	4.0	1.7	YML07	HMG	3.1	1.3	YER0(	ARG!	3.5	1.3	YDL02	---	3.4	1.3
YGR2;	SCW4	4.0	1.2	YOL1(	-	3.1	1.8	YLR33	VRP:	3.5	1.4	YLR32	---	3.4	1.2
YKL12	DGR2	4.0	1.6	YNL30	BXI1	3.1	1.5	YDR1:	KIN1	3.5	1.4	YNL15	---	3.4	1.2
YER07	ICP55	4.0	1.3	YJL03	IRC1ξ	3.1	1.7	YMR0:	-	3.5	1.7	YKR0(	GPT	3.4	1.4
YML0!	AIM32	4.0	1.3	YMR0ξ	ADH:	3.1	1.3	YLR39	ATP1	3.4	1.6	YLR09	SUL:	3.4	1.4
YKR0(	MRPL:	4.0	1.4	YBL00	SLA1	3.1	1.3	YKL21	OXP:	3.4	1.2	YBR2ξ	---	3.4	1.3
YAR02	UIP3	4.0	1.4	YJR15	DAL5	3.1	1.6	YMR1ξ	GCV:	3.4	1.8	YOL11	SKM	3.4	1.5
YOR1!	ISN1	4.0	1.4	YBL04	EDE1	3.1	1.3	YGR0:	MSP:	3.4	1.3	YIR02	DAL:	3.4	1.2
YNL08	SWS2	4.0	1.4	YJL0!	TDH1	3.1	2.0	YMR0ς	ATP2	3.4	1.3	YOR3ξ	---	3.4	1.5
YLR16	PUS5	4.0	1.5	YLR34	-	3.1	1.4	YDL23	GUD:	3.4	1.5	YGR1!	GTO	3.4	1.2
YPR07	OPY2	4.0	1.4	YAL03	FUN1	3.1	1.3	YPL07	UBP:	3.4	1.3	---	---	3.4	1.2
YPL20	PGC1	4.0	1.2	YPL12	RNY1	3.1	1.8	YKL19	ACP:	3.4	1.4	YDL22	FMP	3.4	2.2
YPL14	MKK2	4.0	1.3	YKL19	PTK1	3.1	1.4	YDR01	GCV:	3.4	1.8	YDL02	RPN	3.4	1.2
YML1:	ERO1	4.0	1.2	YDR1:	ARO:	3.1	1.3	YMR1:	-	3.4	1.2	---	---	3.4	1.5
YAR0(	-	4.0	1.5	YOR2:	WTM	3.1	1.9	YAL06	SEO:	3.4	1.5	YMR0ξ	YTA:	3.4	1.2
YBL00	-	4.0	1.5	YJR00	APL1	3.1	1.3	YGR14	-	3.4	1.6	YMR1ς	---	3.4	1.1
YBR01	-	4.0	1.5	YMR1ς	-	3.1	1.5	YLR20	QRI5	3.4	1.3	YER04	ACA	3.4	1.3
YER1:	-	4.0	1.5	YNL07	MLF3	3.1	1.4	YBR1(	AIM3	3.4	1.5	YDR4(	MFA	###	0.6
YGR0:	-	4.0	1.5	YIR0(	MSL1	3.1	1.2	YGR0ξ	PDC(	3.4	1.5	YGL0:	SCW	###	0.6
YGR1(	-	4.0	1.5	YKL15	APE2	3.1	1.4	YEL04	-	3.4	1.5	YLR06	---	-8.8	0.7
YJR02	-	4.0	1.5	YGL0:	-	3.1	1.3	YDR0:	MIC1	3.4	1.6	YMR2ξ	RIT1	-8.5	0.8
YJR02	-	4.0	1.5	YPR1:	CTR1	3.1	1.2	YKL14	SDH:	3.4	1.5	YPL13	RDS	-8.4	0.8
YLR1!	-	4.0	1.5	YOL0!	THI2(	3.1	1.3	YNL31	ZIM1	3.4	1.3	YDR4;	SNM	-7.9	0.7
YML0:	-	4.0	1.5	YBR0!	REG:	3.0	1.4	YIL134	-	3.4	1.4	YNL14	AAH	-7.9	0.7
YMR04	-	4.0	1.5	YNL15	NSG:	3.0	1.2	YJL0!	-	3.4	1.2	YML0ς	---	-7.6	0.8
YNL28	-	4.0	1.5	YGL07	RCS1	3.0	1.4	YBR2!	-	3.4	1.3	YLR45	SST:	-7.6	0.5
YOL1(	-	4.0	1.5	YER02	GPA2	3.0	1.3	YKL07	SMY:	3.4	1.2	YHR1(	THP:	-7.5	0.7
YOR14	-	4.0	1.5	YMR1:	-	3.0	1.4	YOR2:	WTM	3.4	1.9	YGL2!	ADH	-7.3	0.8
YPL25	-	4.0	1.5	YNL27	SEC2	3.0	1.3	YGR1:	-	3.4	1.3	YOR1(	RAS	-7.2	0.7
YIL09ς	SGA1	4.0	1.3	YDR51	SLF1	3.0	1.2	YEL05	RML:	3.4	1.3	YDR42	---	-7.2	0.8
YDL11	-	4.0	1.6	YHL03	VMR:	3.0	1.7	YHR1(	DNA:	3.4	1.2	YDL20	TRM	-7.0	0.8
YLR28	-	4.0	2.1	YBR02	SCO:	3.0	1.3	YDL03	GPR:	3.4	1.1	YBR0:	---	-7.0	0.7
YNL18	MRPL:	4.0	1.3	YLR13	USB1	3.0	1.4	YPR17	-	3.3	1.7	YOR2:	STE	-6.9	0.8
YDR2ς	SSD1	4.0	1.3	YAL04	GCV:	3.0	1.5	YOR3!	HAP5	3.3	1.3	YLR39	AFG	-6.8	0.7
YMR2(	TPS3	4.0	1.3	YIR0:	DAL7	3.0	1.4	YJR08	-	3.3	1.3	YDR22	---	-6.8	0.8
YOR0:	ROD1	4.0	1.4	YLR46	PAU	3.0	1.3	YMR1!	AIM3	3.3	1.4	YBR22	---	-6.8	0.7

YOR12	CAT5	4.0	1.6	YOL16	PAU4	3.0	1.3	YJL04	MHP1	3.3	1.4	YER12	DSE	-6.7	0.7
YDR47	PEX29	4.0	1.3	YHR19	AIM4	3.0	1.3	YIL070	MAM	3.3	1.4	YJL21	OPT	-6.7	0.8
YBR11	MUD1	4.0	1.2	YAL03	CYC3	3.0	1.4	YBL00	HIR1	3.3	1.2	YHL00	YAP	-6.7	0.8
YHR13	-	4.0	1.3	YLR12	-	3.0	1.4	YNL29	MON	3.3	1.2	YNL21	RAP	-6.7	0.8
YER03	ZRG8	4.0	1.9	YGR12	SYF2	3.0	1.4	YMR29	PRC1	3.3	1.4	YER15	---	-6.6	0.7
YAL03	CYC3	4.0	1.3	YLL02	FRA1	3.0	1.4	YML11	COQ	3.3	1.3	YER17	GRX	-6.6	0.7
YDL01	OSH2	3.9	1.4	YDR40	ADE8	3.0	1.3	YGR12	-	3.3	1.4	YMR12	SAS	-6.6	0.8
YCL03	GID7	3.9	1.4	YBR11	LYS2	3.0	1.3	YML10	NUP1	3.3	1.2	YIL015	BAR	-6.3	0.6
YOR12	GCY1	3.9	1.3	YER14	COX1	3.0	1.5	YGR17	MSM	3.3	1.2	YPL18	---	-6.3	0.8
YKL17	STE3	3.9	1.7	YKL04	DCW	3.0	1.3	YMR13	GAT2	3.3	1.6	YHR12	FUR	-6.3	0.8
YBL06	KIP1	3.9	1.2	YBL05	PIN4	3.0	1.5	YMR27	PGM	3.3	1.3	YHR05	MED	-6.2	0.8
YJR11	JHD2	3.9	1.7	YOR02	-	3.0	1.3	YPR04	MSF1	3.3	1.6	YGL15	CDC	-6.0	0.8
YIL056	VHR1	3.9	1.2	YBR14	RTC2	3.0	2.2	YNL16	CBK1	3.3	1.2	YHR04	FSH	-6.0	0.7
YKR01	TOF2	3.9	1.3	YNL22	ADE1	3.0	1.2	YLR12	YPS3	3.3	1.5	YMR12	---	-6.0	0.8
YBL00	-	3.9	1.4	YNL00	IDP3	3.0	1.4	YPR04	PUF2	3.3	1.3	YLR14	---	-6.0	0.8
YDR26	-	3.9	1.4	YCR02	NPP1	3.0	1.3	YHR05	FYV4	3.3	1.3	YBR26	TSC	-6.0	0.7
YDR36	-	3.9	1.4	YPL15	-	3.0	1.3	YOR00	TIR4	3.3	1.3	YMR13	RRB	-5.9	0.8
YGR02	-	3.9	1.4	YGR01	NMA1	3.0	1.2	YDR01	-	3.3	1.7	YGL10	---	-5.9	0.7
YJR02	-	3.9	1.4	YBR14	IRA1	2.9	1.3	YPL09	MGR	3.3	1.3	YKL11	ABF1	-5.8	0.8
YMR04	-	3.9	1.4	YBR10	VID24	2.9	1.3	YLR42	TUS1	3.3	1.3	YJL15	FAR	-5.8	0.6
YNL28	-	3.9	1.4	YOR30	MCH5	2.9	1.5	YBR14	RTC2	3.3	2.4	YGR00	---	-5.8	0.8
YDR25	PAM1	3.9	1.4	YPR16	GPH1	2.9	1.9	YAL04	GCV3	3.3	1.4	YGR03	---	-5.8	0.7
YMR21	-	3.9	1.6	YMR01	-	2.9	1.6	YKL21	DOA1	3.3	1.3	YJR07	LIA1	-5.7	0.7
YMR05	FAR3	3.9	1.3	YPR11	-	2.9	1.3	YPL12	RNY1	3.3	1.8	YDL03	PUS	-5.7	0.8
YJR07	MIR1	3.9	1.3	YGR27	YTA7	2.9	1.2	YJL17	CPS1	3.3	1.4	YDR38	SAC	-5.7	0.8
YBR08	NHP6E	3.9	1.2	YFR03	-	2.9	1.2	YDR38	-	3.3	1.5	YGR26	TNA	-5.6	0.8
YBR09	NHP6E	3.9	1.2	YBR08	NHP6	2.9	1.2	YLR05	-	3.3	1.4	YNL07	NIS1	-5.6	0.6
YCR07	SOL2	3.9	1.4	YBR09	NHP6	2.9	1.2	YLR24	VPS3	3.3	1.3	YDR09	RLI1	-5.6	0.8
YGR24	FMP43	3.9	1.7	YDR18	-	2.9	1.3	YDR35	TCM1	3.3	1.3	YOR35	---	-5.6	0.8
YOR35	MSC6	3.9	1.4	YNL14	ALF1	2.9	1.3	YGR20	CIR1	3.3	1.3	---	---	-5.6	0.7
YGR25	RAD2	3.9	1.3	YCL01	SGF2	2.9	1.4	YLR44	-	3.3	1.8	YPL25	HFI1	-5.6	0.8
YOR18	IES4	3.9	1.3	YLR02	ADE1	2.9	1.2	YJL03	KAR2	3.2	1.2	YNL02	---	-5.6	0.8
YMR19	SPG5	3.9	1.3	YNL27	BNI1	2.9	1.2	YHR03	-	3.2	1.4	YLR32	NMA	-5.5	0.8
YGR07	ROM1	3.9	1.4	YOR38	FRE5	2.9	3.0	YJL12	PBS2	3.2	1.2	YIL104	SHQ	-5.5	0.8
YER05	HMF1	3.9	1.2	YBR05	-	2.9	1.5	YBR21	SDS2	3.2	2.0	YBR06	ORC	-5.5	0.8
YDR51	EMI2	3.9	1.8	YBR08	NHP6	2.9	1.3	YDR51	GRX2	3.2	1.2	YDR24	SNU	-5.5	0.7
YLR39	ART10	3.9	1.3	YLR43	CAR2	2.9	1.4	YLR35	-	3.2	1.3	YGL20	MCM	-5.5	0.8
YLR35	ATG33	3.9	1.9	YJL14	YAK1	2.9	1.3	YHR09	TRA1	3.2	1.3	YOR26	DSE	-5.5	0.7
YML11	-	3.9	1.2	YHR02	MYO1	2.9	1.5	YDR47	JIP4	3.2	1.5	YOL12	---	-5.4	0.8
YBL09	SCS22	3.9	1.5	YDR24	-	2.9	1.3	YGL22	MTC3	3.2	1.3	YOR31	SFG	-5.4	0.7
YBL04	COR1	3.9	1.5	YBR03	EDS1	2.9	2.7	YLR09	MIM2	3.2	1.3	YDL15	CLB3	-5.4	0.8
YHR11	-	3.9	1.4	YOL08	ATG1	2.9	1.4	YLR13	RKM5	3.2	1.3	YHL01	PRS	-5.4	0.8
YPL25	ICY2	3.8	1.6	YIR00	PAN1	2.9	1.3	YGR17	-	3.2	1.9	YNL11	DBP	-5.4	0.7
YCR07	ERS1	3.8	1.5	YDL09	UBX3	2.9	1.4	YOR20	MRM	3.2	1.2	YKR06	UTP	-5.3	0.7
YJL20	NCE10	3.8	1.5	YLR03	DAN2	2.9	1.2	YBR26	FMP2	3.2	1.3	YOR23	DFR	-5.3	0.8
YOR38	-	3.8	1.5	YGR00	STF2	2.9	1.8	YJR08	EAF6	3.2	1.2	YBR19	TAF5	-5.3	0.8
YPL27	-	3.8	1.5	YOR10	PIN2	2.9	1.3	YPL16	REV3	3.2	1.3	YHR07	TRM	-5.3	0.7
YMR01	-	3.8	1.7	YGR14	VPS6	2.9	1.2	YLR41	VIP1	3.2	1.2	YPL03	---	-5.2	0.8
YNL22	ATG4	3.8	1.4	YMR08	VBA1	2.9	1.2	YBL03	STU1	3.2	1.2	YHR17	NMD	-5.2	0.8
YBR26	AIM5	3.8	1.4	YLR03	-	2.9	1.3	YNL19	DUG3	3.2	1.3	YGR26	BUD	-5.2	0.8
YLR05	OSW2	3.8	1.5	YLL01	YEH1	2.9	1.5	YNR04	FPK1	3.2	1.4	YMR23	---	-5.2	0.6
YNR03	SSK2	3.8	1.2	YPR19	SGE1	2.9	1.3	YMR31	ADH6	3.2	1.2	YHR03	---	-5.2	0.7
YEL04	-	3.8	1.5	YIL101	XBP1	2.9	1.9	YEL01	MMS	3.2	1.7	YJR06	RPA	-5.2	0.8
YHR01	ARG4	3.8	1.4	YMR02	CSI1	2.9	1.3	YER07	RNR1	3.2	1.3	YMR02	MAC	-5.2	0.8
YLR21	MSC3	3.8	1.3	YOR12	IAH1	2.9	1.3	YHL02	RIM1	3.2	1.2	YNL06	SUN	-5.1	0.8
YGR25	GND2	3.8	1.5	YLR37	STP3	2.9	1.2	YOR36	PDE2	3.2	1.5	YIR008	PRI1	-5.1	0.7
YOR38	RDR1	3.8	1.2	YJR07	MOG	2.9	1.3	YHR04	YHK8	3.2	1.4	YGL16	SUT	-5.1	0.8
YBL04	EDE1	3.8	1.4	YDL11	ATG2	2.9	1.4	YGL02	SCW	3.2	1.3	YBL06	PRS	-5.1	0.8
YBR08	UBC4	3.8	1.2	YBR22	MCX1	2.9	1.3	YML05	NTE1	3.2	1.3	YPL19	---	-5.1	0.8
YIR037	HYR1	3.8	1.5	YIR003	AIM2	2.9	1.3	YMR31	DIA1	3.2	1.3	YNL32	EGT	-5.1	0.8
YLR40	BLS1	3.8	1.3	YKR10	SIR1	2.9	1.3	YLR02	IRC2	3.2	1.3	YMR31	---	-5.1	0.7
YLR20	COQ9	3.8	1.5	YJL005	CYR1	2.9	1.3	YOR02	STI1	3.2	1.3	YBR04	REB	-5.1	0.8
YBR08	NHP6E	3.8	1.3	YMR31	-	2.9	1.6	YLR38	NAM2	3.2	1.2	YOR35	VTS1	-5.0	0.6
YLR00	-	3.8	1.5	YOR22	RCN2	2.8	1.4	YPL20	PGC1	3.2	1.2	YPL13	UME	-5.0	0.8
YLR03	COX12	3.8	1.4	YMR01	ERG5	2.8	1.3	YIR002	MPH1	3.2	1.3	YLR40	SFP1	-5.0	0.8
YMR02	MSS1	3.8	1.4	YEL04	YEF1	2.8	1.5	YDR31	HIM1	3.2	1.4	YKL01	SWD	-5.0	0.8
YDL24	AAD4	3.8	1.5	YIR018	-	2.8	1.4	YOR15	MRPI	3.2	1.3	YDR39	HPT	-5.0	0.7
YNL07	TPM1	3.8	1.2	YLR26	LCB5	2.8	1.3	YKR06	CCP1	3.2	1.4	YJL19	PHO	-5.0	0.8
YIL040	APQ12	3.8	1.1	YJR14	BAT2	2.8	1.8	YGR07	PEX8	3.2	1.2	YLR28	CTS	-5.0	0.7
YBR12	CBP6	3.7	1.3	YJR04	ANB1	2.8	2.4	YBR05	-	3.2	1.4	YDL22	HO	-5.0	0.8
YPR14	NCE10	3.7	1.2	YJL21	-	2.8	1.4	YIL073	SPO2	3.1	1.8	YPL10	ELP4	-5.0	0.7

YLR15	PCD1	3.7	1.4	YIL114	POR2	2.8	1.4	YBR18	MBA1	3.1	1.4	YPL08	ELP3	-5.0	0.8
YJR03	PET19	3.7	1.5	YBL01	ACH1	2.8	1.4	YEL02	TIM9	3.1	1.4	YGL09	LSG	-5.0	0.8
YOR37	NDD1	3.7	1.4	YIL087	AIM1	2.8	1.5	YML03	AMD	3.1	1.2	YGR04	BUD	-5.0	0.7
YOR00	TIR4	3.7	1.5	YDR16	STB3	2.8	1.3	YIL047	SYG1	3.1	1.2	YNL06	GCD	-5.0	0.8
YGR13	PEX4	3.7	1.5	YDR07	SNF1	2.8	1.3	YIL047	SYG1	3.1	1.2	YOR12	---	-5.0	0.8
YOR10	CRC1	3.7	2.4	YBR25	SRB6	2.8	1.2	YIL066	RNR3	3.1	1.6	YOL02	DIS3	-4.9	0.9
YDL19	GGC1	3.7	1.2	YDR48	CWC	2.8	1.3	YEL04	-	3.1	1.2	YNL31	---	-4.9	0.8
YER05	PET11	3.7	1.6	YHR17	STB5	2.8	1.4	YHR03	RRM	3.1	1.3	YBR27	---	-4.9	0.7
YGR02	-	3.7	1.3	YNR00	LRO1	2.8	1.3	YGL09	SPC1	3.1	1.2	YFL02	STE	-4.8	0.7
YBL05	CMC2	3.7	1.5	YHR00	TDA3	2.8	1.3	YLL02	POM	3.1	1.4	YHR06	RRP	-4.8	0.8
YOR34	PYK2	3.7	1.3	YFR01	-	2.8	1.5	YDR10	STE5	3.1	1.2	YDL11	RRP	-4.8	0.8
YNL17	MRPL	3.7	1.2	YDL13	ARF2	2.8	1.2	YGL00	PMC	3.1	1.2	YHR18	IKI1	-4.8	0.8
YEL04	PAU2	3.7	1.3	YML08	-	2.8	1.4	YBR16	CDC	3.1	1.2	YGR12	UTP	-4.8	0.8
YLR42	ATG17	3.7	1.3	YCL03	GID7	2.8	1.3	YPR00	ICL2	3.1	1.4	YNL31	PHA	-4.8	0.8
YKR09	-	3.7	1.3	YML05	CMP2	2.8	1.3	YBL09	BNA4	3.1	1.3	YDL24	LRG	-4.8	0.8
YOR28	RDL1	3.7	1.4	YGL00	-	2.8	1.8	YNL18	SWT	3.1	1.3	YLR36	---	-4.8	0.8
YBR11	SUS1	3.7	1.2	YNL27	CAF1	2.8	1.4	YPR05	SEC8	3.1	1.2	YLR13	ZRT	-4.8	0.8
YJR12	CAF17	3.7	1.4	YBR06	-	2.8	1.3	YFL01	WWM	3.1	1.3	YOR03	CYC	-4.8	0.8
YCR00	-	3.7	1.4	YPL17	NIP10	2.8	1.4	YLR09	ICT1	3.1	1.3	YMR28	HSH	-4.8	0.8
YLR38	NAM2	3.7	1.3	YJL07	NET1	2.8	1.3	YAL01	NTG1	3.1	1.3	YAL02	ATS	-4.7	0.8
YGL20	SIP2	3.7	1.4	YKR08	HBS1	2.8	1.2	YHR11	DMA	3.1	1.3	YKL04	ELM	-4.7	0.8
YPR04	TIP41	3.6	1.2	YJL07	-	2.8	1.4	YLR14	ACF2	3.1	1.3	YLR00	NOC	-4.7	0.8
YGL25	RMR1	3.6	1.4	YAL05	ACS1	2.8	1.4	YGL18	-	3.1	1.2	YAR03	PRM	-4.7	0.7
YPL11	MRP51	3.6	1.2	YEL04	-	2.8	1.5	YBR14	ARA1	3.1	1.4	YBR06	TRM	-4.7	0.7
YNL23	LAP3	3.6	1.3	YPR15	KRE6	2.8	1.2	YKR09	UBP1	3.1	1.2	YCR05	---	-4.7	0.8
YGL01	-	3.6	1.2	YKL07	STB6	2.8	1.3	YIL036	CST6	3.1	1.3	YML08	DUS	-4.7	0.7
YDL08	-	3.6	1.3	YKR09	PTR2	2.8	1.6	YGL18	STR3	3.1	1.5	YBR03	HMT	-4.7	0.8
YKL10	-	3.6	1.4	YJR00	-	2.8	1.8	YNL30	MRP	3.1	1.2	YMR24	ZRC	-4.7	0.8
YHR14	MRPL	3.6	1.3	YFR02	PTR3	2.8	1.3	YFR02	ATG1	3.1	1.4	YJL17	SWI	-4.7	0.8
YML07	TCB3	3.6	1.2	YMR12	-	2.8	1.3	YGL06	MNP	3.1	1.3	YPL12	TAF	-4.7	0.9
YNL00	MRP7	3.6	1.2	YJL07	ICS3	2.7	1.6	YOR38	-	3.1	1.3	YOR21	SAS	-4.7	0.8
YOR08	TGL5	3.6	1.4	YGR17	-	2.7	1.6	YLR31	MRP	3.1	1.3	YLR29	GCD	-4.7	0.8
YHL04	-	3.6	1.7	YLR15	PCD1	2.7	1.4	YBR14	IRA1	3.1	1.2	YER12	NSA	-4.7	0.8
YML05	NTE1	3.6	1.4	YDR02	REG1	2.7	1.3	YAR07	PHO	###	0.1	YHR00	---	-4.6	0.8
YMR12	ADE17	3.6	1.4	YOL02	MIM1	2.7	1.3	YHR21	PHO	###	0.1	YBR14	---	-4.6	0.7
YHR08	KSP1	3.6	1.3	YDR17	CSN9	2.7	1.4	YDR46	MFA	###	0.5	YDR46	RMT	-4.6	0.8
YAR02	-	3.6	1.3	YOR04	CUE5	2.7	1.2	YOR37	GDH	###	0.4	YIL003	CFD	-4.6	0.8
YAL01	-	3.6	1.5	YLL06	PAU6	2.7	1.3	YKL12	OAC	###	0.3	YHR10	---	-4.6	0.8
YOR15	-	3.6	1.5	YNR07	PAU6	2.7	1.3	YIL121	QDR	###	0.2	YDR16	---	-4.6	0.8
YGR02	MSP1	3.6	1.4	YNL07	APJ1	2.7	1.2	YIL176	PAU1	###	0.2	YFL02	GYP	-4.6	0.8
YGR11	SHY1	3.6	1.6	YIL108	-	2.7	1.2	YJL22	PAU1	###	0.2	YLR21	CDC	-4.6	0.8
YDR21	MFB1	3.6	1.2	YGR16	CLC1	2.7	1.2	YLR46	PAU4	###	0.3	YKL11	PRR	-4.6	0.8
YHL02	RIM4	3.6	1.5	YGL21	MDM	2.7	1.3	YOL16	PAU4	###	0.3	YDR44	DOT	-4.6	0.8
YJL05	-	3.6	1.2	YKR02	NTR2	2.7	1.4	YLL06	PAU6	###	0.3	YLR45	---	-4.6	0.9
YCR03	SYP1	3.6	1.5	YBR13	-	2.7	1.5	YNR07	PAU6	###	0.3	YNR04	TRM	-4.6	0.7
YKL13	SHE2	3.6	1.2	YLR43	ATG2	2.7	1.4	YOR21	STE4	###	0.6	YIR02	YVH	-4.6	0.8
YPL03	PHO85	3.5	1.3	YBR14	ARA1	2.7	1.4	YNR07	AIF1	###	0.3	YHR06	RPP	-4.6	0.8
YNR01	-	3.5	1.5	YOR38	FRE3	2.7	1.6	YLR45	SST2	###	0.4	YOR16	---	-4.6	0.8
YJR08	STE18	3.5	1.3	YNL08	TOP2	2.7	1.3	YGL10	-	###	0.6	YAR00	SEN	-4.6	0.8
YBR26	MRPL	3.5	1.3	YMR20	RAD1	2.7	1.3	YOR23	DFR1	###	0.6	YNR04	---	-4.5	0.7
YOR28	MPD1	3.5	1.3	YMR30	ADH2	2.7	1.3	YFR05	IRC7	###	0.3	YNL18	IPI3	-4.5	0.7
YNR03	RSM19	3.5	1.5	YDR03	PST2	2.7	1.4	YHR21	IMD2	###	0.3	YJR06	HAM	-4.5	0.8
YIL125	KGD1	3.5	1.4	YER08	PTC2	2.7	1.3	YNL14	ALF1	###	0.5	YFL04	RGD	-4.5	0.8
YBR04	FAT1	3.5	1.3	YGL02	ALK1	2.7	1.3	YDL24	COS	-9.9	0.1	YGL03	AGA	-4.5	0.6
YOR09	-	3.5	1.3	YGR09	ASK1	2.7	1.2	YJR16	COS	-9.9	0.1	YNL16	PSD	-4.5	0.8
YBR12	TPS1	3.5	1.6	YJR00	SAG1	2.7	1.4	YAL06	PAU8	-9.6	0.3	YOL01	RCL	-4.5	0.7
YAL03	GIP4	3.5	1.3	YML01	SEL1	2.7	1.3	YOR22	ISU2	-9.6	0.6	YGL16	SUA	-4.5	0.8
YPL23	ENV7	3.5	1.2	YPL01	SWI1	2.7	1.3	YHL04	COS8	-9.6	0.1	YOL08	REX	-4.5	0.7
YDR12	KIN1	3.5	1.4	YDL03	PRM	2.7	1.7	YDR54	-	-9.5	0.2	YOR27	YTM	-4.5	0.8
YDR50	GMC1	3.5	1.3	YKL22	-	2.7	1.3	YBR24	GPX2	-9.4	0.3	YKR04	---	-4.5	0.7
YKL05	MPE1	3.5	1.2	YMR20	HFA1	2.7	1.3	YAR07	IMD1	-9.2	0.2	YNR04	AGA	-4.5	0.4
YPL24	-	3.5	1.2	YML00	YAP1	2.7	1.3	YHR21	IMD2	-9.2	0.2	YDL21	---	-4.5	0.8
YJL12	TRK1	3.5	1.2	YDR30	-	2.7	1.3	YGL00	LEU1	-9.0	0.4	YCL03	GFD	-4.5	0.7
YGR04	-	3.5	1.3	YJL05	-	2.6	1.2	YMR26	RSN1	-8.9	0.6	YCL02	FUS	-4.5	0.4
YPL18	RTC6	3.5	1.2	YKR03	SPO1	2.6	1.3	YDL19	GGC	-8.9	0.7	YML02	APT	-4.5	0.8
YDR06	DOA4	3.5	1.4	YOR12	RGA1	2.6	1.3	YIR02	YVH1	-8.9	0.7	YML02	USA	-4.5	0.7
YNR04	-	3.5	1.5	YLL02	POM	2.6	1.3	YMR01	HXT2	-8.9	0.2	YHR14	DSE	-4.5	0.6
YKL12	MYO3	3.5	1.3	YLR02	SNF7	2.6	1.3	YNL12	NRK1	-8.6	0.6	YKL19	DPH	-4.5	0.8
YJR09	JSN1	3.5	1.3	YBR30	COS2	2.6	1.3	YER17	GRX4	-8.5	0.6	YOR00	---	-4.5	0.8
YDL17	AIR2	3.5	1.3	YML13	COS3	2.6	1.3	YHR00	GPA1	-8.5	0.6	YLR03	RAD	-4.4	0.8
YLR38	STE23	3.5	1.2	YJR11	TDA4	2.6	1.4	YJR00	SUI2	-8.1	0.7	YLR18	EMG	-4.4	0.8

YKL15	APE2	3.5	1.3	YOR0:	PEP1	2.6	1.4	YKR0(	MEH	-8.0	0.6	YDL01	NOP	-4.4	0.8
YIL029	-	3.5	1.5	YML01	PSP2	2.6	1.3	YPL24	CIN2	-8.0	0.5	YLR31	TAD	-4.4	0.8
YPL01	-	3.5	1.8	YLR17	APS1	2.6	1.4	YPL19	-	-7.9	0.7	YPR16	ORC	-4.4	0.8
YOL03	SIL1	3.5	1.3	YDL11	TMA1	2.6	1.4	YPL17	CBC2	-7.8	0.7	YNL30	MCK	-4.4	0.8
YHR08	-	3.5	1.3	YKR06	KTR2	2.6	1.2	YDR38	ATO3	-7.8	0.6	YBR08	TEC	-4.4	0.6
YOR22	MCT1	3.5	1.5	YAR02	-	2.6	1.3	YER18	-	-7.8	0.3	YPR01	TIF6	-4.4	0.8
YJL09	GWT1	3.5	1.2	YLR45	FMP2	2.6	1.4	YOR10	VAM3	-7.7	0.7	YBR25	DUT	-4.4	0.8
YPR02	ATP20	3.5	1.2	YCR06	SED4	2.6	1.3	YHR20	BAT1	-7.7	0.5	YCR08	TUP	-4.4	0.9
YGR16	RTS3	3.4	1.5	YLR33	VRP1	2.6	1.4	YBR30	COS	-7.7	0.4	YBR24	ENP	-4.4	0.8
YDR30	GIC2	3.4	1.3	YJL15	HSP1	2.6	1.3	YML13	COS	-7.7	0.4	YNL20	PSY	-4.4	0.7
YNL13	FYV6	3.4	1.5	YNL16	YGP1	2.6	1.3	YDR11	TMA	-7.6	0.6	YOL06	RIB2	-4.4	0.8
YDR10	TVP15	3.4	1.4	YPR02	YME1	2.6	1.2	YDR26	CIA1	-7.6	0.6	YEL05	HAT	-4.3	0.8
YPR12	-	3.4	1.4	YJL09	CHS6	2.6	1.2	YFR03	PHO4	-7.5	0.6	YPL16	---	-4.3	0.8
YDL21	RRI1	3.4	1.3	YMR1(	PGM2	2.6	1.8	YPR12	ANT1	-7.5	0.6	YNR01	URK	-4.3	0.8
YBR25	MRPS	3.4	1.3	YMR0:	-	2.6	1.2	YPL12	TAF1	-7.5	0.7	YJR01	---	-4.3	0.8
YLR01	TEN1	3.4	1.5	YLR08	GAA1	2.6	1.3	YDR53	PAD1	-7.5	0.4	YCR03	RRP	-4.3	0.8
YIL024	-	3.4	1.2	YOR1!	-	2.6	1.4	YDR16	-	-7.4	0.7	YDR02	---	-4.3	0.7
YFL01	MDJ1	3.4	1.2	YER01	BUD2	2.6	1.3	YDR26	SWM	-7.3	0.7	YAR00	NUP	-4.3	0.8
YER09	RAD51	3.4	1.3	YDL12	SNA4	2.6	1.2	YGR00	TFG2	-7.2	0.7	YOR07	---	-4.3	0.9
YJL02	BBC1	3.4	1.4	YLR17	RFX1	2.6	1.3	YLR22	CDC4	-7.2	0.8	YOL05	SPE	-4.3	0.8
YLR13	SLX4	3.4	1.1	YHL04	COS8	2.6	1.6	YOR27	FSF1	-7.1	0.7	YCR06	HCM	-4.3	0.8
YFL04	-	3.4	1.2	YIL138	TPM2	2.5	1.2	YLR18	SAM	-7.1	0.6	YNL05	COX	-4.3	0.8
YDL13	ARF2	3.4	1.1	YDR5(	GMC	2.5	1.3	YDR27	RNH	-7.1	0.7	YML08	---	-4.3	0.8
YDR51	SLF1	3.4	1.3	YGR28	BGL2	2.5	1.2	YER18	-	-7.0	0.3	YGL21	NCS	-4.3	0.7
YNL12	-	3.4	1.4	YAR05	FLO1	2.5	1.3	YJL11	CCT7	-6.9	0.7	YER09	PRS	-4.3	0.8
YLR44	-	3.4	1.8	YGR18	RNR4	2.5	1.3	YDR44	DOT	-6.9	0.7	YNR06	DSE	-4.3	0.8
YBL01	-	3.4	1.2	YEL07	DSF1	2.5	1.7	YER15	-	-6.9	0.7	YNL24	NAR	-4.3	0.8
YJL15	INO1	3.4	1.3	YNR07	DSF1	2.5	1.7	YJL12	LSM1	-6.9	0.7	YLL03	PRP	-4.3	0.8
YOR01	TIR2	3.4	1.3	YDL15	-	2.5	1.2	YLR21	FRE1	-6.9	0.6	YJL01	CCT	-4.2	0.9
YKL09	CWP2	3.4	1.1	YJR09	BUD4	2.5	1.2	YLR04	-	-6.8	0.5	YDL17	GLT	-4.2	0.9
YHR09	HXT1	###	0.1	YOR2(	HIS3	2.5	1.3	YER05	HIS1	-6.7	0.7	YCR07	RSA	-4.2	0.8
YAR07	PHO11	###	0.3	YOL03	-	2.5	1.3	YLR41	BER	-6.6	0.7	YDL23	PHO	-4.2	0.9
YHR21	PHO12	###	0.3	YHR15	RTT1	2.5	1.4	YFR04	ERJ5	-6.6	0.6	YGR16	PUS	-4.2	0.8
YDR38	ATO3	###	0.2	YOL02	IFM1	2.5	1.3	YCL00	-	-6.6	0.6	YOR17	SYC	-4.2	0.8
YNL04	-	###	0.2	YJL15	SNA3	2.5	1.3	YLR44	FPR4	-6.5	0.7	YBL10	RTG	-4.2	0.8
YDR22	-	###	0.3	YOR35	HAP5	2.5	1.3	YKR02	GCN	-6.4	0.7	YHR00	GPA	-4.2	0.8
YMR01	HXT2	###	0.0	YLL01	PUF3	2.5	1.4	YJL15	FAR1	-6.4	0.5	YKR00	RSC	-4.2	0.8
YJL19	RPS14	###	0.4	YJL20	RCY1	2.5	1.2	YER07	ALD5	-6.4	0.6	YMR2:	DML	-4.2	0.8
YHR21	IMD2	###	0.1	YLR43	ECM3	2.5	1.2	YHR04	-	-6.3	0.7	YKL10	AAT	-4.2	0.8
YGR12	MEP1	###	0.3	YNL24	SLA2	2.5	1.2	YCL06	VAC1	-6.3	0.7	YGR13	CAF	-4.2	0.8
YNL09	-	###	0.3	YDL17	DLD2	2.5	1.2	YNL15	RPC	-6.2	0.6	YOL12	YGK	-4.2	0.8
YDL24	-	###	0.4	YLR11	AVL9	2.5	1.3	YPL27	SAM	-6.2	0.6	YLR04	---	-4.2	0.7
YCR05	-	###	0.5	YDL02	-	2.5	1.3	YIL021	RPB	-6.2	0.7	YER02	GCD	-4.2	0.9
YIL013	PDR11	###	0.5	YCL02	HBN1	2.5	1.3	YKL15	SRP	-6.2	0.7	YBL02	HAP	-4.2	0.8
YOR37	ATF1	###	0.4	YDR42	SNX4	2.5	1.3	YDR49	SEC2	-6.1	0.7	YNR05	POP	-4.2	0.9
YLR32	NMA1	###	0.5	YJL12	-	2.5	1.3	YGL02	GET	-6.1	0.7	YNL07	RNH	-4.2	0.7
YBL03	URA7	###	0.5	YER15	BUR6	2.5	1.2	YMR18	SSO	-6.1	0.6	YPR12	ANT	-4.2	0.8
YLR44	FPR4	###	0.5	YPR0(	HAL1	2.5	1.3	YPR05	NHP6	-6.1	0.7	YKL07	DHR	-4.1	0.7
YML00	ERG6	###	0.6	YMR17	ECM5	2.5	1.3	YHL04	ARN	-6.1	0.4	YNR04	MVD	-4.1	0.8
YOL02	TSR4	###	0.6	YOR13	ORT1	2.5	1.2	YOL16	BDS	-6.0	0.8	YBR02	POA	-4.1	0.8
YDR16	-	###	0.5	YBR04	ZTA1	2.5	1.2	YPL25	BBP1	-6.0	0.7	YOL09	WRS	-4.1	0.8
YEL04	UTR2	###	0.5	YER13	PMD1	2.5	1.2	YGR12	-	-6.0	0.6	YOR05	NOB	-4.1	0.8
YGR24	SDA1	###	0.5	YDR53	HSP3	2.5	1.8	YEL01	EDC	-6.0	0.7	YLR22	BUR	-4.1	0.8
YNL14	AAH1	###	0.4	YGL16	RAD5	2.5	1.2	YIL072	HOP	-5.9	0.3	YGR07	PAC	-4.1	0.8
YER04	MXR1	###	0.4	YDR31	IPK1	2.5	1.3	YOR10	RAS	-5.9	0.7	YPL06	BTS	-4.1	0.8
YEL01	EDC3	###	0.6	YDR08	STN1	2.5	1.4	YIL015	BAR	-5.9	0.5	YIL085	KTR	-4.1	0.8
YDR46	RMT2	###	0.5	YBR0(	GPI18	2.5	1.2	YMR13	CIN4	-5.9	0.6	YNL04	---	-4.1	0.8
YOR15	PDR5	###	0.5	YDR51	EMI1	2.5	1.2	YDR24	MNN	-5.9	0.7	YBL02	NCL	-4.1	0.9
YIR020	YVH1	###	0.6	YDR08	AFR1	2.5	1.3	YKR03	GMH	-5.9	0.7	---	---	-4.1	0.8
YNL11	DBP2	###	0.5	YIL026	IRR1	2.5	1.3	YGL21	SKI8	-5.9	0.7	YEL04	GDA	-4.1	0.8
YJR04	CYC1	###	0.7	YMR2:	ESC1	2.5	1.4	YDL22	HO	-5.9	0.7	YBR26	REI1	-4.1	0.7
YHR19	RIX1	###	0.5	YNL19	CHS1	2.5	1.2	YMR09	NPL6	-5.9	0.6	YPR17	BSP	-4.1	0.8
YOR27	YTM1	###	0.5	YER13	-	2.5	1.3	YOR12	LEO1	-5.9	0.7	YBR18	---	-4.1	0.8
YPR00	SUT2	###	0.7	YNL13	SRV2	2.5	1.2	YIL011	TIR3	-5.8	0.5	YBR21	---	-4.1	0.8
YER15	-	###	0.6	YJL13	AIM2	2.5	1.3	YGL24	RTF1	-5.8	0.7	YBR22	---	-4.1	0.8
YML08	DUS1	###	0.6	YER14	UBP5	2.4	1.2	YOR24	PUS7	-5.8	0.6	YML02	UNG	-4.1	0.8
YKL07	DHR2	-9.9	0.5	YHR15	TDA1	2.4	1.3	YGR24	SDA	-5.8	0.7	YBL01	FUS	-4.1	0.6
YMR13	RRB1	-9.8	0.6	YCR06	TAH1	2.4	1.3	YCL02	FUS1	-5.8	0.3	YIL145	PAN	-4.1	0.9
YHR17	NMD3	-9.8	0.5	YCL01	NFS1	2.4	1.3	YGL25	RTG	-5.8	0.7	YGL17	---	-4.1	0.8
YGL11	NSA1	-9.7	0.6	YKL12	MYO	2.4	1.3	YIL158	AIM2	-5.8	0.6	YGR20	SER	-4.1	0.9
YBR09	PHO5	-9.7	0.5	YHL02	RIM4	2.4	1.4	YNL29	PUS4	-5.7	0.7	YEL01	EDC	-4.1	0.8

YGL16	SUA5	-9.7	0.7	YKL06	YNK1	2.4	1.2	YGR24	BRF1	-5.7	0.7	YOL02	---	-4.1	0.8
YNL03	-	-9.7	0.5	YDR05	YOS9	2.4	1.2	YER10	GLE2	-5.7	0.8	YDR41	ERD	-4.1	0.8
YNL01	-	-9.7	0.5	YGL22	VID30	2.4	1.3	YHR13	SPL2	-5.7	0.6	YOR30	RAX	-4.1	0.7
YJL20	NUC1	-9.5	0.7	YEL00	YEA4	2.4	1.3	YOR31	SFG1	-5.7	0.6	YML11	TAF8	-4.1	0.8
YOR21	SAS5	-9.4	0.7	YDR40	PDR1	2.4	1.2	YFL06	COS4	-5.7	0.7	YHR12	MSH	-4.1	0.7
YDL18	LYS20	-9.4	0.7	YJL02	RNR2	2.4	1.3	YGR29	COS6	-5.7	0.7	YOL09	TRM	-4.1	0.8
YPR12	ANT1	-9.4	0.6	YDR12	SAC6	2.4	1.2	YNL33	COS1	-5.7	0.7	YPL15	TGS	-4.1	0.8
YBR24	ENP1	-9.4	0.5	YJL01	MAD3	2.4	1.3	YDL03	PUS9	-5.6	0.7	YJR02	---	-4.1	0.8
YJR07	LIA1	-9.4	0.6	YER14	FTR1	2.4	1.4	YCL03	HIS4	-5.6	0.7	YJL19	---	-4.1	0.8
YGR18	HGH1	-9.4	0.6	YGL02	CWH	2.4	1.3	YML06	POB3	-5.6	0.7	YBL03	POL	-4.0	0.8
YML07	HMG1	-9.3	0.6	YBR00	UGA2	2.4	1.2	YOL02	TSR4	-5.6	0.8	YNL11	CYB	-4.0	0.7
YNR01	SMM1	-9.3	0.6	YOL05	AIM39	2.4	1.2	YPL08	RPS9	-5.6	0.8	YJR00	SUI2	-4.0	0.8
YOL12	TRM11	-9.2	0.6	YGR25	-	2.4	1.3	YNL25	GIS2	-5.6	0.8	YLR04	---	-4.0	0.7
YBR27	EFM2	-9.2	0.6	YMR07	CTF1	2.4	1.2	YGR10	-	-5.6	0.2	YHR19	RIX1	-4.0	0.8
YKL02	MAK11	-9.2	0.5	YOR01	RTS1	2.4	1.3	YGR10	-	-5.6	0.2	YML02	YOX	-4.0	0.7
YJL19	PHO90	-9.2	0.6	YBR08	SEC1	2.4	1.3	YIL082	-	-5.6	0.2	YNL17	NOP	-4.0	0.8
YOR14	PNO1	-9.1	0.6	YDR45	NHX1	2.4	1.2	YIL082	-	-5.6	0.2	YJL20	NUC	-4.0	0.8
YOL08	REX4	-9.0	0.5	YGL11	-	2.4	1.2	YPR14	ASN1	-5.6	0.7	YJL06	UTP	-4.0	0.8
YER12	NSA2	-9.0	0.6	YPL07	GCR1	2.4	1.2	YHR20	PPX1	-5.6	0.6	YPL08	RPS	-4.0	0.8
YIL003	CFD1	-9.0	0.6	YGR19	FYV8	2.4	1.3	YLR35	ADE1	-5.6	0.7	YBR16	POP	-4.0	0.9
YIL053	RHR2	-8.9	0.7	YCL06	MRC1	2.4	1.4	YLL00	RTT1	-5.6	0.7	YLL00	RTT1	-4.0	0.8
YGL25	HFM1	-8.9	0.2	YOR31	FAA1	2.4	1.3	YDR42	TIF35	-5.5	0.6	YNL08	TCB	-4.0	0.8
YOL15	HPF1	-8.9	0.2	YDR33	MSN5	2.4	1.2	YGL11	CUE3	-5.5	0.8	YDR37	---	-4.0	0.8
YGL20	MIG2	-8.8	0.5	YDL03	PRM7	2.4	1.8	YEL04	UTR2	-5.5	0.7	YGR12	PPT1	-4.0	0.7
YOR10	RAS1	-8.7	0.6	YMR00	PLB1	2.4	2.1	YJL01	VTC4	-5.5	0.7	YOR25	CLP1	-4.0	0.9
YMR01	SOK2	-8.7	0.5	YKL13	HSK3	2.4	1.2	YFL02	STE2	-5.5	0.5	YGR18	HGH	-4.0	0.8
YOR05	NOB1	-8.7	0.6	YMR04	ARG8	2.4	1.2	YNL14	MFA2	-5.5	0.7	YOL01	HTZ1	-4.0	0.8
YPR11	MRI1	-8.7	0.7	YER05	PIC2	2.4	1.5	YGR12	PPT1	-5.5	0.7	YDR52	---	-4.0	0.8
YPL23	YAR1	-8.6	0.7	YGL09	VPS4	2.4	1.2	YPR13	VPS6	-5.4	0.7	YPR04	TIF5	-4.0	0.9
YNL06	GCD10	-8.6	0.7	YDR45	PPN1	2.4	1.2	YHR17	NMD3	-5.4	0.7	YCR01	MAK	-3.9	0.9
YKR06	UTP30	-8.5	0.6	YBR05	UBP1	2.4	1.2	YCR10	AAD3	-5.4	0.7	YDL04	SIR2	-3.9	0.8
YPR05	YMC1	-8.5	0.7	YER18	-	2.4	1.3	YOL16	AAD1	-5.4	0.7	YHR09	HXT	-3.9	0.7
YNL14	ALF1	-8.5	0.6	YPR04	PUF2	2.4	1.3	YER18	-	-5.4	0.6	YHL00	STE	-3.9	0.9
YLR28	NNT1	-8.4	0.7	YBR20	LDH1	2.4	1.2	YDL15	MSH5	-5.4	0.6	YOR01	PET	-3.9	0.8
YNR04	MVD1	-8.4	0.6	YPL07	UBP1	2.4	1.2	YOR05	ETT1	-5.4	0.7	YHR05	CIC1	-3.9	0.8
YOL09	TRM10	-8.4	0.6	YBR29	VBA2	2.3	1.2	YDR41	ERD1	-5.4	0.7	YOR34	---	-3.9	0.8
YMR04	ERB1	-8.4	0.6	YLR34	FKS1	2.3	1.2	YOR09	RKI1	-5.4	0.7	YBL03	URA	-3.9	0.8
YJL06	UTP18	-8.4	0.6	YHR13	ARO9	2.3	1.2	YPL10	ELP4	-5.3	0.7	YOR10	VAM	-3.9	0.8
YBR03	HMT1	-8.3	0.6	YDL08	-	2.3	1.3	YOL05	GPM	-5.3	0.6	YDR24	MNN	-3.9	0.8
YDR23	LYS4	-8.3	0.7	YOR21	AIM4	2.3	1.2	YAR01	KIN3	-5.3	0.7	YGR07	---	-3.9	0.8
YOR16	PNS1	-8.3	0.5	YOR22	WTM	2.3	1.4	YAR01	ADE1	-5.3	0.7	YLR05	IES3	-3.9	0.8
YJL20	ACO2	-8.2	0.6	YDR47	-	2.3	1.3	YOR28	PLP2	-5.3	0.8	YGL06	PUS	-3.9	0.8
YBR20	DUR1,	-8.2	0.5	YDL07	YET3	2.3	1.3	YGL06	DUO1	-5.3	0.6	YKL01	RAM	-3.9	0.8
YLR13	ZRT2	-8.2	0.7	YJL08	IML2	2.3	1.2	YNL08	TCB2	-5.3	0.8	YDR44	---	-3.9	0.8
YKR08	RPF2	-8.1	0.5	YHR00	SOD2	2.3	1.2	YBR02	YPK3	-5.3	0.6	YBL00	HTB	-3.9	0.8
YJL12	GCD14	-8.1	0.6	YHR17	FMO1	2.3	1.4	YDR53	APA2	-5.3	0.7	YLR07	RGR	-3.9	0.9
YDR12	-	-8.1	0.7	YPL17	TRE1	2.3	1.2	YKL18	LOT5	-5.3	0.7	YGR28	BIO2	-3.9	0.8
YLL06	MMP1	-8.1	0.3	YDL02	ARP2	2.3	1.2	YMR14	TIF34	-5.2	0.8	YIL119	RPI1	-3.9	0.8
YCR07	RSA4	-8.1	0.6	YKL21	UBA1	2.3	1.2	YGL25	MNT2	-5.2	0.7	YGL01	ATE1	-3.9	0.8
YDR14	MKC7	-8.1	0.7	YPR18	ATG1	2.3	1.4	YHR10	GEP4	-5.2	0.7	YBR09	PHO	-3.9	0.8
YBL10	SRO77	-8.0	0.5	YBR21	PYC2	2.3	1.2	YLR12	-	-5.2	0.5	YNL24	RPA	-3.9	0.8
YML05	IMD4	-8.0	0.7	YGR13	-	2.3	1.2	YKL11	KTI12	-5.2	0.7	YKL12	PMU	-3.9	0.9
YNR06	FRE4	-8.0	0.4	YBR23	ABD1	2.3	1.2	YOR19	BFR1	-5.2	0.8	YMR15	NUP	-3.9	0.8
YLR00	NOC3	-8.0	0.7	YMR12	-	2.3	1.2	YNR04	AGA1	-5.2	0.3	YPL21	NIP7	-3.9	0.8
YOR37	GDH1	-8.0	0.7	YNL16	FMP4	2.3	1.3	YKL20	STE6	-5.2	0.7	YLR41	CDC	-3.9	0.8
YER00	NUG1	-8.0	0.7	YCR08	ABP1	2.3	1.3	YPR07	NOT5	-5.2	0.7	YKL03	IXR1	-3.9	0.8
YNL31	EMW1	-7.9	0.7	YMR30	UBP1	2.3	1.3	YNR05	LYS9	-5.1	0.7	YNR00	RPC	-3.9	0.9
YGR17	RBG2	-7.9	0.7	YOR13	IDH2	2.3	1.2	YNL11	DBP2	-5.1	0.7	YPL27	---	-3.9	0.7
YHL01	PRS3	-7.9	0.7	YPL22	MMT2	2.3	1.3	YHR05	CPR2	-5.1	0.7	YDL04	PRP	-3.9	0.9
YNR00	RPC34	-7.9	0.6	YBL10	ECM2	2.3	1.2	YGR26	FOL2	-5.1	0.8	YOR32	LDB1	-3.8	0.8
YER06	FCY22	-7.8	0.5	YDR03	ARO3	2.3	1.2	YPL08	SEN5	-5.1	0.7	YOL15	FRE	-3.8	0.4
YBR09	PHO3	-7.8	0.6	YPL10	MSD1	2.3	1.2	YBR16	TOS1	-5.1	0.7	YJR12	---	-3.8	0.8
YIL020	HIS6	-7.8	0.5	YDL05	MCH1	2.3	1.3	YBL07	-	-5.1	0.7	YMR03	MIH1	-3.8	0.9
YIL064	SEE1	-7.7	0.5	YOL00	SIN3	2.3	1.2	YPR11	MRI1	-5.1	0.8	YDR36	BCP	-3.8	0.8
YDL03	PUS9	-7.7	0.7	YBR13	AGP2	2.3	1.4	YHR08	STE1	-5.1	0.7	YIL159	BNR	-3.8	0.8
YHR07	TRM5	-7.7	0.5	YLR30	HRI1	2.3	1.2	YOL05	-	-5.1	0.8	YNL14	MFA	-3.8	0.8
YNL18	IPI3	-7.7	0.6	YGL01	JAC1	2.3	1.4	YOR07	UFE1	-5.0	0.7	YFL03	RPL	-3.8	0.8
YML09	TAF13	-7.7	0.6	YMR13	PSO2	2.3	1.3	YPL09	PNG1	-5.0	0.8	YJL07	PSF	-3.8	0.8
YIL104	SHQ1	-7.7	0.7	YMR26	TPS3	2.3	1.2	YCL05	KRR1	-5.0	0.6	YBR19	MSI1	-3.8	0.8
YBL05	TOD6	-7.7	0.4	YGR23	SLI1	2.3	1.2	YGR22	TOS2	-5.0	0.6	YPR11	---	-3.8	0.9
YMR21	GUA1	-7.7	0.7	YKL17	NNK1	2.3	1.3	YGL04	RNA1	-5.0	0.7	YOL00	PHO	-3.8	0.8

YHR1(	SOL3	-7.6	0.7	YJL18!	-	2.3	1.5	YGR1(	TIF4(	-5.0	0.7	YHR1:	NSG	-3.8	0.8
YDL05	LHP1	-7.6	0.7	YLR35	ATG3	2.3	1.5	YBR22	MCX:	-5.0	0.7	YMR1!	TPP:	-3.8	0.8
YLR21	FRE1	-7.6	0.6	YER06	THO1	2.3	1.2	YKL18	-	-5.0	0.6	YHR01	ARD	-3.8	0.8
YBR0(	GPI18	-7.6	0.7	YCR0:	RBK1	2.3	1.3	YLR19	HCR:	-5.0	0.8	YGR0;	PRP	-3.8	0.8
YBL06	PRS4	-7.6	0.7	YDR0!	BMH:	2.3	1.2	YAL02	SAW	-4.9	0.7	YNL28	CAF-	-3.8	0.8
YMR2!	HAS1	-7.5	0.5	YAR02	-	2.3	1.3	YBL11	YRF1	-4.9	0.7	YLR02	---	-3.8	0.8
YJL12:	ALB1	-7.5	0.5	YBL06	PRX1	2.3	1.4	YDR5-	YRF1	-4.9	0.7	YJL12!	GCD	-3.8	0.8
YMR2:	RRP5	-7.5	0.7	YDR4!	VPS3	2.3	1.2	YEL07	YRF1	-4.9	0.7	YOR3!	---	-3.8	0.7
YGR1!	NSR1	-7.5	0.5	YFL01	-	2.2	1.3	YER1!	YRF1	-4.9	0.7	YPL27	---	-3.8	0.7
YBR2-	GPX2	-7.5	0.4	YJL16-	TPK1	2.2	1.5	YGR2!	YRF1	-4.9	0.7	YDR12	TRM	-3.8	0.8
YDR3(	BCP1	-7.5	0.5	YLL06	AYT1	2.2	1.3	YHL05	YRF1	-4.9	0.7	YBL06	AST:	-3.8	0.8
YDR5:	CAB1	-7.5	0.7	YLR0(	NSE1	2.2	1.2	YHR21	YRF1	-4.9	0.7	YDR2!	SUR	-3.8	0.7
YJR06	HAM1	-7.5	0.6	YFR01	FAB1	2.2	1.2	YIL177	YRF1	-4.9	0.7	YPR1(	FHL:	-3.8	0.8
YBR1(	YMC2	-7.4	0.5	YLR25	SYM:	2.2	1.6	YJL22!	YRF1	-4.9	0.7	YEL03	RAD	-3.8	0.8
YOL07	BRX1	-7.4	0.6	YHR1!	SPO1	2.2	1.2	YLL06	YRF1	-4.9	0.7	YLR44	SEC	-3.8	0.9
YDL16	FAP7	-7.4	0.7	YKL21	COS!	2.2	1.3	YLL06	YRF1	-4.9	0.7	YJR14	HMS	-3.8	0.7
YKL19	DPH2	-7.4	0.6	YNL25	RAD!	2.2	1.3	YLR46	YRF1	-4.9	0.7	YKL05	NUP	-3.8	0.8
YDR2-	PRP28	-7.3	0.6	YMR2!	YKU7	2.2	1.3	YLR46	YRF1	-4.9	0.7	YHR0:	BRL:	-3.7	0.7
YDL01	NOP1	-7.3	0.7	YGL02	STT3	2.2	1.2	YML1:	-	-4.9	0.7	YLR38	CSR	-3.7	0.8
YKL14	LTV1	-7.3	0.4	YBR2!	PCA1	2.2	1.4	YNL33	YRF1	-4.9	0.7	YML0(	GIS-	-3.7	0.8
YEL06	SIT1	-7.3	0.2	YGR2(	APL6	2.2	1.2	YOR3!	YRF1	-4.9	0.7	YMR1-	TIF3	-3.7	0.9
YOR0!	RKI1	-7.3	0.6	YCR1(	-	2.2	1.4	YPL28	YRF1	-4.9	0.7	YGR0;	SMD	-3.7	0.8
YKL18	PRS1	-7.3	0.7	YAL00	SSA1	2.2	1.2	YPR2(	-	-4.9	0.7	---	---	-3.7	0.7
YNL17	NOP1:	-7.2	0.6	YKR0!	-	2.2	1.3	YJR01	-	-4.9	0.7	YOL1:	PFK:	-3.7	0.8
YDL11	TRM3	-7.2	0.7	YGR1:	PEX4	2.2	1.3	YEL05	MAK:	-4.9	0.7	YML0!	GIM!	-3.7	0.8
YER0!	HIS1	-7.2	0.8	YDR2(	VPS6	2.2	1.3	YHR0(	LRP1	-4.9	0.5	YNL30	YPT:	-3.7	0.8
YDR3!	UTP5	-7.2	0.5	YBR05	-	2.2	1.4	YBR2(	APM:	-4.9	0.8	YMR0:	PEX	-3.7	0.9
YDL12	-	-7.2	0.6	YGR2!	COQ(	2.2	1.4	YMR2:	TRI1	-4.9	0.7	YER11	SPR	-3.7	0.8
YPL00	HAT1	-7.2	0.5	YIL07:	SPO:	2.2	1.6	YER0(	THO:	-4.9	0.7	YGL0(	CDH	-3.7	0.8
YJL21:	OPT1	-7.1	0.6	YPR1!	TPO3	2.2	1.2	YNL23	PDR:	-4.9	0.8	YEL03	MCM	-3.7	0.8
YNL20	RIO2	-7.1	0.6	YDL19	UFD2	2.2	1.2	YER01	BIM1	-4.9	0.7	YLR35	ADE	-3.7	0.8
YDR0:	DAS2	-7.1	0.7	YMR0(	SEG1	2.2	1.4	YNL00	HRB:	-4.9	0.8	YJL17(	ATG	-3.7	0.9
YNL11	NCS2	-7.1	0.6	YGL1!	MDS:	2.2	1.3	YBR1(	YMC:	-4.9	0.6	YEL02	SNU	-3.7	0.9
YCR0:	FEN2	-7.1	0.4	YMR2:	ERG(	2.2	1.2	YOR1(	IES4	-4.9	0.7	YJL00:	CCT	-3.7	0.9
YER11	KAP12	-7.1	0.6	YIL15!	GUT:	2.2	1.2	YIL08!	KTR7	-4.8	0.7	YMR0'	SEC	-3.7	0.8
YDR1(	TRM8:	-7.1	0.6	YJL17-	KRE!	2.2	1.2	YFR0(	SAD:	-4.8	0.7	YJR03	GEA	-3.7	0.8
YDL08	LUC7	-7.1	0.6	YIL017	VID2(	2.2	1.3	YMR1:	RRB:	-4.8	0.8	YML01	TRM	-3.7	0.8
YNL30	RPL18	-7.0	0.7	YEL06	NPR:	2.2	1.2	YBL11	YRF1	-4.8	0.7	YOR2!	TIM1	-3.7	0.9
YKR0:	RPC3;	-7.0	0.6	YBL06	KIP1	2.2	1.2	YDR5-	YRF1	-4.8	0.7	YNL31	---	-3.7	0.9
YDR1:	TRM1	-7.0	0.5	YLR08	SMC-	2.2	1.3	YEL07	YRF1	-4.8	0.7	YLR01	MEU	-3.7	0.8
YGR1:	UTP8	-7.0	0.6	YEL02	GEA:	2.2	1.2	YER1!	YRF1	-4.8	0.7	YMR2(	---	-3.7	0.8
YDL20	TRM8	-7.0	0.7	YIL162	SUC:	2.2	1.2	YFL06	YRF1	-4.8	0.7	YOR1-	PNO	-3.7	0.8
YHR2(	MNL1	-7.0	0.7	YDR01	-	2.2	1.4	YGR2!	YRF1	-4.8	0.7	YCL05	KAR	-3.7	0.7
YML05	CYB2	-7.0	0.3	YMR2'	BUL1	2.2	1.2	YHL05	YRF1	-4.8	0.7	YKR0-	UIP5	-3.7	0.8
YEL03	UTR4	-7.0	0.7	YAL01	MDM	2.2	1.2	YHR21	-	-4.8	0.7	YJL06	NUP	-3.7	0.8
YDL03	SLM3	-6.9	0.8	YMR2(	ROT1	2.2	1.3	YIL177	YRF1	-4.8	0.7	YKL02	MAK	-3.7	0.8
YIL16!	-	-6.9	0.3	YKL01	ARC1	2.2	1.2	YJL22!	YRF1	-4.8	0.7	YBL08	---	-3.7	0.6
YOL1!	HPF1	-6.9	0.3	YJR05	PTK2	2.2	1.2	YLL06	YRF1	-4.8	0.7	YMR0!	NPL(	-3.7	0.8
YDR2(	PHM6	-6.9	0.3	YGR0!	-	2.2	1.3	YLL06	YRF1	-4.8	0.7	YLR17	DPH	-3.7	0.8
YNL02	-	-6.9	0.7	YOR3!	-	2.2	1.3	YLR46	YRF1	-4.8	0.7	YDR2(	---	-3.7	0.8
YOL01	-	-6.8	0.5	YJR04	CYC1	###	0.3	YLR46	YRF1	-4.8	0.7	YML0!	VPS	-3.6	0.8
YHR1(	GEP4	-6.8	0.6	YDR4(	MFA1	###	0.3	YML1:	-	-4.8	0.7	YBR1!	---	-3.6	0.8
YJL05	MTR4	-6.8	0.6	YMR3:	FET4	###	0.3	YNL33	YRF1	-4.8	0.7	YLR39	COX	-3.6	0.8
YIL091	UTP25	-6.8	0.6	YLR04	-	###	0.2	YOR3!	YRF1	-4.8	0.7	YLR05	REX	-3.6	0.8
YER0(	UTP7	-6.8	0.6	YBR0!	PHO!	###	0.3	YPL28	YRF1	-4.8	0.7	YMR0-	ARG	-3.6	0.7
YNR01	URK1	-6.8	0.7	YDL22	HO	###	0.4	YPR2(	-	-4.8	0.7	YOR2(	DED	-3.6	0.8
YCL03	HIS4	-6.8	0.8	YHR0-	FSH1	###	0.4	YLL06	MHT:	-4.8	0.6	YKR0!	BAS	-3.6	0.9
YOR2-	PUS7	-6.8	0.6	YKL12	OAC:	###	0.3	YGR0:	TIM2	-4.8	0.7	YCR0:	SNT	-3.6	0.8
YLR19	PWP1	-6.8	0.6	YOR1(	LEU9	###	0.5	YOR2-	SRL1	-4.8	0.8	YOL07	AVO	-3.6	0.8
YBL02	NCL1	-6.8	0.7	YGL1(	-	###	0.4	YOL04	NGL:	-4.8	0.7	YGR0:	MSB	-3.6	0.8
YMR2(	ABZ2	-6.8	0.7	YDR2:	-	-9.6	0.4	YLR18	TOS-	-4.8	0.7	YOR1(	MTR	-3.6	0.9
YDL17	GLT1	-6.7	0.8	YER07	ALD5	-9.6	0.4	YNR02	CPR(	-4.8	0.8	YOL14	RRP	-3.6	0.8
YFR0!	IRC7	-6.7	0.5	YER0(	MNN:	-9.2	0.4	YPL01	VTC:	-4.8	0.7	YBR1:	SHE	-3.6	0.8
YBL07	KTI11	-6.7	0.7	YDR2(	PHM(	-9.1	0.2	YDL20	TRM(	-4.8	0.8	YGR1;	MSM	-3.6	0.9
YIL00(	URM1	-6.7	0.6	YOR1(	RAS1	-9.0	0.5	YKL00	DID4	-4.8	0.7	YLR40	---	-3.6	0.8
YOL1!	ENB1	-6.7	0.3	YER06	FCY2	-8.9	0.4	YGR1(	NOP'	-4.8	0.5	YHR0(	STE:	-3.6	0.8
YPL08	RPS9/	-6.7	0.7	YJL21:	OPT1	-8.8	0.5	YBR1-	SUP-	-4.8	0.8	YEL04	UTR	-3.6	0.8
YGR2:	ZPR1	-6.7	0.5	YBR0(	TEC1	-8.8	0.4	YGR1(	GTR:	-4.7	0.8	YGR1!	SKI6	-3.6	0.9
YHR0(	GPA1	-6.7	0.7	YHR1:	SPL2	-8.7	0.4	YDL21	SHR:	-4.7	0.8	YHR0(	RRP	-3.6	0.8
YLR39	AFG2	-6.6	0.7	YLR04	-	-8.5	0.4	YIL11-	POR:	-4.7	0.6	YML0(	---	-3.6	0.8
YKR0!	TRM2	-6.6	0.6	YNL27	MET:	-8.5	0.3	YPL27	SAM-	-4.7	0.8	YPR17	DPB	-3.6	0.8

YPL27	SAM4	-6.6	0.7	YOR1(	PNS1	-8.5	0.5	YDL14	RPN5	-4.7	0.7	YGL09	SRM	-3.6	0.9
YMR2:	RNT1	-6.6	0.5	YAR07	PHO:	-8.2	0.5	YMR1:	DLT1	-4.7	0.8	YFR02	CDC	-3.6	0.9
YPL18	RTT10	-6.6	0.7	YHR2	PHO:	-8.2	0.5	YOL01	TLG2	-4.7	0.7	YOR3:	TEA:	-3.6	0.8
YJR11	-	-6.6	0.6	YHR12	FUR1	-8.1	0.6	YAL03	MTW	-4.7	0.7	YBL00	---	-3.6	0.9
YOL15	ZPS1	-6.6	0.7	YJR01	ILV3	-8.1	0.5	YDR25	CHL4	-4.7	0.7	YLR26	SEC	-3.6	0.8
YPL19	-	-6.5	0.7	YIL015	BAR1	-8.0	0.4	YOR2:	ABP1	-4.7	0.7	YNL20	RTT:	-3.6	0.8
YMR1	TIF34	-6.5	0.8	YKL04	PHD1	-8.0	0.4	YCL03	STE5	-4.7	0.7	YNR0:	PPG	-3.6	0.9
YKR09	BAS1	-6.5	0.8	YOR2:	DFR1	-7.9	0.6	YDR11	ALT2	-4.6	0.6	YKL16	PIR1	-3.6	0.8
YDR42	BNA7	-6.5	0.7	YLR45	SST2	-7.9	0.4	YML09	-	-4.6	0.8	YGL07	RPB	-3.6	0.8
YJR00	SUI2	-6.5	0.7	YJL15	FAR1	-7.9	0.4	YMR0(	KAR5	-4.6	0.6	YOR08	TGL!	-3.6	0.8
YBR14	MAK5	-6.5	0.7	YGL25	ADH4	-7.6	0.6	YJL00	SYS1	-4.6	0.8	YDR27	RNH	-3.6	0.9
YPL12	NAN1	-6.5	0.6	YBR09	PHO:	-7.3	0.5	YML07	FPR3	-4.6	0.7	YCR05	CTR	-3.6	0.8
YHR14	IMP3	-6.5	0.6	YEL04	GLY1	-7.3	0.6	YJR11	NNF1	-4.6	0.7	YLR40	---	-3.6	0.8
YHR05	CIC1	-6.5	0.6	YMR2:	-	-7.2	0.4	YJR04	POL:	-4.6	0.8	YOR15	---	-3.6	0.8
YPL10	ELP4	-6.5	0.7	YCL02	FUS1	-7.1	0.2	YDR04	HEM:	-4.6	0.8	YMR1	---	-3.6	0.8
YJL21:	-	-6.5	0.5	YGL0(	LEU1	-6.9	0.5	YML04	PRM(	-4.6	0.4	YCL06	VAC	-3.6	0.8
YBR06	ECM2	-6.5	0.5	YGR1(	CLB6	-6.9	0.5	YPR16	RHO:	-4.6	0.8	YBR25	TRS:	-3.6	0.8
YDL13	RGT2	-6.4	0.6	YKL09	MBR:	-6.8	0.5	YBR04	CST2	-4.6	0.8	YDR49	SEC	-3.5	0.8
YER04	CAJ1	-6.4	0.8	YNL14	MFA2	-6.8	0.5	YJL20	NUC:	-4.6	0.8	YLL01	COX	-3.5	0.7
YNL29	PUS4	-6.4	0.6	YIL121	QDR2	-6.7	0.5	YPR01	TIF6	-4.6	0.8	YDL03	PRP	-3.5	0.8
YDL06	TSR1	-6.4	0.6	YHR0(	GPA1	-6.6	0.6	YDR28	PHM(	-4.5	0.5	YBL08	ALG	-3.5	0.8
YLR18	EMG1	-6.4	0.7	YHR06	RRP4	-6.6	0.7	YGL23	TAN1	-4.5	0.7	YBL08	ALG	-3.5	0.8
YGL17	ROK1	-6.4	0.5	YER15	-	-6.4	0.7	YPR05	TFB4	-4.5	0.7	YML05	IMD4	-3.5	0.9
YGL23	TAN1	-6.4	0.7	YLL06	MHT:	-6.4	0.5	YOR0(	TSR:	-4.5	0.7	YDL10	---	-3.5	0.8
YPL16	-	-6.4	0.7	YIL119	RPI1	-6.2	0.6	YMR2:	RNA:	-4.5	0.7	YDR15	RPA	-3.5	0.8
YER02	MIG3	-6.3	0.5	YGL03	AGA2	-6.2	0.4	YNL22	URE2	-4.5	0.7	YOR14	SFL:	-3.5	0.8
YOL12	HRP1	-6.3	0.7	YLR06	-	-6.2	0.6	YLR26	PDR8	-4.5	0.6	YOR19	LIP5	-3.5	0.8
YEL04	GLY1	-6.3	0.5	YOL06	MET2	-6.2	0.6	YDR02	SES1	-4.5	0.8	YGR12	COG	-3.5	0.9
YLR10	ERG2	-6.3	0.7	YER12	DSE1	-6.2	0.6	YMR14	-	-4.5	0.8	YKR02	GCN	-3.5	0.9
YNL29	TRF5	-6.3	0.7	YHR2(	BAT1	-6.1	0.5	YPR05	BRR:	-4.5	0.8	YOR25	---	-3.5	0.9
YKL08	HOT1:	-6.3	0.6	YOR2:	STE4	-6.0	0.7	YGR2:	ZPR1	-4.5	0.7	YDR18	ATC	-3.5	0.8
YBR26	REI1	-6.2	0.6	YBR24	RRT2	-6.0	0.7	YER0(	NUG:	-4.5	0.7	YOL14	DCP	-3.5	0.8
YPR01	RPA1:	-6.2	0.7	YJR15	PGU:	-6.0	0.3	YOR1(	LEU9	-4.5	0.8	YDR24	---	-3.5	0.8
YKL21	TRP3	-6.2	0.7	YER04	MXR:	-6.0	0.6	YER17	RAD2	-4.5	0.7	YLR22	CCC	-3.5	0.9
YFR03	MET1(	-6.2	0.5	YML02	APT1	-6.0	0.7	YBR29	MAL:	-4.5	0.5	YOR3:	MRS	-3.5	0.8
YML09	-	-6.2	0.7	YOR34	-	-6.0	0.6	YGL21	YPT3	-4.5	0.8	YNR0:	DBP	-3.5	0.8
YNL24	RPA49	-6.2	0.6	YNL05	COX5	-6.0	0.6	YJR11	ILM1	-4.5	0.7	YOR14	ELG	-3.5	0.8
YHR2(	SCH9	-6.2	0.7	YCL03	GFD2	-5.9	0.5	YCL03	RRP7	-4.5	0.7	YBR15	RIB7	-3.5	0.8
YLR01	BRE2	-6.2	0.7	YFL02	STE2	-5.9	0.5	YER13	GLC7	-4.5	0.7	YLR28	NNT	-3.5	0.8
YOR29	RRS1	-6.1	0.6	YKR06	UTP3	-5.8	0.6	YOL13	CDC:	-4.5	0.8	YJL08	ARP	-3.5	0.8
YGR28	IMA1	-6.1	0.8	YCL05	KAR4	-5.8	0.5	YGR2(	SER2	-4.4	0.8	YDL06	UBC	-3.5	0.8
YMR2(	-	-6.1	0.7	YLR34	KAP9	-5.8	0.7	YDR29	SRP1	-4.4	0.7	YPR11	RPC	-3.5	0.8
YOR2	FSF1	-6.1	0.8	YER09	PRS2	-5.8	0.6	YOL11	MSB4	-4.4	0.7	YMR25	---	-3.5	0.9
YGL14	ARO2	-6.1	0.7	YML08	DUS1	-5.8	0.6	YDR46	RMT2	-4.4	0.7	YNL07	MKS	-3.5	0.9
YGR12	PPT1	-6.1	0.6	YPL24	-	-5.8	0.7	YMR29	GOT:	-4.4	0.8	YPR18	SMX	-3.5	0.8
YJL09	CHS6	-6.1	0.7	YNL21	-	-5.7	0.6	YFL00	VTC2	-4.4	0.7	YOR22	RPB	-3.5	0.8
YAR03	PRM9	-6.1	0.7	YGR05	MUP:	-5.7	0.5	YOL09	WRS	-4.4	0.8	YOR0(	---	-3.5	0.8
YOL14	NOP8	-6.1	0.6	YMR1:	SAS2	-5.7	0.7	YMR0	ABF2	-4.4	0.8	YJR11	NNF	-3.5	0.8
YNL01	SPO1	-6.1	0.6	YLL06	MMP	-5.7	0.4	YKL04	PRI2	-4.4	0.7	YNL21	MGS	-3.5	0.8
YDL21	NOP6	-6.1	0.5	YCR02	RHB1	-5.6	0.7	YGL03	AGA2	-4.4	0.6	YKL18	PRS	-3.5	0.9
YLR40	UTP21	-6.1	0.7	YHR03	-	-5.6	0.5	YER07	ICP5	-4.3	0.8	YLR34	KAP	-3.5	0.9
YDR27	RNH2(	-6.0	0.8	YAL04	AIM2	-5.6	0.6	YOR2:	SAS5	-4.3	0.8	YKL07	---	-3.5	0.8
YGR14	ENP2	-6.0	0.5	YOR0(	CYT1	-5.5	0.6	YKL02	MAK:	-4.3	0.7				
YGR2(	ELP2	-6.0	0.6	YDL04	SIR2	-5.5	0.6	YNL17	NOP:	-4.3	0.8				
YLR43	TSR2	-6.0	0.6	YCR01	MAK:	-5.5	0.7	YBR12	OPY:	-4.3	0.6				
YLR07	RFU1	-6.0	0.5	YDR47	TRS3	-5.5	0.7	YDR15	GIR2	-4.3	0.7				
YOR3	NOP58	-6.0	0.7	YLR39	AFG2	-5.5	0.6	YBL02	HAP:	-4.3	0.8				
YDR08	RRP1	-6.0	0.5	YLR36	GRX8	-5.4	0.6	YOR3:	SPS4	-4.3	0.5				
YOL13	ALR1	-6.0	0.7	YNR05	BIO4	-5.4	0.6	YGR15	RSR:	-4.3	0.7				
YPL15	TGS1	-6.0	0.7	YOR05	NOB:	-5.4	0.6	YLR20	SEC:	-4.3	0.8				
YOR34	RPA43	-6.0	0.7	YER17	GRX4	-5.3	0.6	YKR01	YPT5	-4.3	0.8				
YML1(	-	-6.0	0.7	YOR04	RSB1	-5.3	0.4	YHR1(	CDC:	-4.3	0.8				
YJL07	PSF2	-6.0	0.7	YBL06	PRS4	-5.3	0.7	YCL05	KAR4	-4.3	0.7				
YJL10	PRM1(	-6.0	0.3	YNL11	DBP2	-5.2	0.6	YNL20	RTT1	-4.3	0.8				
YLL05	FRE6	-5.9	0.7	YDL24	LRG1	-5.2	0.6	YDL21	NOP(	-4.3	0.6				
YCL03	GFD2	-5.9	0.7	YEL01	EDC3	-5.2	0.7	YLR10	SEN2	-4.3	0.8				
YBL11	YRF1-	-5.9	0.4	YLR41	-	-5.1	0.6	YJL01	CCT:	-4.3	0.8				
YDR54	YRF1-	-5.9	0.4	YOR1(	RGS2	-5.1	0.4	YPL25	HFI1	-4.3	0.8				
YER19	YRF1-	-5.9	0.4	YGR15	CYS4	-5.1	0.7	YDL04	PRP1	-4.2	0.8				
YFL06	YRF1-	-5.9	0.4	YPL01	VTC3	-5.1	0.6	YPR03	ERV2	-4.2	0.8				
YGR29	YRF1-	-5.9	0.4	YER18	-	-5.1	0.5	YLR35	ILV5	-4.2	0.7				

YHL04	YRF1-	-5.9	0.4	YNR0(	NRM1	-5.1	0.6	YMR1(	HLJ1	-4.2	0.7
YHR21	-	-5.9	0.4	YDR34	HXT7	-5.0	0.4	YBR09	RXT2	-4.2	0.7
YIL177	YRF1-	-5.9	0.4	YDR34	HXT6	-5.0	0.4	YDL20	NHP2	-4.2	0.7
YJL225	YRF1-	-5.9	0.4	YNL06	GCD1	-5.0	0.7	YLR03	RSC5	-4.2	0.6
YLL06	YRF1-	-5.9	0.4	YEL00	YEA6	-5.0	0.7	YGR0(	MUQ	-4.2	0.8
YLL06	YRF1-4	-5.9	0.4	YPL27	SAM4	-5.0	0.7	YLR36	RPS2	-4.2	0.7
YLR46	YRF1-	-5.9	0.4	YGR28	-	-5.0	0.6	YLR28	NNT1	-4.2	0.8
YML13	-	-5.9	0.4	YML04	PRM(	-4.9	0.4	YLR41	CDC7	-4.2	0.8
YNL33	YRF1-	-5.9	0.4	YKL02	-	-4.9	0.7	YLR17	CBF5	-4.2	0.7
YPL28	YRF1-	-5.9	0.4	YHR16	DBP8	-4.9	0.6	YDR34	SVF1	-4.2	0.7
YPR20	-	-5.9	0.4	YOR00	TSR3	-4.9	0.7	YDR42	RPN9	-4.2	0.8
YHR16	DBP8	-5.9	0.6	YDR41	ERD1	-4.9	0.7	YBR24	-	-4.2	0.7
YDR03	KRS1	-5.9	0.7	YIL040	APQ1	-4.9	0.7	YOR18	SER1	-4.2	0.8
YJR00	MPP1(	-5.9	0.5	YLR24	-	-4.9	0.7	YER02	GCD1	-4.1	0.8
YOL06	APM4	-5.9	0.8	YJL01	VTC4	-4.8	0.6	YIL013	PDR1	-4.1	0.8
YBR24	HIS7	-5.9	0.7	YDR41	SYF1	-4.8	0.6	YNL24	VPS7	-4.1	0.8
YFR03	PHO4	-5.9	0.6	YJR07	LIA1	-4.8	0.7	YGR03	-	-4.1	0.5
YIL019	FAF1	-5.9	0.4	YIL104	SHQ1	-4.8	0.7	YOR03	CKB2	-4.1	0.8
YNL23	-	-5.8	0.6	YLR18	EMG1	-4.8	0.7	YMR20	INP1	-4.1	0.7
YML02	APT1	-5.8	0.7	YNL29	PUS4	-4.8	0.6	YOL10	TPT1	-4.1	0.7
YDL04	PRP11	-5.8	0.8	YNL14	AAH1	-4.8	0.7	YDR14	TAF1	-4.1	0.7
YGL12	PRP43	-5.8	0.7	YGL21	SKI8	-4.8	0.7	YLR00	CMS1	-4.1	0.6
YOR33	ALA1	-5.8	0.8	YPL18	RTT1	-4.7	0.7	YLR14	SPE4	-4.1	0.7
YOR09	TMA46	-5.8	0.7	YOL01	RCL1	-4.7	0.6	YAL06	PAU8	-4.1	0.6
YLR45	SST2	-5.8	0.6	YDL24	-	-4.7	0.7	YBL10	PAU9	-4.1	0.6
YNL21	RAP1	-5.8	0.7	YGR07	-	-4.7	0.6	YDR54	PAU1	-4.1	0.6
YMR18	-	-5.8	0.7	YKL09	CUE2	-4.7	0.7	YGL26	PAU1	-4.1	0.6
YOL12	TRM13	-5.8	0.7	YNR04	-	-4.7	0.7	YGR29	PAU1	-4.1	0.6
YCR05	PWP2	-5.8	0.6	YLR14	SPE4	-4.6	0.6	YHL04	PAU1	-4.1	0.6
YKL11	KTI12	-5.8	0.7	YGR10	-	-4.6	0.2	YHR15	SPO1	-4.1	0.8
YDR36	KEI1	-5.8	0.7	YGR10	-	-4.6	0.2	YLR42	URA4	-4.1	0.7
YOR02	-	-5.8	0.7	YIL082	-	-4.6	0.2	YPR05	MAK3	-4.0	0.7
YDR29	SRP10	-5.8	0.6	YIL082	-	-4.6	0.2	YOL07	BRX1	-4.0	0.7
YGR03	-	-5.7	0.4	YOR09	RKI1	-4.6	0.7	YER05	HMF1	-4.0	0.8
YKL10	AAT1	-5.7	0.7	YCL02	-	-4.5	0.6	YFR03	RSC8	-4.0	0.7
YDR10	ARX1	-5.7	0.6	YHR10	TRR2	-4.5	0.7	YGL09	USE1	-4.0	0.6
YOR19	MCA1	-5.7	0.8	YOL05	SPE2	-4.5	0.7	YCR01	-	-4.0	0.6
YNL06	NOP2	-5.7	0.6	YDR16	-	-4.5	0.7	YOR21	AIM4	-4.0	0.8
YJR04	URB2	-5.7	0.7	YJL19	ELO1	-4.5	0.6	YKL12	SRP2	-4.0	0.6
YHR12	FUR1	-5.7	0.8	YKL13	RMA1	-4.5	0.6	YFL02	GYP8	-4.0	0.7
YHL03	EFM1	-5.7	0.6	YIR02(	YVH1	-4.5	0.8	YBL01	FUS3	-4.0	0.6
YDR02	FAL1	-5.7	0.6	YHR08	IPI1	-4.5	0.6	YBR09	PHO5	-4.0	0.7
YJR09	JJJ3	-5.7	0.6	YPL08	RPS9	-4.5	0.7	YBL11	YRF1	-4.0	0.3
YNL07	IMP4	-5.7	0.6	YCR07	RSA4	-4.5	0.6	YBL11	YRF1	-4.0	0.3
YGR10	NOP7	-5.7	0.5	YJL12	GCD1	-4.5	0.7	YDR54	YRF1	-4.0	0.3
YLR10	SEN2	-5.7	0.8	YJR14	HMS2	-4.4	0.6	YEL07	YRF1	-4.0	0.3
YDR41	RRP17	-5.7	0.6	YPR12	CLB5	-4.4	0.7	YER19	YRF1	-4.0	0.3
YCL05	KRR1	-5.6	0.6	YDR44	APT2	-4.4	0.7	YGR29	YRF1	-4.0	0.3
YKL12	PGM1	-5.6	0.6	YNL04	-	-4.4	0.2	YHR21	YRF1	-4.0	0.3
YER17	GRX4	-5.6	0.6	YOL01	-	-4.4	0.2	YHR21	YRF1	-4.0	0.3
YDL03	DBP10	-5.6	0.6	YOR07	-	-4.4	0.2	YIL177	YRF1	-4.0	0.3
YDR26	CIA1	-5.6	0.8	YOR35	VTS1	-4.4	0.6	YJL225	YRF1	-4.0	0.3
YJL11	CCT7	-5.6	0.6	YPL27	SAM3	-4.4	0.7	YLL06	YRF1	-4.0	0.3
YGR21	RTA1	-5.6	0.4	YMR01	-	-4.4	0.6	YLL06	YRF1	-4.0	0.3
YDL11	RRP42	-5.6	0.8	YNR05	NOG2	-4.3	0.6	YLR46	YRF1	-4.0	0.3
YBR15	CNS1	-5.6	0.6	YML06	TEM1	-4.3	0.7	YLR46	YRF1	-4.0	0.3
YDR11	ALT2	-5.6	0.5	YGR03	-	-4.3	0.5	YML13	-	-4.0	0.3
YMR31	ADH6	-5.6	0.7	YOL04	NGL1	-4.3	0.7	YNL33	YRF1	-4.0	0.3
YLR22	ECM22	-5.6	0.7	YNL11	NCS2	-4.3	0.6	YOR39	YRF1	-4.0	0.3
YDR39	HPT1	-5.6	0.6	YBR26	REI1	-4.3	0.6	YPL28	YRF1	-4.0	0.3
YNL13	KRE33	-5.6	0.7	YOL14	ARG8	-4.3	0.7	YPR20	-	-4.0	0.3
YPL05	PDR12	-5.6	0.5	YDL00	PTC1	-4.3	0.7	YLL00	MMM	-4.0	0.7
YLR01	MEU1	-5.5	0.7	YKL12	RRN3	-4.3	0.7	YHR08	GAR1	-4.0	0.7
YCR04	BUD23	-5.5	0.7	YOR37	-	-4.3	0.5	YBR24	ARO4	-4.0	0.7
YPR01	TIF6	-5.5	0.8	YBR06	TRM7	-4.3	0.7	YCR10	RDS1	-4.0	0.6
YPL01	RRP12	-5.5	0.6	YLR02	SDO1	-4.3	0.7	YLL04	ATG1	-4.0	0.7
YER12	LCP5	-5.5	0.6	YDL12	VCX1	-4.3	0.7	YDL10	NSE4	-4.0	0.7
YPL09	NOG1	-5.5	0.6	YML05	IMD4	-4.3	0.8	YLR08	EMP7	-3.9	0.8
YOL04	NOP12	-5.5	0.6	YBR03	HMT1	-4.3	0.7	YGL07	RPB9	-3.9	0.8
YML01	TRM9	-5.4	0.7	YKL07	DHR2	-4.2	0.7	YOL01	-	-3.9	0.7
YDR35	SBE2	-5.4	0.8	YDR18	ATC1	-4.2	0.6	YKL11	VPH2	-3.9	0.7

YOR1	RIO1	-5.4	0.6	YGR1	PPT1	-4.2	0.7	YBL11	-	-3.9	0.6
YOL01	RCL1	-5.4	0.7	YER1	GLE2	-4.2	0.8	YDR5	-	-3.9	0.6
YPL21	NIP7	-5.4	0.6	YOR2	YTM1	-4.2	0.7	YEL07	-	-3.9	0.6
YPL20	TYW1	-5.4	0.7	YPL10	ELP4	-4.2	0.7	YER1	-	-3.9	0.6
YDR0	SNF11	-5.4	0.7	YHR1	THP2	-4.2	0.7	YFL06	-	-3.9	0.6
YIR03	-	-5.4	0.6	YAL05	ECM	-4.2	0.5	YGR2	-	-3.9	0.6
YBR0	TRM7	-5.4	0.7	YDL16	CDC	-4.2	0.7	YHL05	-	-3.9	0.6
YBR1	SUP4	-5.4	0.8	YGR1	CLB1	-4.2	0.7	YHR21	-	-3.9	0.6
YJR14	HMS2	-5.4	0.6	YGR1	-	-4.2	0.4	YIL17	-	-3.9	0.6
YDR2	MNN1	-5.4	0.8	YHR1	RIX1	-4.2	0.7	YJL22	-	-3.9	0.6
YLR12	DIP2	-5.3	0.7	YGL0	LSG1	-4.1	0.7	YLL06	-	-3.9	0.6
YPR0	TAH1	-5.3	0.7	YOR2	CLP1	-4.1	0.8	YLL06	-	-3.9	0.6
YDR0	RRP8	-5.3	0.6	YGR0	THG1	-4.1	0.7	YLR46	-	-3.9	0.6
YHR1	-	-5.3	0.7	YNR0	-	-4.1	0.6	YLR46	-	-3.9	0.6
YDL14	NOP1	-5.3	0.6	YIL13	FLX1	-4.1	0.7	YML1	-	-3.9	0.6
YOL0	WRS1	-5.3	0.7	YDR2	RNH	-4.1	0.7	YNL33	-	-3.9	0.6
YPR0	ASA1	-5.3	0.7	YKL20	STE6	-4.1	0.7	YOR3	-	-3.9	0.6
YOL05	SPE2	-5.3	0.7	YPR05	TFB4	-4.1	0.7	YPL28	-	-3.9	0.6
YMR0	-	-5.3	0.4	YER07	PTP3	-4.1	0.7	YPR2	-	-3.9	0.6
YLR14	SPE4	-5.3	0.7	YOL12	YGK	-4.1	0.7	YLR42	RPN	-3.9	0.7
YDL06	-	-5.3	0.6	YBR0	RFC5	-4.1	0.7	YGL00	COG	-3.9	0.7
YOL07	NBA1	-5.3	0.6	YDR23	LYS4	-4.0	0.8	YDR41	RRP	-3.9	0.7
YAL03	RBG1	-5.2	0.7	YKL01	SWD	-4.0	0.7	YPR0	TIF5	-3.9	0.8
YGL0	LSG1	-5.2	0.7	YDR4	IZH1	-4.0	0.7	YJR00	-	-3.9	0.7
YOR0	CIN5	-5.2	0.7	YHR0	RPP1	-4.0	0.7	YKR07	-	-3.9	0.7
YNR0	NOG2	-5.2	0.6	YOR3	CIR2	-4.0	0.8	YDR37	VPS	-3.9	0.7
YPL04	NOP4	-5.2	0.6	YIL08	KTR7	-4.0	0.7	YOR2	RUD	-3.9	0.8
YAL01	CYS3	-5.2	0.8	YMR3	SAM	-4.0	0.7	YBL08	ALG	-3.9	0.8
YOR0	UTP23	-5.2	0.6	YPL27	SAM	-4.0	0.7	YBL08	ALG	-3.9	0.8
YDR4	TRS31	-5.2	0.8	YDL20	TRM	-4.0	0.8	YLR32	SFH1	-3.9	0.8
YLR38	CSR1	-5.2	0.7	YHR0	QCR	-4.0	0.7	YMR3	FET4	-3.9	0.8
YNL15	RPC31	-5.2	0.7	YKL06	-	-4.0	0.6	YKL18	SPE1	-3.9	0.8
YJL13	YUR1	-5.2	0.7	YDR2	MET	-3.9	0.5	YMR0	ARP	-3.9	0.8
YHR0	IPI1	-5.2	0.6	YGR0	TPC1	-3.9	0.7	YAR0	SEN	-3.9	0.8
YML0	TRM1	-5.2	0.8	YLR39	COX	-3.9	0.8	YOR3	ALA1	-3.9	0.8
YLR28	-	-5.2	0.7	YJL01	RPC1	-3.9	0.5	YHR1	-	-3.9	0.8
YJR13	NMD5	-5.2	0.7	YDR1	TRM	-3.9	0.7	YJR01	TMA	-3.9	0.8
YGL05	TYW3	-5.2	0.7	YPL19	-	-3.9	0.8	YKR02	RPC	-3.9	0.7
YGR1	MTR3	-5.2	0.7	YDR2	SNU	-3.9	0.7	YER0	FCY2	-3.9	0.7
YOL1	BDS1	-5.2	0.7	YPL03	MET	-3.9	0.7	YLL05	FRE	-3.9	0.8
YIL10	DPH1	-5.2	0.7	YLR32	NMA	-3.9	0.8	YNR0	-	-3.9	0.8
YNL12	NRK1	-5.1	0.7	YKR0	PCC1	-3.9	0.8	YNL21	-	-3.9	0.8
YNL22	ADE1	-5.1	0.8	YPL05	PDR1	-3.9	0.6	YBR0	RFC5	-3.9	0.8
YDL12	VCX1	-5.1	0.7	YLR21	CLB4	-3.9	0.7	YKL16	TPK	-3.9	0.7
YHR1	UTP9	-5.1	0.6	YJR10	RSM	-3.9	0.7	YDR2	ATP5	-3.9	0.8
YBL05	-	-5.1	0.7	YBR0	TAT1	-3.9	0.7	YGL1	CDC	-3.9	0.8
YLR00	CMS1	-5.1	0.6	YLR38	REH1	-3.9	0.8	YOL1	WSC	-3.9	0.8
YBR1	POP7	-5.1	0.8	YKR05	TRM	-3.9	0.7	YKL04	VPS	-3.9	0.8
YPR1	NOC4	-5.1	0.6	YOR0	UTP2	-3.8	0.7	YDR2	-	-3.9	0.6
YMR0	UTP15	-5.1	0.7	YPL26	ACM	-3.8	0.7	YPL23	RVB	-3.9	0.8
YLL01	SOF1	-5.1	0.6	YKL10	HAP	-3.8	0.7	YOR0	ATX2	-3.8	0.8
YMR2	ERG2	-5.1	0.8	YBR1	GDT1	-3.8	0.7	YKR0	PCC	-3.8	0.8
YAL02	SAW1	-5.1	0.7	YBL01	FUS3	-3.8	0.6	YDL05	PBP	-3.8	0.8
YGL2	ZRT1	-5.1	0.5	YHR0	HXT1	-3.8	0.7	YLR26	YPT	-3.8	0.6
YPL08	SEN5	-5.1	0.7	YJR14	-	-3.8	0.6	YOL12	HRP	-3.8	0.7
YKL14	RPC2	-5.1	0.7	YLR35	ILV5	-3.8	0.8	YCR1	AAD	-3.8	0.8
YPL08	ELP3	-5.1	0.8	YDL11	RRP	-3.8	0.8	YDL09	SRP	-3.8	0.8
YDR5	SAM2	-5.1	0.7	YOL11	PAP2	-3.8	0.7	YGL1	SEH	-3.8	0.8
YDR2	BFR2	-5.1	0.6	YHR0	HXT4	-3.8	0.3	YBR0	TRM	-3.8	0.8
YOL06	PRS5	-5.0	0.7	YNL24	SUI1	-3.8	0.8	YPR1	RPN	-3.8	0.8
YDR1	ATC1	-5.0	0.7	YJL17	ATG	-3.8	0.8	YDR2	RTT1	-3.8	0.7
YEL04	TCA17	-5.0	0.7	YER16	CHD	-3.8	0.7	YPL10	-	-3.8	0.8
YNL06	YDJ1	-5.0	0.8	YDL05	LHP1	-3.8	0.8	YBR21	-	-3.8	0.8
YLL00	MMM1	-5.0	0.7	YGR0	SLX9	-3.8	0.7	YBR22	-	-3.8	0.8
YJL01	CCT3	-5.0	0.7	YDR1	RSM	-3.8	0.7	YBR2	-	-3.8	0.8
YDL15	SAS1	-5.0	0.5	YJR11	NNF1	-3.8	0.7	YGL1	SUT1	-3.8	0.8
YLR40	DUS3	-5.0	0.6	YOR1	SFL1	-3.7	0.7	YPL21	PUS	-3.8	0.7
YNL14	PGA2	-5.0	0.7	YGR2	PHO	-3.7	0.7	YBL05	SEC	-3.8	0.8
YJL01	NOP9	-5.0	0.6	YDR0	IPT1	-3.7	0.8	YJR01	SPC	-3.8	0.8
YJR02	MDE1	-5.0	0.7	YDR5	PAD1	-3.7	0.6	YLR38	REH	-3.8	0.8
YER0	ARB1	-5.0	0.7	YDR0	FAL1	-3.7	0.7	YPL15	PRM	-3.8	0.7

YJR07	NPA3	-5.0	0.8	YBR02	OLA1	-3.7	0.8	YGL25	ZRT1	-3.8	0.6
YNL16	IBD2	-5.0	0.6	YGL02	SCW	-3.7	0.7	YOR2(	PAC1	-3.8	0.8
YGL12	NAB2	-5.0	0.7	YNL21	PEX1	-3.7	0.7	YOL06	MET2	-3.8	0.8
YPR11	RPC4(	-5.0	0.7	YOL15	GRE2	-3.7	0.7	YBR21	AME1	-3.8	0.8
YLR24	-	-5.0	0.7	YNL28	CAF4	-3.7	0.7	YPL05	ARL3	-3.7	0.7
YBR23	-	-5.0	0.5	YDR28	NSE3	-3.7	0.7	YHR13	YCK1	-3.7	0.8
YMR0(	ADI1	-5.0	0.6	YJR01	-	-3.7	0.7	YLR36	NMD4	-3.7	0.8
YLL03	RIX7	-5.0	0.6	YIL103	DPH1	-3.7	0.7	YDR38	YRA1	-3.7	0.8
YMR3:	ELP6	-4.9	0.8	YJL18	-	-3.7	0.7	YML01	SPT5	-3.7	0.8
YDR44	UTP6	-4.9	0.6	YMR0(	MIC1	-3.7	0.8	YDR08	RRP8	-3.7	0.6
YLR35	ADE13	-4.9	0.8	YNL10	AIM3	-3.7	0.7	YNR0(	-	-3.7	0.6
YOR34	RPA19	-4.9	0.8	YDR11	ALT2	-3.7	0.6	YDR3(	HNT2	-3.7	0.8
YPL11	PEX25	-4.9	0.7	YNL23	CSL4	-3.7	0.7	YPL21	SRP7	-3.7	0.7
YBR24	-	-4.9	0.7	YLR13	ZRT2	-3.7	0.8	YHR12	LSM1	-3.7	0.8
YKR04	UIP5	-4.9	0.7	YPR01	TIF6	-3.7	0.8	YGR0(	ADE6	-3.7	0.8
YMR0:	PEX12	-4.9	0.7	YJL13	LCB3	-3.7	0.8	YPR18	RPO2	-3.7	0.8
YGR0(	VHT1	-4.9	0.5	YBR0(	RER2	-3.7	0.7	YER13	RTR1	-3.7	0.8
YGR28	BIO2	-4.9	0.7	YNL02	-	-3.7	0.8	YBL03	URA7	-3.7	0.8
YKL16	TPK3	-4.9	0.7	YPL18	POS5	-3.6	0.7	YGL12	NAB2	-3.7	0.8
YPR19	RPC82	-4.9	0.6	YLR18	SAM1	-3.6	0.7	YPL23	SUI3	-3.7	0.8
YIR01:	SQT1	-4.9	0.6	YBR1(	FES1	-3.6	0.8	YLL06	AYT1	-3.7	0.7
YLR00	RLP24	-4.9	0.7	YLR41	BER1	-3.6	0.7	YPR16	JIP5	-3.7	0.8
YGR1:	-	-4.9	0.7	YAL03	POP5	-3.6	0.7	YDR48	VPS6	-3.7	0.8
YLR33	SGD1	-4.8	0.6	YFR0(	NIC9	-3.6	0.8	YML01	OST6	-3.7	0.8
YDR12	INO2	-4.8	0.7	YKR08	OMA1	-3.6	0.7	YCR02	FEN2	-3.7	0.7
YAL02	MAK1(	-4.8	0.5	YML09	-	-3.6	0.8	YDR12	TRM1	-3.7	0.7
YMR24	ZRC1	-4.8	0.8	YHR07	TRM5	-3.6	0.7	YDR52	AGE1	-3.7	0.8
YOR25	TMA16	-4.8	0.6	YBR19	-	-3.6	0.6	YML1(	URA5	-3.7	0.8
YGR23	PEX21	-4.8	0.7	YOL02	DIS3	-3.6	0.8	YNL05	OCA2	-3.6	0.8
YPR16	TIF3	-4.8	0.8	YPR06	ARO7	-3.6	0.7	YDL00	MED1	-3.6	0.7
YPL15	PRP46	-4.8	0.8	YLR45	-	-3.6	0.8	YNL24	RPA4	-3.6	0.8
YMR3(	ATM1	-4.8	0.8	YHR19	UTP9	-3.6	0.6	YBR27	EFM2	-3.6	0.8
YNL21	IES2	-4.8	0.7	YLR10	SEN2	-3.6	0.8	YER14	-	-3.6	0.8
YER04	TPA1	-4.8	0.7	YOL0(	PHO8	-3.6	0.8	YIR00	DJP1	-3.6	0.8
YLL05	AQY2	-4.8	0.6	YDR08	RRP1	-3.6	0.7	YDR13	-	-3.6	0.7
YMR2:	ERG8	-4.8	0.8	YML02	NSE5	-3.6	0.7	YDL24	LRG1	-3.6	0.7
YGR28	-	-4.8	0.7	YGR07	PAC1	-3.6	0.8	YHR14	CRP1	-3.6	0.6
YPR04	TIF5	-4.8	0.8	YBR27	EFM2	-3.6	0.7	YOR31	NOP5	-3.6	0.8
YJL14	RPA34	-4.8	0.6	YGR18	HGH1	-3.6	0.8	YPL12	HHO1	-3.6	0.8
YGR2(	-	-4.8	0.7	YNL27	-	-3.6	0.5	YPL20	TYW	-3.6	0.8
YLR07	XYL2	-4.8	0.6	YMR1:	DLT1	-3.6	0.8	YIL005	EPS1	-3.6	0.8
YDL03	PRM7	-4.8	0.4	YLR25	-	-3.6	0.8	YIL078	THS1	-3.6	0.8
YAR0(	SEN34	-4.8	0.7	YLR00	NOC:	-3.5	0.8	YBR05	MUM	-3.6	0.7
YLR31	TAD3	-4.8	0.8	YNL07	IMP4	-3.5	0.7	YLR43	TSR2	-3.6	0.7
YOL04	NGL1	-4.7	0.8	YHR05	MED(	-3.5	0.8	YDR06	LCB2	-3.6	0.8
YNL11	NOP15	-4.7	0.6	YMR2:	RNH1	-3.5	0.7	YOR37	ATF1	-3.6	0.8
YHR12	ARP1	-4.7	0.7	YKL10	AAT1	-3.5	0.8	YBR22	-	-3.6	0.8
YHR08	GAR1	-4.7	0.7	YDR0(	-	-3.5	0.7	YDR03	ARO3	-3.6	0.8
YDL23	PHO13	-4.7	0.8	YPL16	SVS1	-3.5	0.6	YKR05	MRS4	-3.6	0.8
YPL26	DIM1	-4.7	0.7	YDR11	TMA(	-3.5	0.8	YMR22	ERG8	-3.6	0.8
YDR07	IPT1	-4.7	0.7	YDR14	SAN1	-3.5	0.7	YOL08	REX4	-3.6	0.7
YDR5(	GNP1	-4.7	0.7	YBR24	ENP1	-3.5	0.7	YMR1(	ILV2	-3.6	0.8
YHR04	DOG1	-4.7	0.7	YLL04	ATG1	-3.5	0.6	YOR0(	UTP2	-3.6	0.7
YPL03	TRM44	-4.7	0.7	YLR19	PBA1	-3.5	0.8	YDL01	GRX6	-3.6	0.8
YCR03	RRP43	-4.7	0.7	YHR05	CIC1	-3.5	0.7	YDL06	PEX1	-3.6	0.8
YBR25	DUT1	-4.7	0.7	YPL03	TRM4	-3.5	0.7	YOR26	RPN8	-3.6	0.7
YNL00	RLP7	-4.7	0.7	YML12	PHO8	-3.5	0.7	YMR24	CUS1	-3.5	0.8
YDR01	SNQ2	-4.7	0.6	YNR04	AGA1	-3.5	0.4	YLR08	SRL2	-3.5	0.8
YPL06	-	-4.7	0.7	YJL03	TAD2	-3.5	0.7	YLR18	VTA1	-3.5	0.8
YBR04	CST26	-4.7	0.8	YPL18	COA2	-3.5	0.6	YOL14	DCP1	-3.5	0.8
YIL096	-	-4.7	0.6	YBR15	RIB7	-3.5	0.8	YNR05	BRE5	-3.5	0.6
YDR06	RTR2	-4.7	0.6	YMR0(	UTP1	-3.5	0.7	YER0(	NOP1	-3.5	0.6
YJR12	-	-4.7	0.7	YCR04	BUD2	-3.5	0.7	YML06	TEM1	-3.5	0.8
YER02	GCD11	-4.7	0.7	YKL20	ADD6	-3.5	0.8	YDR03	KRS1	-3.5	0.8
YMR3:	-	-4.7	0.7	YPL25	HFI1	-3.5	0.8	YPR16	TIF3	-3.5	0.8
YGR1(	-	-4.7	0.2	YJR06	CBF1	-3.5	0.7	YLR00	NOC:	-3.5	0.8
YGR1(	-	-4.7	0.2	YOR1:	TRS3	-3.5	0.7	YML12	RSC9	-3.5	0.8
YIL082	-	-4.7	0.2	YDR01	KCS1	-3.4	0.8	YML02	RPS1	-3.5	0.8
YIL082	-	-4.7	0.2	YOR17	MED4	-3.4	0.7	YMR25	GFD1	-3.5	0.7
YBR22	-	-4.6	0.7	YLL00	RTT1	-3.4	0.7	YIR01	RPR2	-3.5	0.8
YPR13	RRP9	-4.6	0.7	YBL03	POL1	-3.4	0.8	YGL18	COX4	-3.5	0.7

YNR05	ESF2	-4.6	0.7	YFL02	FRS2	-3.4	0.8	YPL00	HAT1	-3.5	0.7
YLR36	-	-4.6	0.7	YJR02	MDE1	-3.4	0.7	YOR15	PUP1	-3.5	0.8
YCR01	-	-4.6	0.6	YBR25	TRS2	-3.4	0.7	YLR37	SUR4	-3.5	0.9
YLR14	SMD3	-4.6	0.7	YHR17	NMD3	-3.4	0.8	YBL06	PRS4	-3.5	0.8
YGR09	-	-4.6	0.8	YNL07	NIS1	-3.4	0.7	YJL17	ATG2	-3.5	0.9
YOR07	BUD21	-4.6	0.6	YBR02	YPK3	-3.4	0.7	YDL13	SRF1	-3.5	0.8
YBL00	PDR3	-4.6	0.7	YGR14	BTN2	-3.4	0.7	YML03	YMD8	-3.5	0.8
YIR025	MND2	-4.6	0.8	YOR10	INP53	-3.4	0.8	YER17	RAD3	-3.5	0.9
YPR18	RPO26	-4.6	0.8	YOL06	PRS5	-3.4	0.7	YBL04	-	-3.5	0.6
YHR08	LRP1	-4.6	0.6	YNL21	RAP1	-3.4	0.8	YAR00	SWD	-3.5	0.8
YJR01	-	-4.6	0.7	YJR14	MGM	-3.4	0.7	YNL30	MCK1	-3.5	0.7
YPR17	PRP4	-4.6	0.7	YGR10	PCP1	-3.4	0.7	YDL12	-	-3.5	0.7
YDR51	EMI1	-4.6	0.8	YBL00	HTA2	-3.4	0.7	YKL00	MRT4	-3.5	0.7
YIL079	AIR1	-4.6	0.6	YOL09	HMI1	-3.4	0.7	YGR25	NOP1	-3.5	0.7
YAR07	IMD2	-4.5	0.7	YDR02	DAS2	-3.4	0.8	YJR04	NUP8	-3.5	0.8
YHR21	IMD2	-4.5	0.7	YDR14	MTQ2	-3.4	0.8	YER18	ISC10	-3.5	0.7
YLR43	IMD3	-4.5	0.7	YLR35	BUD8	-3.4	0.8	YPL03	MET3	-3.5	0.7
YJL098	SAP18	-4.5	0.7	YKL02	MAK1	-3.4	0.7	YBR12	TFC1	-3.5	0.8
YKR02	GCN3	-4.5	0.8	YOR29	-	-3.4	0.7	YLR15	ASP3	-3.4	0.8
YOR30	MCH5	-4.5	0.6	YPR12	ANT1	-3.4	0.7	YLR15	ASP3	-3.4	0.8
YOR21	STE4	-4.5	0.8	YNR04	COQ1	-3.4	0.7	YLR15	ASP3	-3.4	0.8
YMR12	ECM10	-4.5	0.7	YGL07	HNM1	-3.3	0.8	YLR16	ASP3	-3.4	0.8
YHR20	PPX1	-4.5	0.8	YDR28	RRP4	-3.3	0.7	YMR03	SUB1	-3.4	0.8
YDR28	RRP45	-4.5	0.7	YHR03	PIH1	-3.3	0.8	YOR11	RTC5	-3.4	0.8
YNL20	RTT10	-4.5	0.8	YDR19	REF2	-3.3	0.8	YDR19	REF2	-3.4	0.8
YDL01	CDC7	-4.5	0.8	YMR04	MCM	-3.3	0.7	YJR01	ILV3	-3.4	0.8
YBR08	AAC3	-4.5	0.7	YER13	-	-3.3	0.7	YBL03	POL1	-3.4	0.8
YGL23	ADE5,	-4.5	0.8	YER07	VTC1	-3.3	0.8	YMR00	CDC5	-3.4	0.8
YJR06	RPA12	-4.5	0.7	YBR23	ERT1	-3.3	0.7	YOR14	THI80	-3.4	0.7
YKL09	UTP11	-4.5	0.6	YLR44	FPR4	-3.3	0.8	YDR28	RRP4	-3.4	0.7
YGL03	-	-4.5	0.7	YLR07	RFU1	-3.3	0.7	YMR02	PEX1	-3.4	0.9
YFL04	OTU1	-4.5	0.7	YBR16	UBS1	-3.3	0.8	YDL20	CWC	-3.4	0.7
YOR25	SEC63	-4.5	0.7	YER18	-	-3.3	0.7	YOR05	NOB1	-3.4	0.8
YDR28	ZIP1	-4.5	0.5	YNL24	VPS7	-3.3	0.7	YNL25	NRD1	-3.4	0.8
YKR05	MRS4	-4.5	0.7	YDL06	TSR1	-3.3	0.7	YER12	NSA2	-3.4	0.8
YEL04	IES6	-4.5	0.7	YLR29	GCD7	-3.3	0.8	YOL09	TRM1	-3.4	0.8
YOL09	HMI1	-4.5	0.7	YKL18	PRS1	-3.3	0.8	YOR01	AUS1	-3.4	0.7
YGR09	ASK10	-4.4	0.8	YML10	SEC6	-3.3	0.8	YOL01	HTZ1	-3.4	0.8
YBL04	-	-4.4	0.6	YDL12	-	-3.3	0.7	YNL09	RPS7	-3.4	0.8
YDR48	CWC2	-4.4	0.7	YJR05	HIT1	-3.2	0.6	YOL00	IZH2	-3.4	0.8
YDR09	RLI1	-4.4	0.7	YOL08	REX4	-3.2	0.7	YDR07	IPT1	-3.4	0.7
YNR05	BIO3	-4.4	0.7	YOL10	NDJ1	-3.2	0.4	YMR17	MMT	-3.4	0.8
YLR22	UTP13	-4.4	0.7	YDR45	PFA5	-3.2	0.8	YNL03	GPI1	-3.4	0.7
YOR20	RET1	-4.4	0.8	YOR29	TIM18	-3.2	0.8	YGR13	PRE9	-3.4	0.9
YOR22	RPB8	-4.4	0.8	YDR09	RLI1	-3.2	0.8	YLL06	MMP	-3.4	0.6
YML07	BET5	-4.4	0.7	YOR29	RRS1	-3.2	0.7	YNL04	-	-3.4	0.7
YLR19	PEX13	-4.4	0.8	YKL07	MUD2	-3.2	0.8	YGR11	NUP5	-3.4	0.8
YHR08	RPF1	-4.4	0.6	YNL31	EMW	-3.2	0.8	YGL18	GTS1	-3.3	0.8
YLR29	GCD7	-4.4	0.8	YGL12	SOH1	-3.2	0.7	YCR05	-	-3.3	0.8
YKL00	MET14	-4.4	0.5	YJL13	GLG2	-3.2	0.7	YDR54	YRF1	-3.3	0.1
YPL23	RVB2	-4.4	0.8	YHR00	LEU5	-3.2	0.7	YEL07	YRF1	-3.3	0.1
YOL05	GPD2	-4.4	0.7	YER17	RAD3	-3.2	0.8	YER19	YRF1	-3.3	0.1
YKR00	OSH6	-4.4	0.7	YGL07	RPB9	-3.2	0.8	YGR29	YRF1	-3.3	0.1
YNL11	RPC19	-4.4	0.8	YPL21	PCL8	-3.2	0.8	YIL177	YRF1	-3.3	0.1
YOR01	AUS1	-4.4	0.7	YMR03	MIH1	-3.2	0.8	YJL225	YRF1	-3.3	0.1
YGR16	TIF463	-4.4	0.7	YGL18	COX4	-3.2	0.7	YLR46	YRF1	-3.3	0.1
YEL05	POL5	-4.4	0.7	YNR06	-	-3.2	0.8	YLR46	YRF1	-3.3	0.1
YOR26	-	-4.4	0.7	YMR04	ERB1	-3.2	0.8	YLR46	YRF1	-3.3	0.1
YNL02	FAP1	-4.4	0.7	YDL04	PRP1	-3.2	0.8	YNL33	YRF1	-3.3	0.1
YDR34	SVF1	-4.4	0.7	YOR29	-	-3.2	0.5	YPL28	YRF1	-3.3	0.1
YGL15	ARI1	-4.4	0.7	YDR26	-	-3.2	0.8	YLR19	SIK1	-3.3	0.7
YMR32	SAM4	-4.4	0.7	YJL065	DLS1	-3.2	0.8	YDL17	DLD2	-3.3	0.8
YPL27	SAM4	-4.4	0.7	YOL14	RRP4	-3.2	0.8	YER13	RPS2	-3.3	0.9
YGR07	-	-4.3	0.7	YDR14	PEX7	-3.2	0.8	YBR16	TYR1	-3.3	0.8
YIL110	HPM1	-4.3	0.7	YBR08	POL3	-3.2	0.8	YDR11	APC4	-3.3	0.7
YNL16	-	-4.3	0.7	YHR10	GEP4	-3.2	0.8	YCR03	RRP4	-3.3	0.7
YIL004	BET1	-4.3	0.7	YMR06	KAR5	-3.2	0.7	YER03	YPT3	-3.3	0.9
YGL25	MNT2	-4.3	0.7	YDR18	UPS3	-3.2	0.6	YJL08	ARP4	-3.3	0.8
YNL25	NRD1	-4.3	0.8	YBR22	-	-3.2	0.8	YEL05	POL5	-3.3	0.8
YOR33	TEA1	-4.3	0.7	YDR15	RPA1	-3.1	0.8	YOR20	NPT1	-3.3	0.8
YLR43	IMD3	-4.3	0.7	YBR21	-	-3.1	0.8	YDL16	CDC3	-3.3	0.8

YPL26	KEL3	-4.3	0.7
YOR35	VTS1	-4.3	0.6
YOR00	YSP3	-4.3	0.6
YOR10	LEU9	-4.3	0.8
YNR06	DSE4	-4.3	0.7
YDL15	RPC53	-4.3	0.6
YOR19	LIP5	-4.3	0.7
YJR12	-	-4.3	0.7
YJL08	ARP4	-4.3	0.8
YLR27	DBP9	-4.3	0.6
YPL06	ALD6	-4.3	0.7
YMR07	SEC14	-4.3	0.8
YOR11	RTC5	-4.3	0.8
YJL03	HCA4	-4.3	0.6
YKR03	GAP1	-4.3	0.5
YPL10	-	-4.3	0.8
YGL24	RTF1	-4.3	0.7
YOL13	CDC33	-4.3	0.8
YER00	PAC2	-4.3	0.7
YHR04	-	-4.3	0.8
YGR25	NOP19	-4.2	0.7
YHR06	RRP3	-4.2	0.8
YGR05	LST7	-4.2	0.6
YDR12	DPB4	-4.2	0.8
YPL02	ERG10	-4.2	0.8
YLR08	EMP70	-4.2	0.8
YPL14	KES1	-4.2	0.7
YDL20	NHP2	-4.2	0.7
YPR06	SPE3	-4.2	0.8
YLR38	IKI3	-4.2	0.8
YDR49	IZH1	-4.2	0.7
YGL05	MST27	-4.2	0.4
YDR20	UME6	-4.2	0.8
YGR14	VPS62	-4.2	0.8
YML04	PRM6	-4.2	0.5
YFR00	LOC1	-4.2	0.5
YBL04	FUI1	-4.2	0.7
YJL19	ELO1	-4.2	0.7
YPL05	LCL1	-4.2	0.6
YBR22	MCX1	-4.2	0.8
YPL21	PUS1	-4.2	0.7
YJR10	ADO1	-4.2	0.8
YDR32	ASP1	-4.2	0.7
YBR16	ARL1	-4.1	0.7
YGR10	-	-4.1	0.6
YIL082	-	-4.1	0.6
YOR07	ATX2	-4.1	0.8
YLR17	DPH5	-4.1	0.8
YOL14	RRP40	-4.1	0.8
YIL142	CCT2	-4.1	0.8
YNL08	TCB2	-4.1	0.8
YKL18	SPE1	-4.1	0.6
YKL00	MRT4	-4.1	0.7
YDL18	RBS1	-4.1	0.8
YHR07	ERG7	-4.1	0.7
YPL19	NAB3	-4.1	0.7
YPL13	RDS2	-4.1	0.8
YKL01	URB1	-4.1	0.7
YGR00	TFG2	-4.1	0.8
YOL14	PPM2	-4.1	0.5
YGL09	SRM1	-4.1	0.8
YLR17	CBF5	-4.1	0.8
YLR06	ENV10	-4.1	0.8
YHR03	ERC1	-4.1	0.7
YDR14	MTQ2	-4.1	0.8
YDR39	NCB2	-4.1	0.7
YMR13	-	-4.1	0.9
YOR24	ENV9	-4.1	0.8
YGR03	ACB1	-4.1	0.8
YDR47	SNM1	-4.1	0.8
YJR03	CPR7	-4.1	0.8

YBR22	-	-3.1	0.8
YDR49	SEC2	-3.1	0.8
YGR14	NAT2	-3.1	0.8
YGL05	TYW3	-3.1	0.8
YDR46	TFB3	-3.1	0.8
YBR17	SMY2	-3.1	0.8
YDR24	-	-3.1	0.6
YMR09	NPL6	-3.1	0.7
YBR06	BAP2	-3.1	0.7
YDL16	SFA1	-3.1	0.8
YBR14	-	-3.1	0.7
YHR18	IKI1	-3.1	0.8
YCR01	-	-3.1	0.6
YGR09	TEL2	-3.1	0.8
YDR07	SHU2	-3.1	0.7
YGL08	LCL3	-3.1	0.7
YGL01	ATE1	-3.1	0.8
YDL20	HEM3	-3.1	0.8
YGR03	TIM21	-3.1	0.8
YNL20	RIO2	-3.1	0.8
YAR07	-	-3.1	0.5
YHR21	-	-3.1	0.5
YJL06	UTP1	-3.1	0.7
YGL19	CDC5	-3.1	0.8
YOR14	THI80	-3.1	0.7
YMR22	FSH2	-3.1	0.7
YHR14	IMP3	-3.1	0.7
YDR04	BAP3	-3.1	0.8
YIL027	KRE2	-3.1	0.8
YPL09	GLR1	-3.1	0.8
YMR16	HLJ1	-3.1	0.8
YOL00	IZH2	-3.1	0.8
YDR10	ARX1	-3.0	0.7
YGL01	CKB1	-3.0	0.8
YDR12	-	-3.0	0.8
YMR21	DML1	-3.0	0.8
YJL19	-	-3.0	0.8
YLR01	MEU1	-3.0	0.8
YJL12	NIT2	-3.0	0.7
YJL00	CTK2	-3.0	0.8
YNL18	IPI3	-3.0	0.7
YDR39	HPT1	-3.0	0.7
YGR21	ZPR1	-3.0	0.7
YBR17	SWD	-3.0	0.8
YOR26	PAC1	-3.0	0.8
YKL02	URA6	-3.0	0.7
YMR28	RIT1	-3.0	0.8
YNL31	PHA2	-3.0	0.8
YHR20	RPN1	-3.0	0.8
YDR11	APC4	-3.0	0.7
YBR29	CTP1	-3.0	0.7
YFL03	RPL2	-3.0	0.8
YBR05	MUM	-3.0	0.7
YPR11	MRI1	-3.0	0.8
YJL17	ASG7	-3.0	0.4
YGR04	KSS1	-3.0	0.8
YHL02	OPI1	-3.0	0.8
YFL00	VTC2	-3.0	0.8
YCR04	-	-3.0	0.8
YPR19	ARR1	-3.0	0.8
YLR40	UTP2	-3.0	0.8
YJL01	CCT3	-3.0	0.8
YNL28	POP3	-3.0	0.8
YBL09	MRP2	-3.0	0.7
YPR12	AXL1	-3.0	0.8
YCL00	-	-3.0	0.8
YJL20	NUC1	-2.9	0.8
YPL08	ELP3	-2.9	0.8
YDR39	-	-2.9	0.8
YHR03	BRL1	-2.9	0.7
YDR39	UTP5	-2.9	0.7

YHR13	NSG1	-3.3	0.8
YBR13	SHE3	-3.3	0.8
YDR40	RPB7	-3.3	0.9
YBR00	RER2	-3.3	0.8
YDL18	RBS1	-3.3	0.8
YGR09	TPC1	-3.3	0.8
YPL18	MRN	-3.3	0.8
YOR02	-	-3.3	0.8
YOR24	MET7	-3.3	0.8
YOR19	LIP5	-3.3	0.8
YKL14	LTV1	-3.3	0.7
YGL25	ADH4	-3.3	0.8
YDL24	-	-3.3	0.8
YIL118	RHO3	-3.3	0.8
YIL110	HPM1	-3.3	0.7
YBR23	PBP2	-3.3	0.7
YGL15	-	-3.2	0.8
YNL30	RPL1	-3.2	0.8
YEL04	GLY1	-3.2	0.8
YFL04	SEC5	-3.2	0.9
YKL18	PRS1	-3.2	0.8
YBL03	HEK2	-3.2	0.7
YDR18	UPS3	-3.2	0.7
YBR08	POL3	-3.2	0.8
YOR34	RPA4	-3.2	0.8
YBL02	NCL1	-3.2	0.9
YMR06	RNA1	-3.2	0.8
YNL02	-	-3.2	0.7
YOR11	RPT5	-3.2	0.8
YHR02	PPA1	-3.2	0.9
YLR06	ENV1	-3.2	0.7
YJL01	NOP9	-3.2	0.7
YHL02	OPI1	-3.2	0.8
YJL00	CCT8	-3.2	0.9
YJL03	SNX4	-3.2	0.8

YGL22	FRA2	-4.1	0.7	YOR37	ATF1	-2.9	0.8
YGR09	RRP46	-4.1	0.8	YLR35	ORM1	-2.9	0.7
YOR16	SWT1	-4.1	0.8	YDL02	GPD1	-2.9	0.7
YOL12	YGK3	-4.1	0.7	YLR11	MSL5	-2.9	0.8
YOR10	VAM3	-4.0	0.8	YGL09	NBP3	-2.9	0.8
YGL10	SEH1	-4.0	0.8	YBL06	AST1	-2.9	0.8
YPL11	CAR1	-4.0	0.8	YKR07	DRE2	-2.9	0.7
YGL12	MON1	-4.0	0.5	YGR20	ELP2	-2.9	0.7
YDR42	TIF35	-4.0	0.7	YLR34	-	-2.9	0.8
YOR24	MET7	-4.0	0.8	YPR14	NOC4	-2.9	0.7
YML02	NSE5	-4.0	0.7	YGR15	NSR1	-2.9	0.7
YER17	DMC1	-4.0	0.6	YMR05	SEN1	-2.9	0.7
YDR28	-	-4.0	0.8	YCR03	RRP4	-2.9	0.8
YDR02	NSI1	-4.0	0.7	YDL06	UBC9	-2.9	0.8
YOL14	PEX11	-4.0	0.7	YGL16	SUT1	-2.9	0.8
YLL00	DRS1	-4.0	0.6	YER00	NUG1	-2.9	0.8
YDR41	SYF1	-4.0	0.8	YHL00	LAG1	-2.9	0.8
YBL02	-	-4.0	0.7	YGL15	CDC4	-2.9	0.8
YOL05	GPM3	-4.0	0.7	YDL15	CLB3	-2.9	0.8
YOR03	CKB2	-4.0	0.8	YLR07	BOS1	-2.9	0.8
YBR21	-	-4.0	0.8	YNL16	PSD1	-2.9	0.8
YBR22	-	-4.0	0.8	YKR02	GCN3	-2.9	0.8
YHL01	OTU2	-4.0	0.7	YOR36	PRT1	-2.9	0.8
YGR12	-	-4.0	0.5	YGL08	MF(A	-2.9	0.4
YJL008	CCT8	-4.0	0.7	YKR08	PRP1	-2.9	0.8
YKL00	AUR1	-4.0	0.8	YKL11	PRR1	-2.9	0.8
YIL145	PAN6	-4.0	0.8	YOR27	FSF1	-2.9	0.8
YDR11	FOB1	-4.0	0.8	YGR21	-	-2.8	0.8
YPR06	ARO7	-4.0	0.7	YKL00	MRT4	-2.8	0.7
YLR19	SIK1	-3.9	0.7	YNL19	DUG3	-2.8	0.8
YDR35	TRR1	-3.9	0.9	YLL06	-	-2.8	0.5
YBR29	CTP1	-3.9	0.7	YML10	CAC2	-2.8	0.8
YHR14	RPC10	-3.9	0.8	YGL17	-	-2.8	0.8
YKL17	EBP2	-3.9	0.6	YHR16	MTG2	-2.8	0.8
YNR01	PRP2	-3.9	0.7	YLR40	FLD1	-2.8	0.8
YFL00	DEG1	-3.9	0.7	YDR30	GPI11	-2.8	0.8
YGL02	CGR1	-3.9	0.5	YIL074	SER3	-2.8	0.8
YKR02	DBP7	-3.9	0.6	YNL30	TOS6	-2.8	0.8
YKR06	MET1	-3.9	0.7	YOR27	CAF2	-2.8	0.8
YGL16	SUT1	-3.9	0.8	YGR10	SRB5	-2.8	0.8
YKR09	SRP40	-3.9	0.6	YOL00	CSI2	-2.8	0.7
YLR06	-	-3.9	0.8	YBR15	AMN1	-2.8	0.7
YGL04	DST1	-3.9	0.8	YHR03	ERC1	-2.8	0.8
YLR12	-	-3.9	0.7	YGR09	UTP2	-2.8	0.8
YPR06	FCY1	-3.9	0.8	YDL10	KIN28	-2.8	0.8
YJR06	CCT5	-3.9	0.8	YOL07	BRX1	-2.8	0.8
YDR49	PUF6	-3.9	0.6	YDL09	RAM1	-2.8	0.8
YIL072	HOP1	-3.9	0.5	YJL13	MRS3	-2.8	0.8
YBL01	RRN6	-3.9	0.8	YPR03	ERV2	-2.8	0.8
YLR22	RSA3	-3.9	0.7	YLR41	CDC7	-2.8	0.8
YJL20	PRP21	-3.9	0.7	YMR28	HSH1	-2.8	0.8
YOL11	MSB4	-3.9	0.8	YPL17	CBC2	-2.8	0.8
YDR22	GTB1	-3.9	0.7	YFL03	MOB2	-2.8	0.8
YDR21	-	-3.9	0.9	YER00	YND1	-2.8	0.8
YER09	TRP2	-3.9	0.7	YKL05	ASK1	-2.8	0.8
YLL06	MHT1	-3.9	0.7	YOR25	TUM1	-2.8	0.8
YDR30	PRO1	-3.9	0.8	YPL06	BTS1	-2.8	0.8
YPL04	SGF11	-3.9	0.8	YPR13	SPN1	-2.8	0.8
YDL14	CCT4	-3.9	0.9	YLR15	-	-2.8	0.4
YNL29	CLA4	-3.9	0.8	YDR19	CAB5	-2.8	0.7
YBL11	YRF1-	-3.9	0.5	YDR48	SLD5	-2.8	0.8
YDR54	YRF1-	-3.9	0.5	YCL06	BUD5	-2.8	0.7
YER19	YRF1-	-3.9	0.5	YCR03	BUD5	-2.8	0.7
YFL06	YRF1-	-3.9	0.5	YMR20	-	-2.8	0.8
YGR29	YRF1-	-3.9	0.5	YPR13	RRP9	-2.7	0.7
YHL04	YRF1-	-3.9	0.5	YPR12	YLH4	-2.7	0.8
YHR21	-	-3.9	0.5	YHR13	NSG1	-2.7	0.8
YIL177	YRF1-	-3.9	0.5	YIL064	SEE1	-2.7	0.8
YJL225	YRF1-	-3.9	0.5	YIL020	HIS6	-2.7	0.7
YLR46	-	-3.9	0.5	YPL15	PRP4	-2.7	0.8
YLR46	YRF1-	-3.9	0.5	YMR13	RRB1	-2.7	0.8

YML13	-	-3.9	0.5	YGR0	POP	-2.7	0.7
YNL33	YRF1-	-3.9	0.5	YOL0	SIL1	-2.7	0.8
YPL28	YRF1-	-3.9	0.5	YHL02	WSC	-2.7	0.5
YPR2	-	-3.9	0.5	YBR2	YPT1	-2.7	0.8
YLR36	NMD4	-3.9	0.8	YPL08	MOT	-2.7	0.8
YIL127	RRT14	-3.9	0.6	YHR1	CTF8	-2.7	0.8
YMR1	CIN4	-3.9	0.7	YDR1	ENT5	-2.7	0.8
YMR1	SSO2	-3.8	0.7	YPL13	UME	-2.7	0.8
YGL1	-	-3.8	0.8	YHR0	RRP	-2.7	0.8
YMR0	NPL6	-3.8	0.8	YPL12	HHO	-2.7	0.8
YOL0	IZH2	-3.8	0.8	YMR1	GAT	-2.7	0.7
YPR1	JIP5	-3.8	0.8	YOR1	MTR	-2.7	0.8
YPL27	SAM3	-3.8	0.7	YDL23	PHO	-2.7	0.8
YGR2	SER2	-3.8	0.8	YMR2	ROY	-2.7	0.8
YPR1	MMS1	-3.8	0.6	YGR0	PEX3	-2.7	0.8
YKL03	TTI1	-3.8	0.8	YGL0	GEP	-2.7	0.7
YHR0	SSF1	-3.8	0.6	YNR0	PPG1	-2.7	0.8
YER1	GLE2	-3.8	0.8	YPR1	RPC	-2.7	0.8
YMR2	-	-3.8	0.6	YOR1	OST2	-2.7	0.8
YOR3	PRT1	-3.8	0.8	YJL08	ARG	-2.7	0.8
YML12	ERG1	-3.8	0.8	YLR33	SGD	-2.7	0.7
YGR0	GCD2	-3.8	0.8	YLR32	-	-2.7	0.8
YPR0	HTS1	-3.8	0.8	YKR0	RPF2	-2.7	0.8
YDL16	CDC3	-3.8	0.8	YMR1	INP2	-2.7	0.8
YJR10	ECM2	-3.8	0.8	YDR4	RMT	-2.7	0.8
YNL0	-	-3.8	0.6	YBL04	PSY4	-2.7	0.8
YHR1	IKI1	-3.8	0.8	YJR04	TAH1	-2.7	0.8
YNR0	DBP6	-3.8	0.8	YGL0	ERP	-2.7	0.8
YNR0	-	-3.8	0.6	YLR40	DUS	-2.7	0.7
YMR2	TMA2	-3.8	0.6	YIL091	UTP2	-2.7	0.8
YCL03	STE50	-3.8	0.8	YAR0	NUP	-2.7	0.8
YPR0	SMK1	-3.8	0.7	YKL01	RAM	-2.7	0.8
YGL2	MCM6	-3.8	0.8	YER0	PAC	-2.7	0.7
YOR0	TSR3	-3.8	0.7	YDR1	ARG	-2.7	0.8
YLR41	BER1	-3.8	0.8	YJL20	LAA1	-2.7	0.8
YBL00	-	-3.8	0.7	YKR0	MRP	-2.7	0.8
YBL00	-	-3.8	0.7	YPL22	ALG5	-2.7	0.8
YDR1	-	-3.8	0.7	YKL18	HYM	-2.7	0.8
YMR0	-	-3.8	0.7	YPL19	PRM	-2.7	0.6
YMR0	-	-3.8	0.7	YGL24	BRR	-2.7	0.8
YNL28	-	-3.8	0.7	YML0	VPS7	-2.6	0.8
YNL28	-	-3.8	0.7	YDR2	MNN	-2.6	0.8
YJR04	POL32	-3.8	0.8	YPL23	RVB	-2.6	0.8
YML01	-	-3.8	0.8	YNL31	PFS2	-2.6	0.8
YGR0	-	-3.8	0.8	YOL06	APM	-2.6	0.8
YOL0	-	-3.8	0.8	YBR2	ARO	-2.6	0.8
YLL03	GRC3	-3.8	0.7	YHR0	PUT2	-2.6	0.8
YDR3	UTP4	-3.8	0.7	YBL00	LDB7	-2.6	0.8
YGR0	ADE6	-3.8	0.8	YML0	RAD	-2.6	0.8
YEL03	ECM1	-3.8	0.7	YJL21	PEX2	-2.6	0.8
YGR0	CAX4	-3.7	0.7	YMR2	SAP3	-2.6	0.8
YMR1	NDE1	-3.7	0.6	YDL08	LUC7	-2.6	0.8
YGR0	SMD1	-3.7	0.8	YDL00	RMD	-2.6	0.8
YOR1	SLP1	-3.7	0.8	YDR0	RRG	-2.6	0.8
YNL10	-	-3.7	0.8	YNL08	EOS1	-2.6	0.8
YNL16	RIA1	-3.7	0.8	YOR0	-	-2.6	0.8
YKR0	TRZ1	-3.7	0.8	YOR0	HIR2	-2.6	0.8
YGR1	ATF2	-3.7	0.7	YDR4	UTP6	-2.6	0.7
YER1	RAD2	-3.7	0.8	YFR04	RMD	-2.6	0.7
YMR2	RIT1	-3.7	0.8	YBL08	ALG3	-2.6	0.8
YIL172	FSP2	-3.7	0.8	YBL08	ALG3	-2.6	0.8
YJL22	FSP2	-3.7	0.8	YJL05	-	-2.6	0.4
YOL1	FSP2	-3.7	0.8	YOR3	TYE7	-2.6	0.7
YML11	DAT1	-3.7	0.7	YMR1	MUB	-2.6	0.8
YAL05	ECM1	-3.7	0.6	YPL13	RDS	-2.6	0.8
YDR3	CDC4	-3.7	0.7	YPL12	SPC	-2.6	0.8
YCR0	FEN1	-3.7	0.8	YLL03	PRP1	-2.6	0.8
YMR2	HSH1	-3.7	0.8	YHR2	PPX1	-2.6	0.8
YBR2	RRT2	-3.7	0.9	YKR0	BET3	-2.6	0.8
YPR0	SRP5	-3.7	0.8	YDL15	RPC	-2.6	0.7
YOR2	NOC2	-3.7	0.7	YGL1	NUP	-2.6	0.8

YPL21	-	-3.7	0.7	YHR03	RRM3	-2.6	0.8
YPL14	PXA1	-3.7	0.6	YPR05	BRR1	-2.6	0.8
YJL05(	ZAP1	-3.7	0.7	YGL25	VEL1	-2.6	0.7
YGL07	DBP3	-3.7	0.7	YJR13	TTI2	-2.6	0.8
YLL05	-	-3.7	0.7	YMR2	-	-2.6	0.7
YNR04	TRM11	-3.7	0.8	YDR17	HMO	-2.6	0.8
YBL11	YRF1-	-3.7	0.3	YGR09	RRP4	-2.6	0.8
YBL11	YRF1-	-3.7	0.3	YPL21	NIP7	-2.6	0.8
YDR54	YRF1-	-3.7	0.3	YBL03	URA7	-2.6	0.8
YEL07	YRF1-	-3.7	0.3	YMR22	MTF1	-2.6	0.8
YER19	YRF1-	-3.7	0.3	YML09	VPS9	-2.5	0.8
YGR29	YRF1-	-3.7	0.3	YBL02	-	-2.5	0.8
YHR21	YRF1-	-3.7	0.3	YPL09	NOG1	-2.5	0.8
YHR21	YRF1-	-3.7	0.3	YGL02	PGD1	-2.5	0.8
YIL177	YRF1-	-3.7	0.3	YAR07	IMD1	-2.5	0.7
YJL22	YRF1-	-3.7	0.3	YHR21	IMD2	-2.5	0.7
YLL06	YRF1-	-3.7	0.3	YOR02	CIN5	-2.5	0.8
YLL06	YRF1-	-3.7	0.3	YMR03	ARP9	-2.5	0.8
YLR46	YRF1-	-3.7	0.3	YBL02	RRN1	-2.5	0.8
YLR46	YRF1-	-3.7	0.3	YGL16	SUA5	-2.5	0.8
YML13	-	-3.7	0.3	YBL05	TOD6	-2.5	0.7
YNL33	YRF1-	-3.7	0.3	YML04	RRN1	-2.5	0.7
YOR39	YRF1-	-3.7	0.3	YOR22	ODC2	-2.5	0.7
YPL28	YRF1-	-3.7	0.3	YNL07	RNH2	-2.5	0.8
YPR20	-	-3.7	0.3	YDR39	SHE9	-2.5	0.8
YNL22	POP1	-3.7	0.8	YNL25	MRPI	-2.5	0.8
YGL01	ERG4	-3.7	0.8	YNL28	MRPI	-2.5	0.8
YHR04	BCD1	-3.7	0.7	YHR12	MSH1	-2.5	0.8
YPL09	SEC62	-3.6	0.9	YLR09	ICT1	-2.5	0.8
YCR08	-	-3.6	0.8	YNR01	SMM	-2.5	0.8
YML01	SPT5	-3.6	0.8	YKR02	BCH2	-2.5	0.8
YJR01	TMA22	-3.6	0.9	YOL15	HPF1	-2.5	0.6
YKR02	BCH2	-3.6	0.7	YNR04	TRM1	-2.5	0.8
YLL01	YEH1	-3.6	0.7	YOR34	RPA4	-2.5	0.8
YMR1	FOL3	-3.6	0.8	YEL03	MCM	-2.5	0.8
YJR13	MCM2	-3.6	0.8	YIR02	MRS1	-2.5	0.8
YJR13	MNS1	-3.6	0.8	YOR24	MET7	-2.5	0.8
YNL04	SFB2	-3.6	0.8	YBR12	TFC1	-2.5	0.8
YER14	-	-3.6	0.8	YLR08	EMP7	-2.5	0.8
YNL07	RNH20	-3.6	0.7	YGR12	-	-2.5	0.8
YCL00	-	-3.6	0.8	YPL24	HUT1	-2.5	0.8
YEL07	RMD6	-3.6	0.8	YMR23	RNT1	-2.5	0.7
YHR06	RRP4	-3.6	0.9	YNL26	ORC5	-2.5	0.8
YKL19	PEX1	-3.6	0.7	YDR50	LPP1	-2.5	0.7
YGL02	PIB2	-3.6	0.8	YOR12	AFI1	-2.5	0.8
YGL01	CKB1	-3.6	0.8	YBR04	CST2	-2.5	0.8
YGR09	PRP31	-3.6	0.8	YGR12	UTP8	-2.5	0.8
YGL13	CEG1	-3.6	0.8	YDR49	PUF6	-2.5	0.7
YMR23	RNH1	-3.6	0.8	YAR00	SWD	-2.5	0.8
YLR18	MDL1	-3.6	0.8	YBR22	-	-2.5	0.8
YPL05	KTR6	-3.5	0.8	YDR24	-	-2.5	0.8
YOL05	-	-3.5	0.9	YJL07	PSF2	-2.5	0.8
YBR25	POP4	-3.5	0.7	YNL12	NAF1	-2.5	0.8
YDR19	RVB1	-3.5	0.9	YGL21	KIP3	-2.5	0.8
YLR13	NHA1	-3.5	0.8	YOR13	-	-2.5	0.8
YPL14	FRK1	-3.5	0.8	YNL03	GPI15	-2.5	0.8
YML04	RRN11	-3.5	0.6	YGL24	RAI1	-2.5	0.8
YLR18	VTA1	-3.5	0.8	YOL13	CDC3	-2.5	0.9
YOR36	PIP2	-3.5	0.7	YML08	TDA9	-2.5	0.8
YIL078	THS1	-3.5	0.8	YBR25	DUT1	-2.5	0.8
YDR02	SES1	-3.5	0.8	YOR20	DED1	-2.5	0.8
YNR06	-	-3.5	0.8	YPL04	SSN3	-2.5	0.8
YOR06	CKA2	-3.5	0.8	YLR24	ARV1	-2.5	0.8
YIL158	AIM20	-3.5	0.7	YBL00	HTB2	-2.5	0.8
YNL02	-	-3.5	0.6	YCR05	PWP1	-2.5	0.8
YJR04	TAH11	-3.5	0.9	YNL29	CLA4	-2.5	0.8
YDR31	SSF2	-3.5	0.7	YLL01	SOF1	-2.4	0.8
YOR00	RRP6	-3.5	0.7	YGR22	TOS2	-2.4	0.8
YCL03	RRP7	-3.5	0.8	YPL12	TFB2	-2.4	0.8
YML07	FPR3	-3.5	0.8	YCR02	HTL1	-2.4	0.7
YNL04	BOP3	-3.5	0.8	YDR26	CIA1	-2.4	0.8

YGL21	SKI8	-3.5	0.8	YGL22	SDT1	-2.4	0.8
YDR4	DOT1	-3.5	0.8	YNL24	RPA	-2.4	0.8
YJL18	MNN1	-3.5	0.8	YCR0	-	-2.4	0.7
YMR0	BUD2	-3.5	0.6	YNR0	SNF1	-2.4	0.8
YGR2	FOL2	-3.5	0.9	YHR2	IMD2	-2.4	0.7
YNL12	NAF1	-3.5	0.7	YDR3	-	-2.4	0.8
YNR0	PDR1	-3.5	0.4	YDR3	ASP1	-2.4	0.8
YDR23	FMN1	-3.5	0.8	YER02	RPN	-2.4	0.8
YJL18	GON7	-3.5	0.8	YPL20	IPL1	-2.4	0.8
YGR2	ZUO1	-3.4	0.8	YOL13	HRT1	-2.4	0.8
YGR1	ASN2	-3.4	0.8	YMR3	PSE1	-2.4	0.8
YGR1	GTO1	-3.4	0.8	YOR3	RAX1	-2.4	0.8
YGL25	VEL1	-3.4	0.7	YMR2	CEF1	-2.4	0.8
YOR1	BFR1	-3.4	0.8	YDR4	RRP1	-2.4	0.8
YHR0	SSZ1	-3.4	0.8	YOR1	PNO	-2.4	0.8
YGL17	SAE2	-3.4	0.7	YIL08	SDS	-2.4	0.8
YBL00	UTP2	-3.4	0.8	YNL22	SSU	-2.4	0.8
YDL14	-	-3.4	0.8	YML0	PIF1	-2.4	0.8
YLR22	CDC4	-3.4	0.9	YJR10	ABM	-2.4	0.8
YNR0	AGA1	-3.4	0.5	YJL11	PHO	-2.4	0.8
YAL00	SPO7	-3.4	0.8	YBL02	HAP	-2.4	0.8
YBR2	MAL3	-3.4	0.4	YKL19	DPH	-2.4	0.8
YGR2	MAL1	-3.4	0.4	YJR08	CSN1	-2.4	0.9
YLR28	-	-3.4	0.7	YMR0	AVO	-2.4	0.7
YER07	-	-3.4	0.6	YNL06	MTQ	-2.4	0.8
YJR01	MET3	-3.4	0.7	YDR1	HOM	-2.4	0.8
YMR1	-	-3.4	0.8	YMR2	TAF9	-2.4	0.8
YIL021	RPB3	-3.4	0.8	YGL01	KAP1	-2.4	0.9
YJR13	ECM1	-3.4	0.7	YNR0	PDR1	-2.4	0.5
YEL02	SNU1	-3.4	0.9	YBL08	-	-2.4	0.7
YIL15	MCM1	-3.4	0.8	YLR28	-	-2.4	0.7
YJL02	RRN7	-3.4	0.8	YML0	AIM3	-2.4	0.3
YPL06	BTS1	-3.4	0.8	YDL01	NOP	-2.4	0.8
YOR1	RGA1	-3.4	0.8	YBR1	CDC	-2.4	0.8
YFR0	SAD1	-3.4	0.8	YML01	-	-2.4	0.8
YDR0	MAK2	-3.4	0.8	YCR1	ADH	-2.4	0.8
YDR2	EBS1	-3.4	0.7	YBR1	PEX3	-2.3	0.8
				YPL21	PUS1	-2.3	0.8
				YHR0	-	-2.3	0.8
				YOR1	SLP1	-2.3	0.8
				YJR09	-	-2.3	0.8
				YPL16	-	-2.3	0.8
				YNL23	KEX2	-2.3	0.8
				YOL0	RFC	-2.3	0.8
				YFR04	KEG1	-2.3	0.8
				YCL00	RER1	-2.3	0.8
				YER02	GCD	-2.3	0.9
				YOR3	SFG1	-2.3	0.8
				YKL13	OCT1	-2.3	0.8
				YJL20	ACO	-2.3	0.8
				YDR31	MCM	-2.3	0.8
				YMR1	EAR1	-2.3	0.8
				YLR00	PAM	-2.3	0.8
				YMR2	UBP	-2.3	0.8
				YCL05	KRR1	-2.3	0.8
				YLR43	IMD3	-2.3	0.8
				YOL0	HST1	-2.3	0.8
				YFR0	PHO	-2.3	0.8
				YDL13	SRF1	-2.3	0.8
				YKL01	ATP7	-2.3	0.8
				YOL0	TRM	-2.3	0.8
				YOR2	TMA1	-2.3	0.8
				YBR2	TSC1	-2.3	0.8
				YJR07	MIR1	-2.3	0.8
				YLR17	CBF5	-2.3	0.8
				YBL02	LSM	-2.3	0.9
				YIL16	-	-2.3	0.6
				YOL1	HPF1	-2.3	0.6
				YOR1	LIP5	-2.3	0.8
				YNL31	RFA2	-2.3	0.9
				YOR1	RIO1	-2.3	0.7

YJL18(	MNN!	-2.3	0.8
YJL19	PHO!	-2.3	0.8
YNL04	ALG1	-2.3	0.8
YEL04	TCA1	-2.3	0.8
YJR11	-	-2.3	0.8
YER05	-	-2.3	0.5
YDR18	-	-2.3	0.7
YDR25	CTA1	-2.3	0.5
YGL14	ROG:	-2.3	0.7
YLR24	MAP:	-2.3	0.8
YPR1(	YTH1	-2.3	0.8
YBL05	PTH2	-2.3	0.8
YBR23	OM14	-2.3	0.8
YOL15	ZPS1	-2.3	0.9
YCL05	FYV5	-2.3	0.7
YDR5(	PLM2	-2.3	0.8
YBR15	RPB5	-2.3	0.8
YDR07	PPH3	-2.3	0.8
YHR2(	SCH!	-2.3	0.8
YOR24	ESA1	-2.3	0.9
YNL17	NOP:	-2.3	0.8
YML03	-	-2.3	0.4
YGL1(	SEH1	-2.2	0.8
YPR05	NHP6	-2.2	0.8
YPL07	GPI2	-2.2	0.7
YLR43	TSR2	-2.2	0.8
YPL26	KAR!	-2.2	0.8
YOR2	ISU2	-2.2	0.9
YMR2(	PRP2	-2.2	0.8
YNL27	BOR:	-2.2	0.8
YOL02	TSR4	-2.2	0.8
YNL06	YDJ1	-2.2	0.9
YGR07	SMD:	-2.2	0.9
YGL17	ROK:	-2.2	0.8
YDR19	HST4	-2.2	0.8
YNL32	LEM3	-2.2	0.8
YGR0(	MUQ	-2.2	0.8
YER07	ICP5!	-2.2	0.8
