# Supplementary material for: Environmental Stresses Disrupt Telomere Length Homeostasis
Source: PLoS Genet. 2013 Sep 5;9(9):e1003721. doi: 10.1371/journal.pgen.1003721 (PMC3764183; doi:10.1371/journal.pgen.1003721)
Supplement: Table S3 — List of genes whose expression changed upon growth on stressing conditions. TLM genes are marked “1”, non-TLM genes “0”. Increased expression upon stress is denoted “1”, reduced expression appears as “−1”. (PDF) [file pgen.1003721.s008.pdf]

Is TLM	Gene name	Systematic name	Caffeine	37°C	Ethanol
0	AAC1	YMR056C	1	-	1
0	AAC3	YBR085W	-	-	-1
0	AAD10	YJR155W	1	1	1
0	AAD15	YOL165C	1	-1	-
0	AAD16	YFL057C	1	-	1
0	AAD3	YCR107W	1	-1	-
0	AAD4	YDL243C	1	-	1
0	AAD6	YFL056C	-	-	1
0	AAH1	YNL141W	-1	-	-1
0	AAT1	YKL106W	-1	-	-1
0	ABC1	YGL119W	-	1	-
0	ABD1	YBR236C	1	-	-
0	ABF2	YMR072W	-	-1	-
0	ABM1	YJR108W	-1	-	-
0	ABP1	YCR088W	1	-	-
0	ABP140	YOR239W	-	-1	-
0	ABZ2	YMR289W	-	-	-1
0	ACA1	YER045C	1	-	-
0	ACB1	YGR037C	-	-	-1
0	ACF2	YLR144C	-	1	-
0	ACH1	YBL015W	1	-	-
0	ACM1	YPL267W	-1	-	-
0	ACN9	YDR511W	-	1	1
0	ACO1	YLR304C	1	1	-
0	ACO2	YJL200C	-1	-	-1
0	ACP1	YKL192C	-	1	-
0	ACS1	YAL054C	1	1	-
0	ADD66	YKL206C	-1	-	-
0	ADE1	YAR015W	1	-1	-
1	**ADE12**	**YNL220W**	1	-	-1
0	ADE13	YLR359W	1	-1	-1
0	ADE16	YLR028C	1	-	-
0	ADE17	YMR120C	1	-	1
0	ADE2	YOR128C	1	-	-
0	ADE3	YGR204W	1	-	-
0	ADE4	YMR300C	1	-	-
0	ADE5,7	YGL234W	1	-	-1
0	ADE6	YGR061C	1	-1	-1
0	ADE8	YDR408C	1	-	-
0	ADH2	YMR303C	1	1	-
0	ADH3	YMR083W	1	-	-
0	ADH4	YGL256W	-1	-1	-
0	ADH5	YBR145W	-	-	1
0	ADH6	YMR318C	-	1	-1
0	ADH7	YCR105W	-1	-	-
0	ADI1	YMR009W	-	-	-1
1	**ADO1**	**YJR105W**	-	-	-1
0	ADR1	YDR216W	1	1	1
0	AFG2	YLR397C	-1	-	-1

0	AFG3	YER017C	-	-	1
0	AFI1	YOR129C	-1	-	-
0	AFR1	YDR085C	1	-	-
0	AGA1	YNR044W	-1	-1	-1
0	AGA2	YGL032C	-1	-1	-
0	AGE1	YDR524C	-	-1	-
1	**AGP2**	**YBR132C**	1	-	-
0	AGX1	YFL030W	-	1	1
0	AHA1	YDR214W	-	1	-
1	**AHC2**	**YCR082W**	-	1	-
0	AIF1	YNR074C	-	-1	-
0	AIM11	YER093C-A	-	1	1
0	AIM13	YFR011C	-	1	1
0	AIM17	YHL021C	1	1	1
0	AIM18	YHR198C	-	1	1
0	AIM19	YIL087C	1	-	1
0	AIM2	YAL049C	-1	-	-
0	AIM20	YIL158W	-	-1	-1
0	AIM21	YIR003W	1	-	-
0	AIM23	YJL131C	1	-	1
1	**AIM24**	**YJR080C**	-	-	1
0	AIM3	YBR108W	-	1	-
0	AIM32	YML050W	-	1	1
0	AIM33	YML087C	-1	-	-
0	AIM36	YMR157C	-	1	-
0	AIM37	YNL100W	-1	-	-
0	AIM39	YOL053W	1	-	1
0	AIM4	YBR194W	-	1	-
0	AIM41	YOR215C	1	-1	1
0	AIM45	YPR004C	1	1	1
0	AIM46	YHR199C	1	1	-
0	AIM5	YBR262C	-	1	1
0	AIM7	YDR063W	-	-	1
0	AIR1	YIL079C	-	-	-1
0	AIR2	YDL175C	-	-	1
0	AKL1	YBR059C	1	-	1
0	AKR1	YDR264C	1	-	-
0	AKR2	YOR034C	1	-	-
1	**ALA1**	**YOR335C**	-	-1	-1
0	ALB1	YJL122W	-	-	-1
0	ALD2	YMR170C	-	1	-
0	ALD3	YMR169C	-	1	1
0	ALD4	YOR374W	1	1	1
0	ALD5	YER073W	-1	-1	-
0	ALD6	YPL061W	-	-	-1
0	ALF1	YNL148C	1	-1	-1
0	ALG11	YNL048W	-1	-	-
0	ALG3	YBL082C	-1	-1	-
0	ALG3	YBL083C	-1	-1	-
0	ALG5	YPL227C	-1	-	-

0	ALK1	YGL021W	1	-	-
0	ALP1	YNL270C	1	1	1
0	ALR1	YOL130W	-	-	-1
0	ALT1	YLR089C	1	-	1
0	ALT2	YDR111C	-1	-1	-1
0	AMD1	YML035C	-	1	-
0	AMD2	YDR242W	1	-	-
0	AME1	YBR211C	-	-1	-
0	AMN1	YBR158W	-1	-	-
0	AMS1	YGL156W	1	1	1
0	ANB1	YJR047C	1	-	-
0	ANT1	YPR128C	-1	-1	-1
0	APA2	YDR530C	-	-1	1
1	**APC4**	**YDR118W**	-1	-1	-
0	APE2	YKL157W	1	-	1
0	APJ1	YNL077W	1	-	-
0	APL1	YJR005W	1	-	-
0	APL6	YGR261C	1	-	-
0	APM3	YBR288C	-	-1	-
0	APM4	YOL062C	-1	-	-1
0	APP1	YNL094W	1	-	-
0	APQ12	YIL040W	-1	-	1
0	APS1	YLR170C	1	-	-
0	APT1	YML022W	-1	-	-1
0	APT2	YDR441C	-1	-	-
0	AQR1	YNL065W	-	1	-
0	AQY2	YLL052C	1	-	-1
0	ARA1	YBR149W	1	1	-
0	ARB1	YER036C	-	-	-1
0	ARC19	YKL013C	1	-	-
0	ARE1	YCR048W	1	1	-
0	ARE2	YNR019W	-	1	-
0	ARF2	YDL137W	1	-	1
0	ARG1	YOL058W	-	-	1
1	**ARG2**	**YJL071W**	-	1	-
0	ARG3	YJL088W	-1	1	1
0	ARG4	YHR018C	-	-	1
0	ARG5,6	YER069W	-	1	-
0	ARG8	YOL140W	-1	1	-
0	ARG80	YMR042W	1	-	-
1	**ARG82**	**YDR173C**	-1	-	-
0	ARI1	YGL157W	-	-	-1
0	ARK1	YNL020C	-	1	-
0	ARL1	YBR164C	-	-	-1
0	ARL3	YPL051W	-	-1	-
0	ARN1	YHL040C	1	-	-
0	ARN2	YHL047C	1	-1	-
0	ARO1	YDR127W	1	-	-
0	ARO10	YDR380W	1	1	1
0	ARO2	YGL148W	-	-	-1

0	ARO3	YDR035W	1	-1	-
0	ARO4	YBR249C	-1	-1	-
0	ARO7	YPR060C	-1	-	-1
0	ARO80	YDR421W	-	1	-
0	ARO9	YHR137W	1	1	1
0	ARP1	YHR129C	-	-	-1
1	**ARP2**	**YDL029W**	1	-	-
0	ARP4	YJL081C	-	-1	-1
0	ARP5	YNL059C	1	-	-
0	ARP9	YMR033W	-1	-1	-
0	ARR1	YPR199C	-1	-	-
0	ARR2	YPR200C	-	-	1
0	ART10	YLR392C	-	-	1
1	**ARV1**	**YLR242C**	-1	-	-
0	ARX1	YDR101C	-1	-	-1
0	ASA1	YPR085C	-	-	-1
0	ASG7	YJL170C	-1	-	-
0	ASK1	YKL052C	-1	-	-
0	ASK10	YGR097W	1	-	-1
0	ASN1	YPR145W	1	-1	-
0	ASN2	YGR124W	-	-	-1
0	ASP1	YDR321W	-1	-	-1
0	ASP3-1	YLR155C	-	-1	-
0	ASP3-2	YLR157C	-	-1	-
0	ASP3-3	YLR158C	-	-1	-
0	ASP3-4	YLR160C	-	-1	-
0	AST1	YBL069W	-1	-	-
1	**ATC1**	**YDR184C**	-1	-	-1
0	ATE1	YGL017W	-1	-	-
0	ATF1	YOR377W	-1	-1	-1
0	ATF2	YGR177C	1	-	-1
0	ATG1	YGL180W	-	1	-
0	ATG10	YLL042C	-1	-1	-
1	**ATG11**	**YPR049C**	-	1	-
0	ATG13	YPR185W	1	-	-
0	ATG14	YBR128C	-	-	1
1	**ATG17**	**YLR423C**	-	-	1
0	ATG18	YFR021W	-	1	-
0	ATG19	YOL082W	1	-	1
0	ATG2	YNL242W	-	1	1
0	ATG20	YDL113C	1	-	-
0	ATG22	YCL038C	1	1	1
0	ATG23	YLR431C	1	-	-
0	ATG26	YLR189C	1	1	-
0	ATG27	YJL178C	-1	-1	-
0	ATG29	YPL166W	1	-	-
0	ATG33	YLR356W	1	-	1
0	ATG34	YOL083W	1	1	1
0	ATG4	YNL223W	-	-	1
0	ATG8	YBL078C	1	-	1

0	ATG9	YDL149W	-	1	1
0	ATH1	YPR026W	1	1	1
1	**ATM1**	**YMR301C**	-	-	-1
0	ATO2	YNR002C	1	1	-
0	ATO3	YDR384C	-	-1	-1
0	ATP10	YLR393W	-	1	-
0	ATP12	YJL180C	-	-	1
0	ATP14	YLR295C	-	-	1
0	ATP15	YPL271W	-	-	1
0	ATP17	YDR377W	-	-	1
0	ATP18	YML081C-A	-	-	1
0	ATP20	YPR020W	-	-	1
0	ATP25	YMR098C	-	1	-
0	ATP3	YBR039W	-	-	1
0	ATP4	YPL078C	-	-	1
0	ATP5	YDR298C	-	-1	1
0	ATP7	YKL016C	-1	-	1
0	ATR1	YML116W	-	-	1
0	ATX2	YOR079C	-	-1	-1
0	AUR1	YKL004W	-	-	-1
0	AUS1	YOR011W	1	-1	-1
0	AVL9	YLR114C	1	-	-
0	AVO2	YMR068W	-1	-	-
0	AVT6	YER119C	-	1	-
0	AVT7	YIL088C	-	-	1
0	AXL1	YPR122W	-1	-	1
0	AYR1	YIL124W	-	-	1
0	AYT1	YLL063C	1	-1	-
0	AZF1	YOR113W	-	1	-
0	BAP2	YBR068C	-1	-	-
0	BAP3	YDR046C	-1	-	-
0	BAR1	YIL015W	-1	-1	-
0	BAS1	YKR099W	-	-	-1
0	BAT1	YHR208W	-1	-1	-
0	BAT2	YJR148W	1	-	-
0	BBC1	YJL020C	1	1	1
1	**BBP1**	**YPL255W**	-	-1	-
0	BCD1	YHR040W	-	-	-1
0	BCH1	YMR237W	-	1	-
0	BCH2	YKR027W	-1	-	-1
0	BCP1	YDR361C	-	-	-1
0	BDH1	YAL060W	1	1	-
0	BDH2	YAL061W	-	1	-
0	BDS1	YOL164W	-	-1	-1
0	BER1	YLR412W	-1	-1	-1
0	BET1	YIL004C	-	-	-1
0	BET3	YKR068C	-1	-	-
0	BET5	YML077W	-	-	-1
0	BFR1	YOR198C	-	-1	-1
0	BFR2	YDR299W	-	-	-1

0	BGL2	YGR282C	1	-	-
0	BIM1	YER016W	-	-1	-
0	BIO2	YGR286C	-	-	-1
0	BIO3	YNR058W	1	-	-1
0	BIO4	YNR057C	-1	-	-
0	BIT61	YJL058C	-	1	-
0	BLI1	YKL061W	1	-	-
0	BLS1	YLR408C	-	-	1
1	**BMH2**	**YDR099W**	1	-	-
0	BNA1	YJR025C	-	1	1
0	BNA2	YJR078W	1	1	1
0	BNA4	YBL098W	1	1	1
0	BNA6	YFR047C	-	1	-
0	BNA7	YDR428C	-	-	-1
0	BNI1	YNL271C	1	-	-
0	BNR1	YIL159W	-	1	-
0	BOP2	YLR267W	1	1	-
0	BOP3	YNL042W	-	-	-1
0	BOR1	YNL275W	-1	-	-
0	BOS1	YLR078C	-1	-	-
0	BPH1	YCR032W	1	-	-
1	**BRE2**	**YLR015W**	-	-	-1
0	BRE5	YNR051C	-	-1	-
0	BRF1	YGR246C	-	-1	-
0	BRL1	YHR036W	-1	-	-
0	BRR1	YPR057W	-1	-1	-
0	BRR6	YGL247W	-1	-	-
0	BRX1	YOL077C	-1	-1	-1
0	BSC2	YDR275W	-	-	1
0	BTN2	YGR142W	-1	-	-
0	BTS1	YPL069C	-1	-	-1
1	**BUD21**	**YOR078W**	-	-	-1
0	BUD22	YMR014W	-	-	-1
1	**BUD23**	**YCR047C**	-1	-	-1
0	BUD25	YER014C-A	1	-	-
0	BUD4	YJR092W	1	-	-
0	BUD5	YCL068C	-1	-	-
0	BUD5	YCR038C	-1	-	-
0	BUD8	YLR353W	-1	-	1
0	BUL1	YMR275C	1	-	-
0	BUR6	YER159C	1	-	1
0	BXI1	YNL305C	1	1	1
0	CAB1	YDR531W	-	-	-1
0	CAB2	YIL083C	1	-	-
0	CAB5	YDR196C	-1	-	-
0	CAC2	YML102W	-1	-	-
0	CAF120	YNL278W	1	-	-
0	CAF17	YJR122W	-	1	1
0	CAF20	YOR276W	-1	-	-
0	CAF40	YNL288W	-1	-	-

0	CAJ1	YER048C	-	-	-1
0	CAR1	YPL111W	-	-	-1
0	CAR2	YLR438W	1	1	-
0	CAT2	YML042W	-	1	-
0	CAT5	YOR125C	-	-	1
1	**CAX4**	**YGR036C**	-	-	-1
1	**CBC2**	**YPL178W**	-1	-1	-
0	CBF1	YJR060W	-1	-	-
1	**CBF5**	**YLR175W**	-1	-1	-1
0	CBK1	YNL161W	-	1	-
0	CBP4	YGR174C	-	1	1
0	CBP6	YBR120C	-	-	1
0	CCM1	YGR150C	-	1	-
0	CCP1	YKR066C	-	1	-
0	CCT2	YIL142W	-	-	-1
0	CCT3	YJL014W	-1	-1	-1
0	CCT4	YDL143W	-	-	-1
0	CCT5	YJR064W	-	-	-1
0	CCT7	YJL111W	-	-1	-1
0	CCT8	YJL008C	-	-1	-1
1	**CCW14**	**YLR390W-A**	1	-	-
0	CDA1	YLR307W	-	1	-
0	CDC12	YHR107C	-	-1	-
0	CDC26	YFR036W	-	1	-
0	CDC28	YBR160W	-1	1	-
0	CDC33	YOL139C	-1	-1	-1
1	**CDC36**	**YDL165W**	-1	-1	-1
0	CDC4	YFL009W	-	1	-
0	CDC40	YDR364C	-	-	-1
0	CDC42	YLR229C	-	-1	-1
0	CDC43	YGL155W	-1	-1	-
0	CDC5	YMR001C	-	-1	-
0	CDC55	YGL190C	-1	-	-
0	CDC7	YDL017W	-	-	-1
1	**CDC73**	**YLR418C**	-1	-1	-
0	CDD1	YLR245C	-	1	-
0	CEF1	YMR213W	-1	-	-
0	CEG1	YGL130W	-	-	-1
0	CEM1	YER061C	1	1	1
0	CFD1	YIL003W	-	-	-1
0	CGR1	YGL029W	-	-	-1
0	CHC1	YGL206C	1	1	-
0	CHD1	YER164W	-1	-	-
0	CHL4	YDR254W	-	-1	-
0	CHS1	YNL192W	1	-	-
0	CHS6	YJL099W	1	-	-1
0	CHZ1	YER030W	1	-	-
0	CIA1	YDR267C	-1	-1	-1
0	CIC1	YHR052W	-1	-	-1
0	CIN1	YOR349W	-	1	-

0	CIN2	YPL241C	-	-1	-
0	CIN4	YMR138W	-	-1	-1
0	CIN5	YOR028C	-1	-	-1
0	CIR1	YGR207C	1	1	1
0	CIR2	YOR356W	-1	-	-
0	CIT1	YNR001C	1	1	1
0	CIT2	YCR005C	1	-	1
0	CIT3	YPR001W	1	1	1
0	CKA2	YOR061W	-	-	-1
0	CKB1	YGL019W	-1	-	-1
0	CKB2	YOR039W	-	-1	-1
0	CLA4	YNL298W	-1	-	-1
0	CLB1	YGR108W	-1	-	1
0	CLB3	YDL155W	-1	-	-
0	CLB4	YLR210W	-1	-	-
0	CLB5	YPR120C	-1	-	-
0	CLB6	YGR109C	-1	-	-
0	CLC1	YGR167W	1	-	-
0	CLD1	YGR110W	-	1	1
0	CLG1	YGL215W	1	-	-
0	CLN1	YMR199W	-	-	1
0	CLN2	YPL256C	-	-	1
0	CLP1	YOR250C	-1	-	-
0	CMC2	YBL059C-A	-	1	1
0	CMC4	YMR194C-B	1	1	1
0	CMK2	YOL016C	1	1	1
0	CMP2	YML057W	1	-	-
0	CMS1	YLR003C	-	-1	-1
0	CNL1	YDR357C	1	-	-
0	CNS1	YBR155W	-	-	-1
0	COA1	YIL157C	-	1	1
0	COA2	YPL189C-A	-1	-	-
0	COA3	YJL062W-A	-	1	-
0	COA4	YLR218C	1	1	1
0	COG7	YGL005C	-	-1	-
0	COQ1	YBR003W	-	-	1
0	COQ2	YNR041C	-1	-	-
0	COQ5	YML110C	-	1	1
0	COQ6	YGR255C	1	-	1
0	COQ9	YLR201C	-	-	1
0	COR1	YBL045C	-	-	1
0	COS1	YNL336W	1	-1	1
0	COS12	YGL263W	1	-	-
0	COS2	YBR302C	1	-1	-
0	COS3	YML132W	1	-1	-
0	COS4	YFL062W	1	-1	1
0	COS5	YJR161C	1	-1	-
0	COS6	YGR295C	1	-1	1
0	COS7	YDL248W	1	-1	-
0	COS8	YHL048W	1	-1	1

0	COS9	YKL219W	1	-	-
0	COT1	YOR316C	1	-	-
0	COX12	YLR038C	-	-	1
0	COX13	YGL191W	-	-	1
0	COX14	YML129C	-	1	1
0	COX15	YER141W	1	-	1
0	COX16	YJL003W	-	-	1
0	COX17	YLL009C	-	1	1
0	COX18	YGR062C	-	-	1
0	COX20	YDR231C	1	1	1
0	COX23	YHR116W	-	1	-
0	COX4	YGL187C	-1	-1	1
0	COX5A	YNL052W	-1	-	-
0	COX5B	YIL111W	1	1	1
0	COX6	YHR051W	-	-	1
0	COX7	YMR256C	-	-	1
0	COX8	YLR395C	-1	-	1
0	COX9	YDL067C	-	-	1
0	CPA1	YOR303W	1	1	-
0	CPA2	YJR109C	1	-	1
0	CPR2	YHR057C	-	-1	-
0	CPR4	YCR069W	1	-	-
0	CPR6	YLR216C	-	1	-
0	CPR7	YJR032W	-	-	-1
0	CPR8	YNR028W	-	-1	1
0	CPS1	YJL172W	1	1	-
0	CPT1	YNL130C	-	1	-
0	CRC1	YOR100C	1	1	1
0	CRG1	YHR209W	1	1	-
0	CRH1	YGR189C	1	-	-
0	CRP1	YHR146W	-	-1	-
0	CRR1	YLR213C	1	-	-
0	CRS5	YOR031W	-	1	-
0	CRT10	YOL063C	-	1	-
0	CSH1	YBR161W	-	-	1
0	CSI1	YMR025W	1	-	-
0	CSI2	YOL007C	-1	-	-
0	CSL4	YNL232W	-1	-	-
1	**CSM1**	**YCR086W**	-	1	-
0	CSN12	YJR084W	-1	-	-
0	CSN9	YDR179C	1	-	-
0	CSR1	YLR380W	-	-	-1
0	CST26	YBR042C	-1	-1	-1
1	**CST6**	**YIL036W**	-	1	-
0	CST9	YLR394W	-	1	-
0	CTA1	YDR256C	-1	-	-
1	**CTF18**	**YMR078C**	1	-	-
1	**CTF8**	**YHR191C**	-1	-	-
0	CTK2	YJL006C	-1	-	-
0	CTL1	YMR180C	1	-	-

0	CTP1	YBR291C	-1	-	-1
0	CTR1	YPR124W	1	-	-
0	CTT1	YGR088W	-	1	-
0	CUE2	YKL090W	-1	-	-
0	CUE3	YGL110C	-	-1	-
0	CUE4	YML101C	-	-	1
0	CUE5	YOR042W	1	-	-
0	CUP1-1	YHR053C	-	-	1
0	CUP1-2	YHR055C	-	-	1
0	CUP2	YGL166W	-	1	-
0	CUR1	YPR158W	-	-	1
0	CUS1	YMR240C	-	-1	-
0	CWC2	YDL209C	-	-1	-
0	CWC21	YDR482C	1	-	-1
0	CWC25	YNL245C	-	1	-
0	CWH41	YGL027C	1	-	-
0	CWP1	YKL096W	-	1	-
0	CWP2	YKL096W-A	-	-	1
0	CYB2	YML054C	-	-	-1
0	CYB5	YNL111C	1	-	-
0	CYC1	YJR048W	-1	-	-1
0	CYC3	YAL039C	1	1	1
0	CYC7	YEL039C	1	1	1
0	CYM1	YDR430C	-	1	-
1	**CYR1**	**YJL005W**	1	1	1
0	CYS3	YAL012W	-	-	-1
0	CYS4	YGR155W	-1	-	-
0	CYT1	YOR065W	-1	-	1
0	CYT2	YKL087C	-	1	1
0	DAK1	YML070W	-	1	-
0	DAL1	YIR027C	1	1	-
0	DAL3	YIR032C	1	1	-
0	DAL5	YJR152W	1	-	-
0	DAL7	YIR031C	1	-	-
0	DAN1	YJR150C	1	-	1
0	DAN2	YLR037C	1	-	-
0	DAN3	YBR301W	1	-	-
0	DAN4	YJR151C	1	-	-
0	DAP1	YPL170W	1	-	-
0	DAS1	YJL149W	1	1	-
0	DAS2	YDR020C	-1	-	-1
0	DAT1	YML113W	-	-	-1
0	DBP10	YDL031W	-	-	-1
0	DBP2	YNL112W	-1	-1	-1
0	DBP3	YGL078C	-	-	-1
0	DBP6	YNR038W	-	-	-1
0	DBP7	YKR024C	-	-	-1
0	DBP8	YHR169W	-1	-	-1
0	DBP9	YLR276C	-	-	-1
0	DCP1	YOL149W	-	-1	-

0	DCS1	YLR270W	1	1	1
0	DCS2	YOR173W	1	1	1
0	DCW1	YKL046C	1	-	-
0	DDR2	YOL052C-A	1	1	1
0	DDR48	YMR173W	-	1	1
0	DDR48	YMR173W-A	-	1	1
0	DED1	YOR204W	-1	-	-
0	DEG1	YFL001W	-	-	-1
0	DFG5	YMR238W	1	-	-
0	DFR1	YOR236W	-1	-1	-
0	DGA1	YOR245C	-	-	1
0	DGR2	YKL121W	-	-	1
0	DHR2	YKL078W	-1	-	-1
0	DIA1	YMR316W	1	1	-
0	DIA4	YHR011W	-	-	1
0	DIC1	YLR348C	-	-	1
0	DID4	YKL002W	-	-1	-
0	DIF1	YLR437C	1	-	-
0	DIM1	YPL266W	-	-	-1
0	DIN7	YDR263C	-	-	1
0	DIP2	YLR129W	-	-	-1
0	DIP5	YPL265W	1	-	-
0	DIS3	YOL021C	-1	-	-
0	DIT1	YDR403W	1	1	1
0	DJP1	YIR004W	-	-1	-
0	DLD2	YDL178W	1	-1	-
0	DLD3	YEL071W	1	-	-
0	DLS1	YJL065C	-1	-	-
0	DLT1	YMR126C	-1	-1	-
0	DMA1	YHR115C	-	1	-
0	DMC1	YER179W	-	-	-1
0	DML1	YMR211W	-1	-	-
0	DNA2	YHR164C	-	1	-
0	DOA1	YKL213C	-	1	-
1	**DOA4**	**YDR069C**	-	1	1
0	DOG1	YHR044C	1	-	1
0	DOG2	YHR043C	1	-	1
0	DOP1	YDR141C	-	1	-
1	**DOT1**	**YDR440W**	-	-1	-1
0	DOT6	YER088C	-	1	1
1	**DPB4**	**YDR121W**	-	-	-1
0	DPH1	YIL103W	-1	-	-1
0	DPH2	YKL191W	-1	-	-1
0	DPH5	YLR172C	-	-	-1
0	DPP1	YDR284C	-	-	1
0	DRE2	YKR071C	-1	-	-
0	DRS1	YLL008W	-	-	-1
0	DSE1	YER124C	-1	-	-
0	DSE4	YNR067C	1	1	-1
0	DSF1	YEL070W	1	-	-

0	DSF1	YNR073C	1	-	-
0	DSF2	YBR007C	-	-	1
0	DST1	YGL043W	-	-	-1
0	DUF1	YOL087C	1	1	1
0	DUG3	YNL191W	-1	1	-
1	**DUN1**	**YDL101C**	-	-	1
0	DUO1	YGL061C	-	-1	-
0	DUR1,2	YBR208C	1	-	-1
0	DUR3	YHL016C	1	-	-
0	DUS1	YML080W	-1	-	-1
0	DUS3	YLR401C	-	-	-1
0	DUS4	YLR405W	-1	-	-
0	DUT1	YBR252W	-1	-	-1
0	EAF6	YJR082C	-	1	-
0	EAR1	YMR171C	-1	-	-
0	EBP2	YKL172W	-	-	-1
1	**EBS1**	**YDR206W**	-	-	-1
0	ECL1	YGR146C	1	-	-
0	ECM1	YAL059W	-1	-	-1
0	ECM10	YEL030W	-	-	-1
0	ECM13	YBL043W	-	-	1
0	ECM15	YBL001C	1	1	-
0	ECM16	YMR128W	-	-	-1
0	ECM17	YJR137C	-	-	-1
0	ECM19	YLR390W	-	1	-
0	ECM2	YBR065C	-	-	-1
0	ECM21	YBL101C	1	1	-
0	ECM22	YLR228C	-	-	-1
0	ECM27	YJR106W	1	-	-1
0	ECM29	YHL030W	-	1	-
0	ECM3	YOR092W	-	1	1
0	ECM30	YLR436C	1	1	1
0	ECM38	YLR299W	1	1	-
0	ECM4	YKR076W	1	1	1
0	ECM40	YMR062C	1	1	-
0	ECM5	YMR176W	1	-	-
0	ECM8	YBR076W	-	1	-
0	EDC1	YGL222C	-	-	1
0	EDC2	YER035W	1	1	1
0	EDC3	YEL015W	-1	-1	-1
0	EDE1	YBL047C	1	1	1
0	EDS1	YBR033W	1	-	-
0	EFM1	YHL039W	-	-	-1
0	EFM2	YBR271W	-1	-1	-1
0	EHT1	YBR177C	-	1	1
0	ELO1	YJL196C	-1	-	-1
0	ELP2	YGR200C	-1	-	-1
0	ELP3	YPL086C	-1	-	-1
0	ELP4	YPL101W	-1	-1	-1
0	ELP6	YMR312W	-	-	-1

0	EMG1	YLR186W	-1	-	-1
0	EMI1	YDR512C	1	1	-1
0	EMI2	YDR516C	-	1	1
0	EMI5	YOL071W	-	1	-
0	EMP46	YLR080W	1	1	1
0	EMP70	YLR083C	-1	-1	-1
0	EMW1	YNL313C	-1	-	-1
0	ENB1	YOL158C	1	-	-1
0	ENO1	YGR254W	-	1	1
0	ENP1	YBR247C	-1	-	-1
0	ENP2	YGR145W	-	-	-1
0	ENT2	YLR206W	1	-	-
0	ENT5	YDR153C	-1	-	-
0	ENV10	YLR065C	-	-1	-1
0	ENV7	YPL236C	-	-	1
0	ENV9	YOR246C	-	-	-1
0	EOS1	YNL080C	-1	-	-
0	EPL1	YFL024C	1	-	-
0	EPS1	YIL005W	-	-1	-
0	ERB1	YMR049C	-1	-	-1
0	ERC1	YHR032W	-1	-	-1
0	ERD1	YDR414C	-1	-1	-
0	ERF2	YLR246W	-	-	1
0	ERG1	YGR175C	1	-	-
0	ERG10	YPL028W	-	-	-1
0	ERG11	YHR007C	1	-	-
0	ERG12	YMR208W	1	-	-
0	ERG13	YML126C	-	-	-1
1	**ERG2**	**YMR202W**	1	-	-1
0	ERG25	YGR060W	1	-	-
0	ERG26	YGL001C	1	-	-
0	ERG27	YLR100W	-	-	-1
0	ERG28	YER044C	1	1	1
0	ERG3	YLR056W	1	-	-
0	ERG4	YGL012W	1	-	-1
0	ERG5	YMR015C	1	-	-
0	ERG6	YML008C	1	-	-1
0	ERG7	YHR072W	1	-	-1
0	ERG8	YMR220W	1	-1	-1
0	ERG9	YHR190W	1	-	-
1	**ERJ5**	**YFR041C**	-	-1	-
0	ERO1	YML130C	-	1	1
0	ERP6	YGL002W	-1	-	-
0	ERS1	YCR075C	-	-	1
0	ERT1	YBR239C	-1	-	-
0	ERV2	YPR037C	-1	-1	-
1	**ESA1**	**YOR244W**	-1	-	-
0	ESBP6	YNL125C	1	1	1
0	ESC1	YMR219W	1	-	-
0	ESF2	YNR054C	-	-	-1

1	**ESS1**	**YJR017C**	1	-	-
0	ETR1	YBR026C	-	1	1
0	ETT1	YOR051C	-	-1	-
0	EUG1	YDR518W	1	-	-
0	EXG2	YDR261C	-	1	-
0	FAA1	YOR317W	1	1	1
0	FAB1	YFR019W	1	1	-
0	FAF1	YIL019W	-	-	-1
1	**FAL1**	**YDR021W**	-1	-	-1
0	FAP1	YNL023C	-	-	-1
0	FAP7	YDL166C	-	-	-1
0	FAR1	YJL157C	-1	-1	-
0	FAR3	YMR052W	-	-	1
0	FAT1	YBR041W	-	-	1
0	FCY1	YPR062W	-	-	-1
0	FCY21	YER060W	-1	-1	-
0	FCY22	YER060W-A	-	-	-1
0	FEN1	YCR034W	-	-	-1
0	FEN2	YCR028C	-	-1	-1
0	FES1	YBR101C	-1	-	-
0	FET4	YMR319C	-1	-1	-
0	FHN1	YGR131W	1	-	-
0	FIS1	YIL065C	-	1	-
0	FIT1	YDR534C	1	-	-
0	FIT2	YOR382W	1	1	1
0	FIT3	YOR383C	1	1	1
0	FKH2	YNL068C	-	1	-
0	FKS1	YLR342W	1	-	-
0	FLC1	YPL221W	1	1	-
0	FLC2	YAL053W	1	1	-
0	FLD1	YLR404W	-1	-	-
0	FLO1	YAR050W	1	-	-
0	FLX1	YIL134W	-1	-	-
0	FMN1	YDR236C	-	-	-1
0	FMO1	YHR176W	1	-	-
0	FMP16	YDR070C	-	1	1
0	FMP21	YBR269C	-	1	-
0	FMP23	YBR047W	1	1	1
0	FMP25	YLR077W	-	1	-
0	FMP27	YLR454W	1	-	-
0	FMP33	YJL161W	1	1	1
0	FMP40	YPL222W	1	1	1
0	FMP41	YNL168C	1	-	-
0	FMP43	YGR243W	-	-	1
0	FMP45	YDL222C	-	1	-
0	FMP46	YKR049C	-	1	1
0	FMP48	YGR052W	1	1	-
0	FMS1	YMR020W	1	-	-
1	**FOB1**	**YDR110W**	-	-	-1
0	FOL2	YGR267C	-	-1	-1

0	FOL3	YMR113W	-	-	-1
0	FPK1	YNR047W	-	1	-
0	FPR3	YML074C	-	-1	-1
0	FPR4	YLR449W	-1	-1	-1
0	FRA1	YLL029W	1	-	-
0	FRA2	YGL220W	-	-	-1
0	FRE1	YLR214W	1	-1	-1
0	FRE3	YOR381W	1	-	-
0	FRE4	YNR060W	-	-	-1
0	FRE5	YOR384W	1	-	-
0	FRE6	YLL051C	1	-1	-1
0	FRK1	YPL141C	-	-	-1
0	FRS2	YFL022C	-1	-	-
0	FRT2	YAL028W	1	1	1
0	FSF1	YOR271C	-1	-1	-1
0	FSH1	YHR049W	-1	-	1
0	FSH2	YMR222C	-1	-	-
0	FSP2	YIL172C	1	1	-1
0	FSP2	YJL221C	1	1	-1
0	FSP2	YOL157C	1	1	-1
0	FTR1	YER145C	1	-	-
0	FUI1	YBL042C	-	-	-1
0	FUN14	YAL008W	-	1	1
0	FUN19	YAL034C	1	1	-
0	FUR1	YHR128W	-1	-	-1
0	FUS1	YCL027W	-1	-1	-
0	FUS3	YBL016W	-1	-1	-
0	FYV10	YIL097W	-	1	1
1	**FYV4**	**YHR059W**	-	1	1
0	FYV5	YCL058C	-1	-	-
1	**FYV6**	**YNL133C**	-	-	1
0	FYV8	YGR196C	1	-	-
0	GAA1	YLR088W	1	-	-
0	GAC1	YOR178C	-	1	1
0	GAD1	YMR250W	-	1	1
0	GAL3	YDR009W	1	1	1
0	GAL80	YML051W	-	-	1
0	GAP1	YKR039W	1	-	-1
0	GAR1	YHR089C	-	-1	-1
0	GAS2	YLR343W	-	1	1
0	GAT1	YFL021W	-	-	1
0	GAT2	YMR136W	-1	1	1
0	GCD10	YNL062C	-1	-	-1
0	GCD11	YER025W	-1	-1	-1
0	GCD14	YJL125C	-1	-	-1
0	GCD2	YGR083C	-	-	-1
0	GCD7	YLR291C	-1	-	-1
0	GCN3	YKR026C	-1	-1	-1
0	GCR1	YPL075W	1	-	-
0	GCV1	YDR019C	1	1	1

0	GCV2	YMR189W	1	1	1
1	**GCV3**	**YAL044C**	1	1	1
0	GCY1	YOR120W	1	1	1
0	GDB1	YPR184W	1	1	1
0	GDE1	YPL110C	1	-	-
0	GDH1	YOR375C	1	-1	-1
0	GDH2	YDL215C	1	1	1
0	GDH3	YAL062W	1	1	-
0	GDT1	YBR187W	-1	-	-
0	GEA2	YEL022W	1	-	-
0	GEP4	YHR100C	-1	-1	-1
0	GEP7	YGL057C	-1	-	-
0	GET1	YGL020C	-	-1	-
0	GFA1	YKL104C	1	-	-
0	GFD1	YMR255W	-	-1	-
0	GFD2	YCL036W	-1	-	-1
0	GGA1	YDR358W	1	-	-
0	GGC1	YDL198C	-	-1	1
0	GIC2	YDR309C	-	-	1
0	GID7	YCL039W	1	-	1
0	GID8	YMR135C	1	1	1
0	GIP2	YER054C	1	1	1
0	GIP3	YPL137C	-	1	-
0	GIP4	YAL031C	-	-	1
0	GIR2	YDR152W	-	-1	-
0	GIS1	YDR096W	-	-	1
0	GIS2	YNL255C	-	-1	-
0	GLC3	YEL011W	1	1	1
0	GLC7	YER133W	-	-1	-
0	GLC8	YMR311C	-	-	1
0	GLE2	YER107C	-1	-1	-1
0	GLG1	YKR058W	-	-	1
0	GLG2	YJL137C	-1	-	-
0	GLK1	YCL040W	1	1	1
0	GLN1	YPR035W	1	-	1
1	**GLO4**	**YOR040W**	1	-	-
0	GLR1	YPL091W	-1	-	-
0	GLT1	YDL171C	-	-	-1
0	GLY1	YEL046C	-1	-1	-1
0	GMC1	YDR506C	1	-	1
0	GMH1	YKR030W	-	-1	-
0	GND2	YGR256W	1	1	1
0	GNP1	YDR508C	-	-	-1
1	**GON7**	**YJL184W**	-	-	-1
0	GOR1	YNL274C	1	1	1
0	GOT1	YMR292W	-	-1	-
0	GPA1	YHR005C	-1	-1	-1
0	GPA2	YER020W	1	-	1
0	GPD1	YDL022W	-1	1	-
0	GPD2	YOL059W	-	-	-1

0	GPG1	YGL121C	1	1	1
0	GPH1	YPR160W	1	1	1
0	GPI11	YDR302W	-1	-	-
0	GPI15	YNL038W	-1	-1	-
0	GPI18	YBR004C	1	-	-1
0	GPI2	YPL076W	-1	-	-
0	GPM2	YDL021W	1	1	1
0	GPM3	YOL056W	-	-1	-1
0	GPR1	YDL035C	1	1	1
0	GPT2	YKR067W	1	1	1
0	GPX2	YBR244W	-	-1	-1
0	GRC3	YLL035W	-	-	-1
0	GRE2	YOL151W	-1	1	-
0	GRE3	YHR104W	-	1	1
0	GRR1	YJR090C	-	1	-
0	GRS2	YPR081C	1	-	-
0	GRX1	YCL035C	1	1	1
0	GRX2	YDR513W	-	1	1
0	GRX4	YER174C	-1	-1	-1
0	GRX6	YDL010W	1	-1	1
0	GRX8	YLR364W	-1	1	-
0	GSC2	YGR032W	1	1	1
0	GSP2	YOR185C	1	1	1
0	GSY1	YFR015C	-	-	1
0	GSY2	YLR258W	-	1	1
0	GTB1	YDR221W	1	-	-1
0	GTF1	YGR102C	-	1	1
0	GTO1	YGR154C	-	-	-1
0	GTO3	YMR251W	1	-	-
1	**GTR2**	**YGR163W**	-	-1	-
0	GTS1	YGL181W	-	-1	-
0	GTT1	YIR038C	1	-	-
0	GTT2	YLL060C	1	-	1
0	GUA1	YMR217W	-	-	-1
0	GUD1	YDL238C	-	1	-
0	GUF1	YLR289W	-	1	-
0	GUT1	YHL032C	-	-	1
0	GUT2	YIL155C	1	-	1
0	GWT1	YJL091C	-	-	1
0	GYL1	YMR192W	1	-	-
0	GYP5	YPL249C	-	1	-
0	GYP7	YDL234C	1	1	1
0	GYP8	YFL027C	-	-1	-
0	HAA1	YPR008W	-	1	1
0	HAL1	YPR005C	1	1	-
0	HAM1	YJR069C	-	-	-1
0	HAP1	YLR256W	1	-	1
0	HAP3	YBL021C	-1	-1	-
0	HAP4	YKL109W	-1	-	1
0	HAP5	YOR358W	1	1	-

0	HAS1	YMR290C	-	-	-1
0	HAT1	YPL001W	-	-1	-1
0	HBN1	YCL026C-B	1	1	1
0	HBS1	YKR084C	1	-	-
0	HBT1	YDL223C	1	1	-
0	HCA4	YJL033W	-	-	-1
1	**HCH1**	**YNL281W**	-	1	-
1	**HCR1**	**YLR192C**	-	-1	-
1	**HEK2**	**YBL032W**	-	-1	-
0	HEM12	YDR047W	-	-1	-
0	HEM13	YDR044W	1	-	-
0	HEM14	YER014W	1	-	-
0	HEM3	YDL205C	-1	-	-
0	HER1	YOR227W	1	1	1
0	HES1	YOR237W	1	-	-
0	HFA1	YMR207C	1	-	-
1	**HFI1**	**YPL254W**	-1	-1	-
0	HFM1	YGL251C	-	-	-1
0	HGH1	YGR187C	-1	-	-1
0	HHO1	YPL127C	-1	-1	-
0	HIM1	YDR317W	-	1	-
0	HIR1	YBL008W	-	1	-
0	HIR2	YOR038C	-1	-	-
0	HIS1	YER055C	-	-1	-1
0	HIS3	YOR202W	1	1	-
0	HIS4	YCL030C	1	-1	-1
0	HIS5	YIL116W	1	-	-
0	HIS6	YIL020C	-1	-	-1
0	HIS7	YBR248C	1	-	-1
1	**HIT1**	**YJR055W**	-1	-	-
0	HLJ1	YMR161W	-1	-1	-
0	HMF1	YER057C	-	-1	1
0	HMG1	YML075C	1	-	-1
0	HMI1	YOL095C	-1	-	-1
1	**HMO1**	**YDR174W**	-1	-	-
0	HMS1	YOR032C	-	-	1
0	HMS2	YJR147W	-1	-	-1
0	HMT1	YBR034C	-1	-	-1
0	HMX1	YLR205C	1	-	-
0	HNM1	YGL077C	-1	-	-
0	HNT2	YDR305C	-	-1	-
0	HO	YDL227C	-1	-1	-
0	HOM2	YDR158W	-1	-	-
0	HOP1	YIL072W	-	-1	-1
0	HOR2	YER062C	-	1	1
0	HOR7	YMR251W-A	1	1	1
0	HOS3	YPL116W	-	-	1
0	HOT1	YMR172W	1	-	-
0	HOT13	YKL084W	1	-	-1
0	HPA3	YEL066W	1	1	-

0	HPF1	YOL155C	-1	1	-1
0	HPM1	YIL110W	-	-1	-1
0	HPT1	YDR399W	-1	-	-1
0	HRB1	YNL004W	-	-1	-
0	HRI1	YLR301W	1	-	-
0	HRP1	YOL123W	-	-1	-1
1	**HRT1**	**YOL133W**	-1	-	-
0	HSH155	YMR288W	-1	-	-1
0	HSK3	YKL138C-A	1	-	-
0	HSP10	YOR020C	-	1	-
1	**HSP104**	**YLL026W**	1	1	1
0	HSP12	YFL014W	-	1	1
0	HSP150	YJL159W	1	1	-
0	HSP26	YBR072W	1	1	1
0	HSP30	YCR021C	-	1	1
0	HSP31	YDR533C	1	1	1
0	HSP42	YDR171W	-	1	1
0	HSP60	YLR259C	-	1	-
0	HSP78	YDR258C	1	1	1
1	**HSP82**	**YPL240C**	-	1	1
1	**HST1**	**YOL068C**	-1	-	-
0	HST4	YDR191W	-1	-	-
0	HTA2	YBL003C	-1	-	-
0	HTB2	YBL002W	-1	-	-
1	**HTL1**	**YCR020W-B**	-1	-	-
0	HTS1	YPR033C	-	-	-1
0	HTZ1	YOL012C	-	-1	-
0	HUG1	YML058W-A	1	1	-
0	HUT1	YPL244C	-1	-	-
0	HXK1	YFR053C	-	1	1
0	HXT1	YHR094C	-1	-	-1
0	HXT11	YOL156W	1	-	-
0	HXT2	YMR011W	-	-1	-1
0	HXT3	YDR345C	1	-	-
0	HXT4	YHR092C	-1	-	1
0	HXT6	YDR343C	-1	1	1
0	HXT7	YDR342C	-1	1	1
0	HYM1	YKL189W	-1	-	1
0	HYR1	YIR037W	-	-	1
0	IAH1	YOR126C	1	-	-
0	IBD2	YNL164C	-	-	-1
0	ICL1	YER065C	-	-	1
0	ICL2	YPR006C	1	1	-
0	ICP55	YER078C	-1	-1	1
0	ICS2	YBR157C	1	-	1
0	ICS3	YJL077C	1	-	-
0	ICT1	YLR099C	-1	1	-
0	ICY1	YMR195W	1	1	1
0	ICY2	YPL250C	-	-	1
0	IDH1	YNL037C	1	1	1

0	IDH2	YOR136W	1	1	1
0	IDP1	YDL066W	1	-	-
0	IDP2	YLR174W	-	1	-
0	IDP3	YNL009W	1	-	-
0	IES2	YNL215W	-	-	-1
0	IES4	YOR189W	-	-1	1
0	IES5	YER092W	1	-	-
1	**IES6**	**YEL044W**	-	-	-1
0	IFM1	YOL023W	1	1	1
0	IGD1	YFR017C	1	1	1
0	IKI1	YHR187W	-1	-	-1
0	IKI3	YLR384C	-	-	-1
0	IKS1	YJL057C	-	1	1
0	ILM1	YJR118C	-	-1	-
0	ILV2	YMR108W	-	-1	-
0	ILV3	YJR016C	-1	-1	-
1	**ILV5**	**YLR355C**	-1	-1	-
0	IMA1	YGR287C	-	1	-1
0	IMD1	YAR073W	-1	-1	-
0	IMD2	YAR075W	-	-	-1
0	IMD2	YHR216W	-1	-1	-1
0	IMD3	YHR216W	-1	-1	-1
0	IMD3	YLR432W	-1	-	-1
0	IMD4	YML056C	-1	-	-1
0	IMG1	YCR046C	-	1	-
1	**IMG2**	**YCR071C**	-	1	1
0	IML2	YJL082W	1	1	-
0	IMP3	YHR148W	-1	-	-1
0	IMP4	YNL075W	-1	-	-1
0	INH1	YDL181W	-	1	1
0	INO1	YJL153C	1	1	1
0	INO2	YDR123C	-	-	-1
0	INP1	YMR204C	-	-1	-
0	INP2	YMR163C	-1	-	-
0	INP53	YOR109W	-1	-	-
0	IPI1	YHR085W	-1	-	-1
0	IPI3	YNL182C	-1	-	-1
1	**IPK1**	**YDR315C**	1	-	-
0	IPL1	YPL209C	-1	-	-
0	IPT1	YDR072C	-1	-1	-1
0	IRA1	YBR140C	1	1	-
0	IRC10	YOL015W	1	-	-
1	**IRC15**	**YPL017C**	1	1	1
0	IRC18	YJL037W	1	1	1
0	IRC23	YOR044W	-	-	1
0	IRC25	YLR021W	-	1	-
0	IRC7	YFR055W	-	-1	-1
0	IRR1	YIL026C	1	-	-
0	ISC10	YER180C	-	-1	-
0	ISM1	YPL040C	-	-	1

0	ISN1	YOR155C	-	-	1
0	ISU1	YPL135W	-	1	-
0	ISU2	YOR226C	-1	-1	1
0	ITC1	YGL133W	-	1	-
0	IZH1	YDR492W	-1	-	-1
0	IZH2	YOL002C	-1	-1	-1
0	IZH3	YLR023C	1	-	-
0	JAC1	YGL018C	1	1	-
0	JHD2	YJR119C	-	-	1
0	JID1	YPR061C	-	1	1
0	JIP4	YDR475C	-	1	1
0	JIP5	YPR169W	-	-1	-1
0	JJJ3	YJR097W	-	-	-1
0	JSN1	YJR091C	1	1	1
0	KAP104	YBR017C	-	1	-
0	KAP122	YGL016W	-1	-	-
0	KAP123	YER110C	-	-	-1
0	KAP95	YLR347C	-1	1	-
0	KAR2	YJL034W	-	1	-
0	KAR4	YCL055W	-1	-1	-
0	KAR5	YMR065W	-1	-1	-
0	KAR9	YPL269W	-1	-	-
1	**KCS1**	**YDR017C**	-1	-	-
0	KDX1	YKL161C	1	-	-
0	KEG1	YFR042W	-1	1	-
0	KEI1	YDR367W	-	-	-1
0	KEL1	YHR158C	1	-	-
0	KEL2	YGR238C	1	1	1
0	KEL3	YPL263C	-	-	-1
0	KES1	YPL145C	-	-	-1
0	KEX2	YNL238W	-1	-	-
0	KGD1	YIL125W	-	-	1
0	KIC1	YHR102W	-	1	-
0	KIN1	YDR122W	-	1	1
0	KIN28	YDL108W	-1	-	-
0	KIN3	YAR018C	-	-1	-
0	KIN82	YCR091W	1	1	1
0	KIP1	YBL063W	1	-	1
0	KIP3	YGL216W	-1	-	-
0	KNH1	YDL049C	1	1	1
0	KNS1	YLL019C	1	1	1
0	KRE1	YNL322C	-	1	1
0	KRE27	YIL027C	-1	-	-
1	**KRE33**	**YNL132W**	-	-	-1
0	KRE6	YPR159W	1	-	1
0	KRE9	YJL174W	1	-	-
0	KRR1	YCL059C	-1	-1	-1
1	**KRS1**	**YDR037W**	-	-1	-1
0	KSP1	YHR082C	1	1	1
0	KSS1	YGR040W	-1	-	-

0	KTI11	YBL071W-A	-	1	-1
0	KTI12	YKL110C	-	-1	-1
0	KTR2	YKR061W	1	-	1
0	KTR4	YBR199W	1	-	-
0	KTR6	YPL053C	-	-	-1
0	KTR7	YIL085C	-1	-1	-
0	LAA1	YJL207C	-1	-	-
0	LAG1	YHL003C	-1	-	-
0	LAP2	YNL045W	1	-	-
0	LAP3	YNL239W	1	-	1
0	LAP4	YKL103C	1	1	1
0	LCB2	YDR062W	-	-1	-
0	LCB3	YJL134W	-1	-	1
0	LCB5	YLR260W	1	-	-
0	LCL1	YPL056C	-	-	-1
0	LCL3	YGL085W	-1	-	-
0	LCP5	YER127W	-	-	-1
1	**LDB7**	**YBL006C**	-1	-	-
0	LDH1	YBR204C	1	-	1
0	LEM3	YNL323W	-1	-	-
1	**LEO1**	**YOR123C**	-	-1	-
0	LEU1	YGL009C	-1	-1	-
0	LEU4	YNL104C	-	-	1
0	LEU5	YHR002W	-1	-	-
0	LEU9	YOR108W	-1	-1	-1
0	LHP1	YDL051W	-1	-	-1
0	LIA1	YJR070C	-1	-	-1
0	LIP5	YOR196C	-1	-1	-1
0	LOC1	YFR001W	-	-	-1
0	LOH1	YJL038C	1	1	1
0	LOT5	YKL183W	-	-1	-
0	LPE10	YPL060W	-	-	1
0	LPP1	YDR503C	-1	-	-
0	LRG1	YDL240W	-1	-1	-
0	LRO1	YNR008W	1	-	-
1	**LRP1**	**YHR081W**	-	-1	-1
0	LSB3	YFR024C-A	1	-	-
0	LSC2	YGR244C	-	1	1
1	**LSG1**	**YGL099W**	-1	-	-1
0	LSM1	YJL124C	-	-1	-
0	LSM12	YHR121W	-	-1	-
0	LSM2	YBL026W	-1	-	-
0	LSP1	YPL004C	-	-	1
1	**LST7**	**YGR057C**	-	-	-1
0	LTV1	YKL143W	-	-1	-1
0	LUC7	YDL087C	-1	-	-1
0	LYS1	YIR034C	1	1	-
0	LYS2	YBR115C	1	-	-
0	LYS20	YDL182W	-	-	-1
0	LYS21	YDL131W	-	1	-

0	LYS4	YDR234W	-1	-	-1
0	LYS9	YNR050C	-	-1	-
0	MAD3	YJL013C	1	-	-
0	MAG1	YER142C	-	-	1
1	**MAK10**	**YEL053C**	-	-1	-
0	MAK11	YKL021C	-1	-1	-1
0	MAK16	YAL025C	-	-	-1
1	**MAK21**	**YDR060W**	-	-	-1
1	**MAK3**	**YPR051W**	-	-1	-
0	MAK32	YCR019W	-1	-	-
0	MAK5	YBR142W	-	-	-1
0	MAL12	YGR292W	-	-	-1
0	MAL32	YBR299W	-	-	-1
0	MAL33	YBR297W	1	-1	1
0	MAM33	YIL070C	-	1	1
0	MAP1	YLR244C	-1	-	-
0	MBA1	YBR185C	-	1	-
0	MBR1	YKL093W	-1	-	-
0	MCA1	YOR197W	-	-	-1
0	MCH1	YDL054C	1	-	-
0	MCH4	YOL119C	1	-	-
0	MCH5	YOR306C	1	-	-1
0	MCK1	YNL307C	-	-1	-
0	MCM1	YMR043W	-1	-	-
0	MCM10	YIL150C	-	-	-1
0	MCM21	YDR318W	-1	-	-
0	MCM22	YJR135C	-	-	-1
1	**MCM3**	**YEL032W**	-1	-	-
1	**MCM6**	**YGL201C**	-	-	-1
0	MCR1	YKL150W	-	1	1
0	MCT1	YOR221C	1	-	1
0	MCX1	YBR227C	1	-1	-1
0	MDE1	YJR024C	-1	-	-1
0	MDG1	YNL173C	-	1	1
0	MDH1	YKL085W	-	-	1
0	MDJ1	YFL016C	-	1	1
0	MDL1	YLR188W	-	-	-1
1	**MDM10**	**YAL010C**	1	-	-
0	MDM12	YOL009C	-	-	1
0	MDM30	YLR368W	-	1	-
0	MDM31	YHR194W	-	-	1
0	MDM34	YGL219C	1	-	1
0	MDM35	YKL053C-A	1	1	1
0	MDS3	YGL197W	1	-	-
0	MED2	YDL005C	-	-1	-
0	MED4	YOR174W	-1	-	-
0	MED6	YHR058C	-1	-	-
0	MEF1	YLR069C	-	1	1
0	MEF2	YJL102W	-	-	1
0	MEH1	YKR007W	-	-1	-

0	MEI4	YER044C-A	1	1	-
0	MEP1	YGR121C	1	1	-1
0	MEP2	YNL142W	1	-	-
0	MET1	YKR069W	-	-	-1
0	MET10	YFR030W	-	-	-1
0	MET14	YKL001C	-	-	-1
0	MET2	YNL277W	-1	-	-
0	MET22	YOL064C	-1	-1	-
0	MET28	YIR017C	1	-	-
0	MET3	YJR010W	-	-	-1
0	MET31	YPL038W	-1	-1	-
0	MET32	YDR253C	-1	-	-
1	**MET7**	**YOR241W**	-1	-1	-1
0	MEU1	YLR017W	-1	-	-1
0	MF(ALPHA)2	YGL089C	-1	-	-
0	MFA1	YDR461W	-1	-1	-
0	MFA2	YNL145W	-1	-1	-
0	MFB1	YDR219C	-	-	1
0	MGM101	YJR144W	-1	-	-
0	MGR2	YPL098C	-	1	-
0	MGT1	YDL200C	1	-	-
0	MHO1	YJR008W	1	1	1
0	MHP1	YJL042W	-	1	1
0	MHR1	YDR296W	-	1	1
0	MHT1	YLL062C	-1	-1	-1
0	MIA40	YKL195W	-	-	1
0	MIC14	YDR031W	-	1	1
0	MIC17	YMR002W	-1	1	-
0	MIG2	YGL209W	-	-	-1
0	MIG3	YER028C	-	-	-1
0	MIH1	YMR036C	-1	-	-
0	MIM1	YOL026C	1	-	-
0	MIM2	YLR099W-A	-	1	-
0	MIP6	YHR015W	1	1	-
0	MIR1	YJR077C	-1	-	1
0	MKC7	YDR144C	-	-	-1
0	MKK2	YPL140C	-	-	1
0	MLC2	YPR188C	-	1	-
0	MLF3	YNL074C	1	1	-
0	MLH3	YPL164C	1	1	-
0	MLS1	YNL117W	-	-	1
1	**MMM1**	**YLL006W**	-	-1	-1
0	MMP1	YLL061W	-1	-1	-1
0	MMS1	YPR164W	-	-	-1
0	MMS2	YGL087C	-	1	1
1	**MMS21**	**YEL019C**	-	1	-
0	MMT1	YMR177W	-	-1	-
0	MMT2	YPL224C	1	-	-
0	MND2	YIR025W	-	-	-1
0	MNL1	YHR204W	-	-	-1

0	MNN1	YER001W	-1	-	-
0	MNN10	YDR245W	-1	-1	-1
0	MNN11	YJL183W	-	-	-1
0	MNN4	YKL201C	1	-	-
0	MNN5	YJL186W	-1	-	-
0	MNP1	YGL068W	-	1	-
0	MNS1	YJR131W	-	-	-1
0	MNT2	YGL257C	-	-1	-1
0	MNT3	YIL014W	-	-	1
0	MOB2	YFL034C-B	-1	-	-
0	MOG1	YJR074W	1	1	1
0	MON1	YGL124C	-	-	-1
0	MON2	YNL297C	1	1	-
0	MOS1	YCL057C-A	-	1	1
0	MOT1	YPL082C	-1	1	-
0	MPC54	YOR177C	1	1	-
0	MPD1	YOR288C	-	-	1
0	MPE1	YKL059C	-	-	1
0	MPH1	YIR002C	-	1	-
0	MPM1	YJL066C	1	1	1
0	MPP10	YJR002W	-	-	-1
0	MPT5	YGL178W	-	1	-
1	**MRC1**	**YCL061C**	1	-	-
0	MRH4	YGL064C	-	1	-
0	MRI1	YPR118W	-1	-1	-1
0	MRK1	YDL079C	-	1	1
0	MRM1	YOR201C	-	1	-
0	MRN1	YPL184C	-	-1	1
0	MRP1	YDR347W	-	1	-
0	MRP10	YDL045W-A	-	1	1
0	MRP13	YGR084C	-	-	1
0	MRP17	YKL003C	-	1	-
0	MRP20	YDR405W	-	-	1
0	MRP21	YBL090W	-1	-	-
0	MRP4	YHL004W	-	-	1
0	MRP49	YKL167C	-	1	1
0	MRP51	YPL118W	-	-	1
0	MRP7	YNL005C	-	-	1
0	MRP8	YKL142W	-	1	1
0	MRPL1	YDR116C	-	-	1
0	MRPL10	YNL284C	-1	-	-
0	MRPL11	YDL202W	-	-	1
0	MRPL13	YKR006C	-	1	1
0	MRPL15	YLR312W-A	-	1	-
0	MRPL16	YBL038W	-	-	1
0	MRPL17	YNL252C	-1	-	1
0	MRPL19	YNL185C	-	1	1
0	MRPL20	YKR085C	-1	1	-
0	MRPL22	YNL177C	-	-	1
0	MRPL23	YOR150W	-	1	1

0	MRPL24	YMR193W	-	-	1
1	**MRPL3**	**YMR024W**	-	-	1
0	MRPL32	YCR003W	-	-	1
0	MRPL33	YMR286W	-	1	-
0	MRPL36	YBR122C	-	1	1
0	MRPL37	YBR268W	-	-	1
1	**MRPL38**	**YKL170W**	-	-	1
0	MRPL39	YML009C	-	1	1
0	MRPL4	YLR439W	-	1	-
0	MRPL40	YPL173W	-	1	1
1	**MRPL44**	**YMR225C**	-	1	1
0	MRPL50	YNR022C	-	1	-
0	MRPL6	YHR147C	-	1	1
0	MRPL9	YGR220C	-	1	1
0	MRPS17	YMR188C	-	-	1
1	**MRPS18**	**YNL306W**	-	1	-
0	MRPS28	YDR337W	-	-	1
0	MRPS5	YBR251W	-	-	1
0	MRPS8	YMR158W	-	1	-
0	MRS1	YIR021W	-1	-	-
0	MRS2	YOR334W	-	1	1
0	MRS3	YJL133W	-1	-	-
0	MRS4	YKR052C	-	-1	-1
1	**MRT4**	**YKL009W**	-1	-1	-1
0	MSA2	YKR077W	-	-	1
0	MSB2	YGR014W	-	-	1
0	MSB3	YNL293W	1	-	-
0	MSB4	YOL112W	1	-1	-1
0	MSC1	YML128C	1	1	1
0	MSC3	YLR219W	-	-	1
0	MSC6	YOR354C	-	-	1
0	MSC7	YHR039C	1	-	-
0	MSD1	YPL104W	1	1	-
0	MSF1	YPR047W	-	1	1
0	MSH1	YHR120W	-1	-	-
0	MSH5	YDL154W	-	-1	-
0	MSL1	YIR009W	1	-	-
0	MSL5	YLR116W	-1	-	-
0	MSM1	YGR171C	-	1	-
0	MSN4	YKL062W	1	1	1
0	MSN5	YDR335W	1	1	-
0	MSP1	YGR028W	-	1	1
0	MSS1	YMR023C	-	-	1
0	MSS4	YDR208W	-	1	-
0	MST27	YGL051W	-	-	-1
0	MSY1	YPL097W	-	1	1
0	MTC2	YKL098W	-	1	-
0	MTC3	YGL226W	-	1	-
0	MTD1	YKR080W	1	1	1
0	MTF1	YMR228W	-1	-	-

0	MTG2	YHR168W	-1	-	-
0	MTH1	YDR277C	-	-	1
0	MTL1	YGR023W	1	1	1
0	MTO1	YGL236C	-	1	-
0	MTQ1	YNL063W	-1	-	-
0	MTQ2	YDR140W	-1	-	-1
1	**MTR10**	**YOR160W**	-1	-	-
1	**MTR3**	**YGR158C**	-	-	-1
0	MTR4	YJL050W	-	-	-1
0	MTW1	YAL034W-A	-	-1	-
0	MUB1	YMR100W	-1	-	-
0	MUD1	YBR119W	-	-	1
0	MUD2	YKL074C	-1	-	-
0	MUK1	YPL070W	1	-	-
0	MUM2	YBR057C	-1	-1	-
0	MUP1	YGR055W	-1	-	-
0	MUQ1	YGR007W	-1	-1	-
0	MVD1	YNR043W	-	-	-1
0	MVP1	YMR004W	1	-	-
0	MXR1	YER042W	-1	-	-1
0	MXR2	YCL033C	-	1	-
0	MYO1	YHR023W	1	-	-
0	MYO2	YOR326W	1	-	-
0	MYO3	YKL129C	1	1	1
0	MYO4	YAL029C	-	-	1
0	MZM1	YDR493W	-	1	-
0	NAB2	YGL122C	-	-1	-1
0	NAB3	YPL190C	-	-	-1
0	NAF1	YNL124W	-1	-	-1
0	NAM2	YLR382C	-	1	1
0	NAN1	YPL126W	-	-	-1
0	NAT2	YGR147C	-1	-	-
0	NAT4	YMR069W	-	1	-
0	NBA1	YOL070C	-	-	-1
0	NBP35	YGL091C	-1	-	-
0	NCA3	YJL116C	1	1	1
0	NCB2	YDR397C	-	-	-1
0	NCE101	YJL205C	-	-	1
0	NCE102	YPR149W	-	-	1
0	NCL1	YBL024W	-	-1	-1
1	**NCP1**	**YHR042W**	1	-	-
0	NCS2	YNL119W	-1	-	-1
0	NDD1	YOR372C	-	-	1
0	NDE1	YMR145C	-	-	-1
0	NDE2	YDL085W	-	1	1
0	NDI1	YML120C	-	-	1
0	NDJ1	YOL104C	-1	-	-
0	NDL1	YLR254C	1	-	1
0	NDT80	YHR124W	1	-	1
1	**NET1**	**YJL076W**	1	-	-

0	NFS1	YCL017C	1	-	-
0	NGL1	YOL042W	-1	-1	-1
0	NGL3	YML118W	1	1	1
0	NHA1	YLR138W	-	-	-1
1	**NHP2**	**YDL208W**	-	-1	-1
0	NHP6A	YPR052C	-1	-1	-
0	NHP6B	YBR089C-A	1	-	1
0	NHP6B	YBR090C	1	-	1
0	NHX1	YDR456W	1	-	1
0	NIC96	YFR002W	-1	-	-
0	NIP100	YPL174C	1	-	-
0	NIP7	YPL211W	-1	-	-1
0	NIS1	YNL078W	-1	-	-
0	NIT2	YJL126W	-1	-	-
0	NMA1	YLR328W	-1	-	-1
0	NMA2	YGR010W	1	-	-
0	NMD3	YHR170W	-1	-1	-1
0	NMD4	YLR363C	-	-1	-1
0	NMD5	YJR132W	-	-	-1
0	NNF1	YJR112W	-1	-1	-
0	NNK1	YKL171W	1	-	-
0	NNT1	YLR285W	-	-1	-1
0	NOB1	YOR056C	-1	-1	-1
0	NOC2	YOR206W	-	-	-1
0	NOC3	YLR002C	-1	-1	-1
1	**NOC4**	**YPR144C**	-1	-	-1
0	NOG1	YPL093W	-1	-	-1
0	NOG2	YNR053C	-1	-	-1
0	NOP1	YDL014W	-1	-	-1
0	NOP12	YOL041C	-	-	-1
0	NOP13	YNL175C	-1	-1	-1
0	NOP14	YDL148C	-	-	-1
0	NOP15	YNL110C	-	-	-1
0	NOP16	YER002W	-	-1	-
0	NOP19	YGR251W	-	-1	-1
0	NOP2	YNL061W	-	-	-1
0	NOP4	YPL043W	-	-	-1
0	NOP58	YOR310C	-	-1	-1
0	NOP6	YDL213C	-	-1	-1
0	NOP7	YGR103W	-	-1	-1
0	NOP8	YOL144W	-	-	-1
0	NOP9	YJL010C	-	-1	-1
0	NOT5	YPR072W	-	-1	-
0	NPA3	YJR072C	-	-	-1
1	**NPL6**	**YMR091C**	-1	-1	-1
1	**NPP1**	**YCR026C**	1	-	-
0	NPR1	YNL183C	-	1	-
0	NPR2	YEL062W	1	1	-
0	NPT1	YOR209C	-	-1	-
0	NQM1	YGR043C	1	1	1

0	NRD1	YNL251C	-	-1	-1
0	NRG1	YDR043C	1	-	1
0	NRG2	YBR066C	1	-	1
0	NRK1	YNL129W	-	-1	-1
0	NRM1	YNR009W	-1	-	-
0	NRT1	YOR071C	1	-	-
0	NSA1	YGL111W	-	-	-1
0	NSA2	YER126C	-	-1	-1
0	NSE1	YLR007W	1	-	-
0	NSE3	YDR288W	-1	-	-
0	NSE4	YDL105W	-	-1	-
0	NSE5	YML023C	-1	-	-1
0	NSG1	YHR133C	-1	-1	-
0	NSG2	YNL156C	1	-	-
0	NSI1	YDR026C	-	-	-1
1	**NSR1**	**YGR159C**	-1	-	-1
0	NTE1	YML059C	-	1	1
0	NTG1	YAL015C	-	1	-
0	NTH1	YDR001C	1	1	1
0	NTH2	YBR001C	-	1	1
1	**NTR2**	**YKR022C**	1	-	-
0	NUC1	YJL208C	-1	-1	-1
0	NUG1	YER006W	-1	-1	-1
0	NUP157	YER105C	-	1	-
0	NUP170	YBL079W	-	1	-
0	NUP188	YML103C	-	1	-
0	NUP49	YGL172W	-1	-	-
0	NUP57	YGR119C	-	-1	-
1	**NUP60**	**YAR002W**	-1	-	-
0	NUP85	YJR042W	-	-1	-
0	NVJ1	YHR195W	-	-	1
0	OAC1	YKL120W	-1	-1	-
0	OAF1	YAL051W	-	1	-
0	OCA2	YNL056W	-	-1	-
0	OCA6	YDR067C	-	-	1
0	OCH1	YGL038C	-	-	1
0	OCT1	YKL134C	-1	-	-
0	ODC2	YOR222W	-1	-	-
0	OLA1	YBR025C	-1	-	-
0	OM14	YBR230C	-1	1	1
0	OM45	YIL136W	-	1	1
0	OMA1	YKR087C	-1	-	-
1	**OPI1**	**YHL020C**	-1	-1	-
0	OPI3	YJR073C	1	1	1
0	OPT1	YJL212C	-1	-	-1
0	OPT2	YPR194C	-	1	-
0	OPY1	YBR129C	-	-1	-
0	OPY2	YPR075C	-	-	1
1	**ORC5**	**YNL261W**	-1	-	-
0	ORM2	YLR350W	-1	-	-

0	ORT1	YOR130C	1	-	-
0	OSH2	YDL019C	-	-	1
0	OSH6	YKR003W	-	-	-1
0	OST2	YOR103C	-1	-	-
0	OST6	YML019W	-	-1	-
0	OSW2	YLR054C	1	1	1
0	OSW5	YMR148W	-	-	1
0	OTU1	YFL044C	-	-	-1
0	OTU2	YHL013C	-	-	-1
0	OXP1	YKL215C	-	1	-
0	OYE3	YPL171C	-	-	1
0	PAC1	YOR269W	-1	-1	-
0	PAC10	YGR078C	-1	-	-
0	PAC2	YER007W	-1	-	-1
0	PAD1	YDR538W	-1	-1	-
0	PAI3	YMR174C	1	1	1
0	PAM1	YDR251W	-	-	1
0	PAM18	YLR008C	-1	-	-
0	PAN1	YIR006C	1	-	-
0	PAN6	YIL145C	-	-	-1
0	PAP2	YOL115W	-1	-	-
0	PAU1	YIL176C	-	-1	-
0	PAU1	YJL223C	-	-1	-
0	PAU10	YDR542W	1	-1	-
0	PAU11	YGL261C	1	-1	-
0	PAU12	YGR294W	1	-1	-
0	PAU13	YHL046C	1	-1	-
0	PAU2	YEL049W	1	1	1
0	PAU4	YLR461W	1	-1	-
0	PAU4	YOL161C	1	-1	-
0	PAU5	YFL020C	1	1	1
0	PAU6	YLL064C	1	-1	-
0	PAU6	YNR076W	1	-1	-
0	PAU7	YAR020C	1	1	-
0	PAU8	YAL068C	1	-1	-
0	PAU9	YBL108C-A	1	-1	-
0	PBA1	YLR199C	-1	-	-
0	PBI2	YNL015W	1	1	1
1	**PBP2**	**YBR233W**	-	-1	-
0	PBP4	YDL053C	-	-1	-
0	PBS2	YJL128C	-	1	-
0	PCA1	YBR295W	1	-	-
0	PCC1	YKR095W-A	-1	-1	-
0	PCD1	YLR151C	1	-	1
0	PCL5	YHR071W	1	-	1
0	PCL8	YPL219W	-1	-	-
0	PCM1	YEL058W	1	-	-
1	**PCP1**	**YGR101W**	-1	-	-
0	PDC6	YGR087C	-	1	-
0	PDE1	YGL248W	1	1	1

0	PDE2	YOR360C	-	1	-
0	PDH1	YPR002W	1	1	1
0	PDR1	YGL013C	1	1	-
0	PDR10	YOR328W	1	1	-
0	PDR11	YIL013C	1	-1	-1
0	PDR12	YPL058C	-1	-	-1
0	PDR15	YDR406W	1	1	-
0	PDR16	YNL231C	-	-1	-
0	PDR18	YNR070W	-1	-	-1
0	PDR3	YBL005W	-	-	-1
0	PDR5	YOR153W	-	-	-1
0	PDR8	YLR266C	-	-1	-
1	**PDS5**	**YMR076C**	1	-	-
0	PEP12	YOR036W	1	-	-
0	PEP4	YPL154C	1	1	1
0	PES4	YFR023W	-	1	-
0	PET10	YKR046C	1	1	1
0	PET100	YDR079W	-	1	1
0	PET112	YBL080C	-	1	-
0	PET117	YER058W	-	1	1
0	PET123	YOR158W	-	1	1
0	PET127	YOR017W	-	1	-
0	PET191	YJR034W	-	1	1
0	PET309	YLR067C	-	-	1
0	PET494	YNR045W	-	-	1
0	PET54	YGR222W	-	-	1
0	PEX1	YKL197C	-	-	-1
0	PEX11	YOL147C	-	-	-1
0	PEX12	YMR026C	-	-1	-1
0	PEX13	YLR191W	-	-	-1
0	PEX17	YNL214W	-1	-	-
0	PEX18	YHR160C	-	1	-
0	PEX19	YDL065C	-	-1	-
0	PEX2	YJL210W	-1	-	-
0	PEX21	YGR239C	-	1	-1
0	PEX25	YPL112C	-	-	-1
0	PEX29	YDR479C	-	-	1
0	PEX30	YLR324W	-	1	-
0	PEX31	YGR004W	-1	-	-
0	PEX32	YBR168W	-1	-	-
0	PEX4	YGR133W	1	-	1
0	PEX7	YDR142C	-1	-	-
0	PEX8	YGR077C	-	1	-
0	PFA5	YDR459C	-1	-	-
0	PFK26	YIL107C	1	1	1
0	PFS2	YNL317W	-1	-	-
0	PGA2	YNL149C	-	-	-1
0	PGC1	YPL206C	-	1	1
1	**PGD1**	**YGL025C**	-1	-	-
0	PGM1	YKL127W	-	-	-1

0	PGM2	YMR105C	1	1	1
0	PGM3	YMR278W	-	1	1
0	PGU1	YJR153W	-1	-	1
0	PHA2	YNL316C	-1	-	-
0	PHB1	YGR132C	-	-	1
0	PHD1	YKL043W	-1	-	1
0	PHM6	YDR281C	-1	-1	-1
0	PHM7	YOL084W	-	1	-
0	PHM8	YER037W	1	1	1
0	PHO11	YAR071W	-1	-1	-1
0	PHO12	YHR215W	-1	-1	-1
0	PHO13	YDL236W	-1	-	-1
0	PHO3	YBR092C	-1	1	-1
0	PHO4	YFR034C	-1	-1	-1
0	PHO5	YBR093C	-1	-1	-1
1	**PHO80**	**YOL001W**	-1	-	-
0	PHO81	YGR233C	-1	-	-
0	PHO84	YML123C	-1	-	-
1	**PHO85**	**YPL031C**	-	-	1
0	PHO86	YJL117W	-1	-	-
1	**PHO87**	**YCR037C**	1	1	-
0	PHO90	YJL198W	-1	-	-1
0	PIB2	YGL023C	1	-	-1
0	PIC2	YER053C	1	1	1
1	**PIF1**	**YML061C**	-1	-	-
0	PIH1	YHR034C	-1	-	-
0	PIM1	YBL022C	-	1	-
0	PIN2	YOR104W	1	-	-
0	PIN3	YPR154W	1	-	1
0	PIN4	YBL051C	1	-	-
0	PIP2	YOR363C	-	-	-1
0	PIR3	YKL163W	1	1	1
0	PKH1	YDR490C	-	1	-
0	PKH2	YOL100W	1	1	-
1	**PKP1**	**YIL042C**	-	-	1
0	PKP2	YGL059W	1	1	1
0	PLB1	YMR008C	1	-	-
0	PLB2	YMR006C	1	-	1
0	PLB3	YOL011W	1	-	-
0	PLM2	YDR501W	-1	-	-
0	PLP2	YOR281C	-	-1	-
0	PMC1	YGL006W	1	1	1
0	PMD1	YER132C	1	-	-
0	PMT5	YDL093W	1	-	-
0	PNC1	YGL037C	-	1	1
0	PNG1	YPL096W	-	-1	-
0	PNO1	YOR145C	-1	-	-1
0	PNP1	YLR209C	-	-	1
0	PNS1	YOR161C	-1	1	-1
0	POA1	YBR022W	-	1	-

0	POB3	YML069W	-	-1	-
0	POF1	YCL047C	-	1	-
1	**POL12**	**YBL035C**	-1	-1	-
1	**POL30**	**YBR088C**	-1	-1	-
1	**POL32**	**YJR043C**	-	-1	-1
0	POL5	YEL055C	-	-1	-1
0	POM33	YLL023C	1	1	1
0	POP1	YNL221C	-	-	-1
0	POP3	YNL282W	-1	-	-
0	POP4	YBR257W	-	-	-1
0	POP5	YAL033W	-1	-	-
0	POP6	YGR030C	-1	-	-
1	**POP7**	**YBR167C**	-	-	-1
0	POR1	YNL055C	-	1	1
0	POR2	YIL114C	1	-1	-
0	POS5	YPL188W	-1	-	-
0	PPA1	YHR026W	-	-1	-
0	PPA2	YMR267W	-	1	-
1	**PPE1**	**YHR075C**	-	-	1
0	PPG1	YNR032W	-1	-	-
0	PPH3	YDR075W	-1	-	-
0	PPM2	YOL141W	-	-	-1
0	PPN1	YDR452W	1	-	1
0	PPT1	YGR123C	-1	-1	-1
0	PPT2	YPL148C	-	1	-
0	PPX1	YHR201C	-1	-1	-1
0	PPZ2	YDR436W	-	1	-
0	PRB1	YEL060C	1	1	1
0	PRC1	YMR297W	-	1	-
0	PRD1	YCL057W	1	-	-
0	PRE9	YGR135W	-	-1	-
0	PRI2	YKL045W	-	-1	-
0	PRM10	YJL108C	-	-	-1
0	PRM2	YIL037C	-	-	1
0	PRM3	YPL192C	-1	-	-
0	PRM4	YPL156C	1	-1	-
0	PRM5	YIL117C	1	-	-
0	PRM6	YML047C	-1	-1	-1
0	PRM7	YDL038C	1	-	-
0	PRM7	YDL039C	1	-	-1
0	PRM9	YAR031W	1	-	-1
1	**PRO1**	**YDR300C**	-	-	-1
0	PRP11	YDL043C	-1	-1	-1
0	PRP16	YKR086W	-1	-	-
0	PRP19	YLL036C	-1	-	-
0	PRP2	YNR011C	-	-	-1
0	PRP21	YJL203W	-	-	-1
0	PRP24	YMR268C	-1	-	-
0	PRP28	YDR243C	-	-	-1
1	**PRP31**	**YGR091W**	-	-	-1

1	**PRP4**	**YPR178W**	-	-	-1
1	**PRP43**	**YGL120C**	-	-	-1
0	PRP46	YPL151C	-1	-	-1
0	PRR1	YKL116C	-1	-	-
0	PRS1	YKL181W	-1	-1	-1
0	PRS2	YER099C	-1	-	-
1	**PRS3**	**YHL011C**	-	-	-1
0	PRS4	YBL068W	-1	-1	-1
1	**PRS5**	**YOL061W**	-1	-	-1
0	PRT1	YOR361C	-1	-	-1
0	PRX1	YBL064C	1	1	1
0	PRY1	YJL079C	1	-	1
0	PRY3	YJL078C	-	1	-
0	PSD1	YNL169C	-1	-	-
0	PSD2	YGR170W	1	-	-
0	PSE1	YMR308C	-1	-	-
0	PSF2	YJL072C	-1	-	-1
0	PSK1	YAL017W	-	1	1
0	PSO2	YMR137C	1	-	-
0	PSP1	YDR505C	-	1	-
0	PSP2	YML017W	1	-	-
0	PST1	YDR055W	1	1	-
0	PST2	YDR032C	1	1	1
1	**PSY4**	**YBL046W**	-1	-	-
1	**PTC1**	**YDL006W**	-1	-	-
0	PTC2	YER089C	1	-	-
0	PTH2	YBL057C	-1	-	-
0	PTK1	YKL198C	1	-	-
0	PTK2	YJR059W	1	1	1
0	PTM1	YKL039W	1	-	-
0	PTP2	YOR208W	1	1	-
0	PTP3	YER075C	-1	-	-
0	PTR2	YKR093W	1	-	-
0	PTR3	YFR029W	1	1	-
0	PUF2	YPR042C	1	1	-
0	PUF3	YLL013C	1	-	-
0	PUF6	YDR496C	-1	-	-1
0	PUN1	YLR414C	1	-	1
0	PUP1	YOR157C	-	-1	-
0	PUS1	YPL212C	-1	-1	-1
0	PUS4	YNL292W	-1	-1	-1
0	PUS5	YLR165C	-	-	1
0	PUS7	YOR243C	-	-1	-1
0	PUS9	YDL036C	-	-1	-1
0	PUT1	YLR142W	1	1	-
0	PUT2	YHR037W	-1	-	-
0	PWP1	YLR196W	-	-	-1
0	PWP2	YCR057C	-1	-	-1
0	PXA1	YPL147W	-	-	-1
0	PXL1	YKR090W	-	-	1

0	PYC1	YGL062W	1	1	1
0	PYC2	YBR218C	1	-	-
0	PYK2	YOR347C	1	1	1
0	QCR10	YHR001W-A	-1	1	1
0	QCR2	YPR191W	-	-	1
0	QCR7	YDR529C	-	-	1
0	QCR8	YJL166W	-	-	1
0	QCR9	YGR183C	-	-	1
0	QDR1	YIL120W	-	1	-
0	QDR2	YIL121W	-1	-1	-
0	QDR3	YBR043C	1	-	-
0	QRI5	YLR204W	-	1	1
0	RAD14	YMR201C	1	-	-
0	RAD16	YBR114W	-	1	-
0	RAD2	YGR258C	1	1	1
1	**RAD24**	**YER173W**	-	-1	-1
0	RAD28	YDR030C	1	1	1
0	RAD3	YER171W	-1	-1	-
0	RAD4	YER162C	-	-	1
1	**RAD50**	**YNL250W**	1	-	-
0	RAD51	YER095W	-	-	1
0	RAD52	YML032C	-1	-	-
0	RAD54	YGL163C	1	1	1
0	RAD7	YJR052W	1	-	-
0	RAI1	YGL246C	-1	-	-
0	RAM1	YDL090C	-1	-	-
0	RAM2	YKL019W	-1	-	-
1	**RAP1**	**YNL216W**	-1	-	-1
0	RAS1	YOR101W	-1	-1	-1
0	RAX1	YOR301W	-1	-	-
0	RBG1	YAL036C	-	-	-1
0	RBG2	YGR173W	-	-	-1
0	RBK1	YCR036W	1	-	-
0	RBS1	YDL189W	-	-1	-1
0	RCF1	YML030W	-	-	1
0	RCF2	YNR018W	-	1	-
0	RCL1	YOL010W	-1	-	-1
0	RCN2	YOR220W	1	1	1
0	RCR1	YBR005W	1	-	-
0	RCR2	YDR003W	-	-	1
0	RCS1	YGL071W	1	-	-
0	RCY1	YJL204C	1	-	-
0	RDL1	YOR285W	1	1	1
0	RDL2	YOR286W	-	1	-
0	RDR1	YOR380W	1	-	1
0	RDS1	YCR106W	-	-1	-
0	RDS2	YPL133C	-1	-	-1
0	REE1	YJL217W	1	-	-
1	**REF2**	**YDR195W**	-1	-1	-
0	REG1	YDR028C	1	-	-

0	REG2	YBR050C	1	-	1
0	REH1	YLR387C	-1	-1	-
0	REI1	YBR267W	-1	-	-1
0	RER1	YCL001W	-1	-	-
1	**RER2**	**YBR002C**	-1	-1	-
0	RET1	YOR207C	-	-	-1
0	REV3	YPL167C	-	1	-
0	REX4	YOL080C	-1	-1	-1
1	**RFA2**	**YNL312W**	-1	-	-
0	RFC4	YOL094C	-1	-	-
0	RFC5	YBR087W	-1	-1	-
0	RFS1	YBR052C	-	1	1
0	RFU1	YLR073C	-1	-	-1
0	RFX1	YLR176C	1	1	-
0	RGA1	YOR127W	1	-	-1
0	RGI1	YER067W	-	-	1
0	RGL1	YPL066W	-	1	-
0	RGM1	YMR182C	1	1	1
0	RGS2	YOR107W	-1	1	-
0	RGT2	YDL138W	1	-	-1
0	RHB1	YCR027C	-1	-	-
0	RHO1	YPR165W	-	-1	-
0	RHO3	YIL118W	-	-1	-
0	RHR2	YIL053W	-	-	-1
0	RIA1	YNL163C	-	-	-1
0	RIB7	YBR153W	-1	-	-
0	RIM101	YHL027W	-	1	-
0	RIM11	YMR139W	-	-	1
0	RIM15	YFL033C	-	1	1
0	RIM20	YOR275C	1	1	-
0	RIM21	YNL294C	1	-	-
0	RIM4	YHL024W	1	1	1
0	RIM8	YGL045W	-	-	1
0	RIO1	YOR119C	-1	-	-1
0	RIO2	YNL207W	-1	-	-1
0	RIP1	YEL024W	-	-	1
0	RIT1	YMR283C	-1	-	-1
0	RIX1	YHR197W	-1	-	-1
0	RIX7	YLL034C	-	-	-1
0	RKI1	YOR095C	-1	-1	-1
0	RKM5	YLR137W	-	1	-
0	RLI1	YDR091C	-1	-	-1
0	RLM1	YPL089C	1	1	-
0	RLP24	YLR009W	-	-	-1
0	RLP7	YNL002C	-	-	-1
0	RMA1	YKL132C	-1	-	-
0	RMD1	YDL001W	-1	-	-
0	RMD11	YHL023C	-	-	1
0	RMD6	YEL072W	-	1	-1
0	RMD8	YFR048W	-1	-	-

0	RME1	YGR044C	1	-	-
1	**RMI1**	**YPL024W**	-	1	1
0	RML2	YEL050C	-	1	1
0	RMR1	YGL250W	-	-	1
0	RMT2	YDR465C	-1	-1	-1
0	RNA1	YMR235C	-	-1	-
1	**RNA14**	**YMR061W**	-	-1	-
1	**RNA15**	**YGL044C**	-	-1	-
0	RNH1	YMR234W	-1	-	-1
1	**RNH201**	**YNL072W**	-1	-	-1
0	RNH202	YDR279W	-1	-1	-1
1	**RNR1**	**YER070W**	-	1	-
1	**RNR2**	**YJL026W**	1	1	-
0	RNR3	YIL066C	1	1	-
0	RNR4	YGR180C	1	-	-
1	**RNT1**	**YMR239C**	-1	-	-1
0	RNY1	YPL123C	1	1	1
0	ROD1	YOR018W	-	-	1
0	ROG1	YGL144C	-1	-	-
0	ROG3	YFR022W	1	1	-
0	ROK1	YGL171W	-1	-	-1
0	ROM1	YGR070W	1	1	1
0	ROT1	YMR200W	1	-	-
0	ROX1	YPR065W	-	-	1
0	ROY1	YMR258C	-1	-	-
0	RPA12	YJR063W	-	-	-1
0	RPA135	YPR010C	-	-	-1
1	**RPA14**	**YDR156W**	-1	-	-
0	RPA190	YOR341W	-	-	-1
0	RPA34	YJL148W	-	-	-1
0	RPA43	YOR340C	-1	-1	-1
0	RPA49	YNL248C	-1	-1	-1
0	RPB3	YIL021W	-	-1	-1
1	**RPB5**	**YBR154C**	-1	-	-
1	**RPB7**	**YDR404C**	-	-1	-
0	RPB8	YOR224C	-	-	-1
1	**RPB9**	**YGL070C**	-1	-1	-
1	**RPC10**	**YHR143W-A**	-	-	-1
0	RPC17	YJL011C	-1	-	-
0	RPC19	YNL113W	-	-	-1
0	RPC25	YKL144C	-	-	-1
0	RPC31	YNL151C	-	-1	-1
0	RPC34	YNR003C	-	-	-1
0	RPC37	YKR025W	-	-1	-1
0	RPC40	YPR110C	-1	-	-1
0	RPC53	YDL150W	-1	-	-1
0	RPC82	YPR190C	-	-	-1
0	RPF1	YHR088W	-	-	-1
0	RPF2	YKR081C	-1	-	-1
0	RPH1	YER169W	-	1	-

0	RPI1	YIL119C	-1	-	1
0	RPL15B	YMR121C	-	1	-
0	RPL18B	YNL301C	-	-1	-1
0	RPL22B	YFL034C-A	-1	-	-
0	RPM2	YML091C	-	-	1
0	RPN10	YHR200W	-1	-	-
0	RPN13	YLR421C	-	-1	-
0	RPN3	YER021W	-1	-	-
1	**RPN5**	**YDL147W**	-	-1	-
1	**RPN7**	**YPR108W**	-	-1	-
0	RPN8	YOR261C	-	-1	-
0	RPN9	YDR427W	-	-1	-
0	RPO26	YPR187W	-	-1	-1
0	RPP1	YHR062C	-1	-	-
0	RPR2	YIR015W	-	-1	-
0	RPS14B	YJL191W	-	1	-1
0	RPS18B	YML026C	-	-1	-
0	RPS22B	YLR367W	-	-1	-
0	RPS26B	YER131W	-	-1	-
0	RPS7B	YNL096C	-	-1	-
0	RPS9A	YPL081W	-1	-1	-1
1	**RPT5**	**YOR117W**	-	-1	-
0	RRB1	YMR131C	-1	-1	-1
0	RRF1	YHR038W	-	-	1
0	RRG1	YDR065W	-1	-	-
0	RRI1	YDL216C	-	-	1
0	RRI2	YOL117W	1	1	-
1	**RRM3**	**YHR031C**	-1	1	-
0	RRN10	YBL025W	-1	-	-
0	RRN11	YML043C	-1	-	-1
0	RRN3	YKL125W	-1	-	-
0	RRN6	YBL014C	-	-	-1
0	RRN7	YJL025W	-	-	-1
0	RRP1	YDR087C	-1	-	-1
0	RRP12	YPL012W	-	-	-1
1	**RRP17**	**YDR412W**	-1	-1	-1
0	RRP3	YHR065C	-1	-	-1
0	RRP4	YHR069C	-1	-	-1
0	RRP40	YOL142W	-1	-	-1
0	RRP42	YDL111C	-1	-	-1
0	RRP43	YCR035C	-1	-1	-1
0	RRP45	YDR280W	-1	-1	-1
0	RRP46	YGR095C	-1	-	-1
0	RRP5	YMR229C	-	-	-1
0	RRP6	YOR001W	-	-	-1
0	RRP7	YCL031C	-	-1	-1
1	**RRP8**	**YDR083W**	-	-1	-1
0	RRP9	YPR137W	-1	-	-1
0	RRS1	YOR294W	-1	-	-1
0	RRT14	YIL127C	-	-	-1

0	RRT2	YBR246W	-1	-	-1
0	RRT8	YOL048C	-	-	1
0	RSA3	YLR221C	-	-	-1
0	RSA4	YCR072C	-1	-	-1
0	RSB1	YOR049C	-1	1	1
0	RSC30	YHR054C	1	1	1
0	RSC30	YHR056C	1	1	1
0	RSC58	YLR033W	-	-1	-
1	**RSC8**	**YFR037C**	-	-1	-
0	RSC9	YML127W	-	-1	-
0	RSF2	YJR127C	-	1	1
1	**RSM10**	**YDR041W**	-	-	1
0	RSM18	YER050C	-	1	1
0	RSM19	YNR037C	-	1	1
0	RSM22	YKL155C	-	1	1
0	RSM24	YDR175C	-1	-	-
0	RSM26	YJR101W	-1	1	1
0	RSM27	YGR215W	-	1	1
0	RSM7	YJR113C	-	1	-
0	RSN1	YMR266W	-	-1	-
0	RSR1	YGR152C	-	-1	-
0	RTA1	YGR213C	1	-	-1
0	RTC2	YBR147W	1	1	1
0	RTC3	YHR087W	-	1	1
0	RTC5	YOR118W	-	-1	-1
0	RTC6	YPL183W-A	-	1	1
1	**RTF1**	**YGL244W**	-	-1	-1
0	RTG2	YGL252C	-	-1	-
0	RTK1	YDL025C	1	-	1
0	RTN2	YDL204W	-	1	-
0	RTR1	YER139C	-	-1	-
0	RTR2	YDR066C	-	-	-1
0	RTS1	YOR014W	1	-	-
0	RTS3	YGR161C	1	-	1
0	RTT10	YPL183C	-1	-	-1
0	RTT103	YDR289C	-	-1	-
0	RTT106	YNL206C	-	-1	-1
0	RTT107	YHR154W	1	-	-
0	RTT109	YLL002W	-1	-1	-
0	RUD3	YOR216C	-	-1	-
0	RVB1	YDR190C	-	-	-1
0	RVB2	YPL235W	-1	-1	-1
0	RXT2	YBR095C	-	-1	-
0	SAC6	YDR129C	1	1	-
1	**SAD1**	**YFR005C**	-	-1	-1
0	SAE2	YGL175C	-	-	-1
0	SAG1	YJR004C	1	-	-
0	SAM1	YLR180W	-1	-1	-
0	SAM2	YDR502C	-	-	-1
0	SAM3	YPL274W	-1	-1	-1

0	SAM4	YMR321C	-1	-	-1
0	SAM4	YPL273W	-1	-1	-1
1	**SAN1**	**YDR143C**	-1	-	-
0	SAP185	YJL098W	-	-	-1
1	**SAP30**	**YMR263W**	-1	-	-
0	SAS10	YDL153C	-	-	-1
0	SAS2	YMR127C	-1	-	-
0	SAS5	YOR213C	-	-1	-1
0	SAW1	YAL027W	-	-1	-1
0	SBE2	YDR351W	-	-	-1
0	SCH9	YHR205W	-1	-	-1
0	SCM3	YDL139C	-	-	1
0	SCM4	YGR049W	1	-	-
0	SCO2	YBR024W	1	-	1
0	SCS22	YBL091C-A	1	-	1
0	SCS7	YMR272C	-	-	1
0	SCW11	YGL028C	-1	1	-
0	SCW4	YGR279C	-	-	1
0	SDA1	YGR245C	-	-1	-1
0	SDH1	YKL148C	-	1	1
0	SDH2	YLL041C	-	-	1
0	SDH3	YKL141W	-	-	1
0	SDH4	YDR178W	-	-	1
0	SDO1	YLR022C	-1	-	-
0	SDS22	YKL193C	-	-	1
0	SDS24	YBR214W	1	1	1
0	SDS3	YIL084C	-1	-	-
0	SDT1	YGL224C	-1	-	-
0	SEC13	YLR208W	-	-1	-
0	SEC14	YMR079W	-	-	-1
0	SEC16	YPL085W	-	1	-
0	SEC17	YBL050W	-	-1	-
0	SEC18	YBR080C	1	-	-
0	SEC2	YNL272C	1	-	-
1	**SEC20**	**YDR498C**	-1	-1	-
0	SEC53	YFL045C	-	-1	-
0	SEC62	YPL094C	-	-	-1
1	**SEC63**	**YOR254C**	-	-	-1
0	SEC65	YML105C	-1	-	-
0	SEC8	YPR055W	-	1	-
0	SEC9	YGR009C	1	-	-
0	SED1	YDR077W	1	1	1
0	SED4	YCR067C	1	-	-
0	SEE1	YIL064W	-1	-	-1
0	SEG1	YMR086W	1	-	-
0	SEH1	YGL100W	-1	-1	-1
0	SEL1	YML013W	1	-	-
0	SEN15	YMR059W	-1	-	-
0	SEN2	YLR105C	-1	-1	-1
0	SEN34	YAR008W	-	-1	-1

1	**SEN54**	**YPL083C**	-	-1	-1
0	SEO1	YAL067C	1	1	-
0	SER1	YOR184W	1	-1	-
0	SER2	YGR208W	1	-1	-1
0	SER3	YER081W	1	-	-
0	SER33	YIL074C	-1	-	-
0	SES1	YDR023W	-	-1	-1
1	**SET1**	**YHR119W**	-	1	-
0	SET4	YJL105W	1	-	-
0	SET6	YPL165C	-	-	1
0	SFA1	YDL168W	-1	1	-
0	SFB2	YNL049C	-	-	-1
0	SFG1	YOR315W	-1	-1	-
0	SFH1	YLR321C	-	-1	-
0	SFL1	YOR140W	-1	-	-
0	SFP1	YLR403W	-	1	-
0	SGA1	YIL099W	-	1	1
0	SGD1	YLR336C	-1	-	-1
0	SGE1	YPR198W	1	-	-
0	SGF11	YPL047W	-	-	-1
0	SGF29	YCL010C	1	-	-
0	SGN1	YIR001C	-	-	1
0	SGO1	YOR073W	-	-	1
0	SGS1	YMR190C	-	1	-
0	SGT2	YOR007C	-	1	-
0	SHE2	YKL130C	-	-	1
0	SHE3	YBR130C	-	-1	-
0	SHE9	YDR393W	-1	-	-
0	SHM2	YLR058C	1	-	-
0	SHQ1	YIL104C	-1	-	-1
0	SHR3	YDL212W	-	-1	-
0	SHU2	YDR078C	-1	-	-
0	SHY1	YGR112W	-	1	1
0	SIA1	YOR137C	1	1	1
0	SIK1	YLR197W	-	-1	-1
0	SIL1	YOL031C	-1	-	1
1	**SIN3**	**YOL004W**	1	1	-
0	SIP2	YGL208W	-	-	1
0	SIP4	YJL089W	1	1	1
0	SIR1	YKR101W	1	-	-
0	SIR2	YDL042C	-1	-	-
0	SIT1	YEL065W	1	-	-1
0	SKI8	YGL213C	-1	-1	-1
0	SKS1	YPL026C	1	-	1
0	SLA1	YBL007C	1	-	-
1	**SLA2**	**YNL243W**	1	-	-
0	SLD5	YDR489W	-1	-	-
0	SLF1	YDR515W	1	-	1
0	SLI1	YGR212W	1	-	-
0	SLM3	YDL033C	-	-	-1

0	SLM4	YBR077C	1	-	-
0	SLM5	YCR024C	-	1	1
0	SLP1	YOR154W	-1	-	-1
0	SLT2	YHR030C	1	-	-
0	SLX4	YLR135W	-	-	1
0	SLX9	YGR081C	-1	-	-
0	SMC4	YLR086W	1	-	-
0	SMD1	YGR074W	-1	-	-1
0	SMD3	YLR147C	-	-	-1
0	SMF1	YOL122C	-	1	-
0	SMF3	YLR034C	1	-	1
0	SMK1	YPR054W	-	1	-1
0	SMM1	YNR015W	-1	-	-1
0	SMP1	YBR182C	1	-	-
0	SMX2	YFL017W-A	-	1	-
0	SMY1	YKL079W	-	1	-
0	SMY2	YBR172C	-1	-	-
0	SNA2	YDR525W-A	1	-	1
0	SNA3	YJL151C	1	1	1
0	SNA4	YDL123W	1	-	-
0	SNC1	YAL030W	-	1	-
1	**SNC2**	**YOR327C**	1	-	-
0	SNF11	YDR073W	1	-	-1
0	SNF12	YNR023W	-1	-	-
0	SNF2	YOR290C	-	1	-
0	SNF3	YDL194W	-	-	1
1	**SNF7**	**YLR025W**	1	-	-
0	SNM1	YDR478W	-	1	-1
0	SNO1	YMR095C	1	-	1
0	SNO2	YNL334C	1	-	-
0	SNO3	YFL060C	1	-	-
0	SNQ2	YDR011W	-	-	-1
0	SNU13	YEL026W	-	-	-1
0	SNU56	YDR240C	-1	-	-
0	SNX4	YJL036W	-	-1	-
0	SNX41	YDR425W	1	1	-
0	SNZ1	YMR096W	1	-	1
0	SOD2	YHR008C	1	1	1
0	SOF1	YLL011W	-1	-	-1
1	**SOH1**	**YGL127C**	-1	-	-
0	SOK1	YDR006C	-	1	-
0	SOK2	YMR016C	-	-	-1
1	**SOL2**	**YCR073W-A**	-	1	1
0	SOL3	YHR163W	-	-	-1
0	SOL4	YGR248W	1	1	1
0	SOM1	YEL059C-A	1	1	1
0	SPA2	YLL021W	1	-	-
0	SPC1	YJR010C-A	-	-1	-
0	SPC105	YGL093W	-	1	-
0	SPC29	YPL124W	-1	-	-

0	SPE1	YKL184W	-	-1	-1
0	SPE2	YOL052C	-1	-	-1
0	SPE3	YPR069C	-	-	-1
0	SPE4	YLR146C	-1	-1	-1
0	SPG5	YMR191W	-	1	1
0	SPI1	YER150W	-	1	1
0	SPL2	YHR136C	-1	-1	-
1	**SPN1**	**YPR133C**	-1	-	-
0	SPO1	YNL012W	1	-	-1
0	SPO12	YHR152W	1	-1	-
0	SPO14	YKR031C	1	-	-
0	SPO16	YHR153C	1	-	-
0	SPO20	YMR017W	1	-	-
0	SPO22	YIL073C	1	1	-
0	SPO23	YBR250W	-	1	-
0	SPO7	YAL009W	-	-	-1
0	SPO74	YGL170C	-	1	-
0	SPR3	YGR059W	1	1	-
0	SPS19	YNL202W	-	1	-
0	SPS4	YOR313C	-	-1	-
0	SPT5	YML010W	-	-1	-1
0	SQT1	YIR012W	-	-	-1
1	**SRB5**	**YGR104C**	-1	-	-
0	SRB6	YBR253W	1	-	-
0	SRD1	YCR018C	-	1	1
0	SRF1	YDL133W	-1	-1	-
0	SRL1	YOR247W	-	-1	1
0	SRL2	YLR082C	-	-1	-
0	SRL3	YKR091W	1	1	1
0	SRM1	YGL097W	-	-	-1
0	SRO77	YBL106C	1	-	-1
0	SRP101	YDR292C	-	-1	-1
0	SRP102	YKL154W	-	-1	-
0	SRP14	YDL092W	-	-1	-
0	SRP21	YKL122C	-	-1	-
0	SRP40	YKR092C	-	-	-1
0	SRP54	YPR088C	-	-	-1
0	SRP72	YPL210C	-	-1	-
0	SRT1	YMR101C	1	1	-
0	SRV2	YNL138W	1	-	-
0	SRX1	YKL086W	1	1	1
0	SRY1	YKL218C	1	1	-
0	SSA1	YAL005C	1	1	1
0	SSA4	YER103W	-	1	1
0	SSC1	YJR045C	-	1	-
0	SSD1	YDR293C	1	1	1
0	SSE2	YBR169C	1	1	1
0	SSF1	YHR066W	-	-	-1
0	SSF2	YDR312W	-	-	-1
0	SSH4	YKL124W	-	-	1

0	SSK2	YNR031C	-	-	1
0	SSK22	YCR073C	1	1	-
1	**SSN3**	**YPL042C**	-1	-	-
0	SSO2	YMR183C	-	-1	-1
0	SST2	YLR452C	-1	-1	-1
0	SSU1	YPL092W	1	1	1
0	SSU72	YNL222W	-1	-	-
0	SSZ1	YHR064C	-	-	-1
0	STB2	YMR053C	1	1	1
0	STB3	YDR169C	1	1	1
0	STB5	YHR178W	1	-	-
0	STB6	YKL072W	1	-	-
0	STE12	YHR084W	-	-1	-
0	STE18	YJR086W	-	-	1
0	STE2	YFL026W	-1	-1	-
0	STE23	YLR389C	-	-	1
0	STE3	YKL178C	1	1	1
0	STE4	YOR212W	-1	-1	-1
0	STE5	YDR103W	-	1	-
0	STE50	YCL032W	-	-1	-1
0	STE6	YKL209C	-1	-1	-
0	STF1	YDL130W-A	1	1	1
0	STF2	YGR008C	1	1	1
0	STI1	YOR027W	-	1	-
1	**STN1**	**YDR082W**	1	-	-
0	STP2	YHR006W	1	1	-
0	STP3	YLR375W	1	1	-
0	STP4	YDL048C	-	1	1
0	STR3	YGL184C	-	1	-
0	STT3	YGL022W	1	-	-
0	STT4	YLR305C	-	1	-
0	STU1	YBL034C	-	1	-
1	**SUA5**	**YGL169W**	-1	-	-1
0	SUB1	YMR039C	-	-1	-
0	SUC2	YIL162W	1	-	-
0	SUI1	YNL244C	-1	-	-
0	SUI2	YJR007W	-	-1	-1
0	SUI3	YPL237W	-	-1	-
0	SUP45	YBR143C	-	-1	-1
1	**SUR4**	**YLR372W**	-	-1	-
0	SUS1	YBR111W-A	-	-	1
0	SUT1	YGL162W	-1	-1	-1
0	SUT2	YPR009W	1	-	-1
0	SUV3	YPL029W	-	1	-
0	SVF1	YDR346C	-	-1	-1
0	SVS1	YPL163C	-1	-	-
1	**SWD1**	**YAR003W**	-1	-1	-
0	SWD2	YKL018W	-1	-	-
1	**SWD3**	**YBR175W**	-1	-	-
0	SWH1	YAR042W	1	1	-

0	SWI1	YPL016W	1	-	-
0	SWI4	YER111C	1	1	1
0	SWM1	YDR260C	-	-1	-
0	SWM2	YNR004W	-	-	1
0	SWS2	YNL081C	-	-	1
0	SWT1	YOR166C	-	-	-1
0	SWT21	YNL187W	-	1	-
0	SYF1	YDR416W	-1	-	-1
0	SYF2	YGR129W	1	-	-
0	SYG1	YIL047C	-	1	-
0	SYG1	YIL047C-A	-	1	-
0	SYM1	YLR251W	1	1	1
0	SYP1	YCR030C	-	-	1
0	SYS1	YJL004C	-	-1	-
0	TAD2	YJL035C	-1	-	-
0	TAD3	YLR316C	-	-	-1
0	TAF12	YDR145W	-	-1	-
0	TAF13	YML098W	-	-	-1
0	TAF14	YPL129W	-	-1	-
0	TAF9	YMR236W	-1	-	-
0	TAH1	YCR060W	1	1	-
0	TAH11	YJR046W	-1	-	-1
0	TAH18	YPR048W	-	-	-1
0	TAN1	YGL232W	-	-1	-1
0	TAT1	YBR069C	-1	-	-
0	TAZ1	YPR140W	-	-	1
0	TCA17	YEL048C	-1	-	-1
0	TCB1	YOR086C	1	-	1
0	TCB2	YNL087W	-	-1	-1
0	TCB3	YML072C	-	-	1
0	TCM10	YDR350C	1	1	-
0	TDA1	YMR291W	1	-	-
0	TDA10	YGR205W	-	-	1
0	TDA11	YHR159W	1	-	-
0	TDA2	YER071C	1	-	-
0	TDA3	YHR009C	1	1	1
0	TDA4	YJR116W	1	-	-
0	TDA6	YPR157W	1	-	-
0	TDA7	YNL176C	1	-	-
0	TDA9	YML081W	-1	-	-
0	TDH1	YJL052W	1	1	1
0	TEA1	YOR337W	-	-	-1
0	TEC1	YBR083W	-1	-	-
1	**TEL2**	**YGR099W**	-1	-	-
0	TEM1	YML064C	-1	-1	-
1	**TEN1**	**YLR010C**	-	-	1
0	TFB1	YDR311W	-	1	-
0	TFB2	YPL122C	-1	-	-
0	TFB3	YDR460W	-1	-	-
0	TFB4	YPR056W	-1	-1	-

0	TFC1	YBR123C	-1	-1	-
0	TFG2	YGR005C	-	-1	-1
0	TFS1	YLR178C	1	1	1
0	TGL1	YKL140W	-	-	1
0	TGL2	YDR058C	1	1	1
0	TGL3	YMR313C	-	-	1
0	TGL5	YOR081C	-	-	1
1	**TGS1**	**YPL157W**	-	-	-1
0	THG1	YGR024C	-1	-	-
0	THI11	YJR156C	1	1	1
0	THI12	YNL332W	1	1	1
0	THI13	YDL244W	1	1	1
0	THI2	YBR240C	1	1	1
0	THI20	YOL055C	1	1	-
0	THI5	YFL058W	1	1	1
0	THI6	YPL214C	1	1	-
0	THI7	YLR237W	1	1	1
0	THI80	YOR143C	-1	-1	-
0	THO1	YER063W	1	-1	1
1	**THP2**	**YHR167W**	-1	-	-
0	THP3	YPR045C	-	1	-
0	THS1	YIL078W	-	-1	-1
0	TIF3	YPR163C	-	-1	-1
0	TIF34	YMR146C	-	-1	-1
0	TIF35	YDR429C	-	-1	-1
0	TIF4631	YGR162W	-	-1	-1
0	TIF5	YPR041W	-	-1	-1
0	TIF6	YPR016C	-1	-1	-1
0	TIM18	YOR297C	-1	-	-
0	TIM21	YGR033C	-1	-1	-
0	TIM8	YJR135W-A	-	1	-
0	TIM9	YEL020W-A	-	1	-
0	TIP1	YBR067C	-	1	1
0	TIP41	YPR040W	-	-	1
0	TIR1	YER011W	1	1	1
0	TIR2	YOR010C	1	-	1
0	TIR3	YIL011W	1	-1	-
0	TIR4	YOR009W	1	1	1
0	TIS11	YLR136C	1	-	-
0	TLG2	YOL018C	-	-1	-
0	TMA10	YLR327C	1	1	1
0	TMA16	YOR252W	-1	-	-1
0	TMA17	YDL110C	1	1	1
0	TMA22	YJR014W	-	-1	-1
1	**TMA23**	**YMR269W**	-	-	-1
0	TMA46	YOR091W	-	-	-1
0	TMA64	YDR117C	-1	-1	-
0	TMT1	YER175C	1	-	-
0	TNA1	YGR260W	-	1	-
0	TOD6	YBL054W	-1	-	-1

0	TOF2	YKR010C	-	-	1
0	TOM7	YNL070W	-	1	-
0	TOP2	YNL088W	1	-	-
0	TOS1	YBR162C	-	-1	-
0	TOS2	YGR221C	-1	-1	1
0	TOS4	YLR183C	-	-1	-
0	TOS6	YNL300W	-1	-	-
0	TPA1	YER049W	-	-	-1
0	TPC1	YGR096W	-1	-1	-
0	TPK1	YJL164C	1	-	1
0	TPK2	YPL203W	-	-	1
0	TPK3	YKL166C	-	-1	-1
0	TPM1	YNL079C	-	-	1
0	TPM2	YIL138C	1	-	-
0	TPO1	YLL028W	1	1	1
0	TPO2	YGR138C	-	1	1
0	TPO3	YPR156C	1	1	1
0	TPO4	YOR273C	-	1	-
0	TPS1	YBR126C	-	1	1
0	TPS2	YDR074W	-	1	1
0	TPS3	YMR261C	1	1	1
0	TPT1	YOL102C	-	-1	-
0	TRA1	YHR099W	1	1	-
0	TRE1	YPL176C	1	1	-
0	TRF5	YNL299W	-	-	-1
0	TRI1	YMR233W	-	-1	-
1	**TRK1**	**YJL129C**	-	-	1
0	TRM1	YDR120C	-	-1	-1
0	TRM10	YOL093W	-1	-1	-1
0	TRM11	YOL124C	-	-	-1
0	TRM112	YNR046W	-1	-	-1
0	TRM12	YML005W	-	-	-1
0	TRM13	YOL125W	-	-	-1
0	TRM2	YKR056W	-1	-	-1
0	TRM3	YDL112W	-	-	-1
0	TRM44	YPL030W	-1	-	-1
1	**TRM5**	**YHR070W**	-1	-	-1
0	TRM7	YBR061C	-1	-1	-1
0	TRM8	YDL201W	-1	-1	-1
0	TRM82	YDR165W	-1	-	-1
0	TRM9	YML014W	-	-	-1
0	TRP2	YER090W	-	-	-1
0	TRP3	YKL211C	-	-	-1
0	TRR1	YDR353W	-	-	-1
0	TRR2	YHR106W	-1	-	-
1	**TRS20**	**YBR254C**	-1	-	-
0	TRS31	YDR472W	-1	-	-1
0	TRS33	YOR115C	-1	-	-
0	TRX3	YCR083W	-	1	-
0	TRZ1	YKR079C	-	-	-1

0	TSA2	YDR453C	1	1	-
0	TSC10	YBR265W	-1	-	-
0	TSL1	YML100W	1	1	1
1	**TSR1**	**YDL060W**	-1	-	-1
0	TSR2	YLR435W	-1	-1	-1
0	TSR3	YOR006C	-1	-1	-1
0	TSR4	YOL022C	-1	-1	-1
0	TTI1	YKL033W	-	-	-1
0	TTI2	YJR136C	-1	-	-
0	TUF1	YOR187W	-	1	1
0	TUM1	YOR251C	-1	-	-
0	TUS1	YLR425W	-	1	-
0	TVP15	YDR100W	-	-	1
0	TYE7	YOR344C	-1	-	-
0	TYR1	YBR166C	-	-1	-
0	TYW1	YPL207W	-	-1	-1
0	TYW3	YGL050W	-1	-	-1
0	UBA1	YKL210W	1	-	-
0	UBC4	YBR082C	-	-	1
0	UBC5	YDR059C	-	1	1
0	UBC8	YEL012W	1	1	1
0	UBC9	YDL064W	-1	-	-
0	UBI4	YLL039C	1	1	1
0	UBP11	YKR098C	1	1	-
0	UBP14	YBR058C	1	-	-
0	UBP15	YMR304W	1	1	-
0	UBP16	YPL072W	1	1	1
0	UBP2	YOR124C	1	1	1
0	UBP5	YER144C	1	1	-
0	UBP8	YMR223W	-1	-	-
0	UBR1	YGR184C	-	1	-
0	UBS1	YBR165W	-1	-	-
0	UBX3	YDL091C	1	-	-
0	UBX6	YJL048C	-	1	1
0	UFD2	YDL190C	1	-	-
0	UFE1	YOR075W	-	-1	-
0	UGA1	YGR019W	1	1	-
0	UGA2	YBR006W	1	1	1
0	UGP1	YKL035W	-	-	1
0	UGX2	YDL169C	1	1	1
0	UIP3	YAR027W	1	-	1
0	UIP4	YPL186C	1	1	1
0	UIP5	YKR044W	-	-	-1
0	UME1	YPL139C	-1	-	-
0	UME6	YDR207C	-	-	-1
0	UPC2	YDR213W	1	-	-
0	UPS1	YLR193C	-	-	1
0	UPS2	YLR168C	-	-	1
0	UPS3	YDR185C	-1	-1	-
0	URA10	YMR271C	-	-	1

0	URA4	YLR420W	-	-1	-
0	URA5	YML106W	-	-1	-
0	URA6	YKL024C	-1	-	-
0	URA7	YBL039C	-1	-1	-1
0	URA8	YJR103W	-	1	-
0	URB1	YKL014C	-	-	-1
0	URB2	YJR041C	-	-	-1
1	**URE2**	**YNL229C**	-	-1	-
0	URK1	YNR012W	-	-	-1
0	URM1	YIL008W	-	-	-1
0	USB1	YLR132C	1	-	-
0	USE1	YGL098W	-	-1	-
0	USO1	YDL058W	1	-	-
0	UTP11	YKL099C	-	-	-1
0	UTP13	YLR222C	-	-	-1
0	UTP15	YMR093W	-1	-	-1
0	UTP18	YJL069C	-1	-	-1
0	UTP20	YBL004W	-	-	-1
0	UTP21	YLR409C	-1	-	-1
0	UTP22	YGR090W	-1	-	-
0	UTP23	YOR004W	-1	-1	-1
0	UTP25	YIL091C	-1	-	-1
0	UTP30	YKR060W	-1	-	-1
0	UTP4	YDR324C	-	-	-1
0	UTP5	YDR398W	-1	-	-1
0	UTP6	YDR449C	-1	-	-1
0	UTP7	YER082C	-	-	-1
0	UTP8	YGR128C	-1	-	-1
0	UTP9	YHR196W	-1	-	-1
0	UTR2	YEL040W	-	-1	-1
0	UTR4	YEL038W	-	-	-1
0	VAC17	YCL063W	-	-1	-
0	VAM3	YOR106W	-	-1	-1
1	**VAM6**	**YDL077C**	-	1	-
0	VBA1	YMR088C	1	1	-
0	VBA2	YBR293W	1	-	-
0	VCX1	YDL128W	-1	-	-1
0	VEL1	YGL258W	-1	-	-1
0	VFA1	YER128W	1	-	-
0	VHR1	YIL056W	1	1	1
0	VHS3	YOR054C	-	-	1
1	**VHT1**	**YGR065C**	-	-	-1
0	VID24	YBR105C	1	1	1
0	VID28	YIL017C	1	-	1
0	VID30	YGL227W	1	1	1
0	VIP1	YLR410W	-	1	-
0	VMA10	YHR039C-A	-	-	1
0	VMR1	YHL035C	1	-	-
0	VPH2	YKL119C	-	-1	-
0	VPS24	YKL041W	-	-1	-

1	**VPS28**	**YPL065W**	-	-	1
1	**VPS3**	**YDR495C**	1	-	-
1	**VPS34**	**YLR240W**	-	1	-
0	VPS45	YGL095C	1	-	-
0	VPS51	YKR020W	1	-	-
0	VPS55	YJR044C	-	-	1
0	VPS60	YDR486C	-	-1	-
0	VPS62	YGR141W	1	-	-1
0	VPS64	YDR200C	1	-	-
0	VPS66	YPR139C	-	-1	-
0	VPS71	YML041C	-1	-	-
0	VPS73	YGL104C	1	-	1
0	VPS74	YDR372C	-	-1	-
1	**VPS75**	**YNL246W**	-1	-1	-
0	VPS8	YAL002W	-	1	1
1	**VPS9**	**YML097C**	-1	-	-
1	**VRP1**	**YLR337C**	1	1	-
0	VTA1	YLR181C	-	-1	-1
0	VTC1	YER072W	-1	-	-
0	VTC2	YFL004W	-1	-1	-
0	VTC3	YPL019C	-1	-1	-
0	VTC4	YJL012C	-1	-1	-
0	VTS1	YOR359W	-1	-	-1
0	WRS1	YOL097C	-	-1	-1
0	WSC3	YOL105C	-	-1	-
0	WSC4	YHL028W	-1	-	1
0	WTM1	YOR230W	1	1	1
0	WTM2	YOR229W	1	-	-
0	WWM1	YFL010C	-	1	-
0	XBP1	YIL101C	1	1	-
0	XKS1	YGR194C	-	-	1
0	XPT1	YJR133W	-	1	-
0	XYL2	YLR070C	-	-	-1
0	YAK1	YJL141C	1	1	1
0	YAP1	YML007W	1	-	-
0	YAP1801	YHR161C	1	1	1
0	YAP6	YDR259C	1	-	-
0	YAR1	YPL239W	-	-	-1
0	YAT1	YAR035W	1	1	-
0	YAT2	YER024W	1	-	-
0	YCK1	YHR135C	1	-1	-
0	YCT1	YLL055W	1	-	1
0	YDC1	YPL087W	1	1	1
1	**YDJ1**	**YNL064C**	-1	-	-1
0	YEA4	YEL004W	1	-	-
0	YEA6	YEL006W	-1	-	-
0	YEF1	YEL041W	1	1	-
0	YEH1	YLL012W	1	-	-1
0	YET1	YKL065C	1	-	1
0	YET2	YMR040W	-	1	-

0	YET3	YDL072C	1	-	-
0	YFH7	YFR007W	-	1	-
0	YGK3	YOL128C	-1	-	-1
0	YGP1	YNL160W	1	1	1
0	YHK8	YHR048W	-	1	-
0	YHP1	YDR451C	-	-	1
1	**YKU70**	**YMR284W**	1	-	-
0	YLH47	YPR125W	-1	-	-
0	YMC1	YPR058W	-	-	-1
0	YMC2	YBR104W	-	-1	-1
0	YMD8	YML038C	-	-1	-
0	YME1	YPR024W	1	1	-
0	YME2	YMR302C	-	1	1
0	YML6	YML025C	-	-	1
0	YND1	YER005W	-1	-	-
0	YNK1	YKL067W	1	-	-
0	YOS9	YDR057W	1	1	-
0	YOX1	YML027W	-	-	1
0	YPC1	YBR183W	-	-	1
0	YPK2	YMR104C	1	1	-
0	YPK3	YBR028C	-1	-1	-
0	YPK9	YOR291W	-	-	1
0	YPS1	YLR120C	1	1	-
0	YPS3	YLR121C	1	1	1
0	YPS6	YIR039C	1	1	1
0	YPT10	YBR264C	-1	-	-
0	YPT31	YER031C	-	-1	-
0	YPT32	YGL210W	-	-1	-
0	YPT52	YKR014C	-	-1	-
0	YPT53	YNL093W	1	1	1
0	YPT6	YLR262C	-	-1	-
0	YRA1	YDR381W	-	-1	-
0	YRF1-1	YBL111C	-	-1	-1
0	YRF1-1	YBL113C	-	-1	-1
0	YRF1-1	YDR545W	-	-1	-1
0	YRF1-2	YBL112C	-	-1	-1
0	YRF1-2	YDR545W	-	-1	-1
0	YRF1-2	YEL076C-A	-	-1	-
0	YRF1-3	YDR545W	-	-1	-1
0	YRF1-3	YEL077C	-	-1	-1
0	YRF1-3	YER190W	-	-1	-1
0	YRF1-4	YEL077C	-	-1	-1
0	YRF1-4	YER190W	-	-1	-1
0	YRF1-4	YGR296W	-	-1	-1
0	YRF1-4	YLL066C	-	-1	-1
0	YRF1-4	YLR466W	-	-1	-1
0	YRF1-5	YER190W	-	-1	-1
0	YRF1-5	YFL064C	-	-	-1
0	YRF1-5	YFL065C	-	-	-1
0	YRF1-5	YFL066C	-	-1	-

0	YRF1-5	YGR296W	-	-1	-1
0	YRF1-5	YIL177C	-	-1	-1
0	YRF1-5	YLL067C	-	-1	-1
0	YRF1-5	YLR467W	-	-1	-1
0	YRF1-6	YGR296W	-	-1	-1
0	YRF1-6	YHL050C	-	-1	-
0	YRF1-6	YJL225C	-	-1	-1
0	YRF1-6	YNL339C	-	-1	-1
0	YRF1-7	YHL049C	-	-	-1
0	YRF1-7	YHL050C	-	-1	-
0	YRF1-7	YHR218W-A	-	-1	-1
0	YRF1-7	YHR219W	-	-1	-1
0	YRF1-7	YLR464W	-	-1	-
0	YRF1-7	YPL283C	-	-1	-1
0	YRF1-8	YOR396W	-	-1	-1
0	YRO2	YBR054W	-	1	1
0	YSP1	YHR155W	-	1	-
1	**YSP3**	**YOR003W**	-	-	-1
0	YSR3	YKR053C	1	-	-
0	YSW1	YBR148W	1	-	1
0	YTA12	YMR089C	-	-	1
1	**YTA7**	**YGR270W**	1	-	-
0	YTH1	YPR107C	-1	-	-
0	YTM1	YOR272W	-1	-	-1
0	YTP1	YNL237W	1	1	1
0	YUR1	YJL139C	-	-	-1
0	YVH1	YIR026C	-1	-1	-1
0	ZAP1	YJL056C	-	-	-1
0	ZDS1	YMR273C	-	1	1
0	ZIM17	YNL310C	-	1	-
0	ZIP1	YDR285W	-	-	-1
0	ZPR1	YGR211W	-1	-1	-1
0	ZPS1	YOL154W	-1	-	-1
0	ZRC1	YMR243C	-	-	-1
0	ZRG8	YER033C	1	1	1
0	ZRT1	YGL255W	-	-1	-1
0	ZRT2	YLR130C	-1	-	-1
0	ZTA1	YBR046C	1	-	-
0	ZUO1	YGR285C	-	-	-1
0	-	YAL016C-B	-	-	1
0	-	YAL063C-A	1	1	1
0	-	YAR009C	1	-	1
0	-	YAR010C	1	-	-
0	-	YAR028W	1	-	1
0	-	YAR029W	1	1	1
0	-	YAR070C	-1	-	-
0	-	YBL005W-A	1	1	-1
0	-	YBL005W-B	1	1	1
0	-	YBL010C	-	-	1
0	-	YBL028C	-	-	-1

0 -	YBL029C-A	-1	-	1
0 -	YBL029W	-	-	1
0 -	YBL039W-B	1	-	1
0 -	YBL044W	-	-1	-1
0 -	YBL055C	-	-	-1
0 -	YBL059W	-	1	-
0 -	YBL071C-B	-	-1	1
0 -	YBL081W	-1	-	-
0 -	YBL100W-B	1	-	1
0 -	YBL107C	-	-	1
0 -	YBL113W-A	-	-1	-
0 -	YBR012W-A	1	-	-
0 -	YBR012W-B	1	-	1
0 -	YBR053C	1	-	1
0 -	YBR056W	1	1	1
0 -	YBR056W-A	1	-	-
0 -	YBR063C	1	1	-
0 -	YBR071W	1	-	-
0 -	YBR085C-A	1	1	-
0 -	YBR139W	1	1	1
0 -	YBR141C	-1	-	-
0 -	YBR196C-A	-1	-	-
0 -	YBR219C	-1	-1	-1
0 -	YBR220C	-1	-1	-1
0 -	YBR225W	-1	-	-
0 -	YBR230W-A	1	-	1
0 -	YBR238C	-	-	-1
0 -	YBR241C	1	1	1
0 -	YBR242W	-	-1	-1
0 -	YBR255C-A	-	-1	1
0 -	YBR259W	-	1	-
0 -	YBR285W	1	1	1
0 -	YBR287W	-	1	-
0 -	YCL002C	-1	-1	-1
0 -	YCL012C	-	1	-
0 -	YCL019W	1	-	1
0 -	YCL021W-A	-1	-	-
0 -	YCL049C	1	-	1
0 -	YCR007C	1	-	1
0 -	YCR015C	-1	-	-
0 -	YCR016W	-	-1	-1
0 -	YCR043C	-1	-	-
0 -	YCR051W	-	-1	-1
0 -	YCR061W	1	1	-
0 -	YCR075W-A	-	1	-
0 -	YCR087C-A	-	-	-1
0 -	YCR090C	-1	-	-
0 -	YCR099C	1	-	-
0 -	YCR100C	1	-	-
0 -	YDL027C	1	-	1

0	-	YDL063C	-	-	-1
0	-	YDL085C-A	1	-	-
0	-	YDL086W	1	1	1
0	-	YDL114W	-	1	1
0	-	YDL121C	-	-1	-1
0	-	YDL124W	-	1	-
0	-	YDL129W	-1	-	-
0	-	YDL144C	-	-	-1
0	-	YDL157C	1	-	-
0	-	YDL183C	-	1	1
0	-	YDL199C	1	1	1
0	-	YDL241W	-1	-1	-1
0	-	YDR003W-A	-	-	1
0	-	YDR018C	1	1	1
0	-	YDR034C-D	1	-	1
0	-	YDR034W-B	1	1	1
0	-	YDR061W	-1	-	-
0	-	YDR098C-A	1	-	-
0	-	YDR098C-B	1	-	1
0	-	YDR124W	-1	-	-1
0	-	YDR132C	-	-1	-
0	-	YDR161W	-1	-1	-1
0	-	YDR170W-A	1	1	-1
0	-	YDR182W-A	-1	-	-
0	-	YDR186C	1	-	-
0	-	YDR210C-C	1	-	-
0	-	YDR210C-D	1	-	1
0	-	YDR210W	-	-	-1
0	-	YDR210W-B	1	-	1
0	-	YDR222W	-1	-	-1
0	-	YDR246W-A	-1	-1	-
0	-	YDR248C	1	-	-
0	-	YDR249C	-1	-	-
0	-	YDR261C-C	1	-	-
0	-	YDR261C-D	1	-	1
0	-	YDR261W-B	1	-	1
0	-	YDR262W	-1	-	1
0	-	YDR282C	-	-	-1
0	-	YDR307W	1	-	1
0	-	YDR316W-A	1	-	-
0	-	YDR316W-B	1	-	1
0	-	YDR333C	-1	-	-
0	-	YDR365W-A	1	-	-
0	-	YDR365W-B	1	-	1
0	-	YDR379C-A	1	1	1
0	-	YDR381C-A	1	1	1
0	-	YDR391C	-1	-	-
0	-	YDR476C	1	-	-
0	-	YDR541C	-	-1	-
0	-	YDR545C-A	-	-1	-

0	-	YEL020C	-	1	-
0	-	YEL043W	-	1	-
0	-	YEL047C	1	1	1
1	**-**	**YEL057C**	1	1	-
0	-	YEL073C	1	-	1
0	-	YEL077W-A	-	-1	-
0	-	YER010C	-	-	1
0	-	YER034W	1	1	-
0	-	YER053C-A	-1	1	-
0	-	YER077C	-	-	-1
0	-	YER079W	1	-	1
0	-	YER130C	1	-	-
0	-	YER137C	-1	1	-
0	-	YER137C-A	1	-	-
0	-	YER138C	1	-	1
0	-	YER140W	-	-1	-1
0	-	YER152C	-	1	-
0	-	YER156C	-1	-1	-1
0	-	YER158C	1	1	1
0	-	YER159C-A	1	-	-
0	-	YER160C	1	-	1
0	-	YER163C	-	1	-
0	-	YER184C	1	-	-
0	-	YER186C	-1	-1	-
0	-	YER187W	-1	-1	-
0	-	YER188C-A	-	-1	-
0	-	YER190C-B	-	-1	-
0	-	YFL002W-A	1	-	1
0	-	YFL012W	1	1	1
0	-	YFL041W-A	-	-	1
0	-	YFL042C	1	1	-
0	-	YFL054C	1	1	-
0	-	YFL068W	-	-1	-
0	-	YFR012W-A	1	1	1
0	-	YFR016C	1	-	1
0	-	YFR018C	1	-	-
0	-	YFR039C	1	-	-
0	-	YGL006W-A	1	-	-
0	-	YGL010W	-	-	1
0	-	YGL036W	1	1	-
1	**-**	**YGL039W**	1	-	-1
0	-	YGL081W	-	1	1
0	-	YGL101W	-1	-1	-
0	-	YGL114W	1	-	-
0	-	YGL117W	1	1	1
0	-	YGL159W	-	-1	-
0	-	YGL176C	-1	-	-
0	-	YGL185C	-	1	-1
0	-	YGL230C	-	1	1
0	-	YGL242C	1	-	-

0	-	YGL258W-A	1	-	-
0	-	YGR012W	-	1	-
0	-	YGR021W	-	1	1
0	-	YGR026W	-	-	1
0	-	YGR027W-A	1	-	-
0	-	YGR027W-B	1	-	1
0	-	YGR035C	-1	-1	-1
0	-	YGR038C-A	1	-	-
0	-	YGR038C-B	1	-	1
1	**-**	**YGR042W**	-	-	1
0	-	YGR045C	1	-	-
0	-	YGR053C	1	-	1
0	-	YGR054W	-	-	-1
0	-	YGR067C	1	-	-
0	-	YGR079W	-1	-	-1
0	-	YGR093W	-	-	-1
0	-	YGR109W-A	-1	-1	-1
0	-	YGR109W-B	-1	-1	-1
0	-	YGR121W-A	-	-	-1
0	-	YGR122W	-1	-1	-1
0	-	YGR127W	-	1	1
0	-	YGR130C	1	1	1
0	-	YGR146C-A	-1	-	-
0	-	YGR149W	-	1	-
0	-	YGR153W	1	-	-
0	-	YGR161C-C	1	-	-
0	-	YGR161C-D	1	-	1
0	-	YGR161W-B	1	-	1
0	-	YGR174W-A	1	1	1
0	-	YGR210C	-1	-	-
0	-	YGR237C	-	-	1
0	-	YGR250C	1	1	1
0	-	YGR266W	-	-	-1
0	-	YGR283C	-1	-	-1
0	-	YGR296C-B	-	-1	-
0	-	YHL009W-B	1	-	-
1	**-**	**YHL012W**	1	-	-
0	-	YHL044W	-	-	1
0	-	YHL050W-A	-	-1	-
0	-	YHR003C	-1	-	-
0	-	YHR022C	-	1	-
0	-	YHR033W	-1	1	-
0	-	YHR045W	-	-1	-1
0	-	YHR078W	1	-	-
0	-	YHR080C	-	-	1
0	-	YHR097C	-	-	1
0	-	YHR112C	-	1	1
0	-	YHR122W	-	-	-1
0	-	YHR127W	-	-1	-
0	-	YHR138C	1	1	1

0 -	YHR140W	-	1	1
0 -	YHR182W	-	1	-
0 -	YHR214C-B	1	-	1
0 -	YHR214C-C	1	-	-
0 -	YHR214C-E	-1	-	-
0 -	YHR218W	-	-1	-1
0 -	YHR219C-A	-	-1	-
0 -	YHR219W	-	-1	-1
0 -	YIL014C-A	1	1	1
0 -	YIL024C	-	-	1
0 -	YIL029C	-	-	1
0 -	YIL055C	-	1	-
1 **-**	**YIL077C**	-	-	1
0 -	YIL082W	-1	-1	-1
0 -	YIL082W-A	-1	-1	-1
0 -	YIL096C	-	-	-1
0 -	YIL108W	1	1	1
0 -	YIL134C-A	-	1	-
0 -	YIL169C	-1	-	-1
0 -	YIL177C	-	-1	-1
0 -	YIL177W-A	-	-1	-
0 -	YIR007W	1	1	1
0 -	YIR018C-A	1	-	-
0 -	YIR024C	-	1	-
0 -	YIR035C	-	-	-1
0 -	YJL016W	1	1	1
0 -	YJL047C-A	-	1	-
0 -	YJL052C-A	-1	-	-
0 -	YJL055W	1	1	1
0 -	YJL070C	1	1	-
0 -	YJL077W-B	1	-	-
0 -	YJL113W	1	-	-
0 -	YJL127C-B	1	1	-
0 -	YJL133C-A	1	1	1
0 -	YJL144W	1	1	-
0 -	YJL160C	1	-	-
0 -	YJL181W	-1	-	1
0 -	YJL185C	1	-	-
0 -	YJL193W	-1	-	-
0 -	YJL213W	1	-	-
0 -	YJL218W	1	-	-1
0 -	YJL225C	-	-1	-1
0 -	YJL225W-A	-	-1	-
0 -	YJR003C	-	-1	-
0 -	YJR005C-A	1	-	1
0 -	YJR015W	-1	-1	-1
0 -	YJR026W	1	-	-
0 -	YJR027W	1	-	1
0 -	YJR028W	1	-	-
0 -	YJR029W	1	-	1

0 -	YJR056C	-	-	1
0 -	YJR061W	-	1	1
0 -	YJR085C	-	1	-
0 -	YJR096W	-	1	1
0 -	YJR098C	-1	-	-
0 -	YJR111C	-1	-	-
0 -	YJR114W	-	1	-
0 -	YJR115W	-	-	-1
0 -	YJR124C	-	-	-1
0 -	YJR129C	-	-	-1
0 -	YJR141W	-1	-	-
0 -	YJR149W	1	-	-
0 -	YKL023W	1	-	-
0 -	YKL027W	-1	-	-
0 -	YKL068W-A	-1	-	-
0 -	YKL069W	-	1	-
0 -	YKL071W	1	-	-
0 -	YKL091C	-	1	1
0 -	YKL105C	-	-	1
0 -	YKL107W	1	1	-
0 -	YKL151C	1	1	1
0 -	YKL187C	1	-1	-
0 -	YKL222C	1	-	-
0 -	YKR011C	1	-	1
0 -	YKR051W	-	-	1
0 -	YKR075C	-	-1	-
0 -	YKR096W	1	-	1
0 -	YLL053C	1	-	-1
0 -	YLL058W	1	-	-
0 -	YLL066C	-	-1	-1
0 -	YLL066W-A	-	-1	-
0 -	YLL066W-B	-1	-	-
0 -	YLL067C	-	-1	-1
0 -	YLL067W-A	-	-1	-
0 -	YLR001C	-	1	1
0 -	YLR030W	-	1	-
0 -	YLR031W	1	1	-
0 -	YLR035C-A	1	-	1
0 -	YLR040C	-1	-	-
0 -	YLR042C	-1	-1	1
0 -	YLR050C	1	1	-
0 -	YLR053C	1	1	1
0 -	YLR063W	-1	1	-1
0 -	YLR072W	-	1	-
0 -	YLR126C	1	-1	-1
0 -	YLR149C	1	1	1
0 -	YLR152C	1	-	-
0 -	YLR154W-E	-1	-	-
0 -	YLR157C-A	1	-	-
0 -	YLR157C-B	1	-	1

0	-	YLR173W	1	1	-
0	-	YLR177W	1	1	1
0	-	YLR194C	1	-	-
0	-	YLR211C	1	-	-
0	-	YLR227W-A	1	-	-
0	-	YLR227W-B	1	-	1
0	-	YLR243W	-1	-	-1
0	-	YLR253W	-1	-	-
0	-	YLR256W-A	1	-	-
0	-	YLR278C	1	1	1
0	-	YLR281C	-	-	1
0	-	YLR283W	-	-	1
0	-	YLR285C-A	-1	-	-1
0	-	YLR287C	-	-	-1
0	-	YLR290C	-	-	1
0	-	YLR297W	-	1	1
0	-	YLR326W	-1	1	1
0	-	YLR342W-A	-1	-	-
0	-	YLR345W	1	-	1
0	-	YLR352W	-	1	-
0	-	YLR363W-A	-	-	-1
0	-	YLR407W	-	1	1
0	-	YLR410W-B	1	-	1
0	-	YLR413W	-1	-	-
0	-	YLR446W	-	1	1
0	-	YLR455W	-1	-	1
0	-	YLR456W	-	-	1
0	-	YLR462W	-	-	-1
0	-	YLR466C-A	-	-1	-
0	-	YLR466W	-	-1	-1
0	-	YLR467C-A	-	-1	-
0	-	YLR467W	-	-1	-1
0	-	YML002W	1	-	-
0	-	YML007C-A	1	-	1
0	-	YML018C	-1	-	-1
0	-	YML037C	-1	-	-
0	-	YML039W	1	-	1
0	-	YML040W	1	-	-
0	-	YML045W	1	-	1
0	-	YML045W-A	1	-	-
0	-	YML083C	1	-	-
0	-	YML096W	-1	-1	-1
0	-	YML108W	-	-	-1
0	-	YML119W	-	-	1
0	-	YML133C	-	-1	-1
0	-	YML133W-B	-	-1	-
0	-	YMR013W-A	-1	1	-1
0	-	YMR018W	1	1	1
0	-	YMR034C	1	1	-
0	-	YMR045C	1	1	1

0 -	YMR046C	1	1	-1
0 -	YMR050C	1	-	1
0 -	YMR051C	1	-	-
0 -	YMR090W	-	1	1
0 -	YMR102C	-	-	-1
0 -	YMR114C	1	-	-
0 -	YMR122W-A	1	1	1
0 -	YMR124W	1	1	-
0 -	YMR130W	-	-	-1
0 -	YMR134W	1	-	-
0 -	YMR144W	-	-1	-
0 -	YMR166C	-	1	-
0 -	YMR181C	1	1	1
0 -	YMR185W	-	-	-1
0 -	YMR196W	1	1	1
0 -	YMR209C	-1	-	-
0 -	YMR210W	-	-	1
0 -	YMR230W-A	-1	-	-1
0 -	YMR244C-A	1	1	1
0 -	YMR244W	-	1	1
0 -	YMR252C	1	-	-
0 -	YMR265C	-	-	-1
0 -	YMR272W-B	-1	-	-
0 -	YMR310C	-	-	-1
0 -	YMR315W	1	-	-
0 -	YMR317W	1	-	-
0 -	YNL019C	-	-	-1
0 -	YNL022C	-1	-	-1
0 -	YNL024C	-	-1	-1
0 -	YNL033W	-	-	-1
0 -	YNL040W	-	-	-1
0 -	YNL042W-B	-1	-	-
0 -	YNL046W	-	-1	-
0 -	YNL054W-A	1	-	-
0 -	YNL095C	-	-	-1
0 -	YNL097C-B	-	-	-1
0 -	YNL108C	-	-	-1
0 -	YNL122C	-	1	1
0 -	YNL134C	-	-	1
0 -	YNL144C	1	-	1
0 -	YNL162W-A	-	-	-1
0 -	YNL195C	-	1	-
0 -	YNL200C	1	1	1
0 -	YNL208W	1	-	1
0 -	YNL217W	-1	-1	-
0 -	YNL234W	-	-	-1
0 -	YNL277W-A	-1	1	1
0 -	YNL284C-A	1	1	-1
0 -	YNL284C-B	1	1	1
0 -	YNL339W-B	-	-1	-

0	-	YNR014W	-	1	1
0	-	YNR034W-A	1	1	1
0	-	YNR040W	-	1	1
0	-	YNR048W	-1	-	-
0	-	YNR061C	-1	-1	-1
0	-	YNR062C	-1	-1	-1
0	-	YNR065C	1	-	-
0	-	YOL013W-A	-1	-	-
0	-	YOL014W	-	-1	-1
0	-	YOL036W	1	-	-
0	-	YOL057W	-	-1	-1
0	-	YOL092W	-	-	-1
0	-	YOL103W-A	1	-	-
0	-	YOL103W-B	1	-	1
0	-	YOL114C	1	-	1
0	-	YOL159C	-	1	-
0	-	YOL159C-A	-	1	-
0	-	YOL163W	1	-	-
0	-	YOR012W	1	-	-
0	-	YOR020W-A	1	-	1
0	-	YOR021C	-1	-1	-1
0	-	YOR052C	-	1	1
0	-	YOR059C	1	-	1
0	-	YOR062C	1	-	-
0	-	YOR072W-B	-1	-	-
0	-	YOR097C	-	-	1
0	-	YOR131C	-1	-	-
0	-	YOR142W-A	1	-	-
0	-	YOR142W-B	1	-	1
0	-	YOR152C	1	1	1
0	-	YOR186W	-	-	1
0	-	YOR192C-B	1	-	1
0	-	YOR262W	-	-	-1
0	-	YOR289W	1	1	1
0	-	YOR293C-A	-1	-	-
0	-	YOR296W	-1	-	1
0	-	YOR302W	1	1	-
0	-	YOR338W	-	1	-
0	-	YOR342C	-1	-	-
0	-	YOR343W-B	1	-	1
0	-	YOR378W	-1	-	-
0	-	YOR389W	1	1	1
0	-	YOR394C-A	1	-	1
0	-	YOR396C-A	-	-1	-
0	-	YPL014W	-	-	1
0	-	YPL067C	-	1	-
1	**-**	**YPL068C**	-	1	-1
0	-	YPL088W	1	1	-
0	-	YPL107W	-	-1	-1
0	-	YPL150W	1	-	-

0 -	YPL162C	-1	-	-1
0 -	YPL191C	1	-	-
0 -	YPL199C	-1	-1	-1
0 -	YPL216W	-	-	-1
0 -	YPL245W	-1	1	1
0 -	YPL257W-A	1	-	-
0 -	YPL257W-B	1	-	1
0 -	YPL264C	-	-	1
0 -	YPL272C	1	-	-
0 -	YPL277C	1	-	1
0 -	YPL278C	1	-	1
0 -	YPL283W-B	-	-1	-
0 -	YPR013C	1	-	-
0 -	YPR036W-A	1	1	1
0 -	YPR098C	-	-	1
0 -	YPR117W	1	1	1
0 -	YPR127W	1	1	1
0 -	YPR137C-A	1	-	-
0 -	YPR137C-B	1	-	1
0 -	YPR148C	1	-	-
0 -	YPR158C-C	1	-	-
0 -	YPR158C-D	1	-	1
0 -	YPR158W-A	1	-	-
0 -	YPR158W-B	1	-	1
0 -	YPR172W	-	1	-
0 -	YPR202W	-	-	-1
0 -	YPR203W	-	-	-1
0 -	YPR204C-A	-	-1	-
0 -	YPR204W	-	-1	-1
